# Identifying dendritic cell heterogeneity and potential risk genes in atopic dermatitis: integrative scRNA-seq, bulk RNA-seq analyses and experimental validation

**DOI:** 10.3389/fmed.2026.1882497

**Published:** 2026-08-26

**Authors:** Chushan Huang, Pengsheng Chen, Xiaosong Chen

**Affiliations:** 1 Department of Plastic Surgery and Regenerative Medicine, Fujian Medical University Union Hospital, Fujian Medical University, Fuzhou, China; 2 Department of Plastic Surgery and Regenerative Medicine Institute, Fujian Medical University, Fuzhou, China; 3 Engineering Research Center of Tissue and Organ Regeneration, Fujian Province University, Fuzhou, China; 4 Department of Dermatology, Fujian Medical University Union Hospital, Fujian Medical University, Fuzhou, China

**Keywords:** atopic dermatitis, CD1B, dendritic cells, gene expression, single-cell transcriptomics

## Abstract

Atopic dermatitis (AD) is a chronic inflammatory skin condition marked by immune dysregulation and compromised skin barrier function, with dendritic cells (DCs) playing pivotal roles in its pathogenesis. This study aimed to systematically characterize the heterogeneity of DCs in AD, identify key pathogenic subsets and genes, and elucidate their regulatory mechanisms through advanced methodologies. We conducted integrated single-cell RNA sequencing (scRNA-seq) analyses on both AD lesions and normal skin samples, leading to the identification and functional annotation of distinct DC subtypes. Through deconvolution, pseudotime trajectory, and co-expression network analyses, we screened critical cell populations and candidate genes. Causal associations were assessed using Mendelian randomization and colocalization analyses, while transcription factor activity inference, kNN-DREMI, and motif analysis were employed to explore underlying regulatory mechanisms. Our results revealed five distinct DC subsets, with a notable expansion of CD207^+^ migratory DCs $(\mathrm{CD}207_{m}\mathrm{DC})\text{inADlesi}$ ons, contrasting with the enrichment of IL1B^+^ DCs in normal skin. CD207_mDCs exhibited enhanced migratory and antigen-presenting capabilities, with CD1B being significantly upregulated and uniquely enriched within this subset. Integrative analyses suggest that CD1B may play a role in AD pathogenesis, potentially regulated by NOTCH1 signaling. These findings underscore the critical involvement of CD207_mDCs in AD progression and highlight CD1B as a promising candidate gene, implicating the NOTCH1–CD1B axis in Th2 polarization and presenting novel therapeutic targets for intervention in AD.

## 1 Introduction

Atopic dermatitis (AD) is a common inflammatory skin disease characterized by chronic relapsing inflammation and impaired skin barrier function (1, 2). Although the global age-standardized prevalence of AD has remained relatively stable in recent years, the absolute number of affected individuals continues to rise due to population growth. In many countries and regions, the disease burden of AD shows a significant upward trend (3). Clinically, AD primarily manifests as dry skin, intense itching, and recurrent inflammatory lesions, which severely impair patients’ quality of life (4). Extensive research indicates that AD progression is closely associated with aberrant immune activation, predominantly driven by Th2-type immune response characterized by sustained overexpression of cytokines such as IL-4, IL-5, and IL-13, which promote IgE production and perpetuate chronic inflammation (5). Beyond T cell immunity, research indicates that dendritic cells (DCs) also play a crucial role in the immune dysregulation of AD. DCs dysfunction can weaken antimicrobial defense and exacerbate inflammatory responses (6, 7). In contrast to T cells, however, research on DCs in AD remains relatively limited.

As key antigen-presenting cells bridging innate and adaptive immunity, DCs play a central regulatory role in immune imbalance in AD (8). DCs recognize pathogens, allergens, and tissue injury signals through multiple pattern recognition receptors, and their maturation and activation are critical steps in initiating T cell-mediated immune responses (9). Previous studies have shown that DCs are markedly increased in AD lesions and undergo phenotypic and functional reprogramming. This is primarily manifested as enhanced antigen-presenting capacity and elevated pro-inflammatory cytokine secretion, thereby sustaining and amplifying Th2-type immune responses (10, 11). However, existing studies have predominantly focused on the overall infiltration levels of DCs, with limited understanding of the heterogeneity among different DC subtypes and their functional transitions during disease progression.

The CD1 family comprises antigen-presenting molecules structurally similar to the major histocompatibility complex, playing a crucial role in the immune recognition of lipid and glycolipid antigens (12). Among them, CD1B is primarily expressed on professional antigen-presenting cells such as DCs, where it presents microbial or self-derived lipid antigens to activate specific T cell subtypes (12, 13). Although studies have suggested that CD1B^+^ DCs participate in local immune activation in skin diseases (14), their expression characteristics and functional roles in AD remain unclear.

Based on the above background, we employed single-cell transcriptomics (scRNA-seq) to systematically characterize the heterogeneity and functional reprogramming features of DC subtypes within AD lesions, exploring the association between distinct DC subtypes and AD. Subsequently, we identified AD-associated DC subtypes by deconvoluting bulk transcriptomic data. Furthermore, we employed pseudo-time analysis and high dimensional weighted gene co-expression network analysis (hdWGCNA) to infer candidate genes associated with AD risk. To further infer causal relationships between candidate genes and AD risk, we performed Mendelian randomization (MR) and colocalization analyses. Finally, through experimental validation, we identified CD1B as a candidate gene linked to AD risk. In summary, this study aimed to provide new insights for deepening the understanding of the immune pathogenesis of AD and developing precision treatment strategies.

## 2 Materials and methods

### 2.1 Data sources and preprocessing

We acquired scRNA-seq data from 5 AD and normal skin (NS) samples available in the GEO database (GSE269981) for comprehensive analysis. Data integration was performed using “seurat” package (v5.2.1). After quality control (genes/cell: 500–5,000; mitochondrial genes <15%; hemoglobin genes <1%; UMIs >1,000), doublets were removed using “DoubletFinder” (v2.0.4). Data were normalized with SCTransform, and highly variable genes (HVGs) were selected. Dimensionality reduction was performed using PCA (top 20 PCs), with batch correction via “Harmony” (v1.2.3), followed by UMAP visualization. Cell types were annotated automatically with “singleR” package (v2.4.0) and manually refined using established marker genes.

### 2.2 Functional enrichment analysis

We used “clusterProfiler” package (v4.0) to performed Kyoto Encyclopedia of Genes and Genomes (KEGG) pathway enrichment analysis. False Discovery Rate (FDR) <0.05 was set as statistically significant.

### 2.3 Bulk RNA-seq data and single-cell deconvolution

The AD-associated bulk RNA-seq dataset GSE120721 (22 NS, 15 non-lesions (NL), and 15 AD samples) was used for deconvolution analysis (15). Based on scRNA-seq–derived reference profiles, “BayesPrism” package (v2.2.2) was applied to estimate the proportion of DC subsets in bulk RNA-seq samples. A random forest classification model was constructed by using the inferred cell proportion matrix to evaluate the relative contribution of distinct DC subsets to AD pathogenesis, and feature importance was calculated using the “randomForest” package (v4.7.1.2).

### 2.4 Pseudotime trajectory analysis

“CytoTRACE” package (v0.3.3) was used to assess DC stemness and infer differentiation origins. Developmental trajectories were inferred in UMAP space using “slingshot” package (v2.14.0). “TradeSeq” package (v1.20.0) was applied to fit negative binomial generalized additive models along pseudotime, enabling characterization of dynamic gene expression changes. Statistical significance was assessed using “associationTest” (meanLogFC >0.5, *p* < 0.05) and “startVsEndTest” (|log2FC| >1, *p* < 0.05), and representative genes were visualized using “ggplot2” package.

### 2.5 hdWGCNA co-expression network analysis

We performed co-expression network analysis using “hdWGCNA” package (v0.3.0) (16). After hierarchical clustering of samples, a scale-free network (soft-thresholding power confirmed by scale-free topology fit >0.85) was constructed. The adjacency matrix was converted to a topological overlap matrix (TOM), and genes were clustered into modules using average-linkage hierarchical clustering. Module eigengenes (MEs) were calculated to assess correlations with cell subsets, and module membership (kME) was used to identify intramodular hub genes.

### 2.6 Mendelian randomization (MR) and Bayesian colocalization analysis

We obtained cis-eQTL data from the eQTLGen Consortium^1^ to serve as exposure data. Genetic variants associated with each candidate gene were selected as instrumental variables (IVs) from cis-eQTL data using a genome-wide significance threshold of *p* < 5 × 10^−8^. To ensure independence, SNPs were pruned based on linkage disequilibrium (LD) using an *r*^2^ < 0.01 within a 1,000 kb window. Instrument strength was evaluated using the F statistic, and SNPs with *F* < 10 were excluded to avoid weak instrument bias. The IVs were required to satisfy the following conditions: (1) strong association with the exposure (candidate gene expression); (2) independence from confounding factors; (3) association with the outcome (AD) only through the exposure. In addition, GWAS summary statistics for AD were obtained from the FinnGen database (release 12, 2023^2^), including 31,245 cases and 432,874 controls, and were used as the outcome data (finn-b-L12_ATOPIC). Subsequently, Harmonization of exposure and outcome datasets was performed using the “TwoSampleMR” package (v0.6.8). The inverse-variance weighted (IVW) method was used as the primary analysis to estimate the causal effect of changes in gene expression levels on AD risk. Genes with a potential causal effect were screened using a threshold of FDR < 0.05. To verify the robustness of the results, sensitivity analyses including MR-Egger and weighted median methods were also employed to assess and exclude potential biases from horizontal pleiotropy and heterogeneity on the causal estimates.

To determine whether the genetic regulatory signals of candidate genes and AD-GWAS signals share the same causal variant, we used “coloc” package (v5.2.3) to perform bayesian colocalization analysis. The analysis simultaneously incorporated eQTL data from the candidate gene region and AD-GWAS summary statistics. By comparing five possible hypotheses—(1) no association, (2) eQTL association only, (3) GWAS association only, (4) independent associations, and (5) a shared causal variant—we calculated the posterior probability for each scenario. We focused particularly on the posterior probability of the shared causal variant hypothesis (PP. H4), considering PP. H4 > 0.80 as strong evidence for colocalization.

### 2.7 DecoupleR regulatory network analysis

This study performed transcription factor (TF) activity inference using the “DecoupleR” package (v2.12.0) (17). Based on known TF–target gene regulatory relationships, a weighted GSEA (wGSEA) approach was applied to calculate TF activity scores in individual cells, thereby identifying key TFs significantly associated with specific cellular phenotypes.

### 2.8 kNN-DREMI analysis

K-nearest-neighbor density estimation with resampled mutual information (kNN-DREMI) was used to quantify dependencies between genes in scRNA-seq data (18). For each gene pair, the joint probability density was estimated on a fine grid (nGrid) using the kNN approach and then coarse-grained to a lower-resolution grid (nBin) to obtain a stable joint distribution. Joint probabilities were normalized to derive conditional probability densities, from which mutual information was computed to obtain the kNN-DREMI score, capturing directional dependence between genes. Parameters were set to k = 10, nBin = 20, and nGrid = 60. This analysis was implemented using the “scprep.stats.knnDREMI” function from the “scprep” package (v1.2.3) in Python.

### 2.9 Motif analysis and sequence logo visualization

To assess whether candidate regulatory regions contain RBPJ-binding motifs, the position weight matrix for the RBPJ (CSL) transcription factor (MA1116.1) was downloaded from JASPAR. The promoter sequence of CD1B was defined as the 2,000-bp region upstream of the transcription start site and retrieved using the UCSC Genome Browser. Using the MA1116.1 motif matrix, the promoter was scanned with FIMO to predict putative RBPJ-binding sites. Sequence logos were generated using “weblogo” package (v3.7.12) in Python to visualize concordance between predicted sites and the reference motif. By comparing target sequences with the reference RBPJ motif, we assessed enrichment of key nucleotides and evaluated whether predicted sites conform to the canonical RBPJ recognition pattern, thereby inferring the potential regulatory function of the region.

### 2.10 Human skin sample collection and clinical cohort characteristics

The study was approved by the ethics committee (2022KY184). All participants provided written informed consent for the collection of skin samples. Human skin samples were obtained from healthy individuals undergoing skin reduction surgery (mostly for aesthetic purposes), and in the form of biopsies, from lesional skin of patients with AD (2024.12–2026.03). All enrolled AD patients were clinically diagnosed by certified dermatologists adhering to the Williams’ Simplified Diagnostic Criteria (19) for AD and presented with active cutaneous lesions at sampling. A standardized pre-biopsy washout protocol was recommended for all participants, including a minimum 2-week discontinuation of topical treatments and a minimum 4-week discontinuation of systemic anti-inflammatory medications. Considering the real-world clinical scenario and ethical constraints for patients with moderate-to-severe AD, complete washout was not feasible in all cases. Only the six patients who fully satisfied the washout criteria were included for core molecular validation (*n* = 6). The baseline demographic and clinical characteristics of all enrolled participants are presented in Supplementary Table S1. Detailed information regarding previous treatments and individual medication washout status among AD patients is provided in Supplementary Table S2.

### 2.11 Immunohistochemistry (IHC)

All participants (6 patients with AD and 6 healthy controls) provided written informed consent for the collection of skin samples. Paraffin-embedded tissue slides underwent gradient hydration and antigen retrieval. After blocking with goat serum (BOSTER, AR0009), slides were incubated overnight at 4 °C with CD1B antibodies (Biorbyt, orb771547). Secondary antibodies were applied, followed by staining with 3,3′-diaminobenzidine (DAB) (Servicebio, G1212-200 T).

### 2.12 Quantitative reverse transcription polymerase chain reaction (qRT-PCR)

Total RNA was extracted from snap-frozen skin tissues obtained from six patients with AD and six healthy controls (TRIzol reagent, Beyotime, R0016) and reverse-transcribed into cDNA from 1 μg RNA using SuperScript III First-Strand Synthesis SuperMix (Thermo Fisher Scientific, 18,080–044). Quantitative PCR was performed with BeyoFast™ SYBR Green qPCR Mix on a QuantStudio 5 Real-Time PCR System (Applied Biosystems). Relative CD1B expression was normalized to GAPDH and calculated using the 2^−ΔΔCt method. Each biological sample was analyzed in triplicate. Primer sequences were as follows:

GAPDH forward, 5′-TCAAGATCATCAGCAATGCCTCC-3′;

GAPDH reverse, 5′-GCCATCACCCACCAGCATTC-3′;

CD1B forward, 5′-ATCTCTTGGGCGTCCTCAATGC-3′;

CD1B reverse, 5′-GGTAGAATCCTGAGACATGGCAC-3′.

### 2.13 Western blotting

Protein lysates were prepared from snap-frozen AD lesional skin tissues (*n* = 6) and healthy control skin tissues (*n* = 6) using RIPA buffer (PC102; Epizyme) supplemented with PMSF (G2008-1ML; Servicebio). Proteins were separated by SDS-PAGE (10%) and transferred onto PVDF membranes (WJ002; Epizyme). Membranes were blocked with 5% skimmed milk and incubated overnight with primary antibodies. After washing, secondary antibodies were applied, and signal detection was performed with the Enhanced Chemiluminescence Kit (SQ101; Epizyme).

### 2.14 Statistics

Statistical analyses were performed using R (version 4.2.2) and Python (v.3.7.8). A *p* value < 0.05 was considered statistically significant.

## 3 Results

### 3.1 scRNA-seq detects multiple cell types in atopic dermatitis (AD) and Normal skin (NS)

**Figure 1:**
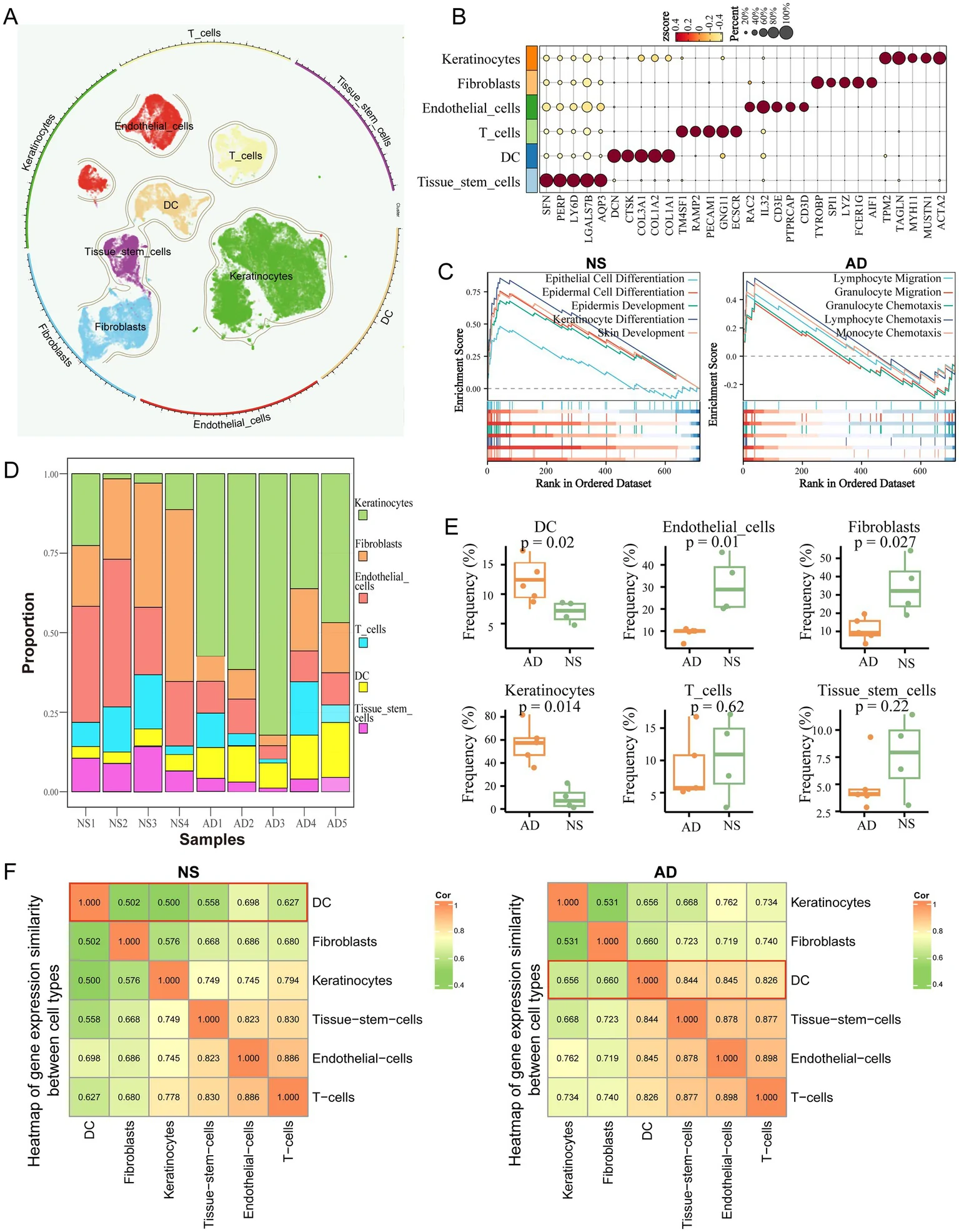
Single-cell RNA sequencing (scRNA-seq) detects multiple cell types in Atopic Dermatitis (AD) and Normal Skin (NS). **(A)** UMAP visualization of six cell types identified in AD and NS samples. **(B)** Cluster annotations using selected marker genes. Color intensity indicates scaled mean expression level. **(C)** Gene Set Enrichment Analysis (GSEA) based on DEGs. **(D)** Proportional distribution of major cell types in AD and NS samples. **(E)** Differences in cell proportions between AD and NS across various cell types. *p* < 0.05 indicates statistically significant differences. **(F)** Intercellular correlation analysis based on gene expression patterns.

After stringent quality control, a total of 71,303 high-quality cells were retained for downstream analyses. UMAP was used for dimensionality reduction and cluster visualization, depicting six cell types (Figures 1A,B): endothelial cells, T cells, dendritic cells (DCs), fibroblasts, keratinocytes, and tissue stem cells. Subsequently, differential expression analysis was performed on AD versus NS samples at the whole-cell level. This analysis identified a total of 186 upregulated and 541 downregulated differentially expressed genes (DEGs) (Supplementary Table S3; screening criteria: FDR < 0.05 and |Log2FC| >1). Functional enrichment analysis revealed that DEGs in the NS group were significantly enriched in pathways related to skin structure maintenance, barrier function, and tissue homeostasis (Figure 1C). In contrast, DEGs in the AD group exhibited characteristics of immune cell infiltration and activation of inflammatory pathways (Figure 1C). Cell proportion analysis further revealed significant differences in cellular composition between AD and NS samples (Figures 1D, E). Specifically, the proportions of DCs and keratinocytes were significantly increased in AD, whereas the proportions of endothelial cells and fibroblasts were notably decreased. Furthermore, intercellular correlation analysis based on gene expression patterns (Figure 1F) revealed that DCs in the AD group exhibited an abnormally active state compared to the NS group, with significantly enhanced interactions with other cell types.

### 3.2 DCs heterogeneity in AD

**Figure 2:**
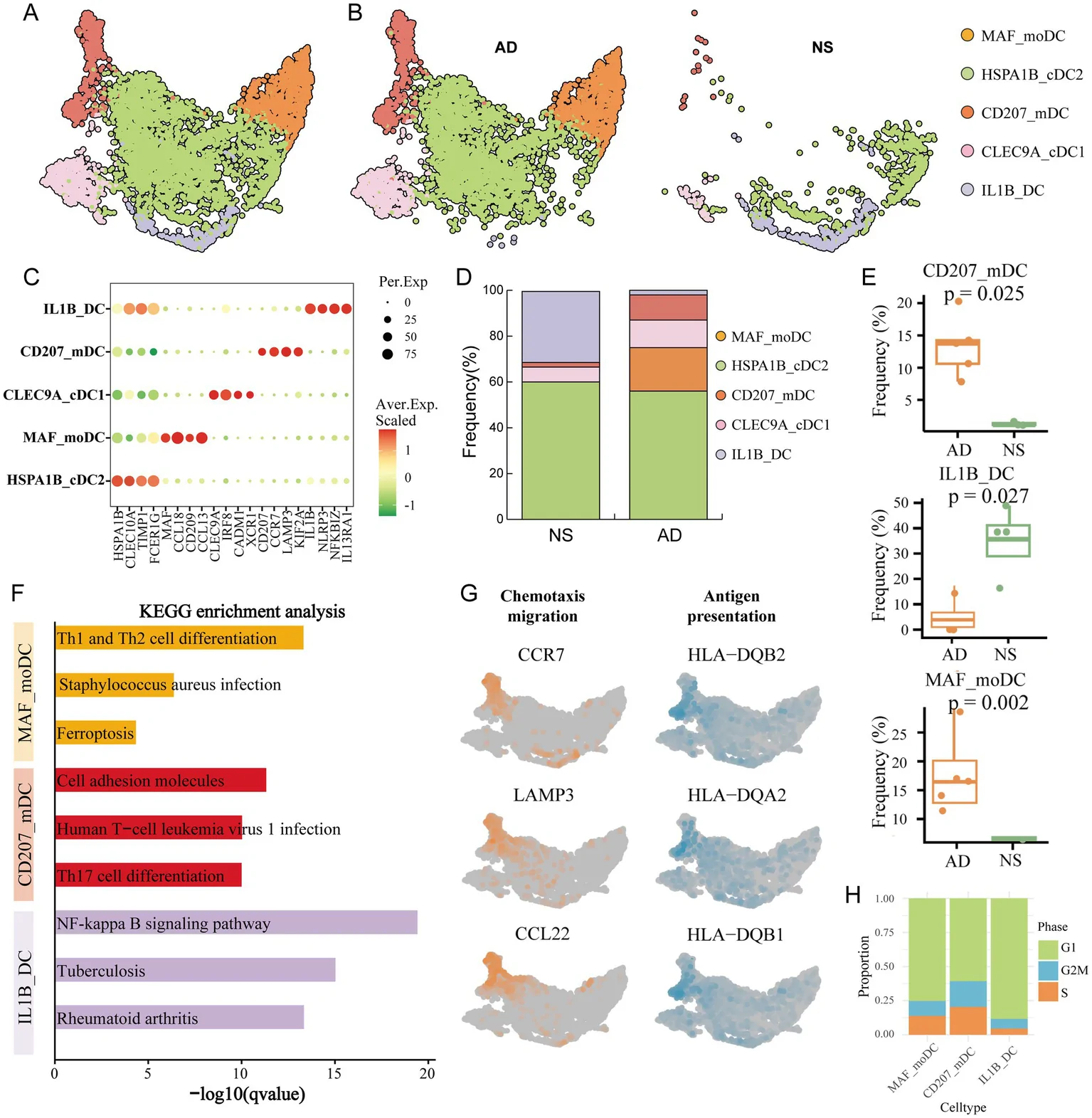
DCs heterogeneity in AD. **(A)** UMAP plot showed 5 subtypes of DCs. **(B)** UMAP plots showed the distribution differences between AD and NS in DC subtypes. **(C)** Marker genes of each subtypes visualized by dot plots. **(D)** Relative proportions of disease groups in each subtypes. **(E)** Box plots illustrated proportional differences of each DC subtypes between AD and NS. **(F)** Functional enrichment results for three key subtypes (MAF_moDC, CD207_mDC, IL1B_DC). **(G)** Expression distribution of representative genes on UMAP. **(H)** Cell cycle analysis showed cell cycle phase proportions (G1/S/G2M) for each subtypes.

Next, we further elucidated the heterogeneity of DCs. Five DC subtypes were identified (Figure 2A). Overall, DCs were significantly enriched in AD samples (Figure 2B). By integrating marker genes for DC subtypes reported in the literature (Supplementary Figure S1A), we identified one cDC1 subtypes (CLEC9A_cDC1), one cDC2 subtypes (HSPA1B_cDC2), one migratory subtypes (mDC: CD207_mDC), one monocyte-derived DC subtypes (moDC: MAF_moDC), and one poorly defined DC subtypes (IL1B_DC). To further elucidate the molecular characteristics and functional differences among DC subtypes, we integrated representative marker genes for each subtype (Supplementary Table S4). Figure 2C further visualizes the expression distribution of key marker genes.

Cell proportion analysis (Figure 2D) showed that MAF_moDC subtype was exclusively present in AD. Additionally, the proportions of CLEC9A_cDC1 and CD207_mDC were elevated in AD samples, whereas IL1B_DC was predominantly enriched in NS samples. Intergroup differential analysis revealed statistically significant differences in the proportions of MAF_moDC, CD207_mDC, and IL1B_DC (Figure 2E). Functional enrichment analysis demonstrated that MAF_moDC cells were enriched in Th1 and Th2 cell differentiation pathways; CD207_mDC primarily participated in cell adhesion molecules and T cell activation pathways; while IL1B_DC was significantly enriched in inflammation-related pathways (Figure 2F). At the gene expression level (Figure 2G; Supplementary Figure S1B), MAF, CCL18, and CCL2—closely associated with T cell differentiation—were highly expressed in MAF_moDC. CD207_mDC highly expressed genes associated with migration (CCR7, LAMP3, CCL22) and antigen presentation (HLA-DQB2, HLA-DQA2, HLA-DQB1); whereas inflammation-related molecules IL1B, BCL2A1, and NLRP3 were concentrated in IL1B_DC. These findings indicate significant heterogeneity among distinct DC subsets within the skin immune microenvironment. Cell cycle analysis (Figure 2H) provided further insights into their proliferative states: CD207_mDC had a higher proportion in the G2M and S phases, suggesting a potentially active proliferative state, whereas IL1B_DC was predominantly arrested in the G1 phase, indicating that most cells were quiescent or exhibit low proliferation.

### 3.3 Deconvolution analysis of bulk RNA-seq data identified CD207_mDC and IL1B_DC as key characteristic subtypes

Based on the AD scRNA-seq atlas constructed in this study, we performed deconvolution analysis on the GSE120721 bulk RNA-seq data (comprising 22 NS, 15 non-lesions (NL), and 15 AD samples). Cell proportion analysis after bulk deconvolution revealed a higher proportion of CD207_mDC in the AD group, while IL1B_DC constituted a larger proportion in both NS and NL groups (Supplementary Figure S1B). This finding aligns with scRNA-seq data (Figures 2D, E). Cell type correlation analysis revealed a significant negative correlation between CD207_mDC and IL1B_DC (Supplementary Figure S1C). Random forest analysis revealed that CD207_mDC and IL1B_DC showed significantly higher MeanDecreaseAccuracy and MeanDecreaseGini values compared to other subtypes (Supplementary Figure S1D), indicating these two subtypes are key characteristic subtypes for distinguishing different groups.

### 3.4 Differentiation trajectory and functional transition of key DC subtypes in AD

**Figure 3:**
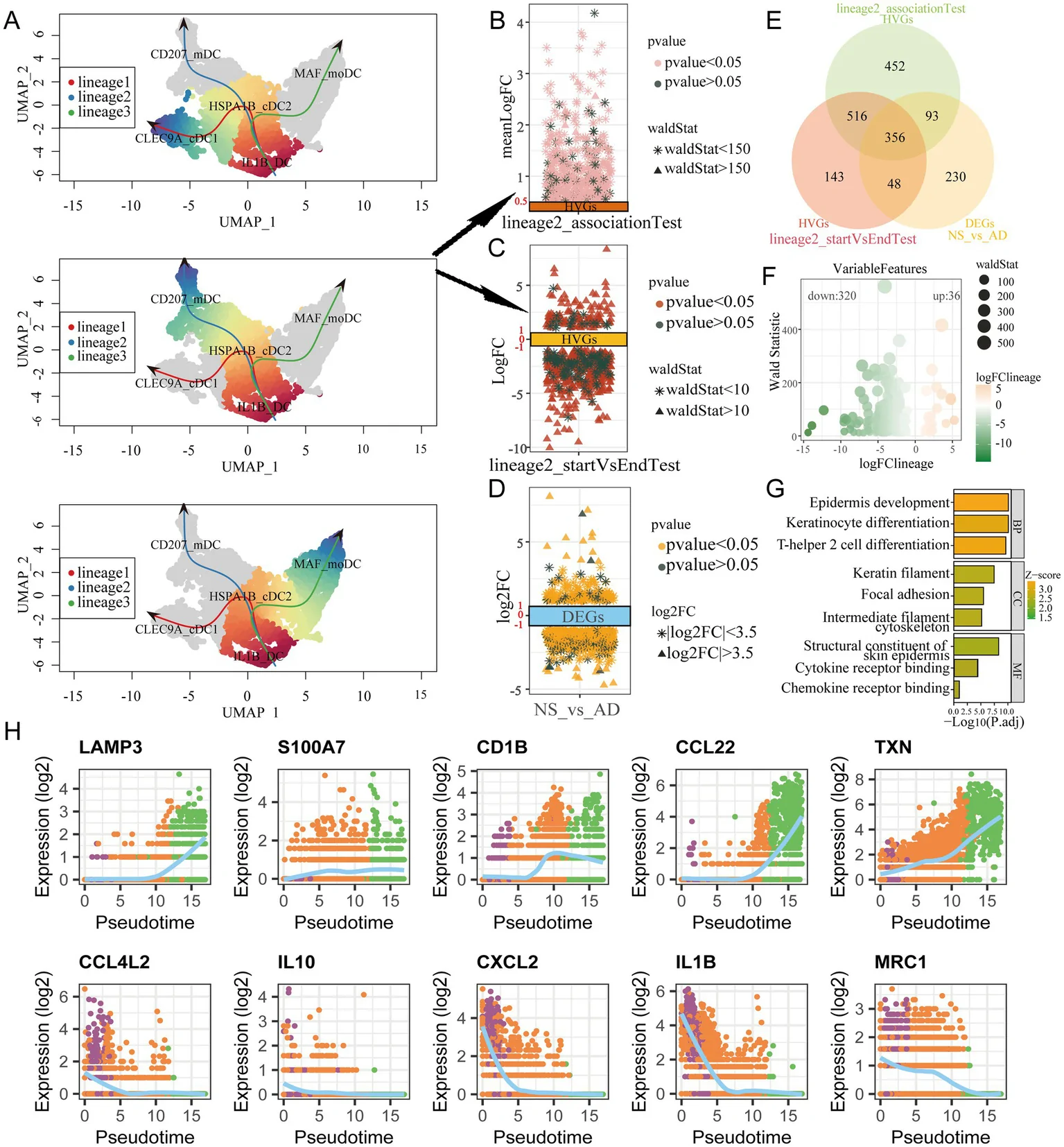
Deconvolution analysis of bulk RNA-seq data identified CD207_mDC and IL1B_DC as key characteristic subtypes. **(A)** Slingshot pseudotime analysis identified three DC differentiation trajectories. Among them, lineage 2 (IL1B_DC → HSPA1B_cDC2 → CD207_mDC) exhibited the most biologically meaningful continuous differentiation trajectory. **(B)** The Christmas tree plot visualized highly variable genes (HVGs) in lineage 2 identified by generalized additive model (GAM) analysis (associationTest) (meanLogFC >0.5, *p* < 0.05). **(C)** The Christmas tree plot visualized HVGs at the start and end points of lineage 2 differentiation (startVsEndTest) (|log_2_FC| >1, *p* < 0.05). **(D)** The Christmas tree plot visualized DEGs between NS and AD in DC cells. **(E)** Intersecting associationTest-HVGs, startVsEndTest-HVGs, and DEGs yields 356 key differential highly variable genes (DEHVGs) (|log2FC| >1, FDR < 0.05). **(F)** Volcano plot visualized 356 DEHVGs. Among them, 36 DEHVGs were upregulated at the differentiation endpoint (CD207_mDC) and 320 were upregulated at the differentiation starting point (IL1B_DC). **(G)** GO enrichment analysis results for DEHVGs. **(H)** Expression trends of key DEHVGs along the pseudo-temporal trajectory.

To investigate the differentiation trajectory and functional transition of DC subtypes, we performed pseudotime analysis. CytoTRACE analysis identified the IL1B_DC as the starting point of the differentiation trajectory (Supplementary Figure S1E). Slingshot analysis identified three distinct trajectories (Figure 3A). Integrating results from the previous deconvolution analysis (Supplementary Figures S1B–D), we focused on the trajectory of lineage2 (IL1B_DC → HSPA1B_cDC2 → CD207_mDC). To identify highly variable genes (HVGs) associated with the differentiation trajectory along lineage2, we performed the following two analytical methods: (1) The associationTest function was used to identify 1,417 HVGs that exhibited significant dynamic changes along the pseudotime trajectory (meanLogFC >0.5, *p* < 0.05) (Supplementary Table S5; Figure 3B). (2) The startVsEndTest function was applied to obtain 1,063 HVGs that showing significant expression differences between the trajectory start and end points (|Log2FC| >1, *p* < 0.05) (Supplementary Table S6; Figure 3C). Subsequently, DEGs analysis identified 727 DEGs between the NS and AD groups within the DCs (|Log2FC| >1, FDR < 0.05) (Supplementary Table S7; Figure 3D).

By taking the intersection of the HVGs and DEGs, we ultimately obtained a set of 356 differentially expressed highly variable genes (DEHVGs) (Figure 3E). DEHVGs comprised 36 upregulated genes (highly expressed in CD207_mDC) and 320 downregulated genes (highly expressed in IL1B_DC) (Figure 3F). DEHVGs not only dynamically change along differentiation trajectories but also exhibit significant expression differences across different groups. GO enrichment analysis revealed that DEHVGs were significantly enriched in pathways related to epidermis development, keratinocyte differentiation, Th2 cell differentiation (Figure 3G). This suggests that DCs differentiation reprogramming is closely associated with skin barrier impairment and the Th2 inflammatory microenvironment in AD. Finally, We visualized the top ten genes with the most significant differences in the lineage2 trajectory (Figure 3H). We found that upregulated genes (LAMP2, S100A7, CD1B, CCL12, TXN) were predominantly associated with antigen processing and presentation and cell migration, while downregulated genes (CCL4L2, IL10, CXCL2, IL1B, MRC1) were predominantly associated with inflammatory responses.

### 3.5 CD1B was identified as a candidate risk-associated gene for AD

**Figure 4:**
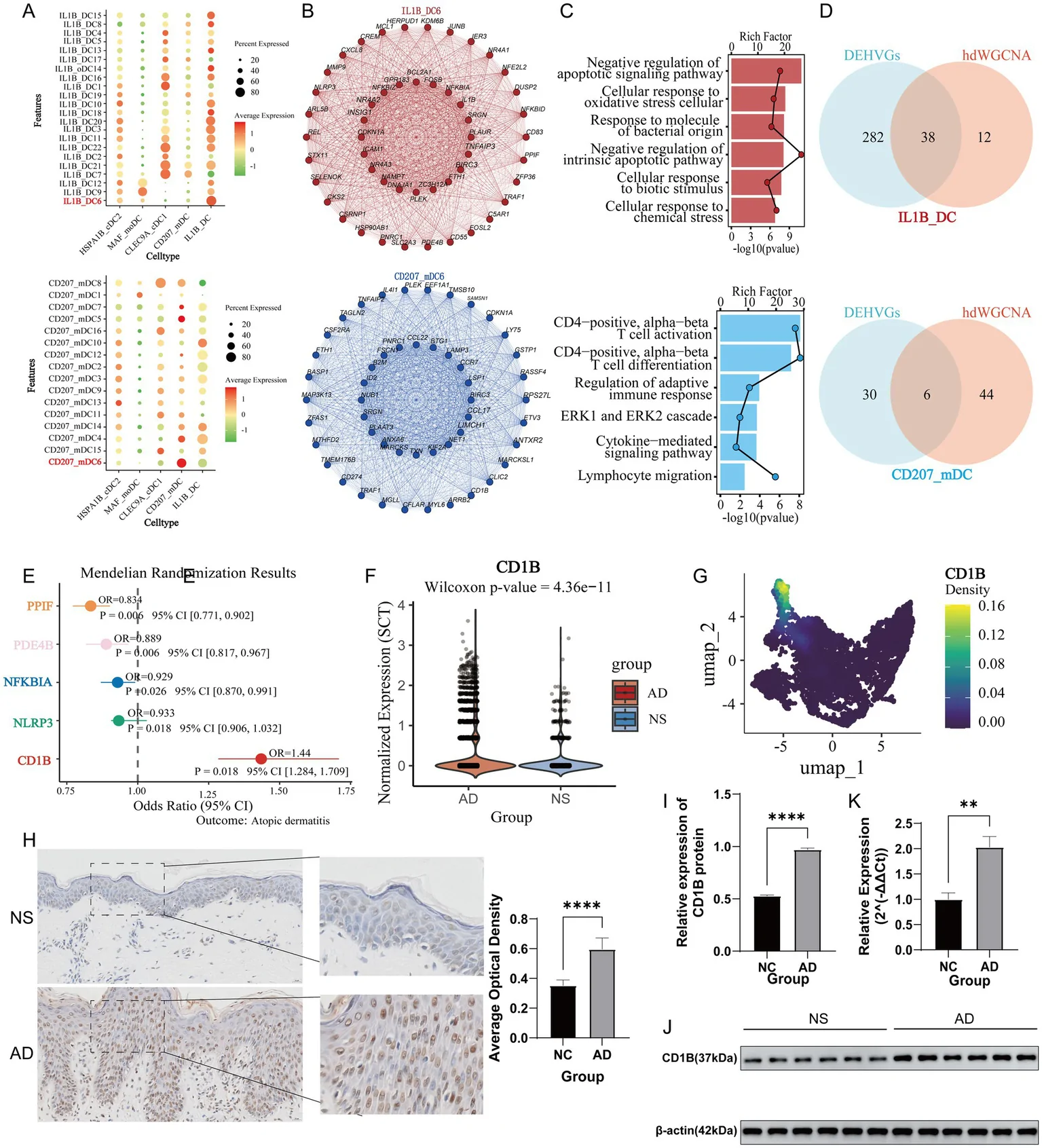
CD1B was identified as a candidate risk-associated gene for AD. **(A)** High-dimensional Weighted Gene Co-expression Network Analysis (hdWGCNA) of the IL1B_DC and CD207_mDC subtypes identified multiple stable gene modules. Module–trait correlation analysis revealed the key core modules associated with the IL1B_DC (top panel) and CD207_mDC (bottom panel) subtypes. **(B)** Screening the top 50 hub genes in the IL1B_DC6 and CD207_mDC6 modules based on node connectivity (kME), and constructing their co-expression regulatory networks: Upper panel (red): IL1B_DC6 hub genes; bottom panel (blue): CD207_mDC6 hub genes. **(C)** GO enrichment analysis of hub genes in the core modules. **(D)** Intersection of pseudotime-derived DEHVGs with core module hub genes yielded 44 key candidate genes (38 overlapping genes in IL1B_DC subtype; 6 genes in the CD207_mDC subtype). **(E)** Mendelian randomization (MR) analysis identified candidate genes with potential causal effects on AD. **(F)** Differential expression of CD1B in AD and NS groups. **(G)** Representative immunohistochemical staining and quantitative analysis of CD1B expression in skin tissues from six patients with AD and six healthy controls. **(H)** Quantitative analysis of average optical density (AOD) from immunohistochemical results demonstrates significantly higher CD1B expression levels in the AD group (*n* = 6) compared to the NS group (*n* = 6). **(I)** Western blot analysis and quantification of CD1B protein expression in six AD lesional skin samples and six healthy control skin samples. **(K)** qRT-PCR analysis revealed CD1B mRNA expression levels in NS and AD tissue. Data are presented as mean ± standard deviation. **p < 0.01, ****p < 0.0001.

To identify distinct functional modules in the IL1B_DC and CD207_mDC, we performed hdWGCNA analysis (Supplementary Figure S2A). Multiple stable functional modules were identified in both subtypes. Among these, IL1B_DC6 and CD207_mDC6 were identified as the core modules for IL1B_DC and CD207_mDC, respectively (Figure 4A). By extracting the top 50 hub genes with the highest connectivity (kME) within each core module, we constructed gene regulatory networks (Figure 4B). GO enrichment analysis revealed that IL1B_DC hub genes primarily participated in pathways such as negative regulation of apoptosis, oxidative stress. CD207_mDC hub genes were significantly enriched in pathways including T cell activation and differentiation, and cell migration (Figure 4C). We intersected the DEHVGs of the subtypes with the hub genes of the core modules (Figure 4D), ultimately identifying 44 key candidate genes (38 of which were highly expressed in IL1B_DC and 6 in CD207_mDC). These genes simultaneously met three criteria: differential expression, dynamic changes along differentiation trajectories, and hub gene of core modules, suggesting they may be key candidate genes for AD progression.

To assess the potential causal relationship between these candidate genes and AD, we further performed MR analysis. Results indicated that four genes (PP1R4, PDE4B, NFKBIA, NLRP3) showed protective effects (OR < 1), while CD1B was a significant risk gene (OR > 1) (Figure 4E; Supplementary Table S8). Subsequent colocalization analysis revealed that only CD1B shared a causal locus with AD GWAS-signals (PP. H4 > 0.80) (Supplementary Table S9). In scRNA-seq data, CD1B expression was significantly upregulated in AD samples (Figure 4F) and specifically enriched in the CD207_mDC subpopulation (Figure 4G). External data (GSE120721) also suggested high expression of CD1B in AD (Figure 4H). Furthermore, IHC showed significantly increased expression of CD1B protein in AD (Figures 4J,K), with similar findings observed in WB (Figures 4I,J). At the mRNA level, qPCR also showed higher expression of the CD1B gene in AD (Figure 4K). In summary, through multi-omics integration analysis, this study supported CD1B as a marker gene of the CD207_mDC and suggested that CD1B may represent a candidate risk gene for AD.

### 3.6 Potential NOTCH1-CD1B regulatory relationship in AD

**Figure 5:**
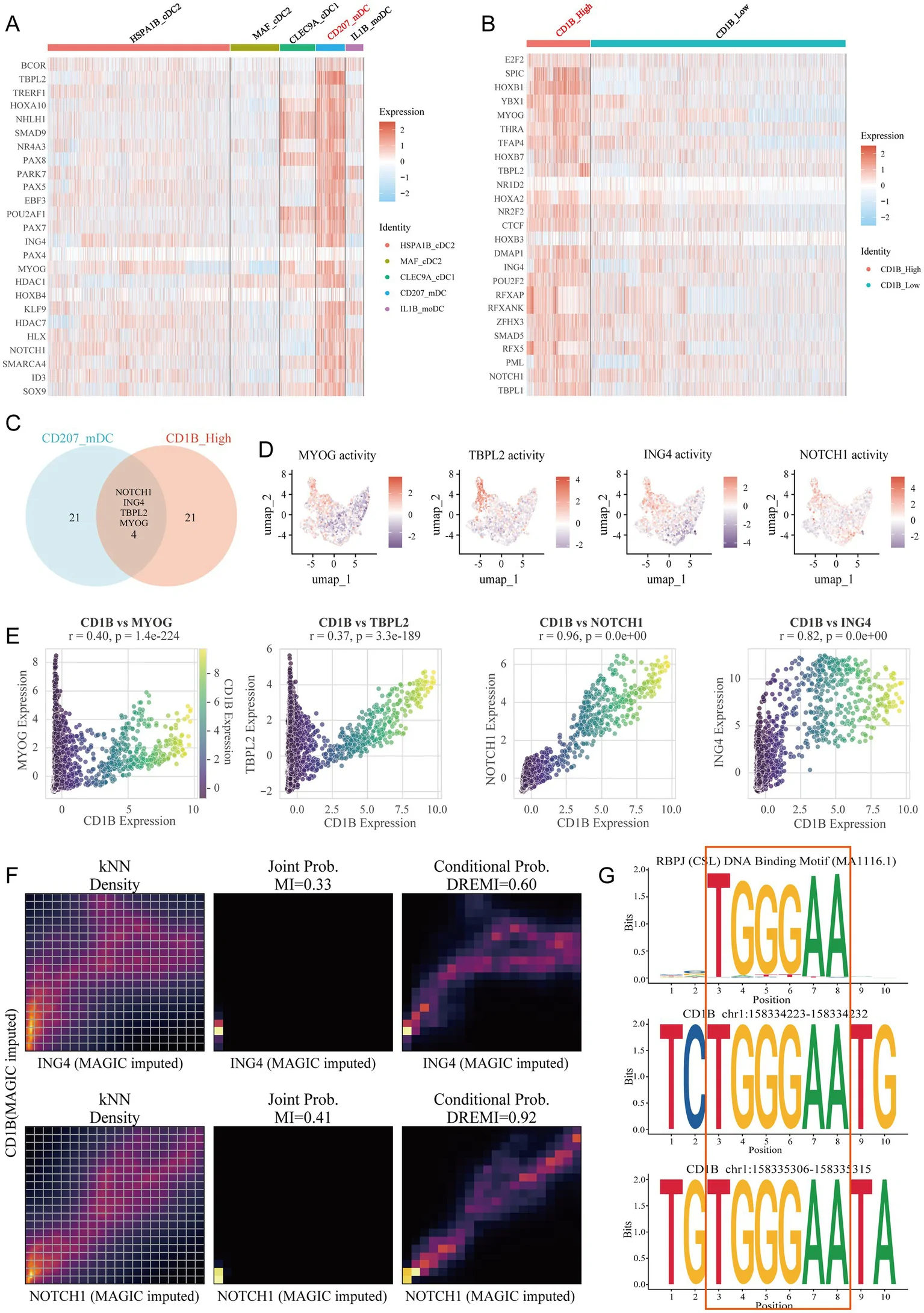
Potential NOTCH1-CD1B regulatory relationship in AD. **(A)** Heatmap displayed the expression activity of key transcription factors (TFs) in the CD207_mDC subtype. **(B)** After subdividing DC cells into CD1B_High and CD1B_Low subtypes based on CD1B expression, comparing the activity differences of key TFs between the two subtypes. **(C)** Venn diagram showed the intersection of the top 25 most active transcription factors in CD207_mDC and CD1B_High, yielding 4 core candidate TFs (NOTCH1, ING4, TBPL2, MYOG). **(D)** UMAP visualized the activity distribution of the four core TFs. **(E)** Scatter plots demonstrated the expression correlations between CD1B and MYOG, TBPL2, NOTCH1, and ING4. **(F)** Conditional dependency analysis based on MAGIC-interpolated data. Left: KNN density distribution; Center: Joint probability and mutual information (MI); Right: Conditional probability and DREMI value. **(G)** RBPJ (CSL) binding motif near the CD1B genomic promoter.

This study further explored the potential mechanisms of CD1B in the CD207_mDC subtype. TF activity analysis identified active modules in CD207_mDC (Figure 5A) and CD1B-high DCs (CD1B+ DCs) (Figure 5B), respectively. We intersected the top 25 most active TFs in both CD207_mDC and CD1B-high subtypes (Figure 5C), identifying four candidate TFs (MYOG, TBPL2, ING4, NOTCH1). These may represent potential regulators of CD1B. Subsequently, we visualized the activity distribution of these TFs in DCs (Figure 5D), revealing that all exhibited the strongest activity in the CD207_mDC, closely matching the expression distribution of CD1B.

Next, we assessed co-expression relationships between CD1B and candidate TFs (Figure 5E). ING4 (*r* = 0.82) and NOTCH1 (*r* = 0.96) exhibited nearly linear positive correlations with CD1B, suggesting a potential regulatory association with CD1B. To further validate the relationship between CD1B and these key TFs, we constructed DREMI scoring models based on MAGIC interpolation method (Figure 5F). Among them, NOTCH1-CD1B showed the highest DREMI score (0.92), suggesting that NOTCH1 may act as a potential upstream regulator of CD1B. Further motif analysis revealed (Figure 5G) binding sequences for the RBPJ/CSL TF within the CD1B gene promoter region. RBPJ/CSL is a key downstream effector molecule of the NOTCH1 signaling pathway. This further supports the potential role of NOTCH1 in regulating CD1B function. Moreover, high CD1B expression showed significant positive correlations with Th2 chemokines (CCL22, CCL17) and Th2 signaling-associated molecules (IL4R, JAK3) (Supplementary Figure S2C), suggesting CD1B+ DCs may drive Th2-type inflammation in AD. Notably, high NOTCH1 expression further enhanced the co-expression relationship between CD1B and CCL22 (Supplementary Figure S2D). This indicates that NOTCH1 may enhance the co-expression relationship between CD1B and CCL22.

## 4 Discussion

Although AD has been extensively studied, the key cellular populations and regulatory networks driving its chronic progression remain incompletely understood. Through a systematic analysis of scRNA-seq data from AD and NS, we uncovered pronounced heterogeneity among DCs. Among these, CD207_mDC was identified as a key characteristic subtype in AD. Notably, we found that CD1B, highly expressed in CD207_mDC, is a candidate gene associated with AD risk, providing new insights into the progression of AD.

DCs serve as key initiators of the cutaneous immune system and play a crucial role in antigen presentation and T cell immune regulation (6, 7). Accumulating evidence has demonstrated that DCs are key drivers of Th2-type immune responses in AD (11, 20, 21). In lesional skin from patients with AD, DC numbers are significantly increased in both the epidermis and dermis (22). Consistent with previous studies, this study observed a significant increase in the proportion of DCs in AD. Furthermore, DCs in the AD group exhibited stronger interactions with T cells. Banfield et al. (8) similarly found that DCs in AD enhanced their connection with T cells. Additionally, existing research indicates that a CD123+ DC subtype in AD is particularly adept at promoting Th2 polarization, thereby exacerbating disease progression. In short, DCs constitute a central immunological hub for initiating and sustaining Th2-type immune responses in AD. Therefore, targeting DCs function has emerged as a promising therapeutic strategy. Recent pharmacological studies have shown that dupilumab ameliorates AD inflammation by inducing DCs apoptosis and suppressing the activity of myeloid DCs. In addition, inhibitors of the OX40/OX40L pathway block DC-mediated costimulatory signaling to T cells by downregulating OX40L expression on DCs, offering another promising avenue for immunological intervention in AD (23).

This study revealed pronounced functional heterogeneity among DCs in AD. MAF_moDC and CD207_mDC were enriched in AD: the former is driven by MAF/MAFB and upregulates multiple chemokines, while the latter is more closely associated with cell migration and antigen presentation. In contrast, IL1B_DC were predominantly distributed in NS, more closely resembling sentinel DCs responsible for maintaining tissue homeostasis (24). These findings indicated that different DC subtypes not only show significant differences in cellular proportions but also perform different functions in AD progression. Early investigations based on flow cytometry and histological analyses identified multiple DC subtypes in AD, including langerhans cells, dermal DCs, and inflammatory dendritic epidermal cells (IDECs), each exerting distinct roles in antigen presentation, TLR-mediated inflammatory amplification, and Th2 polarization (25, 26). For example, IDECs massively infiltrated lesional sites and highly expressed FcεRI and TLR2, rapidly upregulating costimulatory molecules and releasing inflammatory mediators (27). Meanwhile, Langerin-positive dermal DCs have been demonstrated to promote thymic stromal lymphopoietin (TSLP) production and drive cutaneous inflammation (20, 28). In addition, both myeloid and plasmacytoid DCs in the peripheral blood of patients with AD exhibited marked numerical and functional imbalance (29, 30). Collectively, these diverse DC subtypes formed a complex immunological network in AD, providing multilayered regulatory nodes that govern disease onset and progression.

Among all DC subtypes identified in this study, CD207_mDC is of particular interest. This subtype was markedly enriched in AD and exhibited high expression of migration-associated genes (CCR7 and LAMP3) and antigen-presenting genes (CD207). Additionally, CD207_mDC also secrete the Th2 chemokine CCL22. High expression of LAMP3 and CCR7 is a hallmark of mature DCs and suggests a strong capacity for lymph node migration, consistent with previously reported LAMP3+CCR7+ DC populations (26, 31, 32). Multi-omics studies have further shown that LAMP3+CCR7+ DCs co-localize with CCL19-secreting COL18A1+ fibroblasts in leukocyte-infiltrated regions of AD lesions, forming functional units within the local inflammatory microenvironment (31). Notably, CD207_mDC also highly express CD207, a canonical marker of langerhans cells (26). CD207 plays a critical role in antigen recognition, capture, and endocytosis (33). In murine models of AD, CD207-expressing langerhans cells transport antigens from the skin to lymph nodes and mediate Th2 immune responses via FcεRI, thereby completing key steps in the sensitization process (33). Furthermore, CD207_mDC shows marked upregulation of CCL22, whose expression levels positively correlate with AD severity (34). As a chemokine, CCL22 plays a central role in recruiting CCR4+ Th2 cells to inflamed skin and in sustaining and amplifying Th2-type immune responses (35). Collectively, CD207_mDC integrate migratory maturation, antigen presentation, and Th2 cell recruitment, representing a DC subset of high pathological relevance in AD and a potential target for future therapeutic intervention.

In addition to the expansion of CD207_mDC subtype, our data suggest that AD may also involve impaired homeostatic immune regulation. IL1B_DC was predominantly enriched inNS but reduced in AD lesions. The reduction of IL1B_DC was accompanied by decreased expression of several immune regulatory genes. Among these genes, IL10 is of particular relevance because IL-10 limits excessive immune activation and maintains tissue immune homeostasis (36). In addition, reduced MRC1 expression may reflect changes in tissue repair and immune regulatory features (37). Previous studies have demonstrated that AD is characterized not only by increased type 2 inflammation but also by impaired innate immune regulation and host defense mechanisms (38). Therefore, the reduction of IL1B_DC-associated homeostatic signatures may represent an additional mechanism contributing to immune imbalance in AD.

This study further identifies CD1B as a key pathogenic gene in AD. CD1B is markedly upregulated in AD and is predominantly expressed in CD207_mDC. As a non-classical MHC class I–like molecule, CD1B primarily functions in the presentation of lipid antigens to T cells (39). In the context of AD, this function may amplify self or microbial lipid signals exposed by skin barrier disruption, aberrantly activating Th2/Th17 immune responses and sustaining the release of inflammatory cytokines such as IL-4, IL-13, and IL-17, ultimately leading to chronic inflammation (14, 40). We further explored the potential regulatory relationship between NOTCH1 and CD1B. Motif analysis identified putative RBPJ-binding sites within the regulatory region of CD1B, suggesting a possible regulatory interaction mediated by the NOTCH1/RBPJ pathway (41). In keratinocytes, the loss of NOTCH1 expression impairs the skin barrier and induces TSLP expression, thereby exacerbating inflammation (42), whereas NOTCH1 signaling is enhanced in DCs (43, 44). Consistently, we observed elevated NOTCH1 signaling in CD207_mDC. Given that NOTCH1 is a key driver of Th2 differentiation, we propose that enhanced NOTCH1 signaling may upregulate CD1B via RBPJ, which may potentially influence lipid antigen presentation by DCs. Thus, CD1B may represent a potential molecular node linking specific DC subtypes, lipid antigen presentation, and aberrant T-cell immune responses.

In our study, there are several limitations. First, although we experimentally validated the differential expression of CD1B in AD, its specific functional mechanism requires further analysis. Second, this study has not yet systematically elucidated the interactions between DCs and other cell populations, which deserves more in-depth exploration in future studies. Third, MR analysis has several inherent limitations. Although strict instrumental variable selection criteria and sensitivity analyses were applied, the core MR assumptions cannot be completely guaranteed, and residual pleiotropy or heterogeneity may still influence the causal estimates. Fourth, due to limitations inherent in the real-world clinical setting within the clinical validation cohort, it was not possible to implement a strictly standardized drug-eluting period for all enrolled patients. Although we obtained six core validation samples meeting the elution period criteria, this sample size was only sufficient for preliminary verification of protein expression differences and insufficient for comprehensive multivariate adjustment or subgroup stratified analysis. Future studies should expand the sample size in prospective cohorts and standardize the drug-eluting regimen design to eliminate treatment confounding effects. Thus, the MR results should be interpreted with caution.

## 5 Conclusion

In summary, this study systematically analyzed the heterogeneity and functional characteristics of DC subtypes in AD based on scRNA-seq, revealing a significant expansion of CD207_mDC in AD. Further analysis demonstrated that CD1B was not only specifically overexpressed in CD207_mDC but also served as a candidate gene associated with AD risk. These findings provided new evidence for the immunopathological mechanisms underlying AD.

## Data Availability

The original contributions presented in the study are included in the article and its Supplementary material. The datasets analyzed in this study are publicly available. The single-cell RNA-seq dataset (GSE269981) and bulk RNA-seq dataset (GSE120721) can be accessed from the GEO database. GWAS summary statistics were obtained from the FinnGen database, and eQTL data were obtained from the eQTLGen Consortium.

## Footnotes

1
https://eqtlgen.org/

2
https://www.finngen.fi/en
