# Supplementary material for: Identifying dendritic cell heterogeneity and potential risk genes in atopic dermatitis: integrative scRNA-seq, bulk RNA-seq analyses and experimental validation
Source: Front Med (Lausanne). 2026 Aug 26;13:1882497. doi: 10.3389/fmed.2026.1882497 (PMC13553427; doi:10.3389/fmed.2026.1882497)
Supplement: Supplementary file 1 [file Table_1.docx]

**Table S1. Clinical data summary**

| **Characteristic*** | **HC(n=6)** | **AD(n=6)** | **Total(n=12)** |
| --- | --- | --- | --- |
| Age, years:  Mean (range)  Median (IQR) | 39.5 (25–55)  40.5 (31.0–45.0) | 53.17 (22–65)58.0 (52.0–64.0) | 46.33 (22–65)  48.5 (31.0–55.0) |
| Male: No (%) | 2 (33.33%) | 3 (50.00%) | 5 (41.67%) |
| Female: No (%) | 4 (66.67%) | 3 (50.00%) | 7 (58.33%) |
| EASI score:  Mean (range)  Median (IQR) | - | 28.42 (19.50–38.80)28.25 (27.30–28.40) | - |

*calculated from data available in Table E2.

EASI: Eczema Area and Severity Index

**Table S2. Demographics of healthy individuals and AD patients.**

| ID | Age (y.) | Gender | Harvest site | Experimental Cohort |
| --- | --- | --- | --- | --- |
| N1 | 45 | F | Abdomen | IHC/RT-PCR/WB |
| N2 | 44 | F | Abdomen | IHC/RT-PCR/WB |
| N3 | 37 | F | Abdomen | IHC/RT-PCR/WB |
| N4 | 31 | M | Breast | IHC/RT-PCR/WB |
| N5 | 25 | M | Breast | IHC/RT-PCR/WB |
| N6 | 55 | F | Abdomen | IHC/RT-PCR/WB |

| ID | Age (y.) | Gender | EASI | Biopsy site | Prior treatment | Met washout criteria | Experimental Cohort |
| --- | --- | --- | --- | --- | --- | --- | --- |
| A1 | 22 | M | 28.2 | Left abdomen | Topical Mometasone furoate cream, Mupirocin ointment | Yes | HE/IHC/RT-PCR/WB |
| A2 | 52 | F | 28.3 | Upper chest | Anti-allergic treatment and topical anti-inflammatory creams at other hospitals | Yes | HE/IHC/RT-PCR/WB |
| A3 | 64 | M | 38.8 | Right arm | Treated with Chinese medicine at other Hospital (details unknown) | Yes | HE/IHC/RT-PCR/WB |
| A4 | 65 | F | 27.3 | Right forearm | Ebastine, pregabalin, and topical mometasone showed limited efficacy | Yes | HE/IHC/RT-PCR/WB |
| A5 | 55 | F | 28.4 | Left lower leg | Calamine lotion, fusidic acid, halcinonide solution, etc | Yes | HE/IHC/RT-PCR/WB |
| A6 | 61 | M | 19.5 | Neck | Topical agents and symptomatic antipruritic treatment with limited efficacy | Yes | HE/IHC/RT-PCR/WB |

EASI: Eczema Area and Severity Index

NA: not available

| **Table S3. Differential expression analysis between atopic dermatitis and normal skin samples** | | | | | | |
| --- | --- | --- | --- | --- | --- | --- |
| **Gene** | **pvalue** | **Log₂FC** | **pct.1** | **pct.2** | **FDR** | **Cluster** |
| CCL3L1 | 6.85e-125 | -4.723 | 0.01 | 0.162 | 1.69e-120 | NS |
| CCL4L2 | 1.43e-198 | -4.591 | 0.033 | 0.313 | 3.52e-194 | NS |
| LRMDA | 6.94e-101 | -4.248 | 0.006 | 0.125 | 1.71e-96 | NS |
| CCDC85B | 4.79e-224 | -4.131 | 0.018 | 0.289 | 1.18e-219 | NS |
| CHCHD10 | 1.77e-270 | -3.844 | 0.036 | 0.392 | 4.38e-266 | NS |
| C4orf48 | 1.46e-189 | -3.817 | 0.02 | 0.264 | 3.60e-185 | NS |
| NDUFAF8 | 9.06e-77 | -3.761 | 0.009 | 0.111 | 2.24e-72 | NS |
| ATP5F1D | 6.49e-306 | -3.67 | 0.045 | 0.454 | 1.60e-301 | NS |
| AL135905.2 | 8.54e-93 | -3.646 | 0.01 | 0.131 | 2.11e-88 | NS |
| NENF | 1.46e-130 | -3.618 | 0.016 | 0.192 | 3.59e-126 | NS |
| IL1B | 6.73e-206 | -3.609 | 0.144 | 0.534 | 1.66e-201 | NS |
| NLRP3 | 1.35e-200 | -3.555 | 0.053 | 0.371 | 3.32e-196 | NS |
| CXCL3 | 1.73e-181 | -3.514 | 0.101 | 0.446 | 4.27e-177 | NS |
| TMEM160 | 1.71e-118 | -3.508 | 0.017 | 0.183 | 4.21e-114 | NS |
| CFD | 4.00e-158 | -3.284 | 0.072 | 0.368 | 9.87e-154 | NS |
| PID1 | 4.19e-156 | -3.178 | 0.04 | 0.292 | 1.03e-151 | NS |
| DUSP2 | 1.09e-187 | -3.176 | 0.186 | 0.572 | 2.70e-183 | NS |
| RGCC | 9.40e-122 | -3.132 | 0.117 | 0.396 | 2.32e-117 | NS |
| XIST | 1.46e-129 | -3.092 | 0.024 | 0.219 | 3.61e-125 | NS |
| NFKBIZ | 5.23e-178 | -3.074 | 0.13 | 0.484 | 1.29e-173 | NS |
| CXCL1 | 1.92e-34 | -3.04 | 0.026 | 0.108 | 4.73e-30 | NS |
| ZNF503 | 5.85e-71 | -3.033 | 0.016 | 0.131 | 1.44e-66 | NS |
| CXCL2 | 1.92e-149 | -3.026 | 0.139 | 0.47 | 4.74e-145 | NS |
| ATF3 | 1.28e-197 | -3.018 | 0.159 | 0.554 | 3.16e-193 | NS |
| NFKBIA | 1.82e-210 | -2.985 | 0.445 | 0.814 | 4.49e-206 | NS |
| CST3 | 3.19e-255 | -2.963 | 0.863 | 0.977 | 7.88e-251 | NS |
| INSIG1 | 3.36e-177 | -2.957 | 0.223 | 0.608 | 8.29e-173 | NS |
| PPIF | 1.67e-116 | -2.897 | 0.063 | 0.3 | 4.13e-112 | NS |
| FAAP20 | 9.11e-113 | -2.881 | 0.029 | 0.215 | 2.25e-108 | NS |
| BCL2A1 | 2.37e-134 | -2.864 | 0.138 | 0.447 | 5.84e-130 | NS |
| FOSL1 | 1.78e-50 | -2.861 | 0.014 | 0.1 | 4.40e-46 | NS |
| MSRA | 8.91e-69 | -2.803 | 0.017 | 0.132 | 2.20e-64 | NS |
| NFKBID | 2.33e-199 | -2.76 | 0.109 | 0.486 | 5.75e-195 | NS |
| CCL4 | 1.72e-146 | -2.751 | 0.065 | 0.343 | 4.24e-142 | NS |
| KLF2 | 2.30e-58 | -2.751 | 0.027 | 0.145 | 5.67e-54 | NS |
| ZNF511 | 3.13e-48 | -2.674 | 0.015 | 0.101 | 7.74e-44 | NS |
| CDC42EP3 | 1.84e-131 | -2.662 | 0.067 | 0.328 | 4.53e-127 | NS |
| MZT2B | 1.27e-88 | -2.636 | 0.03 | 0.192 | 3.13e-84 | NS |
| RAB20 | 1.41e-45 | -2.609 | 0.018 | 0.105 | 3.47e-41 | NS |
| KLF3 | 1.07e-82 | -2.584 | 0.037 | 0.199 | 2.64e-78 | NS |
| CEBPD | 2.95e-98 | -2.568 | 0.065 | 0.281 | 7.28e-94 | NS |
| ARHGAP6 | 1.71e-68 | -2.559 | 0.032 | 0.171 | 4.23e-64 | NS |
| AP1S2 | 1.16e-171 | -2.537 | 0.123 | 0.48 | 2.86e-167 | NS |
| TLR2 | 3.81e-92 | -2.529 | 0.038 | 0.217 | 9.42e-88 | NS |
| RNF130 | 3.70e-191 | -2.465 | 0.13 | 0.517 | 9.14e-187 | NS |
| PLBD1 | 1.01e-104 | -2.461 | 0.055 | 0.27 | 2.48e-100 | NS |
| TNFAIP3 | 6.08e-149 | -2.457 | 0.223 | 0.575 | 1.50e-144 | NS |
| MAP1LC3B | 4.04e-151 | -2.451 | 0.092 | 0.405 | 9.96e-147 | NS |
| ARHGAP24 | 5.61e-63 | -2.446 | 0.029 | 0.156 | 1.38e-58 | NS |
| CD55 | 4.96e-121 | -2.423 | 0.132 | 0.426 | 1.23e-116 | NS |
| ARL5B | 2.57e-84 | -2.405 | 0.065 | 0.262 | 6.34e-80 | NS |
| ITM2B | 2.17e-213 | -2.404 | 0.378 | 0.763 | 5.35e-209 | NS |
| HEBP1 | 6.18e-65 | -2.404 | 0.03 | 0.161 | 1.53e-60 | NS |
| CSRNP1 | 6.89e-139 | -2.403 | 0.104 | 0.407 | 1.70e-134 | NS |
| LYVE1 | 3.70e-40 | -2.394 | 0.031 | 0.128 | 9.13e-36 | NS |
| HDAC9 | 2.39e-90 | -2.38 | 0.051 | 0.242 | 5.89e-86 | NS |
| PAPSS2 | 1.63e-129 | -2.362 | 0.102 | 0.398 | 4.01e-125 | NS |
| TSPYL2 | 1.66e-39 | -2.354 | 0.035 | 0.135 | 4.10e-35 | NS |
| ICAM1 | 1.29e-136 | -2.344 | 0.204 | 0.537 | 3.18e-132 | NS |
| PDGFB | 9.34e-56 | -2.344 | 0.037 | 0.164 | 2.31e-51 | NS |
| AKR7A2 | 3.11e-47 | -2.335 | 0.025 | 0.125 | 7.68e-43 | NS |
| CXCL8 | 4.29e-141 | -2.329 | 0.169 | 0.514 | 1.06e-136 | NS |
| EIF4A3 | 1.70e-87 | -2.321 | 0.08 | 0.296 | 4.20e-83 | NS |
| IER3 | 1.17e-151 | -2.318 | 0.288 | 0.643 | 2.88e-147 | NS |
| PRELID1 | 1.37e-126 | -2.318 | 0.075 | 0.343 | 3.38e-122 | NS |
| STX11 | 3.78e-108 | -2.294 | 0.095 | 0.351 | 9.34e-104 | NS |
| VSIG4 | 6.01e-140 | -2.292 | 0.093 | 0.395 | 1.48e-135 | NS |
| NME4 | 3.78e-54 | -2.29 | 0.034 | 0.155 | 9.33e-50 | NS |
| VPS37B | 8.88e-79 | -2.281 | 0.052 | 0.23 | 2.19e-74 | NS |
| CCL3 | 4.32e-76 | -2.274 | 0.062 | 0.246 | 1.07e-71 | NS |
| CDC42EP2 | 2.88e-33 | -2.245 | 0.028 | 0.111 | 7.11e-29 | NS |
| MAP2K3 | 8.78e-94 | -2.244 | 0.072 | 0.291 | 2.17e-89 | NS |
| CPED1 | 3.33e-46 | -2.237 | 0.028 | 0.13 | 8.23e-42 | NS |
| CEBPB | 4.57e-71 | -2.223 | 0.053 | 0.22 | 1.13e-66 | NS |
| PER3 | 7.67e-38 | -2.218 | 0.026 | 0.115 | 1.89e-33 | NS |
| CCRL2 | 6.26e-34 | -2.217 | 0.027 | 0.11 | 1.54e-29 | NS |
| MZT2A | 1.74e-43 | -2.208 | 0.028 | 0.126 | 4.30e-39 | NS |
| MMP9 | 2.43e-106 | -2.207 | 0.126 | 0.398 | 6.00e-102 | NS |
| GRASP | 2.80e-56 | -2.206 | 0.034 | 0.159 | 6.92e-52 | NS |
| MCUR1 | 1.14e-71 | -2.201 | 0.049 | 0.214 | 2.82e-67 | NS |
| ADAM28 | 1.46e-47 | -2.195 | 0.033 | 0.145 | 3.61e-43 | NS |
| KLF4 | 1.07e-113 | -2.191 | 0.158 | 0.455 | 2.63e-109 | NS |
| G0S2 | 1.25e-45 | -2.19 | 0.11 | 0.268 | 3.09e-41 | NS |
| ZC3H12A | 7.21e-107 | -2.185 | 0.115 | 0.381 | 1.78e-102 | NS |
| SURF1 | 8.06e-55 | -2.179 | 0.033 | 0.156 | 1.99e-50 | NS |
| CRTAP | 6.21e-142 | -2.177 | 0.106 | 0.421 | 1.53e-137 | NS |
| SNHG7 | 8.54e-49 | -2.176 | 0.03 | 0.14 | 2.11e-44 | NS |
| TOB2 | 4.70e-101 | -2.171 | 0.072 | 0.304 | 1.16e-96 | NS |
| CMTM7 | 5.63e-69 | -2.163 | 0.044 | 0.2 | 1.39e-64 | NS |
| DEPTOR | 3.39e-51 | -2.155 | 0.034 | 0.152 | 8.36e-47 | NS |
| IL13RA1 | 1.27e-115 | -2.153 | 0.1 | 0.375 | 3.14e-111 | NS |
| PTGER3 | 5.08e-53 | -2.15 | 0.039 | 0.165 | 1.25e-48 | NS |
| UBE2D1 | 1.12e-53 | -2.149 | 0.038 | 0.163 | 2.76e-49 | NS |
| FILIP1L | 7.71e-66 | -2.148 | 0.088 | 0.274 | 1.90e-61 | NS |
| CYC1 | 9.24e-64 | -2.143 | 0.044 | 0.193 | 2.28e-59 | NS |
| PIM3 | 7.81e-78 | -2.131 | 0.077 | 0.276 | 1.93e-73 | NS |
| IGFBP7 | 4.50e-51 | -2.125 | 0.047 | 0.178 | 1.11e-46 | NS |
| KDM6B | 5.33e-109 | -2.121 | 0.143 | 0.427 | 1.32e-104 | NS |
| CALHM6 | 5.66e-73 | -2.114 | 0.045 | 0.208 | 1.40e-68 | NS |
| BCL3 | 3.46e-64 | -2.105 | 0.054 | 0.213 | 8.55e-60 | NS |
| EHD1 | 4.75e-31 | -2.099 | 0.032 | 0.116 | 1.17e-26 | NS |
| FTH1 | 1.77e-227 | -2.097 | 0.943 | 0.994 | 4.36e-223 | NS |
| STARD8 | 3.08e-34 | -2.091 | 0.03 | 0.117 | 7.61e-30 | NS |
| FOSB | 5.04e-168 | -2.09 | 0.343 | 0.715 | 1.24e-163 | NS |
| COTL1 | 1.71e-85 | -2.076 | 0.419 | 0.619 | 4.21e-81 | NS |
| NR4A3 | 2.08e-107 | -2.073 | 0.152 | 0.439 | 5.14e-103 | NS |
| TAGAP | 1.06e-87 | -2.07 | 0.123 | 0.369 | 2.62e-83 | NS |
| TIPARP | 3.70e-85 | -2.066 | 0.09 | 0.31 | 9.13e-81 | NS |
| METTL21A | 3.29e-34 | -2.065 | 0.045 | 0.145 | 8.12e-30 | NS |
| RCSD1 | 1.02e-76 | -2.064 | 0.07 | 0.264 | 2.51e-72 | NS |
| BCL7B | 2.02e-34 | -2.063 | 0.028 | 0.113 | 4.98e-30 | NS |
| SELENOP | 6.47e-77 | -2.058 | 0.217 | 0.459 | 1.60e-72 | NS |
| GAS7 | 5.01e-61 | -2.056 | 0.058 | 0.215 | 1.24e-56 | NS |
| PTGS2 | 1.87e-68 | -2.054 | 0.08 | 0.268 | 4.63e-64 | NS |
| PFKFB3 | 1.01e-55 | -2.052 | 0.074 | 0.234 | 2.48e-51 | NS |
| CD302 | 1.18e-140 | -2.049 | 0.158 | 0.503 | 2.92e-136 | NS |
| ANKRD37 | 1.78e-56 | -2.048 | 0.067 | 0.225 | 4.40e-52 | NS |
| FCGR2B | 5.34e-137 | -2.026 | 0.226 | 0.571 | 1.32e-132 | NS |
| GAS6 | 4.88e-90 | -2.019 | 0.103 | 0.341 | 1.20e-85 | NS |
| ZFP36L2 | 2.00e-126 | -2.016 | 0.209 | 0.544 | 4.93e-122 | NS |
| CXCL12 | 9.68e-45 | -2.014 | 0.023 | 0.117 | 2.39e-40 | NS |
| MPST | 5.86e-38 | -2.014 | 0.03 | 0.123 | 1.45e-33 | NS |
| HLA-DRB5 | 9.44e-301 | -2.001 | 0.597 | 0.963 | 2.33e-296 | NS |
| MAP3K8 | 4.47e-88 | -1.997 | 0.107 | 0.345 | 1.10e-83 | NS |
| VEGFA | 1.25e-59 | -1.993 | 0.069 | 0.233 | 3.09e-55 | NS |
| RBM17 | 6.15e-74 | -1.988 | 0.062 | 0.244 | 1.52e-69 | NS |
| SH3BP5 | 1.50e-80 | -1.982 | 0.078 | 0.284 | 3.71e-76 | NS |
| TBC1D7 | 2.26e-33 | -1.974 | 0.029 | 0.115 | 5.58e-29 | NS |
| VPS4A | 9.43e-35 | -1.972 | 0.027 | 0.112 | 2.33e-30 | NS |
| RASGEF1B | 1.92e-98 | -1.966 | 0.116 | 0.376 | 4.74e-94 | NS |
| XBP1 | 4.34e-88 | -1.964 | 0.104 | 0.341 | 1.07e-83 | NS |
| NLRP1 | 1.57e-36 | -1.956 | 0.038 | 0.136 | 3.88e-32 | NS |
| BAG1 | 5.51e-53 | -1.954 | 0.048 | 0.185 | 1.36e-48 | NS |
| COX5A | 2.17e-103 | -1.95 | 0.125 | 0.398 | 5.35e-99 | NS |
| PARVB | 4.66e-61 | -1.936 | 0.055 | 0.21 | 1.15e-56 | NS |
| MCUB | 2.03e-56 | -1.933 | 0.048 | 0.191 | 5.01e-52 | NS |
| TOR3A | 9.61e-48 | -1.933 | 0.053 | 0.184 | 2.37e-43 | NS |
| GPR132 | 1.50e-42 | -1.933 | 0.036 | 0.143 | 3.70e-38 | NS |
| PPP1R10 | 5.77e-84 | -1.924 | 0.158 | 0.407 | 1.42e-79 | NS |
| PER1 | 4.59e-48 | -1.922 | 0.052 | 0.184 | 1.13e-43 | NS |
| NR4A1 | 2.05e-154 | -1.919 | 0.334 | 0.698 | 5.06e-150 | NS |
| GNPTG | 2.73e-52 | -1.919 | 0.049 | 0.186 | 6.74e-48 | NS |
| NCF1 | 3.86e-52 | -1.917 | 0.062 | 0.209 | 9.53e-48 | NS |
| NSUN6 | 1.27e-35 | -1.912 | 0.045 | 0.148 | 3.14e-31 | NS |
| ME1 | 2.27e-48 | -1.908 | 0.047 | 0.174 | 5.61e-44 | NS |
| RALGAPA1 | 5.02e-34 | -1.906 | 0.041 | 0.138 | 1.24e-29 | NS |
| C5AR1 | 1.32e-59 | -1.899 | 0.125 | 0.32 | 3.26e-55 | NS |
| AFF3 | 2.84e-42 | -1.894 | 0.045 | 0.161 | 7.01e-38 | NS |
| CCDC170 | 9.94e-25 | -1.892 | 0.032 | 0.104 | 2.45e-20 | NS |
| GYPC | 1.91e-60 | -1.884 | 0.082 | 0.258 | 4.71e-56 | NS |
| NR4A2 | 7.82e-148 | -1.878 | 0.346 | 0.7 | 1.93e-143 | NS |
| CD83 | 3.23e-144 | -1.871 | 0.4 | 0.729 | 7.98e-140 | NS |
| EPN1 | 3.05e-49 | -1.871 | 0.047 | 0.177 | 7.54e-45 | NS |
| RILPL2 | 1.65e-62 | -1.868 | 0.083 | 0.264 | 4.07e-58 | NS |
| YPEL2 | 4.44e-32 | -1.868 | 0.032 | 0.119 | 1.09e-27 | NS |
| PLD4 | 2.31e-39 | -1.86 | 0.048 | 0.161 | 5.71e-35 | NS |
| PGLS | 4.70e-93 | -1.853 | 0.121 | 0.376 | 1.16e-88 | NS |
| CD163 | 6.83e-94 | -1.845 | 0.127 | 0.393 | 1.69e-89 | NS |
| GRB2 | 1.37e-119 | -1.842 | 0.197 | 0.518 | 3.37e-115 | NS |
| RNASET2 | 3.66e-98 | -1.842 | 0.173 | 0.455 | 9.03e-94 | NS |
| CKS2 | 1.23e-80 | -1.84 | 0.191 | 0.445 | 3.03e-76 | NS |
| RELB | 1.17e-31 | -1.827 | 0.031 | 0.115 | 2.90e-27 | NS |
| ETS2 | 1.12e-59 | -1.826 | 0.094 | 0.275 | 2.76e-55 | NS |
| PRMT9 | 2.39e-32 | -1.823 | 0.043 | 0.139 | 5.90e-28 | NS |
| PSIP1 | 7.64e-31 | -1.823 | 0.033 | 0.119 | 1.89e-26 | NS |
| CD14 | 2.70e-92 | -1.821 | 0.182 | 0.464 | 6.67e-88 | NS |
| PXDC1 | 6.73e-27 | -1.815 | 0.043 | 0.128 | 1.66e-22 | NS |
| NT5C | 4.91e-46 | -1.814 | 0.048 | 0.173 | 1.21e-41 | NS |
| ABHD12 | 1.38e-48 | -1.811 | 0.054 | 0.19 | 3.40e-44 | NS |
| MSR1 | 2.87e-79 | -1.809 | 0.067 | 0.265 | 7.08e-75 | NS |
| B4GALT1 | 6.57e-65 | -1.805 | 0.086 | 0.274 | 1.62e-60 | NS |
| AVPI1 | 3.36e-60 | -1.801 | 0.101 | 0.288 | 8.28e-56 | NS |
| LYN | 4.79e-85 | -1.788 | 0.131 | 0.379 | 1.18e-80 | NS |
| TSPAN3 | 3.87e-31 | -1.788 | 0.039 | 0.129 | 9.54e-27 | NS |
| PLAUR | 2.23e-78 | -1.785 | 0.489 | 0.676 | 5.51e-74 | NS |
| SQSTM1 | 4.54e-115 | -1.784 | 0.267 | 0.594 | 1.12e-110 | NS |
| METTL9 | 1.87e-44 | -1.774 | 0.049 | 0.173 | 4.61e-40 | NS |
| PDE4B | 2.44e-62 | -1.77 | 0.113 | 0.309 | 6.03e-58 | NS |
| FOXO3 | 2.93e-30 | -1.768 | 0.049 | 0.146 | 7.22e-26 | NS |
| ATP1B1 | 8.29e-88 | -1.766 | 0.137 | 0.393 | 2.05e-83 | NS |
| SLC40A1 | 5.25e-48 | -1.763 | 0.114 | 0.283 | 1.29e-43 | NS |
| JOSD1 | 4.50e-37 | -1.757 | 0.048 | 0.157 | 1.11e-32 | NS |
| AC007384.1 | 9.66e-62 | -1.756 | 0.087 | 0.269 | 2.38e-57 | NS |
| TRAPPC5 | 1.78e-77 | -1.754 | 0.102 | 0.323 | 4.39e-73 | NS |
| HERPUD1 | 1.76e-69 | -1.747 | 0.283 | 0.521 | 4.34e-65 | NS |
| RBBP8 | 2.03e-28 | -1.747 | 0.055 | 0.153 | 5.02e-24 | NS |
| EPS8 | 4.93e-50 | -1.746 | 0.067 | 0.216 | 1.22e-45 | NS |
| RTN1 | 4.32e-42 | -1.743 | 0.058 | 0.184 | 1.07e-37 | NS |
| REL | 1.16e-133 | -1.736 | 0.287 | 0.641 | 2.86e-129 | NS |
| NBPF14 | 1.66e-31 | -1.734 | 0.039 | 0.13 | 4.11e-27 | NS |
| MMP19 | 1.15e-19 | -1.729 | 0.092 | 0.183 | 2.83e-15 | NS |
| POU2F2 | 8.39e-48 | -1.726 | 0.067 | 0.21 | 2.07e-43 | NS |
| GNB2 | 4.31e-113 | -1.717 | 0.168 | 0.482 | 1.06e-108 | NS |
| ACAP2 | 1.99e-81 | -1.716 | 0.13 | 0.374 | 4.91e-77 | NS |
| HPCAL1 | 2.57e-28 | -1.714 | 0.037 | 0.121 | 6.35e-24 | NS |
| PTMS | 4.70e-77 | -1.708 | 0.155 | 0.399 | 1.16e-72 | NS |
| NCF4 | 2.26e-75 | -1.706 | 0.134 | 0.367 | 5.57e-71 | NS |
| CMTM3 | 1.38e-64 | -1.706 | 0.088 | 0.278 | 3.40e-60 | NS |
| AGPAT2 | 2.18e-36 | -1.703 | 0.047 | 0.154 | 5.39e-32 | NS |
| TNS1 | 5.75e-20 | -1.699 | 0.036 | 0.103 | 1.42e-15 | NS |
| ZFAND3 | 1.48e-22 | -1.697 | 0.032 | 0.101 | 3.66e-18 | NS |
| ANP32B | 2.15e-70 | -1.696 | 0.123 | 0.343 | 5.31e-66 | NS |
| NFE2L2 | 1.80e-94 | -1.694 | 0.243 | 0.531 | 4.45e-90 | NS |
| JMJD6 | 1.36e-41 | -1.694 | 0.067 | 0.198 | 3.36e-37 | NS |
| GADD45GIP1 | 1.50e-42 | -1.684 | 0.055 | 0.18 | 3.70e-38 | NS |
| GPR34 | 9.20e-23 | -1.684 | 0.045 | 0.125 | 2.27e-18 | NS |
| WWP1 | 3.02e-30 | -1.683 | 0.059 | 0.163 | 7.45e-26 | NS |
| ZNF331 | 5.41e-85 | -1.681 | 0.159 | 0.423 | 1.33e-80 | NS |
| RAB32 | 9.21e-44 | -1.678 | 0.069 | 0.206 | 2.27e-39 | NS |
| PITHD1 | 5.82e-27 | -1.674 | 0.034 | 0.113 | 1.44e-22 | NS |
| SPAG9 | 2.90e-59 | -1.669 | 0.109 | 0.301 | 7.16e-55 | NS |
| A2M | 1.30e-36 | -1.668 | 0.067 | 0.189 | 3.21e-32 | NS |
| RPS2 | 1.87e-167 | -1.665 | 0.688 | 0.875 | 4.62e-163 | NS |
| FCER1A | 1.28e-64 | -1.658 | 0.4 | 0.597 | 3.15e-60 | NS |
| BTG3 | 1.14e-21 | -1.654 | 0.036 | 0.107 | 2.82e-17 | NS |
| TWISTNB | 3.13e-10 | -1.652 | 0.095 | 0.158 | 7.73e-06 | NS |
| ITSN1 | 7.35e-18 | -1.651 | 0.044 | 0.111 | 1.81e-13 | NS |
| CNRIP1 | 5.41e-26 | -1.65 | 0.033 | 0.11 | 1.34e-21 | NS |
| MAFF | 3.66e-39 | -1.648 | 0.086 | 0.224 | 9.04e-35 | NS |
| RAB4A | 1.01e-39 | -1.647 | 0.056 | 0.178 | 2.49e-35 | NS |
| GPR183 | 3.56e-81 | -1.644 | 0.425 | 0.65 | 8.79e-77 | NS |
| TRAF1 | 3.32e-39 | -1.638 | 0.111 | 0.261 | 8.19e-35 | NS |
| FAM174C | 1.33e-29 | -1.637 | 0.043 | 0.134 | 3.27e-25 | NS |
| MEF2C | 2.79e-101 | -1.635 | 0.176 | 0.478 | 6.90e-97 | NS |
| TNF | 1.00e-31 | -1.634 | 0.095 | 0.222 | 2.48e-27 | NS |
| CAMK1 | 7.87e-25 | -1.63 | 0.032 | 0.105 | 1.94e-20 | NS |
| SLFN11 | 1.14e-22 | -1.629 | 0.058 | 0.145 | 2.81e-18 | NS |
| MEPCE | 2.05e-21 | -1.627 | 0.033 | 0.1 | 5.07e-17 | NS |
| SERP1 | 1.60e-89 | -1.616 | 0.206 | 0.488 | 3.95e-85 | NS |
| PIK3R1 | 4.50e-35 | -1.616 | 0.055 | 0.166 | 1.11e-30 | NS |
| OAS1 | 1.69e-23 | -1.616 | 0.033 | 0.104 | 4.18e-19 | NS |
| FAM50A | 1.01e-40 | -1.608 | 0.063 | 0.192 | 2.49e-36 | NS |
| MMP24OS | 3.27e-23 | -1.607 | 0.038 | 0.113 | 8.06e-19 | NS |
| GFRA2 | 1.03e-54 | -1.606 | 0.097 | 0.273 | 2.53e-50 | NS |
| TNFRSF1B | 1.85e-47 | -1.605 | 0.113 | 0.283 | 4.56e-43 | NS |
| PDCD2 | 5.69e-24 | -1.602 | 0.032 | 0.103 | 1.41e-19 | NS |
| SMCHD1 | 3.74e-43 | -1.6 | 0.071 | 0.209 | 9.22e-39 | NS |
| IFNGR2 | 3.57e-37 | -1.6 | 0.064 | 0.187 | 8.80e-33 | NS |
| AOAH | 1.17e-32 | -1.574 | 0.05 | 0.154 | 2.89e-28 | NS |
| NFKBIE | 3.10e-30 | -1.574 | 0.053 | 0.154 | 7.66e-26 | NS |
| GEM | 7.10e-29 | -1.566 | 0.057 | 0.158 | 1.75e-24 | NS |
| PIM2 | 8.07e-24 | -1.566 | 0.042 | 0.122 | 1.99e-19 | NS |
| IL1R2 | 1.56e-52 | -1.563 | 0.301 | 0.501 | 3.84e-48 | NS |
| PFN1 | 1.55e-162 | -1.561 | 0.662 | 0.858 | 3.82e-158 | NS |
| PNPLA2 | 4.17e-29 | -1.55 | 0.051 | 0.148 | 1.03e-24 | NS |
| SNX18 | 8.94e-21 | -1.55 | 0.037 | 0.106 | 2.21e-16 | NS |
| TPRA1 | 3.71e-24 | -1.546 | 0.045 | 0.127 | 9.15e-20 | NS |
| PIK3AP1 | 1.01e-22 | -1.546 | 0.037 | 0.111 | 2.49e-18 | NS |
| RIPK2 | 4.88e-38 | -1.544 | 0.071 | 0.2 | 1.20e-33 | NS |
| CRYL1 | 6.49e-32 | -1.543 | 0.059 | 0.167 | 1.60e-27 | NS |
| FCN1 | 6.66e-34 | -1.536 | 0.088 | 0.218 | 1.64e-29 | NS |
| UBXN11 | 3.90e-29 | -1.536 | 0.044 | 0.135 | 9.63e-25 | NS |
| IL17RA | 4.89e-28 | -1.535 | 0.053 | 0.149 | 1.21e-23 | NS |
| HSF1 | 7.61e-22 | -1.535 | 0.033 | 0.102 | 1.88e-17 | NS |
| FLT3 | 2.50e-19 | -1.535 | 0.039 | 0.107 | 6.16e-15 | NS |
| CCDC50 | 1.11e-33 | -1.534 | 0.066 | 0.183 | 2.73e-29 | NS |
| RAC1 | 5.09e-122 | -1.531 | 0.373 | 0.679 | 1.26e-117 | NS |
| AC004687.1 | 6.37e-24 | -1.527 | 0.049 | 0.133 | 1.57e-19 | NS |
| UROS | 1.26e-23 | -1.524 | 0.041 | 0.12 | 3.11e-19 | NS |
| H1F0 | 2.29e-25 | -1.521 | 0.074 | 0.175 | 5.65e-21 | NS |
| SLBP | 1.93e-22 | -1.521 | 0.042 | 0.119 | 4.78e-18 | NS |
| ARRDC2 | 3.67e-25 | -1.52 | 0.043 | 0.126 | 9.06e-21 | NS |
| ZBTB1 | 1.04e-22 | -1.518 | 0.047 | 0.127 | 2.57e-18 | NS |
| EGR1 | 1.09e-19 | -1.508 | 0.09 | 0.183 | 2.70e-15 | NS |
| ACAA1 | 5.73e-38 | -1.504 | 0.064 | 0.189 | 1.41e-33 | NS |
| MTMR14 | 2.63e-32 | -1.502 | 0.07 | 0.186 | 6.49e-28 | NS |
| CCND2 | 1.16e-38 | -1.499 | 0.098 | 0.243 | 2.85e-34 | NS |
| RC3H1 | 2.24e-22 | -1.499 | 0.044 | 0.122 | 5.52e-18 | NS |
| JUNB | 2.29e-109 | -1.497 | 0.594 | 0.815 | 5.66e-105 | NS |
| NINJ1 | 8.88e-67 | -1.497 | 0.206 | 0.446 | 2.19e-62 | NS |
| PHACTR1 | 9.06e-50 | -1.497 | 0.138 | 0.323 | 2.24e-45 | NS |
| SELENOK | 1.75e-71 | -1.496 | 0.361 | 0.599 | 4.31e-67 | NS |
| MTCH1 | 4.10e-47 | -1.496 | 0.087 | 0.245 | 1.01e-42 | NS |
| PLEK | 1.09e-91 | -1.492 | 0.463 | 0.718 | 2.68e-87 | NS |
| IGFBP5 | 5.29e-22 | -1.488 | 0.038 | 0.111 | 1.31e-17 | NS |
| CREM | 5.56e-52 | -1.485 | 0.2 | 0.403 | 1.37e-47 | NS |
| NAPRT | 6.68e-25 | -1.483 | 0.045 | 0.129 | 1.65e-20 | NS |
| H2AFV | 5.71e-45 | -1.48 | 0.109 | 0.274 | 1.41e-40 | NS |
| PRKCD | 9.39e-32 | -1.475 | 0.061 | 0.17 | 2.32e-27 | NS |
| RAB5IF | 2.06e-22 | -1.475 | 0.045 | 0.124 | 5.08e-18 | NS |
| DIPK2A | 4.97e-31 | -1.472 | 0.081 | 0.199 | 1.23e-26 | NS |
| RNF24 | 7.78e-23 | -1.472 | 0.046 | 0.126 | 1.92e-18 | NS |
| SNX9 | 2.13e-29 | -1.469 | 0.075 | 0.188 | 5.26e-25 | NS |
| FRMD4B | 2.72e-65 | -1.468 | 0.173 | 0.405 | 6.72e-61 | NS |
| AAK1 | 4.14e-22 | -1.468 | 0.04 | 0.115 | 1.02e-17 | NS |
| CREG1 | 3.07e-40 | -1.462 | 0.096 | 0.245 | 7.57e-36 | NS |
| ARL5A | 4.35e-34 | -1.462 | 0.063 | 0.179 | 1.07e-29 | NS |
| RNF166 | 6.40e-22 | -1.462 | 0.038 | 0.111 | 1.58e-17 | NS |
| SOD2 | 4.23e-49 | -1.456 | 0.247 | 0.449 | 1.04e-44 | NS |
| REEP3 | 7.18e-20 | -1.452 | 0.037 | 0.105 | 1.77e-15 | NS |
| GADD45B | 4.34e-115 | -1.449 | 0.501 | 0.774 | 1.07e-110 | NS |
| GPCPD1 | 3.57e-22 | -1.445 | 0.054 | 0.138 | 8.81e-18 | NS |
| AKIRIN1 | 1.68e-24 | -1.444 | 0.05 | 0.136 | 4.15e-20 | NS |
| DDIT3 | 8.07e-22 | -1.444 | 0.055 | 0.138 | 1.99e-17 | NS |
| DNAJA1 | 2.28e-90 | -1.44 | 0.371 | 0.644 | 5.62e-86 | NS |
| RASSF5 | 1.33e-36 | -1.437 | 0.078 | 0.209 | 3.28e-32 | NS |
| TNFRSF10B | 3.69e-23 | -1.437 | 0.051 | 0.135 | 9.12e-19 | NS |
| ARHGAP4 | 6.44e-42 | -1.433 | 0.081 | 0.226 | 1.59e-37 | NS |
| STUB1 | 1.86e-26 | -1.426 | 0.048 | 0.137 | 4.59e-22 | NS |
| HMG20B | 9.58e-25 | -1.425 | 0.05 | 0.138 | 2.36e-20 | NS |
| FOLR2 | 4.04e-63 | -1.424 | 0.178 | 0.415 | 9.96e-59 | NS |
| ARL4A | 5.17e-26 | -1.424 | 0.073 | 0.177 | 1.28e-21 | NS |
| SERTAD1 | 5.31e-46 | -1.419 | 0.163 | 0.348 | 1.31e-41 | NS |
| NRROS | 2.44e-45 | -1.416 | 0.107 | 0.272 | 6.01e-41 | NS |
| ST6GAL1 | 4.05e-19 | -1.414 | 0.043 | 0.114 | 9.99e-15 | NS |
| STAB1 | 7.11e-40 | -1.413 | 0.16 | 0.329 | 1.76e-35 | NS |
| LRP1 | 3.96e-24 | -1.413 | 0.073 | 0.172 | 9.76e-20 | NS |
| ITSN2 | 2.99e-49 | -1.412 | 0.141 | 0.329 | 7.38e-45 | NS |
| IRF1 | 4.15e-39 | -1.412 | 0.118 | 0.273 | 1.03e-34 | NS |
| RANBP1 | 3.06e-29 | -1.409 | 0.07 | 0.179 | 7.54e-25 | NS |
| BTG2 | 6.49e-73 | -1.408 | 0.301 | 0.568 | 1.60e-68 | NS |
| IL10RA | 1.01e-75 | -1.403 | 0.203 | 0.468 | 2.49e-71 | NS |
| AC020916.1 | 1.26e-46 | -1.402 | 0.149 | 0.334 | 3.11e-42 | NS |
| JAK1 | 3.20e-64 | -1.4 | 0.178 | 0.41 | 7.89e-60 | NS |
| FAM53C | 3.28e-23 | -1.4 | 0.061 | 0.152 | 8.09e-19 | NS |
| GPR155 | 4.39e-19 | -1.399 | 0.04 | 0.108 | 1.08e-14 | NS |
| NBPF26 | 8.01e-36 | -1.39 | 0.088 | 0.223 | 1.98e-31 | NS |
| BIN1 | 2.16e-18 | -1.388 | 0.038 | 0.104 | 5.32e-14 | NS |
| CYBB | 5.70e-44 | -1.385 | 0.151 | 0.33 | 1.41e-39 | NS |
| GK | 2.20e-38 | -1.38 | 0.143 | 0.304 | 5.42e-34 | NS |
| DPEP2 | 2.37e-17 | -1.38 | 0.039 | 0.102 | 5.84e-13 | NS |
| GIMAP4 | 1.80e-30 | -1.377 | 0.063 | 0.172 | 4.45e-26 | NS |
| FOSL2 | 1.66e-41 | -1.373 | 0.106 | 0.263 | 4.10e-37 | NS |
| PPP1R15B | 1.50e-30 | -1.369 | 0.09 | 0.213 | 3.69e-26 | NS |
| WAC-AS1 | 5.02e-22 | -1.367 | 0.054 | 0.138 | 1.24e-17 | NS |
| SLC7A7 | 4.23e-24 | -1.36 | 0.056 | 0.146 | 1.04e-19 | NS |
| ETV3 | 3.27e-48 | -1.358 | 0.153 | 0.342 | 8.07e-44 | NS |
| PTGER4 | 6.15e-27 | -1.358 | 0.086 | 0.199 | 1.52e-22 | NS |
| KIAA1109 | 5.85e-18 | -1.358 | 0.048 | 0.118 | 1.44e-13 | NS |
| LAIR1 | 1.41e-25 | -1.353 | 0.064 | 0.161 | 3.48e-21 | NS |
| GATM | 1.58e-24 | -1.353 | 0.075 | 0.176 | 3.90e-20 | NS |
| DDX3X | 5.95e-53 | -1.349 | 0.243 | 0.459 | 1.47e-48 | NS |
| NEK7 | 2.68e-17 | -1.349 | 0.04 | 0.105 | 6.63e-13 | NS |
| UBN1 | 6.88e-17 | -1.346 | 0.04 | 0.103 | 1.70e-12 | NS |
| SYAP1 | 2.01e-35 | -1.345 | 0.143 | 0.299 | 4.95e-31 | NS |
| SLC43A2 | 5.72e-28 | -1.339 | 0.07 | 0.177 | 1.41e-23 | NS |
| ADAP2 | 4.02e-24 | -1.337 | 0.083 | 0.186 | 9.93e-20 | NS |
| RNF145 | 7.09e-21 | -1.336 | 0.06 | 0.144 | 1.75e-16 | NS |
| LAGE3 | 2.01e-19 | -1.331 | 0.058 | 0.138 | 4.96e-15 | NS |
| IGF1 | 2.92e-19 | -1.33 | 0.053 | 0.129 | 7.20e-15 | NS |
| CHD1 | 2.04e-41 | -1.327 | 0.146 | 0.317 | 5.05e-37 | NS |
| RCOR1 | 1.78e-23 | -1.325 | 0.055 | 0.143 | 4.40e-19 | NS |
| TBC1D14 | 1.90e-20 | -1.324 | 0.055 | 0.135 | 4.70e-16 | NS |
| NAAA | 8.49e-35 | -1.32 | 0.131 | 0.283 | 2.10e-30 | NS |
| ATP2B1-AS1 | 8.17e-22 | -1.318 | 0.056 | 0.14 | 2.02e-17 | NS |
| NIPSNAP2 | 2.67e-19 | -1.317 | 0.051 | 0.126 | 6.59e-15 | NS |
| TSC22D2 | 4.00e-17 | -1.317 | 0.059 | 0.133 | 9.87e-13 | NS |
| TNFAIP2 | 2.81e-58 | -1.315 | 0.136 | 0.345 | 6.95e-54 | NS |
| SLC25A19 | 4.63e-23 | -1.314 | 0.074 | 0.171 | 1.14e-18 | NS |
| AC100810.1 | 8.01e-17 | -1.314 | 0.045 | 0.112 | 1.98e-12 | NS |
| CX3CR1 | 2.07e-23 | -1.302 | 0.065 | 0.159 | 5.10e-19 | NS |
| DPP7 | 1.16e-42 | -1.3 | 0.113 | 0.277 | 2.87e-38 | NS |
| IGFBP4 | 3.29e-15 | -1.299 | 0.045 | 0.108 | 8.11e-11 | NS |
| APAF1 | 3.64e-23 | -1.293 | 0.059 | 0.149 | 8.99e-19 | NS |
| SMIM4 | 4.99e-25 | -1.292 | 0.059 | 0.153 | 1.23e-20 | NS |
| NOTCH2NLA | 1.06e-16 | -1.291 | 0.039 | 0.102 | 2.61e-12 | NS |
| SOCS3 | 4.73e-40 | -1.29 | 0.154 | 0.326 | 1.17e-35 | NS |
| NCOR1 | 2.46e-34 | -1.29 | 0.103 | 0.242 | 6.06e-30 | NS |
| DNAJB4 | 1.49e-16 | -1.282 | 0.057 | 0.129 | 3.67e-12 | NS |
| FCHSD2 | 3.97e-22 | -1.281 | 0.067 | 0.159 | 9.81e-18 | NS |
| C5orf24 | 6.90e-16 | -1.281 | 0.048 | 0.114 | 1.70e-11 | NS |
| MCL1 | 4.29e-72 | -1.277 | 0.388 | 0.633 | 1.06e-67 | NS |
| DNAJC1 | 5.71e-16 | -1.273 | 0.04 | 0.101 | 1.41e-11 | NS |
| USP36 | 5.99e-19 | -1.268 | 0.066 | 0.149 | 1.48e-14 | NS |
| UQCRFS1 | 6.79e-27 | -1.266 | 0.074 | 0.182 | 1.68e-22 | NS |
| SERPING1 | 2.11e-24 | -1.266 | 0.069 | 0.166 | 5.20e-20 | NS |
| C1QBP | 6.39e-25 | -1.264 | 0.079 | 0.184 | 1.58e-20 | NS |
| CACUL1 | 5.23e-39 | -1.261 | 0.102 | 0.253 | 1.29e-34 | NS |
| EHD4 | 4.66e-27 | -1.261 | 0.077 | 0.186 | 1.15e-22 | NS |
| UVRAG | 6.85e-43 | -1.259 | 0.16 | 0.34 | 1.69e-38 | NS |
| ZRSR2 | 3.51e-15 | -1.259 | 0.043 | 0.104 | 8.66e-11 | NS |
| MCOLN1 | 1.43e-16 | -1.256 | 0.053 | 0.124 | 3.54e-12 | NS |
| WASF2 | 4.14e-66 | -1.255 | 0.219 | 0.475 | 1.02e-61 | NS |
| CCND3 | 1.29e-32 | -1.255 | 0.095 | 0.227 | 3.19e-28 | NS |
| FCGR2A | 1.32e-63 | -1.254 | 0.205 | 0.448 | 3.26e-59 | NS |
| GLUD1 | 3.86e-26 | -1.254 | 0.155 | 0.289 | 9.52e-22 | NS |
| HIST1H4C | 1.47e-19 | -1.251 | 0.133 | 0.244 | 3.63e-15 | NS |
| OXSR1 | 2.75e-19 | -1.251 | 0.067 | 0.152 | 6.79e-15 | NS |
| ARHGAP5 | 1.59e-16 | -1.251 | 0.073 | 0.152 | 3.92e-12 | NS |
| LRIF1 | 6.63e-21 | -1.242 | 0.088 | 0.185 | 1.64e-16 | NS |
| COLEC12 | 4.67e-11 | -1.24 | 0.057 | 0.113 | 1.15e-06 | NS |
| HMGA1 | 4.89e-25 | -1.237 | 0.117 | 0.235 | 1.21e-20 | NS |
| PMAIP1 | 6.73e-20 | -1.233 | 0.085 | 0.178 | 1.66e-15 | NS |
| EPB41L4A-AS1 | 1.46e-14 | -1.233 | 0.045 | 0.106 | 3.60e-10 | NS |
| SLC4A7 | 4.71e-22 | -1.231 | 0.077 | 0.173 | 1.16e-17 | NS |
| FLI1 | 8.85e-25 | -1.228 | 0.075 | 0.177 | 2.18e-20 | NS |
| PPM1G | 2.55e-25 | -1.225 | 0.094 | 0.207 | 6.30e-21 | NS |
| PLAU | 4.52e-14 | -1.225 | 0.088 | 0.164 | 1.12e-09 | NS |
| CHD2 | 1.68e-36 | -1.224 | 0.135 | 0.293 | 4.14e-32 | NS |
| KIF1B | 1.98e-14 | -1.223 | 0.051 | 0.115 | 4.88e-10 | NS |
| HERC2 | 8.83e-14 | -1.223 | 0.05 | 0.111 | 2.18e-09 | NS |
| TGFBR2 | 1.21e-27 | -1.222 | 0.088 | 0.204 | 2.99e-23 | NS |
| RELL1 | 2.24e-13 | -1.218 | 0.057 | 0.121 | 5.53e-09 | NS |
| SCARB2 | 2.70e-15 | -1.216 | 0.065 | 0.136 | 6.66e-11 | NS |
| MEF2D | 9.41e-16 | -1.215 | 0.042 | 0.104 | 2.32e-11 | NS |
| TLR4 | 2.65e-19 | -1.213 | 0.061 | 0.143 | 6.55e-15 | NS |
| PNRC2 | 3.19e-40 | -1.204 | 0.169 | 0.346 | 7.86e-36 | NS |
| GLA | 2.82e-35 | -1.2 | 0.202 | 0.374 | 6.95e-31 | NS |
| PSTPIP1 | 1.45e-26 | -1.2 | 0.094 | 0.21 | 3.57e-22 | NS |
| RIN3 | 7.97e-18 | -1.199 | 0.057 | 0.132 | 1.97e-13 | NS |
| CCDC93 | 2.00e-11 | -1.199 | 0.047 | 0.1 | 4.94e-07 | NS |
| MRC1 | 7.26e-69 | -1.198 | 0.377 | 0.633 | 1.79e-64 | NS |
| ZYX | 4.72e-60 | -1.195 | 0.263 | 0.505 | 1.17e-55 | NS |
| PABPC4 | 2.62e-38 | -1.195 | 0.135 | 0.299 | 6.46e-34 | NS |
| HMGN1 | 2.61e-39 | -1.193 | 0.15 | 0.32 | 6.45e-35 | NS |
| WIPI2 | 5.41e-17 | -1.193 | 0.059 | 0.133 | 1.33e-12 | NS |
| TMX4 | 8.80e-16 | -1.193 | 0.064 | 0.137 | 2.17e-11 | NS |
| MBP | 5.70e-31 | -1.192 | 0.138 | 0.281 | 1.41e-26 | NS |
| ZFAND5 | 1.25e-33 | -1.191 | 0.201 | 0.366 | 3.07e-29 | NS |
| TP53BP2 | 1.33e-12 | -1.191 | 0.045 | 0.1 | 3.28e-08 | NS |
| IRF8 | 8.38e-50 | -1.19 | 0.159 | 0.366 | 2.07e-45 | NS |
| SMARCB1 | 5.65e-17 | -1.19 | 0.049 | 0.119 | 1.39e-12 | NS |
| EIF4EBP3 | 1.37e-11 | -1.188 | 0.052 | 0.107 | 3.38e-07 | NS |
| FUCA1 | 7.48e-16 | -1.186 | 0.077 | 0.156 | 1.85e-11 | NS |
| TMEM123 | 2.88e-39 | -1.183 | 0.204 | 0.385 | 7.10e-35 | NS |
| COLGALT1 | 8.18e-14 | -1.183 | 0.058 | 0.124 | 2.02e-09 | NS |
| DAB2 | 4.84e-33 | -1.182 | 0.283 | 0.453 | 1.20e-28 | NS |
| FCGRT | 8.03e-87 | -1.181 | 0.412 | 0.718 | 1.98e-82 | NS |
| IL10 | 6.86e-07 | -1.18 | 0.068 | 0.113 | 1.69e-02 | NS |
| PPP1R15A | 7.63e-86 | -1.175 | 0.518 | 0.764 | 1.88e-81 | NS |
| LPCAT2 | 1.55e-31 | -1.171 | 0.112 | 0.25 | 3.83e-27 | NS |
| MARCO | 1.16e-18 | -1.171 | 0.092 | 0.186 | 2.87e-14 | NS |
| GUK1 | 5.10e-51 | -1.17 | 0.188 | 0.403 | 1.26e-46 | NS |
| MCTP1 | 7.65e-40 | -1.17 | 0.118 | 0.279 | 1.89e-35 | NS |
| KDELR2 | 1.95e-24 | -1.169 | 0.078 | 0.182 | 4.82e-20 | NS |
| FKBP4 | 1.98e-13 | -1.169 | 0.052 | 0.113 | 4.89e-09 | NS |
| OTULINL | 4.80e-34 | -1.168 | 0.156 | 0.314 | 1.19e-29 | NS |
| AFF4 | 1.90e-27 | -1.167 | 0.104 | 0.227 | 4.69e-23 | NS |
| KLF10 | 5.12e-35 | -1.161 | 0.167 | 0.331 | 1.26e-30 | NS |
| LILRB5 | 1.03e-20 | -1.159 | 0.105 | 0.208 | 2.54e-16 | NS |
| MIR155HG | 9.09e-18 | -1.157 | 0.053 | 0.126 | 2.24e-13 | NS |
| AKAP9 | 6.17e-29 | -1.156 | 0.159 | 0.303 | 1.52e-24 | NS |
| PANK2 | 1.07e-13 | -1.153 | 0.043 | 0.1 | 2.63e-09 | NS |
| TCEA3 | 1.53e-15 | -1.148 | 0.067 | 0.141 | 3.77e-11 | NS |
| SGMS1 | 5.98e-13 | -1.148 | 0.06 | 0.123 | 1.48e-08 | NS |
| PLCB2 | 5.08e-19 | -1.145 | 0.064 | 0.147 | 1.25e-14 | NS |
| VSIR | 3.60e-37 | -1.144 | 0.194 | 0.369 | 8.89e-33 | NS |
| CASC3 | 2.94e-17 | -1.143 | 0.066 | 0.145 | 7.25e-13 | NS |
| RNF115 | 3.07e-13 | -1.143 | 0.048 | 0.107 | 7.58e-09 | NS |
| GTF3A | 8.87e-23 | -1.142 | 0.086 | 0.189 | 2.19e-18 | NS |
| BRD2 | 1.76e-49 | -1.139 | 0.29 | 0.505 | 4.35e-45 | NS |
| CLTB | 1.60e-22 | -1.139 | 0.083 | 0.185 | 3.95e-18 | NS |
| NUMA1 | 5.90e-22 | -1.137 | 0.085 | 0.186 | 1.46e-17 | NS |
| MKNK2 | 2.26e-19 | -1.135 | 0.068 | 0.154 | 5.58e-15 | NS |
| ANKLE2 | 3.08e-12 | -1.132 | 0.046 | 0.101 | 7.59e-08 | NS |
| RBM47 | 8.45e-22 | -1.129 | 0.11 | 0.22 | 2.09e-17 | NS |
| TBC1D9 | 1.71e-14 | -1.129 | 0.058 | 0.125 | 4.21e-10 | NS |
| MS4A7 | 1.00e-44 | -1.128 | 0.264 | 0.478 | 2.48e-40 | NS |
| SLC66A3 | 2.41e-29 | -1.127 | 0.129 | 0.268 | 5.95e-25 | NS |
| SLC8A1 | 6.25e-23 | -1.127 | 0.088 | 0.193 | 1.54e-18 | NS |
| JAK2 | 9.28e-21 | -1.125 | 0.091 | 0.191 | 2.29e-16 | NS |
| OSBPL1A | 7.73e-14 | -1.123 | 0.049 | 0.11 | 1.91e-09 | NS |
| PTGES2 | 1.74e-13 | -1.121 | 0.047 | 0.106 | 4.28e-09 | NS |
| SLC9A3R1 | 6.73e-13 | -1.12 | 0.049 | 0.108 | 1.66e-08 | NS |
| C7orf50 | 6.94e-19 | -1.119 | 0.089 | 0.182 | 1.71e-14 | NS |
| FAM111A | 2.49e-14 | -1.117 | 0.05 | 0.113 | 6.15e-10 | NS |
| RALGDS | 5.63e-11 | -1.117 | 0.065 | 0.124 | 1.39e-06 | NS |
| GTF2B | 2.62e-26 | -1.115 | 0.168 | 0.304 | 6.45e-22 | NS |
| ATP6V0A1 | 2.77e-09 | -1.115 | 0.052 | 0.1 | 6.85e-05 | NS |
| BRI3 | 3.22e-26 | -1.112 | 0.098 | 0.217 | 7.95e-22 | NS |
| CHRAC1 | 1.31e-14 | -1.112 | 0.065 | 0.135 | 3.24e-10 | NS |
| CCDC80 | 1.96e-15 | -1.11 | 0.043 | 0.104 | 4.83e-11 | NS |
| HBEGF | 3.44e-15 | -1.109 | 0.075 | 0.151 | 8.49e-11 | NS |
| DAP | 4.01e-12 | -1.109 | 0.054 | 0.113 | 9.90e-08 | NS |
| STK38 | 1.92e-15 | -1.107 | 0.062 | 0.134 | 4.75e-11 | NS |
| RERE | 4.71e-18 | -1.105 | 0.067 | 0.149 | 1.16e-13 | NS |
| ADIPOR1 | 6.41e-22 | -1.104 | 0.092 | 0.196 | 1.58e-17 | NS |
| NRIP1 | 2.54e-10 | -1.104 | 0.052 | 0.104 | 6.28e-06 | NS |
| SRSF9 | 1.94e-24 | -1.103 | 0.09 | 0.2 | 4.79e-20 | NS |
| RGL1 | 3.72e-23 | -1.103 | 0.134 | 0.255 | 9.19e-19 | NS |
| GRINA | 4.67e-33 | -1.102 | 0.142 | 0.297 | 1.15e-28 | NS |
| HIVEP1 | 5.53e-12 | -1.102 | 0.063 | 0.125 | 1.37e-07 | NS |
| AREG | 3.32e-11 | -1.102 | 0.108 | 0.178 | 8.19e-07 | NS |
| GAA | 2.59e-24 | -1.101 | 0.091 | 0.201 | 6.39e-20 | NS |
| MAFB | 1.37e-21 | -1.1 | 0.174 | 0.299 | 3.38e-17 | NS |
| SKIL | 1.01e-17 | -1.097 | 0.088 | 0.177 | 2.49e-13 | NS |
| RHOB | 2.66e-17 | -1.097 | 0.152 | 0.257 | 6.56e-13 | NS |
| IVNS1ABP | 5.60e-38 | -1.095 | 0.219 | 0.404 | 1.38e-33 | NS |
| C1QA | 1.24e-47 | -1.092 | 0.383 | 0.592 | 3.05e-43 | NS |
| DOCK8 | 3.39e-37 | -1.09 | 0.164 | 0.336 | 8.37e-33 | NS |
| TGIF1 | 3.81e-26 | -1.085 | 0.145 | 0.277 | 9.41e-22 | NS |
| IFRD1 | 3.39e-18 | -1.085 | 0.083 | 0.171 | 8.37e-14 | NS |
| BAG3 | 7.11e-20 | -1.084 | 0.081 | 0.174 | 1.76e-15 | NS |
| POLR2A | 1.03e-18 | -1.083 | 0.129 | 0.234 | 2.55e-14 | NS |
| HLA-DOA | 1.66e-28 | -1.082 | 0.161 | 0.304 | 4.10e-24 | NS |
| MAT2A | 1.93e-24 | -1.082 | 0.233 | 0.373 | 4.77e-20 | NS |
| CFP | 4.27e-42 | -1.081 | 0.193 | 0.388 | 1.05e-37 | NS |
| LRRFIP2 | 2.35e-21 | -1.08 | 0.096 | 0.199 | 5.81e-17 | NS |
| SNX10 | 1.11e-31 | -1.079 | 0.15 | 0.301 | 2.74e-27 | NS |
| HINT2 | 1.91e-16 | -1.076 | 0.061 | 0.135 | 4.70e-12 | NS |
| UBAP1 | 6.26e-11 | -1.075 | 0.057 | 0.113 | 1.54e-06 | NS |
| PMP22 | 1.01e-15 | -1.074 | 0.119 | 0.21 | 2.49e-11 | NS |
| PYURF | 5.72e-18 | -1.072 | 0.083 | 0.172 | 1.41e-13 | NS |
| STAT2 | 4.98e-10 | -1.071 | 0.056 | 0.109 | 1.23e-05 | NS |
| LILRB2 | 3.38e-26 | -1.07 | 0.109 | 0.232 | 8.35e-22 | NS |
| RAPH1 | 2.10e-12 | -1.07 | 0.048 | 0.105 | 5.19e-08 | NS |
| PTPN18 | 2.25e-17 | -1.068 | 0.08 | 0.165 | 5.56e-13 | NS |
| MGLL | 4.63e-17 | -1.068 | 0.075 | 0.158 | 1.14e-12 | NS |
| ARHGEF2 | 8.53e-20 | -1.065 | 0.096 | 0.195 | 2.11e-15 | NS |
| SLC15A3 | 4.47e-14 | -1.059 | 0.07 | 0.141 | 1.10e-09 | NS |
| FNBP4 | 4.18e-22 | -1.057 | 0.1 | 0.208 | 1.03e-17 | NS |
| VAT1 | 4.85e-10 | -1.057 | 0.064 | 0.12 | 1.20e-05 | NS |
| EIF5 | 2.20e-55 | -1.056 | 0.283 | 0.521 | 5.44e-51 | NS |
| MID1IP1 | 1.50e-13 | -1.052 | 0.081 | 0.154 | 3.70e-09 | NS |
| MGP | 1.99e-20 | -1.049 | 0.04 | 0.112 | 4.90e-16 | NS |
| EPSTI1 | 9.11e-18 | -1.049 | 0.083 | 0.171 | 2.25e-13 | NS |
| TFRC | 5.24e-09 | -1.048 | 0.073 | 0.127 | 1.29e-04 | NS |
| IDH2 | 6.38e-18 | -1.047 | 0.091 | 0.183 | 1.57e-13 | NS |
| PLEKHO1 | 8.36e-23 | -1.045 | 0.132 | 0.252 | 2.06e-18 | NS |
| USP48 | 1.87e-10 | -1.045 | 0.05 | 0.101 | 4.62e-06 | NS |
| WSB1 | 4.86e-52 | -1.043 | 0.346 | 0.574 | 1.20e-47 | NS |
| JMJD1C | 1.18e-31 | -1.043 | 0.168 | 0.325 | 2.92e-27 | NS |
| NTAN1 | 1.85e-11 | -1.043 | 0.048 | 0.102 | 4.56e-07 | NS |
| C3AR1 | 1.99e-16 | -1.042 | 0.054 | 0.125 | 4.92e-12 | NS |
| C20orf194 | 4.64e-13 | -1.042 | 0.054 | 0.115 | 1.15e-08 | NS |
| ATP1A1 | 6.03e-20 | -1.04 | 0.131 | 0.241 | 1.49e-15 | NS |
| ADAM15 | 4.03e-19 | -1.038 | 0.089 | 0.184 | 9.96e-15 | NS |
| LRRC58 | 2.94e-11 | -1.037 | 0.058 | 0.115 | 7.27e-07 | NS |
| SLC25A24 | 3.43e-17 | -1.035 | 0.082 | 0.167 | 8.47e-13 | NS |
| PAXX | 6.75e-17 | -1.035 | 0.086 | 0.172 | 1.67e-12 | NS |
| FGD5-AS1 | 1.89e-13 | -1.034 | 0.074 | 0.144 | 4.65e-09 | NS |
| ST3GAL1 | 8.00e-15 | -1.033 | 0.064 | 0.135 | 1.98e-10 | NS |
| TRIM8 | 1.20e-12 | -1.033 | 0.072 | 0.139 | 2.96e-08 | NS |
| RBM33 | 9.91e-15 | -1.032 | 0.075 | 0.151 | 2.45e-10 | NS |
| PLD3 | 5.44e-32 | -1.031 | 0.187 | 0.35 | 1.34e-27 | NS |
| CASP1 | 1.31e-24 | -1.031 | 0.124 | 0.248 | 3.24e-20 | NS |
| MEF2A | 1.39e-24 | -1.027 | 0.127 | 0.252 | 3.43e-20 | NS |
| CSF1R | 3.24e-51 | -1.024 | 0.306 | 0.541 | 8.01e-47 | NS |
| SH3KBP1 | 2.44e-20 | -1.024 | 0.12 | 0.229 | 6.02e-16 | NS |

| **Table S4. Representative marker genes and functional characteristics of dendritic cell subtypes** | | | | | |
| --- | --- | --- | --- | --- | --- |
|  |  | **Representative genes** | | | |
| **Cluster** | **Functional properties** | **TF** | **cytokine, chemokine and effector molecule** | **cytokine receptor and chemokines receptor** | **other genes** |
| HSPA1B_cDC2 | CLEC10A | SPI1, FOS | CKLF, IFITM2, IFITM3 | —————————— | HSPA1B, HSPA1A, S100A4, TIMP1, FCER1G, CD1E, HLA-DRA, ITGB2 |
| MAF_moDC | CD11B,CD14,CD209 | MAF, MAFB, KLF4 | CCL13, CCL18, CCL14,CCL8, CCL2 | CD163, SIGLEC1, STAB1, CMKLR1, C3AR1, CCR1 | FOLR2, SELENOP, LGMN, MS4A4A, C1QA,MRC1 |
| CLEC9A_cDC1 | CLEC9A | IRF8, BATF3, ID2 | IL12A, CXCL9, CXCL10 | XCR1,CXCR3, CD226, | CADM1, TAP1, TAP2, PSMB8, HLA-DPA1, HLA-DPB1,PSMB9, |
| CD207_mDC | CCL19,LAMP3,CCR7 | IRF4, , ID2, HLX, ZEB1 | CCL17, CCL22, IL15, CCL19, IL32 | CCR7, IL7R, IL22RA2, CSF2RA,TNFRSF9, CD40 | CD207, FSCN1, KIF2A,LY75, CD83, PDCD1LG2, CD274, RGS1 |
| IL1B_DC | CLEC10A、CD11B、CD68 | NFKB1,NFKBIZ,CEBPB, KLF4 | IL1B, CXCL8, CXCL2, CXCL3, CCL3L1,CCL4 | IL1R2, IL13RA1, CX3CR1, CCRL2 | NLRP3, TNFAIP3, MAP3K8, DUSP2, BCL2A1, SOCS3, ICAM1, VSIR, CD83 |

| **Table S5. Pseudotime-Associated Highly Variable Genes Identified by GAM Analysis** | | | |
| --- | --- | --- | --- |
| **gene** | **waldStat** | **pvalue** | **meanLogFC** |
| RAB25 | 11.0844346 | 0.049731357 | 0.636570531 |
| BAZ2A | 11.08550104 | 0.049710857 | 0.543881385 |
| COL5A2 | 11.13530348 | 0.048762337 | 1.822117756 |
| PLA2G7 | 11.17943142 | 0.04793619 | 1.058461496 |
| COLGALT1 | 11.23697499 | 0.046878723 | 0.745418567 |
| MYC | 11.2823985 | 0.046059592 | 0.684552283 |
| GNG12 | 11.3206245 | 0.045380758 | 1.074247332 |
| SERPING1 | 11.34283564 | 0.04499068 | 0.899630966 |
| SIAH1 | 11.59970832 | 0.040704028 | 0.519743116 |
| COL6A5 | 11.61270746 | 0.040497736 | 2.930278496 |
| SLC7A8 | 11.62068297 | 0.040371658 | 1.200034355 |
| PPP6R1 | 11.67438876 | 0.039532319 | 0.567917501 |
| GATA3 | 11.74363641 | 0.038474515 | 1.494900106 |
| RBL2 | 11.76751305 | 0.038116056 | 0.636621331 |
| ACTA2 | 11.82353852 | 0.037287367 | 1.522932473 |
| USF1 | 11.99731775 | 0.034824552 | 0.574997974 |
| TRAPPC8 | 12.00124614 | 0.03477071 | 0.535768816 |
| CAV1 | 12.01163356 | 0.034628721 | 0.712060222 |
| ITSN1 | 12.02330201 | 0.03446988 | 1.248654673 |
| MSL1 | 12.08377438 | 0.033657735 | 0.650394404 |
| UFSP2 | 12.09977535 | 0.033445911 | 0.513702986 |
| SERPINE1 | 12.10621125 | 0.03336107 | 0.74089402 |
| HERC1 | 12.22853589 | 0.031787007 | 0.790355903 |
| PDZD11 | 12.3029654 | 0.030864192 | 0.693328406 |
| FTX | 12.31108469 | 0.030765086 | 0.5962049 |
| ZNF330 | 12.33388721 | 0.030488375 | 0.505424694 |
| OARD1 | 12.48555284 | 0.02870748 | 0.615787854 |
| CTSW | 12.58691704 | 0.027573148 | 0.568323762 |
| JKAMP | 12.67026425 | 0.026672685 | 0.635930683 |
| LLPH | 12.67306094 | 0.026642964 | 0.551174063 |
| WDFY3 | 12.73935246 | 0.025947677 | 0.613091093 |
| CNTRL | 12.85430928 | 0.024782979 | 0.538117129 |
| ZC3H13 | 12.86458558 | 0.024681339 | 0.558834991 |
| SERPINB13 | 12.86504152 | 0.024676838 | 2.2073273 |
| EIF4EBP3 | 12.87915321 | 0.024537939 | 0.725994422 |
| LBR | 12.93857176 | 0.023961268 | 0.533713754 |
| PDXDC1 | 12.98866711 | 0.023485201 | 0.575770427 |
| CYP27A1 | 13.02570852 | 0.023139039 | 2.295648522 |
| SPTBN1 | 13.15851292 | 0.021937758 | 0.76422784 |
| TNFAIP6 | 13.20565433 | 0.021525926 | 0.556360005 |
| TM2D1 | 13.23304577 | 0.021290054 | 0.761747046 |
| NOP16 | 13.24231832 | 0.021210771 | 0.55216329 |
| SNRNP27 | 13.24324864 | 0.021202832 | 0.599787883 |
| MMGT1 | 13.32552128 | 0.020511947 | 0.579123373 |
| HIPK3 | 13.39981388 | 0.019906715 | 0.507290571 |
| GNG11 | 13.40403507 | 0.019872847 | 0.667223916 |
| SPRR2A | 13.46578311 | 0.019383722 | 4.228714012 |
| LMO4 | 13.50834923 | 0.01905331 | 0.507053269 |
| HECTD1 | 13.52103882 | 0.018955864 | 0.575829951 |
| MGP | 13.59180613 | 0.018421161 | 0.93248979 |
| MDP1 | 13.69035575 | 0.017700686 | 0.676599895 |
| ARPIN | 13.8180164 | 0.016807673 | 0.533823172 |
| TNPO3 | 13.86419168 | 0.016495495 | 0.633690909 |
| TCEAL9 | 13.90789546 | 0.016205183 | 0.650621786 |
| DNM2 | 13.9167657 | 0.016146866 | 0.645544501 |
| PCF11 | 13.95358009 | 0.015906986 | 0.737538476 |
| CDV3 | 14.03199665 | 0.015407442 | 0.779587134 |
| CNOT1 | 14.04703202 | 0.015313406 | 0.521143627 |
| CCL24 | 14.18327551 | 0.014486196 | 5.452732086 |
| MAPK6 | 14.20839479 | 0.014338472 | 0.760870496 |
| KIAA1109 | 14.21266665 | 0.014313495 | 0.88636266 |
| ARHGEF1 | 14.24227003 | 0.014141558 | 0.563975041 |
| C12orf75 | 14.29731848 | 0.013827129 | 0.794469276 |
| GBP5 | 14.32936037 | 0.013647234 | 1.638754337 |
| IGKV3-11 | 14.33524925 | 0.013614419 | 2.672491548 |
| GM2A | 14.34104735 | 0.013582184 | 0.705869946 |
| SNHG3 | 14.34718979 | 0.013548116 | 0.506928917 |
| MT1M | 14.38174103 | 0.013358014 | 1.086122476 |
| NUP42 | 14.40268618 | 0.013244032 | 0.608159119 |
| CD163L1 | 14.45142533 | 0.012982428 | 1.892159893 |
| OPN3 | 14.55732447 | 0.012431144 | 0.729159871 |
| CORO7 | 14.61763879 | 0.012127365 | 0.640074683 |
| PPP1R21 | 14.6438572 | 0.011997566 | 0.583249091 |
| UBE2C | 14.65502837 | 0.011942671 | 0.612402332 |
| TMPO | 14.67262254 | 0.011856707 | 0.74163735 |
| SLC8B1 | 14.67815341 | 0.011829807 | 0.726807038 |
| SOX15 | 14.73172786 | 0.011572286 | 1.632179492 |
| MAPKAPK2 | 14.74121804 | 0.011527239 | 0.935158869 |
| DCD | 14.76807977 | 0.011400653 | 1.415282472 |
| ARHGAP15 | 14.82864614 | 0.011120156 | 0.623610112 |
| ZMAT2 | 14.88493207 | 0.010865488 | 0.557498144 |
| GON4L | 14.93131778 | 0.010659873 | 0.514398733 |
| UBR5 | 14.98858084 | 0.010411242 | 0.628287864 |
| SCOC | 15.07777152 | 0.01003515 | 0.671727323 |
| TIMP3 | 15.16533932 | 0.009678744 | 1.382618463 |
| LPL | 15.20470992 | 0.009522536 | 2.093115775 |
| GCHFR | 15.2195895 | 0.009464139 | 0.524506022 |
| ACTL6A | 15.2479641 | 0.009353742 | 0.616662246 |
| APOD | 15.27393143 | 0.009253807 | 0.712535829 |
| CXCL11 | 15.28547282 | 0.009209723 | 2.47322484 |
| UBE2Q2 | 15.29444397 | 0.009175598 | 0.864573367 |
| GPATCH2 | 15.31260004 | 0.00910691 | 0.500697005 |
| CDCP1 | 15.38148826 | 0.008850807 | 0.571249202 |
| DNAJC9 | 15.40071636 | 0.008780581 | 0.842803975 |
| TASOR | 15.44063859 | 0.0086365 | 0.794848738 |
| UBE3A | 15.51023796 | 0.00839079 | 0.670718598 |
| SRGAP2C | 15.62160945 | 0.008011682 | 0.609743784 |
| UBAP2L | 15.63456369 | 0.007968682 | 0.580721816 |
| EIF2AK2 | 15.71379948 | 0.007710516 | 0.558381309 |
| GIGYF2 | 15.85733313 | 0.007263421 | 0.637612939 |
| GLMP | 15.93857552 | 0.007021649 | 0.777738906 |
| SDAD1 | 16.01700938 | 0.006795679 | 0.660787985 |
| IGKC | 16.01989684 | 0.006787497 | 1.186196717 |
| CCL5 | 16.03820082 | 0.006735851 | 1.900015761 |
| CMKLR1 | 16.06434683 | 0.006662742 | 1.539242252 |
| UBE2R2 | 16.0735978 | 0.00663706 | 0.603277997 |
| TPD52L2 | 16.28046867 | 0.006087314 | 0.742034651 |
| SKI | 16.37194855 | 0.005858609 | 0.838777723 |
| PPP3R1 | 16.38549515 | 0.005825462 | 0.753147475 |
| HSPG2 | 16.51475827 | 0.00551823 | 1.382124488 |
| ZMYM2 | 16.62517968 | 0.005268346 | 0.814338014 |
| GALNT1 | 16.63793939 | 0.005240193 | 0.667084372 |
| ELAVL1 | 16.68529152 | 0.005136991 | 0.780409049 |
| PRSS3 | 16.69896794 | 0.005107555 | 2.189105423 |
| DOCK11 | 16.71505426 | 0.005073143 | 0.668181231 |
| ITGB7 | 16.73139074 | 0.005038427 | 0.671918985 |
| WWP1 | 16.91975808 | 0.004654519 | 1.3116752 |
| PXN | 16.97609971 | 0.004545348 | 0.785284924 |
| CERS2 | 17.02345399 | 0.00445553 | 0.500566431 |
| CDKN1C | 17.02455487 | 0.004453463 | 1.810127286 |
| EAPP | 17.10794479 | 0.004299562 | 0.588893128 |
| SS18 | 17.15562534 | 0.004213913 | 0.737097284 |
| TRH | 17.25520249 | 0.004040386 | 4.714110145 |
| CDC20 | 17.41951793 | 0.003769203 | 1.575704584 |
| AFF1 | 17.44491648 | 0.003728906 | 0.639721612 |
| HAT1 | 17.44694155 | 0.003725711 | 0.627859011 |
| PRRC2A | 17.74295195 | 0.003286424 | 0.727073083 |
| NUTM2A-AS1 | 17.9016061 | 0.003072281 | 0.597367712 |
| NRAS | 17.90599996 | 0.003066549 | 0.7645235 |
| EEA1 | 17.94090722 | 0.003021383 | 0.54746696 |
| ERGIC2 | 18.01446079 | 0.002928336 | 0.579825674 |
| GPATCH4 | 18.03671488 | 0.002900741 | 0.739278106 |
| PRDM1 | 18.06885404 | 0.002861338 | 0.734397535 |
| ELL | 18.0963808 | 0.002828007 | 0.829963214 |
| ZNF22 | 18.11512671 | 0.002805526 | 0.747745194 |
| CHCHD5 | 18.1816019 | 0.00272721 | 0.522935718 |
| SRGAP2 | 18.18920884 | 0.002718386 | 0.544357231 |
| ITGAV | 18.26126035 | 0.002636181 | 0.574256548 |
| CCNT2 | 18.33183137 | 0.002558029 | 0.771974338 |
| PIP4K2A | 18.33518909 | 0.002554368 | 0.524672313 |
| MFAP1 | 18.34794594 | 0.002540505 | 0.56311855 |
| STAG2 | 18.47796519 | 0.00240335 | 0.72001126 |
| PUM2 | 18.48054491 | 0.002400703 | 0.758741052 |
| ARHGEF12 | 18.62433399 | 0.002257626 | 0.569312801 |
| HOOK2 | 18.65469879 | 0.002228499 | 0.95983287 |
| MAP7D1 | 18.67439852 | 0.0022098 | 0.908058349 |
| TBC1D14 | 18.69584205 | 0.002189621 | 0.98419606 |
| WNK1 | 18.70662544 | 0.002179542 | 0.551821878 |
| POLR2H | 18.81820508 | 0.002077884 | 0.636059334 |
| NFAT5 | 18.85645543 | 0.002044118 | 0.664935712 |
| COL6A6 | 18.94177403 | 0.00197073 | 1.555346993 |
| SLC39A7 | 18.95204049 | 0.001962075 | 0.743520064 |
| ZC3HAV1 | 18.95367103 | 0.001960704 | 0.558665941 |
| TFPI2 | 18.99162363 | 0.001929054 | 2.568487946 |
| KDM4C | 18.99776242 | 0.001923982 | 0.522701492 |
| CALU | 19.07339799 | 0.001862555 | 0.52697819 |
| KLF5 | 19.09588585 | 0.001844666 | 0.552816206 |
| CEBPZOS | 19.10811992 | 0.001835005 | 0.6221684 |
| XAF1 | 19.11956952 | 0.001826008 | 0.673390899 |
| ATP6V0A1 | 19.13527211 | 0.00181374 | 0.922996823 |
| PIP5K1A | 19.17670064 | 0.00178176 | 0.765277042 |
| OXR1 | 19.29966445 | 0.001690066 | 0.986069105 |
| EHD4 | 19.31151364 | 0.001681479 | 0.643755473 |
| EIF4EBP2 | 19.42325034 | 0.001602583 | 0.689662447 |
| FRY | 19.44620864 | 0.001586828 | 1.101973586 |
| XPO1 | 19.52460664 | 0.001534169 | 0.549514836 |
| BAG5 | 19.57018105 | 0.00150435 | 0.690936592 |
| SNHG16 | 19.70288659 | 0.00142073 | 0.752680205 |
| CD8A | 19.76344418 | 0.001384106 | 0.749585214 |
| ZBTB7A | 19.86748749 | 0.001323339 | 1.085921788 |
| TNC | 19.9070674 | 0.001300918 | 1.591273132 |
| CCDC170 | 19.91663659 | 0.001295553 | 1.443969953 |
| MRPS14 | 19.95612472 | 0.001273645 | 0.732528478 |
| SNAI1 | 20.00728337 | 0.001245804 | 1.67386547 |
| ADAP2 | 20.09371172 | 0.001200122 | 1.005878207 |
| NIN | 20.11697277 | 0.001188111 | 0.816761141 |
| FABP4 | 20.15825463 | 0.001167086 | 2.33811362 |
| SAFB2 | 20.17255618 | 0.001159888 | 0.806447616 |
| DSC2 | 20.19228555 | 0.001150029 | 0.579790045 |
| LGALS3BP | 20.23534937 | 0.001128796 | 1.154606322 |
| PLAC9 | 20.26434971 | 0.001114716 | 2.237951966 |
| SIRPA | 20.2858554 | 0.001104386 | 0.818820504 |
| GPR171 | 20.39329347 | 0.001054173 | 1.622702009 |
| LARP7 | 20.51664517 | 0.000999289 | 0.755028009 |
| SLC20A1 | 20.56982368 | 0.000976505 | 0.705291391 |
| GSTM4 | 20.59667336 | 0.000965197 | 0.503225789 |
| BBIP1 | 20.67301748 | 0.000933742 | 1.161238744 |
| ARIH1 | 20.68884008 | 0.00092735 | 1.018519076 |
| RIPOR2 | 20.73258194 | 0.000909904 | 0.845681936 |
| PPP1R14B | 20.73278589 | 0.000909823 | 0.516728601 |
| PCOLCE | 20.90933442 | 0.000842638 | 1.065758637 |
| CRABP2 | 20.9206873 | 0.000838489 | 1.339006431 |
| DCAF12 | 20.93097251 | 0.000834748 | 0.57973475 |
| DGKZ | 20.9378615 | 0.000832251 | 0.504158124 |
| DMAC1 | 20.94441108 | 0.000829884 | 1.02387319 |
| RAD21 | 20.95447696 | 0.000826259 | 0.559630843 |
| DHX15 | 21.01194372 | 0.000805862 | 0.573362029 |
| TEN1 | 21.09308053 | 0.000777905 | 0.630524827 |
| GAPVD1 | 21.16670935 | 0.000753363 | 0.585480597 |
| PLAC8 | 21.22359972 | 0.000734925 | 2.783478395 |
| FLT1 | 21.29323936 | 0.000712959 | 0.838193088 |
| NRIP1 | 21.36559664 | 0.000690821 | 0.566449748 |
| ANAPC13 | 21.40643044 | 0.000678628 | 0.584213227 |
| LRRK2 | 21.47424162 | 0.000658848 | 0.938243301 |
| COL6A3 | 21.64969678 | 0.000610267 | 1.999430577 |
| ECM1 | 21.76608323 | 0.000580008 | 0.616810887 |
| TRIM8 | 21.79422203 | 0.000572917 | 0.897863894 |
| CEMIP2 | 21.91326877 | 0.000543853 | 1.619183187 |
| MX1 | 22.06811257 | 0.000508215 | 0.599023458 |
| COLEC12 | 22.08392687 | 0.000504708 | 1.76451888 |
| LRMP | 22.15762094 | 0.000488677 | 0.612582464 |
| MILR1 | 22.16230176 | 0.000487675 | 0.7567828 |
| MAN1A2 | 22.28981539 | 0.000461168 | 0.523377481 |
| PAFAH1B3 | 22.31687261 | 0.000455729 | 0.531569651 |
| MKNK2 | 22.34211864 | 0.000450711 | 0.877648273 |
| MKNK1 | 22.344732 | 0.000450195 | 0.813199884 |
| SNHG12 | 22.43844814 | 0.000432061 | 0.529795067 |
| ETNK1 | 22.46636675 | 0.0004268 | 0.525863113 |
| UBA3 | 22.52125288 | 0.00041664 | 0.608272157 |
| TLE4 | 22.53565262 | 0.000414014 | 0.724942094 |
| FGD5-AS1 | 22.58905737 | 0.000404418 | 0.856333854 |
| FNBP4 | 22.61901986 | 0.000399131 | 0.763485248 |
| ARL14EP | 22.7510499 | 0.000376633 | 1.325878479 |
| TARS | 22.82643271 | 0.000364354 | 0.781928716 |
| CUL4A | 22.87053962 | 0.000357354 | 1.020422861 |
| H1F0 | 23.0868017 | 0.000324908 | 0.810163623 |
| FKBP15 | 23.10060096 | 0.00032294 | 0.563504625 |
| HIF1A | 23.19628725 | 0.00030961 | 0.69747017 |
| TPP2 | 23.35444728 | 0.000288759 | 0.691238388 |
| IQSEC1 | 23.38166147 | 0.000285313 | 1.145999434 |
| COL17A1 | 23.38255777 | 0.000285201 | 1.212033974 |
| PDCD4 | 23.52637022 | 0.000267665 | 0.800267013 |
| ERO1A | 23.53004667 | 0.000267231 | 0.917592856 |
| NCK1 | 23.75864587 | 0.000241567 | 0.657989198 |
| COL6A2 | 23.77893172 | 0.000239411 | 0.785322152 |
| RBBP6 | 23.93313344 | 0.000223631 | 0.88720816 |
| RCC2 | 23.93951088 | 0.000223001 | 1.037912974 |
| ZFHX3 | 24.4231254 | 0.000180008 | 0.799899642 |
| ITPRID2 | 24.46605476 | 0.000176614 | 0.804974601 |
| CCDC6 | 24.46897364 | 0.000176385 | 0.938271451 |
| KIAA0100 | 24.48585773 | 0.000175069 | 0.694219045 |
| SLC9A9 | 24.57907646 | 0.000167977 | 0.856830716 |
| ARMCX3 | 24.59693894 | 0.000166651 | 0.704282488 |
| RGS18 | 24.61815111 | 0.00016509 | 3.734548547 |
| HECA | 24.70484573 | 0.000158857 | 0.828062873 |
| GIMAP1 | 24.71646772 | 0.00015804 | 0.884841556 |
| MRFAP1L1 | 24.85987741 | 0.000148287 | 0.764071231 |
| PKP1 | 24.870618 | 0.000147581 | 2.435185353 |
| PRMT7 | 24.9379213 | 0.000143232 | 0.581629925 |
| POSTN | 24.97566308 | 0.000140849 | 1.53863458 |
| LYRM2 | 24.98053576 | 0.000140545 | 0.903175307 |
| CLCA2 | 25.0492452 | 0.000136316 | 1.57326637 |
| OSBPL11 | 25.0845611 | 0.000134192 | 0.535844838 |
| MYCBP2 | 25.13235442 | 0.000131369 | 0.71401995 |
| KPNA2 | 25.18594977 | 0.000128274 | 0.721932075 |
| MRPL17 | 25.19779105 | 0.000127599 | 1.115783521 |
| ABHD2 | 25.2098536 | 0.000126916 | 0.515243313 |
| SNCA | 25.26153849 | 0.00012403 | 1.085617731 |
| TTC7A | 25.28805201 | 0.000122575 | 0.740169848 |
| STX4 | 25.30607129 | 0.000121596 | 0.534683253 |
| EXOC6 | 25.41917925 | 0.000115623 | 0.668363841 |
| WAPL | 25.45844584 | 0.000113618 | 0.683002163 |
| RHOQ | 25.50853107 | 0.00011111 | 1.045583376 |
| NMI | 25.52719614 | 0.00011019 | 0.989987799 |
| TPM2 | 25.58442542 | 0.000107415 | 0.846152795 |
| RERE | 25.59677924 | 0.000106825 | 0.860563132 |
| BCOR | 25.63184359 | 0.000105168 | 0.779735012 |
| PRKACA | 25.7998594 | 9.76E-05 | 0.547505474 |
| LINC00963 | 25.91203941 | 9.28E-05 | 1.399820176 |
| LILRA6 | 25.94529096 | 9.14E-05 | 2.354040219 |
| SERTAD2 | 25.96091887 | 9.08E-05 | 0.742003783 |
| GPR34 | 26.0718937 | 8.64E-05 | 2.282487677 |
| TBC1D9 | 26.13696216 | 8.39E-05 | 0.934340188 |
| GOLGA4 | 26.17461048 | 8.25E-05 | 0.841428423 |
| IL6R | 26.24753923 | 7.99E-05 | 0.551240131 |
| C6orf62 | 26.29375319 | 7.83E-05 | 0.767241744 |
| SPRR2E | 26.55131037 | 6.97E-05 | 1.852903847 |
| IL17RA | 26.67925364 | 6.59E-05 | 1.239132462 |
| BEST1 | 26.72979928 | 6.44E-05 | 1.212223512 |
| RNFT1 | 26.81307266 | 6.20E-05 | 0.667389134 |
| CBWD5 | 26.93138687 | 5.88E-05 | 0.523313838 |
| NIPSNAP3A | 26.93178211 | 5.88E-05 | 0.830007841 |
| RAP2B | 27.05079192 | 5.58E-05 | 0.902626067 |
| NUMA1 | 27.07229411 | 5.52E-05 | 0.775944835 |
| ATP2A2 | 27.07255322 | 5.52E-05 | 0.596784408 |
| MBD4 | 27.08924383 | 5.48E-05 | 1.004581449 |
| SLC8A1 | 27.11313329 | 5.42E-05 | 0.727703928 |
| TTYH3 | 27.13038079 | 5.38E-05 | 1.51786545 |
| GPR65 | 27.1882926 | 5.24E-05 | 0.563285147 |
| SLC46A3 | 27.21787429 | 5.17E-05 | 0.817094944 |
| BRAF | 27.28339219 | 5.02E-05 | 1.154107616 |
| OAZ2 | 27.39552102 | 4.78E-05 | 0.801651401 |
| NMB | 27.43555397 | 4.69E-05 | 2.618612276 |
| NUDT4 | 27.45005222 | 4.66E-05 | 1.3354651 |
| ATM | 27.46494556 | 4.63E-05 | 0.603681136 |
| RANGRF | 27.55914769 | 4.44E-05 | 0.535207509 |
| ABCA6 | 27.82628114 | 3.94E-05 | 1.204443766 |
| TNFRSF18 | 27.90513429 | 3.80E-05 | 0.804599039 |
| JAK2 | 27.9466621 | 3.73E-05 | 0.755107971 |
| HTT | 27.95671855 | 3.71E-05 | 0.510770288 |
| MIR205HG | 27.98601165 | 3.66E-05 | 1.213398369 |
| EVI5 | 28.03627994 | 3.58E-05 | 0.675265871 |
| ARRDC3 | 28.09889578 | 3.48E-05 | 0.830420816 |
| GUSB | 28.17019469 | 3.37E-05 | 0.666429627 |
| CASP3 | 28.27441016 | 3.22E-05 | 0.541837421 |
| PUM3 | 28.2954652 | 3.19E-05 | 0.810641575 |
| TIMP2 | 28.32498653 | 3.14E-05 | 0.651395993 |
| PHF14 | 28.35916434 | 3.10E-05 | 0.897608491 |
| DSG3 | 28.44669352 | 2.98E-05 | 2.783039994 |
| PURB | 28.66782532 | 2.69E-05 | 0.827697486 |
| NUDT3 | 28.7427147 | 2.60E-05 | 0.619259255 |
| DENND4A | 28.7545471 | 2.59E-05 | 0.684606238 |
| VCL | 28.78298973 | 2.56E-05 | 0.779295372 |
| ANKRD28 | 28.83621352 | 2.50E-05 | 0.592401449 |
| STAT1 | 28.86985363 | 2.46E-05 | 0.973258773 |
| CYP1B1 | 29.06836896 | 2.25E-05 | 2.216611051 |
| CTTNBP2 | 29.10038756 | 2.22E-05 | 0.892799525 |
| TMEM106A | 29.12005973 | 2.20E-05 | 0.999561331 |
| GNL3 | 29.1460453 | 2.17E-05 | 0.613445268 |
| LILRB5 | 29.16701232 | 2.15E-05 | 4.531154977 |
| CAVIN2 | 29.19715589 | 2.12E-05 | 1.702727609 |
| HPGD | 29.27131538 | 2.05E-05 | 0.686960047 |
| VAT1 | 29.29755092 | 2.03E-05 | 1.675734159 |
| SMIM25 | 29.46653292 | 1.88E-05 | 3.218626694 |
| TMEM167A | 29.47180542 | 1.87E-05 | 0.530590609 |
| ADM | 29.540486 | 1.82E-05 | 1.471653121 |
| KLF9 | 29.56020157 | 1.80E-05 | 0.503301749 |
| CD209 | 29.62794935 | 1.75E-05 | 1.76233237 |
| IGF2R | 29.74323299 | 1.66E-05 | 0.680143578 |
| EPB41L3 | 29.86914023 | 1.56E-05 | 1.375171293 |
| COL3A1 | 29.96137352 | 1.50E-05 | 1.261946086 |
| GRK3 | 29.97344663 | 1.49E-05 | 0.514245174 |
| DTX3L | 30.01778807 | 1.46E-05 | 1.094892089 |
| MED13L | 30.04257145 | 1.45E-05 | 0.529705111 |
| SAFB | 30.13546069 | 1.39E-05 | 0.63444035 |
| UPF3A | 30.24106556 | 1.32E-05 | 0.611996751 |
| ATP8B4 | 30.25666204 | 1.31E-05 | 1.229891421 |
| CXADR | 30.26628079 | 1.31E-05 | 2.17381687 |
| BIN2 | 30.34555615 | 1.26E-05 | 0.659621807 |
| MAPRE2 | 30.41426294 | 1.22E-05 | 0.669888951 |
| STK10 | 30.54768182 | 1.15E-05 | 0.805289689 |
| SUSD6 | 30.55796167 | 1.15E-05 | 0.540502574 |
| ABHD5 | 30.56390568 | 1.14E-05 | 0.824751592 |
| IGF1 | 30.7114343 | 1.07E-05 | 3.503656012 |
| CARHSP1 | 30.78662343 | 1.03E-05 | 0.608400392 |
| AAK1 | 30.80692628 | 1.02E-05 | 1.288200496 |
| ATP13A3 | 30.8616611 | 9.98E-06 | 0.642327096 |
| TP63 | 30.87277303 | 9.92E-06 | 0.597198607 |
| TRIB1 | 30.98100395 | 9.45E-06 | 0.721443979 |
| POP5 | 30.99544711 | 9.39E-06 | 0.638684167 |
| LIMA1 | 31.06732619 | 9.08E-06 | 0.839352003 |
| GOLIM4 | 31.09643826 | 8.97E-06 | 0.721319689 |
| VEZT | 31.12095926 | 8.87E-06 | 0.972361777 |
| MYOF | 31.13478421 | 8.81E-06 | 0.514878913 |
| LGALS8 | 31.16170666 | 8.70E-06 | 0.881347537 |
| TVP23B | 31.37054663 | 7.91E-06 | 0.542061235 |
| RREB1 | 31.42845225 | 7.71E-06 | 0.739131239 |
| HEXIM1 | 31.63397628 | 7.02E-06 | 0.848115161 |
| IFRD1 | 31.66447418 | 6.92E-06 | 0.982706364 |
| SCN9A | 31.67518914 | 6.89E-06 | 0.591719469 |
| HYOU1 | 31.75090847 | 6.66E-06 | 0.554424242 |
| COL6A1 | 31.84299199 | 6.38E-06 | 1.96972109 |
| FKBP3 | 31.87164897 | 6.30E-06 | 0.862286021 |
| RRP7A | 31.88779903 | 6.25E-06 | 0.850834168 |
| REV3L | 31.9889406 | 5.97E-06 | 1.092906885 |
| RB1CC1 | 32.16939097 | 5.50E-06 | 0.995122395 |
| RARS | 32.18946417 | 5.45E-06 | 0.546916981 |
| HVCN1 | 32.34461846 | 5.08E-06 | 1.261080628 |
| KCNQ1OT1 | 32.46687557 | 4.80E-06 | 1.681116262 |
| SCARB1 | 32.58015635 | 4.56E-06 | 1.612395646 |
| NUP58 | 32.68945989 | 4.34E-06 | 0.895738974 |
| TMEM126A | 32.70571767 | 4.31E-06 | 0.758639426 |
| LUZP1 | 32.71715724 | 4.28E-06 | 0.550075806 |
| SATB1 | 32.85701291 | 4.02E-06 | 0.765527071 |
| DGKA | 32.8813037 | 3.97E-06 | 0.637271296 |
| LRRC25 | 32.92834666 | 3.89E-06 | 2.452108186 |
| WARS | 32.93839352 | 3.87E-06 | 0.59333854 |
| AZIN1 | 33.11711745 | 3.57E-06 | 0.66338228 |
| AKAP9 | 33.1515256 | 3.51E-06 | 0.620854863 |
| ZMPSTE24 | 33.19046785 | 3.45E-06 | 0.73082796 |
| UBLCP1 | 33.27810919 | 3.31E-06 | 0.752652738 |
| ISG20L2 | 33.4256143 | 3.10E-06 | 1.112046314 |
| MANBA | 33.45510489 | 3.06E-06 | 1.071261323 |
| LINC01504 | 33.51275733 | 2.98E-06 | 0.852044767 |
| UBE2E3 | 33.53441767 | 2.95E-06 | 1.022102761 |
| RXRA | 33.70231555 | 2.73E-06 | 0.921774466 |
| KIAA0930 | 33.82339173 | 2.58E-06 | 0.810553863 |
| STXBP2 | 34.0509759 | 2.33E-06 | 0.566716177 |
| ARAP1 | 34.05259835 | 2.32E-06 | 0.768592626 |
| SMIM30 | 34.1049075 | 2.27E-06 | 0.996241064 |
| ARID3A | 34.15148867 | 2.22E-06 | 0.537738401 |
| SOX4 | 34.16377194 | 2.21E-06 | 0.752465279 |
| EP300 | 34.17172905 | 2.20E-06 | 1.010122473 |
| RTCB | 34.20917439 | 2.16E-06 | 0.635434501 |
| TYRP1 | 34.3173449 | 2.06E-06 | 2.187322505 |
| CCL27 | 34.41167936 | 1.97E-06 | 3.029731861 |
| NIPSNAP2 | 34.46511026 | 1.92E-06 | 1.276502137 |
| MIR181A1HG | 34.5348936 | 1.86E-06 | 0.96566796 |
| MAF | 34.54643322 | 1.85E-06 | 1.02944141 |
| COL1A2 | 34.59055271 | 1.82E-06 | 1.494760521 |
| RANBP2 | 34.66467381 | 1.76E-06 | 0.755042147 |
| SEMA4A | 34.73567945 | 1.70E-06 | 1.45374652 |
| CALML5 | 34.75247755 | 1.69E-06 | 1.824467941 |
| PSMG3 | 34.80945867 | 1.64E-06 | 0.53729953 |
| ARL4D | 35.07301005 | 1.46E-06 | 1.381784185 |
| NUDT5 | 35.09226178 | 1.44E-06 | 1.061306439 |
| CLN8 | 35.10785008 | 1.43E-06 | 0.882660777 |
| PAM16 | 35.16308741 | 1.40E-06 | 1.060222329 |
| RIN3 | 35.28194951 | 1.32E-06 | 1.317187411 |
| PLAAT4 | 35.42728749 | 1.24E-06 | 1.20443582 |
| KCTD20 | 35.51819318 | 1.19E-06 | 0.915593228 |
| GBP1 | 35.55058364 | 1.17E-06 | 0.893680097 |
| CCND1 | 35.55724638 | 1.16E-06 | 0.842980749 |
| CCL20 | 35.56770337 | 1.16E-06 | 4.384580333 |
| SLC38A6 | 35.74510063 | 1.07E-06 | 0.605950055 |
| FAM111A | 35.80000446 | 1.04E-06 | 1.171304844 |
| NDEL1 | 35.86676261 | 1.01E-06 | 0.996215086 |
| SGPL1 | 35.93439345 | 9.79E-07 | 0.934034187 |
| NPTN | 35.93664407 | 9.78E-07 | 0.679217641 |
| OGA | 36.11692098 | 9.00E-07 | 0.71158672 |
| A2M | 36.19354239 | 8.69E-07 | 1.349413125 |
| LRRC8C | 36.26929168 | 8.39E-07 | 0.507462844 |
| QKI | 36.78682935 | 6.61E-07 | 0.63964623 |
| DLGAP4 | 36.84878363 | 6.42E-07 | 0.528906689 |
| FCGR3A | 37.07811386 | 5.78E-07 | 4.931644627 |
| HSPD1 | 37.08194666 | 5.77E-07 | 0.652347271 |
| BEX4 | 37.10533031 | 5.71E-07 | 0.645076947 |
| FGFBP1 | 37.20244439 | 5.46E-07 | 1.343321693 |
| EIF4E | 37.23970823 | 5.36E-07 | 0.603592786 |
| TREM2 | 37.25610652 | 5.32E-07 | 1.875386886 |
| NEMF | 37.39091021 | 5.00E-07 | 0.708717285 |
| ZNRD2 | 37.47358733 | 4.81E-07 | 0.8576865 |
| GRK2 | 37.53643672 | 4.68E-07 | 0.968544159 |
| PIP4P2 | 37.67943566 | 4.38E-07 | 0.898205584 |
| TUBB4B | 37.69824976 | 4.34E-07 | 0.586476111 |
| KIT | 37.78586665 | 4.17E-07 | 4.174420757 |
| SIGLEC1 | 37.92706936 | 3.90E-07 | 2.094199335 |
| IGFBP4 | 38.04528917 | 3.70E-07 | 1.81720889 |
| CYBRD1 | 38.08031316 | 3.64E-07 | 0.662330745 |
| TMEM150C | 38.5126294 | 2.98E-07 | 11.43082361 |
| RYBP | 38.52662443 | 2.96E-07 | 1.050433871 |
| CHCHD1 | 38.52919522 | 2.95E-07 | 1.184427928 |
| CRABP1 | 38.58728781 | 2.88E-07 | 4.796222046 |
| DDX27 | 38.70918555 | 2.72E-07 | 0.757451749 |
| IQGAP2 | 38.76251036 | 2.65E-07 | 0.745059675 |
| SLCO2B1 | 38.77894561 | 2.63E-07 | 1.367912068 |
| CSRP2 | 38.79567753 | 2.61E-07 | 0.751281312 |
| DNAJB4 | 38.80172643 | 2.60E-07 | 1.166851044 |
| SH3TC1 | 38.83648957 | 2.56E-07 | 0.947496863 |
| SH3BP2 | 38.9602878 | 2.42E-07 | 0.755557573 |
| CXCR4 | 39.00007176 | 2.37E-07 | 0.74620722 |
| SPEN | 39.03163656 | 2.34E-07 | 0.848576348 |
| MX2 | 39.12900619 | 2.24E-07 | 1.191900736 |
| IL6ST | 39.20198871 | 2.16E-07 | 0.802998834 |
| WDFY4 | 39.23115399 | 2.13E-07 | 0.935080108 |
| PRKACB | 39.26201516 | 2.10E-07 | 0.893133712 |
| MIR22HG | 39.30004263 | 2.07E-07 | 1.214635215 |
| CALHM2 | 39.3999947 | 1.97E-07 | 0.562650373 |
| ANO6 | 39.42907554 | 1.95E-07 | 0.86009415 |
| P2RY14 | 39.46537423 | 1.91E-07 | 0.500062338 |
| MRTO4 | 39.48760547 | 1.89E-07 | 1.396683343 |
| WAC-AS1 | 39.59929942 | 1.80E-07 | 1.326174043 |
| AMD1 | 39.65664982 | 1.75E-07 | 0.56483075 |
| SMCHD1 | 39.6609447 | 1.75E-07 | 0.983421099 |
| ARHGAP5 | 39.68661875 | 1.73E-07 | 1.649803954 |
| AASDHPPT | 40.00612502 | 1.49E-07 | 1.058258584 |
| NSD3 | 40.07094481 | 1.44E-07 | 1.131396535 |
| MRPL50 | 40.2687721 | 1.32E-07 | 1.222489654 |
| SBSN | 40.32150811 | 1.29E-07 | 1.692240796 |
| DNAJA4 | 40.46460241 | 1.20E-07 | 1.542785113 |
| MCOLN1 | 40.513846 | 1.18E-07 | 1.819210296 |
| SPG11 | 40.54691533 | 1.16E-07 | 0.612587683 |
| HRH2 | 40.62992802 | 1.11E-07 | 1.475820925 |
| TRMT1 | 40.71754853 | 1.07E-07 | 0.554062684 |
| TFEC | 40.7662281 | 1.05E-07 | 0.919735826 |
| SLC39A8 | 40.84293262 | 1.01E-07 | 1.639721875 |
| TFRC | 40.84759335 | 1.01E-07 | 1.243122226 |
| DPM2 | 40.94933667 | 9.61E-08 | 0.763138077 |
| SPP1 | 40.99787938 | 9.39E-08 | 2.402361658 |
| GBP2 | 41.01911829 | 9.30E-08 | 1.135188419 |
| WIPI2 | 41.09569178 | 8.97E-08 | 1.19962508 |
| DDX3Y | 41.15596696 | 8.73E-08 | 1.257937769 |
| FXYD3 | 41.20220351 | 8.54E-08 | 1.507173608 |
| RIN2 | 41.22922307 | 8.43E-08 | 1.273758589 |
| PTTG1 | 41.51941509 | 7.37E-08 | 0.827824926 |
| ITM2C | 41.52957229 | 7.33E-08 | 0.668382819 |
| TREM1 | 41.76868484 | 6.56E-08 | 2.433208691 |
| PBDC1 | 41.78146432 | 6.52E-08 | 0.686348696 |
| PNPLA8 | 42.10079398 | 5.62E-08 | 0.885283278 |
| GCNT1 | 42.11100863 | 5.59E-08 | 0.663738801 |
| DCT | 42.19934882 | 5.37E-08 | 4.252070325 |
| ADAM10 | 42.35224067 | 5.00E-08 | 0.719218736 |
| BATF | 42.39854407 | 4.89E-08 | 1.457275769 |
| AC020656.1 | 42.46711169 | 4.74E-08 | 1.233404358 |
| COMMD4 | 42.49636514 | 4.67E-08 | 0.959621164 |
| ASPH | 42.51873109 | 4.63E-08 | 0.644706259 |
| DERL2 | 42.720881 | 4.21E-08 | 0.744890444 |
| GYPC | 42.83491674 | 3.99E-08 | 1.519339043 |
| KIF1B | 42.850318 | 3.96E-08 | 1.679873912 |
| MGAT4A | 42.88839617 | 3.89E-08 | 0.848083721 |
| RB1 | 42.91599002 | 3.84E-08 | 0.840475411 |
| NPL | 42.93814272 | 3.80E-08 | 2.807776568 |
| SCFD1 | 42.9424275 | 3.80E-08 | 0.715199357 |
| PAG1 | 42.99698655 | 3.70E-08 | 1.718573351 |
| LILRB1 | 43.19809051 | 3.37E-08 | 1.519991161 |
| MITF | 43.25440336 | 3.28E-08 | 2.658137591 |
| TMEM131 | 43.28535188 | 3.23E-08 | 1.020981896 |
| SCARB2 | 43.56290394 | 2.84E-08 | 1.33936648 |
| TMEM97 | 43.68131402 | 2.69E-08 | 0.654182164 |
| TMEM173 | 43.81068022 | 2.53E-08 | 0.657033656 |
| MBOAT7 | 43.96189107 | 2.36E-08 | 0.550143395 |
| DOCK2 | 43.9649652 | 2.35E-08 | 0.525017674 |
| APOE | 44.07984698 | 2.23E-08 | 2.236761331 |
| RRAD | 44.16932587 | 2.14E-08 | 2.410056965 |
| TSC22D2 | 44.17205135 | 2.14E-08 | 1.158842026 |
| DSG1 | 44.39949498 | 1.92E-08 | 1.49241391 |
| GIMAP7 | 44.41990736 | 1.90E-08 | 0.680554901 |
| FXYD2 | 44.47807118 | 1.85E-08 | 1.057902173 |
| PLEKHO2 | 44.56499397 | 1.78E-08 | 1.306579519 |
| OLFML2B | 44.56608007 | 1.78E-08 | 3.416731896 |
| FES | 44.61018058 | 1.74E-08 | 0.702874305 |
| CCL13 | 44.66020975 | 1.70E-08 | 1.033774107 |
| PPP1CB | 44.96493458 | 1.47E-08 | 0.687142424 |
| STK32C | 44.99107789 | 1.46E-08 | 0.827074389 |
| HIST1H4C | 45.06278255 | 1.41E-08 | 0.911778001 |
| SLAMF7 | 45.18347157 | 1.33E-08 | 0.858659054 |
| JUND | 45.21598133 | 1.31E-08 | 0.827850019 |
| SERPINB5 | 45.50184764 | 1.15E-08 | 2.201973953 |
| USP36 | 45.51327111 | 1.14E-08 | 1.209035579 |
| TRAPPC2L | 45.61760362 | 1.09E-08 | 0.937301465 |
| RAB32 | 45.68943638 | 1.05E-08 | 1.289935015 |
| STK38 | 46.33260378 | 7.77E-09 | 1.275741717 |
| KRT2 | 46.37779206 | 7.61E-09 | 1.760623345 |
| TET2 | 46.54760867 | 7.03E-09 | 0.515387846 |
| CARM1 | 46.58497359 | 6.90E-09 | 0.877733531 |
| UBAC2 | 46.72276083 | 6.47E-09 | 1.281912269 |
| LRIF1 | 47.00013046 | 5.68E-09 | 0.967354603 |
| REXO2 | 47.01826214 | 5.63E-09 | 1.037374264 |
| IDH1 | 47.02504875 | 5.62E-09 | 0.63407817 |
| ADGRE2 | 47.11637309 | 5.38E-09 | 1.760056922 |
| NAPRT | 47.45372453 | 4.59E-09 | 1.395108521 |
| CLEC12A | 47.46215061 | 4.57E-09 | 4.232539129 |
| GPRIN3 | 47.50071028 | 4.49E-09 | 1.007534959 |
| P2RY13 | 47.50390828 | 4.48E-09 | 1.123543565 |
| ATP6V1D | 47.67866379 | 4.13E-09 | 0.717120536 |
| ACOT13 | 47.71508234 | 4.06E-09 | 0.614040358 |
| AC004687.1 | 47.83089203 | 3.85E-09 | 0.809527112 |
| TMEM60 | 48.01763416 | 3.52E-09 | 1.46949453 |
| C9orf72 | 48.03798028 | 3.49E-09 | 0.813013077 |
| SAR1B | 48.15880397 | 3.30E-09 | 0.715147356 |
| PRKCD | 48.20279189 | 3.23E-09 | 1.056134064 |
| CCR2 | 48.35670401 | 3.00E-09 | 2.042193368 |
| SFT2D2 | 48.37398681 | 2.98E-09 | 0.606957306 |
| HEXA | 48.58512811 | 2.70E-09 | 0.654434125 |
| CRYL1 | 48.68436786 | 2.57E-09 | 1.393728556 |
| AQP9 | 48.74242996 | 2.51E-09 | 4.330715719 |
| CEBPA | 48.78184028 | 2.46E-09 | 0.74637607 |
| CXCL1 | 48.8548764 | 2.38E-09 | 3.404466684 |
| TNFRSF11B | 48.88480153 | 2.34E-09 | 3.718392543 |
| BATF3 | 49.09844089 | 2.12E-09 | 1.284772518 |
| KCNAB2 | 49.1112462 | 2.11E-09 | 1.162550985 |
| SNAPC1 | 49.2810077 | 1.94E-09 | 1.495209886 |
| SMIM4 | 49.31511604 | 1.91E-09 | 1.103552882 |
| CD47 | 49.34234577 | 1.89E-09 | 1.140300436 |
| SPRED1 | 49.52761339 | 1.73E-09 | 1.123343479 |
| CCNL1 | 49.55027096 | 1.71E-09 | 0.710093504 |
| MED31 | 49.7930414 | 1.53E-09 | 0.900906759 |
| MPP1 | 50.10693312 | 1.32E-09 | 1.098406184 |
| CD300A | 50.2578317 | 1.23E-09 | 0.886067695 |
| DPYSL2 | 50.36609948 | 1.17E-09 | 0.665983575 |
| BNIP3L | 50.44390361 | 1.12E-09 | 0.884301938 |
| PLOD3 | 50.51858386 | 1.09E-09 | 0.574261856 |
| FZD2 | 50.60329022 | 1.04E-09 | 1.009358859 |
| KRT6B | 50.61046996 | 1.04E-09 | 1.876642469 |
| AP1B1 | 50.78790607 | 9.56E-10 | 0.650649695 |
| COL1A1 | 50.85251748 | 9.27E-10 | 0.936791495 |
| TMX4 | 51.00187143 | 8.64E-10 | 1.014810605 |
| PER3 | 51.02262483 | 8.56E-10 | 1.85204216 |
| TCTN3 | 51.05800431 | 8.42E-10 | 1.564707908 |
| ADAM28 | 51.1891001 | 7.91E-10 | 0.779840027 |
| SLC4A7 | 51.46073347 | 6.96E-10 | 0.829928105 |
| ANTXR2 | 51.68696525 | 6.25E-10 | 0.501810157 |
| PPM1N | 51.77057176 | 6.01E-10 | 0.652732783 |
| IRF2 | 51.79122357 | 5.95E-10 | 0.568117034 |
| CISD3 | 51.79939214 | 5.93E-10 | 0.574338394 |
| SELL | 51.79990788 | 5.93E-10 | 0.81582033 |
| MT-ND5 | 51.83039223 | 5.84E-10 | 0.530616755 |
| LILRA5 | 51.8705664 | 5.73E-10 | 1.691006855 |
| AGTRAP | 51.97455977 | 5.46E-10 | 0.912850818 |
| ADAMTSL4 | 52.28400392 | 4.72E-10 | 1.14353772 |
| ARHGEF6 | 52.34054979 | 4.59E-10 | 1.10292759 |
| PLBD2 | 52.53442276 | 4.19E-10 | 0.713052071 |
| PNISR | 52.56318635 | 4.13E-10 | 0.520399844 |
| PARP9 | 52.76124904 | 3.77E-10 | 0.619016612 |
| DUSP3 | 52.93263503 | 3.47E-10 | 1.173747641 |
| ST14 | 52.95762492 | 3.43E-10 | 0.663591897 |
| MYLIP | 52.97553282 | 3.40E-10 | 0.769421773 |
| RNMT | 53.46258543 | 2.70E-10 | 0.855363401 |
| ATP6V1C1 | 53.50314093 | 2.65E-10 | 1.364944463 |
| C19orf33 | 53.85187979 | 2.25E-10 | 1.790817982 |
| BCL6 | 53.87398567 | 2.22E-10 | 1.423160686 |
| SP110 | 53.89989421 | 2.20E-10 | 0.518051982 |
| KDELR2 | 53.9339974 | 2.16E-10 | 1.35937472 |
| PARP14 | 54.07559122 | 2.02E-10 | 0.702581259 |
| CLEC11A | 54.08878856 | 2.01E-10 | 0.720867991 |
| TRMT6 | 54.13455474 | 1.97E-10 | 0.90502563 |
| CD84 | 54.14765207 | 1.95E-10 | 0.696561619 |
| RNF144B | 54.43512376 | 1.71E-10 | 0.786194959 |
| UAP1 | 54.55371141 | 1.61E-10 | 1.589784825 |
| KRT15 | 54.61694343 | 1.57E-10 | 0.905554043 |
| GABARAPL1 | 54.67324461 | 1.52E-10 | 1.2288019 |
| CSGALNACT2 | 54.70043538 | 1.50E-10 | 0.607392299 |
| ITGAM | 54.76528575 | 1.46E-10 | 2.784731772 |
| ST3GAL1 | 54.79404396 | 1.44E-10 | 0.898798984 |
| ZNF106 | 54.80391111 | 1.43E-10 | 0.922145413 |
| RNF145 | 54.82950079 | 1.42E-10 | 1.315532233 |
| IFI6 | 54.87582027 | 1.38E-10 | 1.066573654 |
| CCDC167 | 55.17114528 | 1.20E-10 | 1.654547411 |
| KCNN4 | 55.38387046 | 1.09E-10 | 1.06067192 |
| TTF2 | 55.40755056 | 1.08E-10 | 1.00977284 |
| RALGDS | 55.50765504 | 1.03E-10 | 1.875447535 |
| MYCBP | 55.64613916 | 9.61E-11 | 0.543075187 |
| VAMP5 | 55.65203458 | 9.58E-11 | 0.71224476 |
| FAM107B | 56.13610371 | 7.62E-11 | 0.700616042 |
| JMY | 56.29138231 | 7.08E-11 | 2.357591962 |
| MYL9 | 56.38234556 | 6.78E-11 | 2.948306396 |
| EED | 56.81498559 | 5.52E-11 | 2.00709351 |
| HCAR3 | 56.87269178 | 5.37E-11 | 1.11805955 |
| DYNLT1 | 57.17042689 | 4.66E-11 | 0.681829967 |
| FCER2 | 57.21697061 | 4.56E-11 | 3.54927385 |
| NUP214 | 57.30536455 | 4.37E-11 | 0.959593509 |
| TMEM14A | 57.4275292 | 4.13E-11 | 0.885369102 |
| ADIPOR1 | 57.49796502 | 3.99E-11 | 1.180335692 |
| FCGR1A | 57.51720788 | 3.96E-11 | 2.276439193 |
| ATP2B4 | 57.64171007 | 3.73E-11 | 1.149186621 |
| TCIRG1 | 57.66407147 | 3.69E-11 | 0.554192552 |
| ATP6V1B2 | 57.76702907 | 3.51E-11 | 0.62866376 |
| PTGS1 | 58.22875368 | 2.82E-11 | 0.60796804 |
| HACD4 | 58.5145862 | 2.46E-11 | 1.069494556 |
| AFF3 | 58.60397702 | 2.36E-11 | 2.068778194 |
| CCDC50 | 58.72963296 | 2.22E-11 | 1.020181043 |
| ITPR2 | 58.91821599 | 2.03E-11 | 1.151039275 |
| GZF1 | 59.16484535 | 1.81E-11 | 1.805872388 |
| SIPA1L1 | 59.22895452 | 1.75E-11 | 0.501417468 |
| FLI1 | 59.40511622 | 1.61E-11 | 1.234360573 |
| PARVB | 59.73396248 | 1.38E-11 | 1.442478013 |
| SAV1 | 60.05733312 | 1.18E-11 | 1.660941445 |
| SKAP2 | 60.25846856 | 1.07E-11 | 0.601096447 |
| APOC1 | 60.39948629 | 1.01E-11 | 2.699283723 |
| TNFRSF4 | 60.40284703 | 1.00E-11 | 1.115783031 |
| CCR1 | 60.43277508 | 9.89E-12 | 1.732715285 |
| LCE3D | 60.7625713 | 8.45E-12 | 1.469873524 |
| SEPTIN9 | 60.79652982 | 8.32E-12 | 0.552467851 |
| PTPN18 | 60.80751392 | 8.28E-12 | 1.178844172 |
| NRG1 | 60.82487737 | 8.21E-12 | 1.188882333 |
| GEM | 60.85386894 | 8.10E-12 | 2.114608634 |
| UTRN | 61.05939886 | 7.34E-12 | 1.191006065 |
| KLF7 | 61.10172656 | 7.19E-12 | 1.569633695 |
| FAM49A | 61.11869106 | 7.14E-12 | 0.818300747 |
| VDR | 61.16162286 | 6.99E-12 | 0.697667021 |
| PTAFR | 61.18490476 | 6.91E-12 | 0.968799725 |
| CD69 | 61.18776643 | 6.91E-12 | 0.763080071 |
| LTC4S | 61.21769589 | 6.81E-12 | 0.600707697 |
| ADAM15 | 61.22235089 | 6.79E-12 | 1.155560853 |
| MEF2A | 61.38058171 | 6.30E-12 | 0.752826212 |
| KDM7A | 61.38170885 | 6.30E-12 | 1.51991551 |
| SELPLG | 61.48035991 | 6.01E-12 | 0.745663626 |
| TUBB2B | 61.6308573 | 5.59E-12 | 1.664519385 |
| MYO9B | 61.72649196 | 5.34E-12 | 1.379310227 |
| CREG1 | 61.83419965 | 5.07E-12 | 1.40258146 |
| DMXL2 | 62.08900988 | 4.49E-12 | 1.635606952 |
| TLR1 | 62.20152642 | 4.26E-12 | 0.75393724 |
| BRI3 | 62.2682638 | 4.13E-12 | 1.473869722 |
| AKR1B1 | 62.35202256 | 3.97E-12 | 1.199761219 |
| IKZF1 | 62.46160476 | 3.76E-12 | 1.058443419 |
| CNRIP1 | 62.93725982 | 3.00E-12 | 2.275787588 |
| PALM2-AKAP2 | 63.00042241 | 2.91E-12 | 0.651306446 |
| MAP4 | 63.10600553 | 2.77E-12 | 0.709138625 |
| LDLRAD4 | 63.11068429 | 2.76E-12 | 0.914917589 |
| PIGBOS1 | 63.3939487 | 2.41E-12 | 1.991937392 |
| NUMB | 63.42680683 | 2.37E-12 | 0.926082589 |
| MBP | 63.46807052 | 2.33E-12 | 1.010651697 |
| FAM110A | 63.64923707 | 2.14E-12 | 0.842548543 |
| SPTSSA | 63.74101746 | 2.04E-12 | 0.799204686 |
| CYTH4 | 64.02320078 | 1.79E-12 | 1.212839258 |
| ENC1 | 64.08953922 | 1.73E-12 | 2.033870582 |
| ASGR2 | 64.14546428 | 1.69E-12 | 1.127101184 |
| AGPAT2 | 64.23007904 | 1.62E-12 | 1.365376762 |
| NCOA4 | 64.23704739 | 1.61E-12 | 0.919462309 |
| EFHD2 | 64.50331785 | 1.42E-12 | 1.009400884 |
| HSPA6 | 64.64478939 | 1.33E-12 | 0.823312322 |
| PDXK | 64.71809188 | 1.28E-12 | 0.831532751 |
| YME1L1 | 65.15111052 | 1.04E-12 | 0.791276833 |
| BLCAP | 65.18484235 | 1.03E-12 | 0.622821929 |
| SMAP2 | 65.20917711 | 1.01E-12 | 0.721718002 |
| YTHDF3 | 65.29907014 | 9.72E-13 | 1.570050757 |
| TMEM45A | 65.52856473 | 8.71E-13 | 2.979884451 |
| MMP14 | 65.70247894 | 8.01E-13 | 0.688296158 |
| BAG3 | 65.8591488 | 7.43E-13 | 1.350677302 |
| PRKAR2B | 66.14395279 | 6.49E-13 | 0.676840674 |
| RBM47 | 66.30883425 | 6.00E-13 | 1.497262189 |
| NKRF | 66.31841135 | 5.97E-13 | 1.090205099 |
| GNS | 66.40216863 | 5.73E-13 | 1.236811163 |
| PLCB2 | 66.50568522 | 5.46E-13 | 0.998004153 |
| FGD2 | 66.56938426 | 5.29E-13 | 0.964208013 |
| SLC9A3R1 | 66.95963326 | 4.39E-13 | 1.767399557 |
| ZMIZ2 | 67.02003661 | 4.27E-13 | 0.59109436 |
| THEMIS2 | 67.05769033 | 4.19E-13 | 1.530124673 |
| NUFIP2 | 67.19246035 | 3.93E-13 | 0.94289234 |
| ABHD12 | 67.22826687 | 3.86E-13 | 1.480631325 |
| SULT1A1 | 67.33572655 | 3.67E-13 | 1.195270746 |
| CXorf21 | 67.81901044 | 2.91E-13 | 0.951713803 |
| CYCS | 67.83239562 | 2.89E-13 | 0.605187982 |
| METRNL | 67.87782035 | 2.83E-13 | 1.966631076 |
| HLX | 67.91325123 | 2.78E-13 | 1.098795843 |
| TCEA3 | 67.93903889 | 2.75E-13 | 0.963365638 |
| ARHGAP24 | 68.26999358 | 2.35E-13 | 2.419537544 |
| BAG1 | 68.34631575 | 2.26E-13 | 1.539637603 |
| PDK4 | 68.42777981 | 2.18E-13 | 0.914630901 |
| DDIT3 | 68.73397971 | 1.88E-13 | 1.43461668 |
| RAPGEF1 | 68.89692788 | 1.74E-13 | 0.554216957 |
| PIK3R1 | 68.92188273 | 1.72E-13 | 1.487260673 |
| OXSR1 | 69.06407252 | 1.60E-13 | 1.508446853 |
| BLNK | 69.58628788 | 1.25E-13 | 2.127537135 |
| RUBCNL | 69.71241958 | 1.18E-13 | 1.138189033 |
| SPIB | 70.07538817 | 9.88E-14 | 1.042441853 |
| HEXB | 70.44396199 | 8.28E-14 | 1.421150832 |
| S100A14 | 70.68359205 | 7.38E-14 | 1.268769233 |
| KMO | 70.94099434 | 6.53E-14 | 2.204268584 |
| DUSP5 | 71.25583237 | 5.62E-14 | 0.559401812 |
| MARCO | 71.28821647 | 5.53E-14 | 1.344037634 |
| SAMD9L | 71.44437123 | 5.13E-14 | 0.598156534 |
| TNFAIP8L2 | 71.53578367 | 4.91E-14 | 0.711970383 |
| PARM1 | 71.86143505 | 4.20E-14 | 1.018228287 |
| NIBAN1 | 72.18610681 | 3.60E-14 | 1.365581516 |
| MS4A4A | 72.21054062 | 3.55E-14 | 3.490900084 |
| OTUD1 | 72.24381075 | 3.50E-14 | 1.215926471 |
| BIRC2 | 72.33913476 | 3.34E-14 | 0.919646961 |
| RAPH1 | 72.47765153 | 3.12E-14 | 1.388423472 |
| PLEKHO1 | 72.88768393 | 2.56E-14 | 1.242615734 |
| S100A12 | 72.97651068 | 2.45E-14 | 2.844646999 |
| C3AR1 | 72.98122828 | 2.45E-14 | 3.295371754 |
| FDX1 | 73.01320629 | 2.42E-14 | 2.180077204 |
| PSTPIP1 | 73.14703466 | 2.26E-14 | 1.192970286 |
| CD200R1 | 73.16909292 | 2.24E-14 | 1.685491049 |
| AC244090.1 | 73.25763888 | 2.14E-14 | 0.726484627 |
| DUSP6 | 73.38256497 | 2.02E-14 | 0.797936617 |
| HIVEP1 | 73.41943105 | 1.99E-14 | 1.010111942 |
| U2AF1L4 | 73.57022752 | 1.85E-14 | 1.105544917 |
| FAM50A | 73.92665254 | 1.55E-14 | 1.478989152 |
| RBPJ | 74.0821413 | 1.44E-14 | 0.526213769 |
| PLAU | 74.18283976 | 1.38E-14 | 2.1119385 |
| MRPS33 | 74.28615481 | 1.31E-14 | 0.915679668 |
| HES1 | 74.37102315 | 1.25E-14 | 2.217139596 |
| TUBB3 | 74.46011838 | 1.21E-14 | 0.912960976 |
| LINC02245 | 74.50317776 | 1.18E-14 | 0.630025474 |
| SLC43A3 | 74.68281822 | 1.09E-14 | 0.699868342 |
| CX3CR1 | 74.72954842 | 1.05E-14 | 0.842460696 |
| CD93 | 75.09074471 | 8.88E-15 | 1.018852949 |
| EFCAB14 | 75.23159003 | 8.33E-15 | 0.51579918 |
| NCOR1 | 75.27336517 | 8.10E-15 | 1.135338886 |
| OSM | 75.45823903 | 7.44E-15 | 2.675377307 |
| FAM53C | 76.10701924 | 5.44E-15 | 1.341095288 |
| EMB | 76.42296602 | 4.66E-15 | 0.807397359 |
| FAR1 | 76.44065526 | 4.66E-15 | 0.60167095 |
| MAOA | 76.70075174 | 4.11E-15 | 1.631979664 |
| HSPH1 | 76.79340045 | 3.89E-15 | 0.917144366 |
| KRT17 | 77.00400087 | 3.55E-15 | 2.706758806 |
| ME1 | 77.33757297 | 3.00E-15 | 2.454075817 |
| TAP2 | 77.39277581 | 3.00E-15 | 0.760898513 |
| FNIP2 | 77.41981151 | 2.89E-15 | 0.565447082 |
| TOB1 | 77.57821976 | 2.66E-15 | 1.165955586 |
| ABCA1 | 77.65301788 | 2.55E-15 | 1.493454193 |
| PPA1 | 77.72843598 | 2.55E-15 | 0.851027627 |
| MXD1 | 78.07117598 | 2.11E-15 | 1.478442746 |
| MYADM | 78.2598674 | 1.89E-15 | 0.534425956 |
| GNA13 | 78.42031682 | 1.78E-15 | 1.000477592 |
| EVI2A | 78.64341901 | 1.67E-15 | 0.846806268 |
| ARHGAP6 | 78.6438627 | 1.67E-15 | 2.171959387 |
| ANXA4 | 78.84365711 | 1.44E-15 | 0.730790445 |
| PTX3 | 78.96087198 | 1.33E-15 | 4.327422157 |
| ARL5A | 79.01816612 | 1.33E-15 | 1.48015349 |
| STARD8 | 79.03173401 | 1.33E-15 | 2.100928551 |
| PLD4 | 79.29489291 | 1.22E-15 | 1.756852317 |
| VMO1 | 79.3557946 | 1.11E-15 | 0.697240952 |
| CCR5AS | 79.49866068 | 1.11E-15 | 1.630926206 |
| TBXAS1 | 79.5151735 | 1.11E-15 | 2.034277219 |
| MGST1 | 79.73583739 | 9.99E-16 | 1.440895196 |
| LAMP1 | 79.80377729 | 8.88E-16 | 2.088497155 |
| MATK | 79.83337375 | 8.88E-16 | 1.256719577 |
| IRF2BP2 | 80.19058633 | 7.77E-16 | 1.076946899 |
| SKIL | 80.41974026 | 6.66E-16 | 1.220620892 |
| CRLF2 | 80.99698811 | 5.55E-16 | 1.662214667 |
| IFNGR2 | 81.29034934 | 4.44E-16 | 1.556716044 |
| PLXDC2 | 81.46157173 | 4.44E-16 | 0.907103288 |
| NME1 | 81.59656693 | 4.44E-16 | 0.683895699 |
| ARHGAP4 | 82.07420172 | 3.33E-16 | 1.778158751 |
| RAB30 | 82.16832632 | 3.33E-16 | 0.922534869 |
| ASGR1 | 82.2484428 | 3.33E-16 | 1.115847785 |
| MIR503HG | 82.42195617 | 2.22E-16 | 0.993754326 |
| GPCPD1 | 82.52433804 | 2.22E-16 | 1.528345344 |
| LILRB3 | 82.52722063 | 2.22E-16 | 1.50866897 |
| DNPH1 | 83.00539497 | 2.22E-16 | 1.0043545 |
| NAAA | 83.00581887 | 2.22E-16 | 0.819941389 |
| ATP1B3 | 83.20531374 | 2.22E-16 | 1.102524034 |
| B3GNT5 | 83.32438141 | 2.22E-16 | 0.742669971 |
| LAIR1 | 83.39744925 | 1.11E-16 | 3.795730435 |
| SH3KBP1 | 83.8628164 | 1.11E-16 | 0.907040944 |
| ITSN2 | 83.94254709 | 1.11E-16 | 1.092736093 |
| JMJD6 | 83.96478168 | 1.11E-16 | 1.268655102 |
| NBPF14 | 83.9733889 | 1.11E-16 | 1.811212532 |
| KLF10 | 83.98041483 | 1.11E-16 | 1.014125403 |
| EMILIN2 | 84.0481599 | 1.11E-16 | 0.970639481 |
| SNX9 | 84.07737402 | 1.11E-16 | 1.754535264 |
| MCM5 | 84.62984862 | 1.11E-16 | 0.736597393 |
| NAGK | 84.9124206 | 1.11E-16 | 0.872705764 |
| AC020916.1 | 85.0752987 | 1.11E-16 | 0.569745474 |
| SLAMF9 | 85.17988934 | 1.11E-16 | 1.75064171 |
| NOTCH2 | 85.23904491 | 1.11E-16 | 1.275912174 |
| STAB1 | 85.34785001 | 1.11E-16 | 2.932457778 |
| ABL2 | 85.67059099 | 0 | 1.38954958 |
| METTL9 | 85.86691995 | 0 | 1.643641763 |
| IGFBP7 | 85.87924761 | 0 | 1.818336265 |
| CSF3R | 86.04529812 | 0 | 1.67859278 |
| OAS1 | 86.14216599 | 0 | 2.030785519 |
| NCF1 | 86.57860825 | 0 | 1.410041334 |
| FGD4 | 86.60362201 | 0 | 2.322407036 |
| LILRB4 | 86.76460755 | 0 | 0.775915587 |
| CCL3 | 86.81509982 | 0 | 3.286410742 |
| THBS1 | 87.08158033 | 0 | 3.161360097 |
| CCNH | 87.70001976 | 0 | 0.549615664 |
| NRP1 | 88.03538969 | 0 | 1.08247954 |
| HNRNPU | 88.13917756 | 0 | 0.579467269 |
| LGMN | 88.2049863 | 0 | 1.794949027 |
| WTAP | 88.25829661 | 0 | 1.436517036 |
| PRMT9 | 88.31061901 | 0 | 1.237120891 |
| EGR2 | 88.53911965 | 0 | 1.104290623 |
| ASAH1 | 88.7647984 | 0 | 1.485018607 |
| UPP1 | 88.78652938 | 0 | 1.031870385 |
| SRSF9 | 88.7967637 | 0 | 1.477566422 |
| NAA25 | 88.85252204 | 0 | 0.531993702 |
| H2AFX | 88.99476887 | 0 | 1.70371965 |
| UBD | 89.00119851 | 0 | 2.05131081 |
| GRINA | 89.04999168 | 0 | 1.056788291 |
| SNRNP25 | 89.1709795 | 0 | 0.992192908 |
| NME4 | 89.32107857 | 0 | 1.741573423 |
| HAVCR2 | 89.3675776 | 0 | 0.913217923 |
| APAF1 | 89.42278316 | 0 | 1.421883105 |
| IL10 | 90.14923263 | 0 | 5.120998013 |
| CD300E | 90.44892243 | 0 | 2.452667642 |
| GATM | 90.65407551 | 0 | 1.890674252 |
| PNPLA2 | 90.82655902 | 0 | 1.473243296 |
| MTCH1 | 91.25006439 | 0 | 1.438315536 |
| CTSL | 91.45983148 | 0 | 2.066150154 |
| RTN1 | 92.24017937 | 0 | 1.278112194 |
| TNF | 92.53891172 | 0 | 0.827141424 |
| RALGAPA1 | 92.73429197 | 0 | 1.687971315 |
| FPR1 | 93.0783409 | 0 | 1.011645618 |
| CD300C | 93.12083569 | 0 | 1.306157281 |
| P2RY6 | 93.34546795 | 0 | 1.63693264 |
| SLC7A11 | 93.94786702 | 0 | 2.805831211 |
| MCUR1 | 94.00458576 | 0 | 1.395785 |
| CASP1 | 94.48923354 | 0 | 1.156389904 |
| QSOX1 | 94.84336092 | 0 | 0.512782121 |
| ARF6 | 95.00225019 | 0 | 0.679939881 |
| PLXNC1 | 95.10459286 | 0 | 0.591873498 |
| CMTM3 | 95.20980615 | 0 | 1.210968207 |
| ANXA3 | 95.30199338 | 0 | 7.669802409 |
| GIMAP4 | 95.48880911 | 0 | 1.987214227 |
| IL22RA2 | 95.77845303 | 0 | 82.63140896 |
| CARD9 | 96.48247209 | 0 | 0.556478587 |
| C12orf45 | 96.53964439 | 0 | 1.377576201 |
| PDE4A | 96.77562859 | 0 | 1.399939687 |
| CITED2 | 97.95584034 | 0 | 0.866522376 |
| MAFB | 97.98206828 | 0 | 2.276953719 |
| SLC29A1 | 97.99428017 | 0 | 1.266771275 |
| SPRED2 | 98.08668557 | 0 | 0.954540981 |
| LRP1 | 99.03075069 | 0 | 2.613192121 |
| MFSD12 | 99.33343962 | 0 | 0.612634654 |
| CINP | 99.62811317 | 0 | 0.946009807 |
| SNX10 | 99.9000905 | 0 | 1.130733995 |
| KCTD12 | 100.0315599 | 0 | 1.170195765 |
| VASH1 | 100.1385492 | 0 | 0.99809931 |
| PSD3 | 100.2954257 | 0 | 0.68977087 |
| SDS | 100.529818 | 0 | 4.00925062 |
| ARL4C | 100.622552 | 0 | 0.524473218 |
| TLR4 | 100.8343868 | 0 | 2.955568972 |
| PLIN2 | 100.8611006 | 0 | 1.669203798 |
| MFSD1 | 101.1812464 | 0 | 0.656667253 |
| CLEC2B | 101.3763315 | 0 | 0.864597082 |
| GFRA2 | 101.5728937 | 0 | 2.787915314 |
| MIR4435-2HG | 101.5851839 | 0 | 1.37976346 |
| HPS5 | 101.6687046 | 0 | 0.56356599 |
| PDIA4 | 102.0496782 | 0 | 0.714761569 |
| ADCY7 | 102.0503142 | 0 | 0.668951955 |
| PTPRO | 102.1436231 | 0 | 1.356531077 |
| FYTTD1 | 102.6844952 | 0 | 0.717871101 |
| RAB8B | 102.7211136 | 0 | 0.613337679 |
| LUCAT1 | 102.7965241 | 0 | 3.346912272 |
| NFKBIE | 103.4546344 | 0 | 1.575977336 |
| LYST | 103.9436346 | 0 | 0.676390623 |
| TNFRSF1B | 104.029198 | 0 | 1.507343946 |
| ANKH | 104.2876048 | 0 | 0.739408438 |
| MID1IP1 | 104.8532485 | 0 | 1.37108685 |
| PRNP | 104.9558148 | 0 | 1.39582224 |
| RGL1 | 105.4732311 | 0 | 1.978549297 |
| KRT10 | 106.2853148 | 0 | 1.027307344 |
| ALOX5 | 106.4798273 | 0 | 0.555774599 |
| EPN1 | 106.8023304 | 0 | 2.001379065 |
| TRADD | 106.8728593 | 0 | 0.712713831 |
| IL18BP | 106.8994891 | 0 | 1.038347354 |
| AOAH | 107.2808183 | 0 | 2.894103638 |
| SDC2 | 107.3978419 | 0 | 0.90340298 |
| FOXO3 | 107.8218107 | 0 | 2.270293976 |
| POGLUT1 | 108.1613229 | 0 | 1.201784966 |
| PLD3 | 108.1869275 | 0 | 1.88111751 |
| ENO3 | 108.2577793 | 0 | 3.405899335 |
| IL23A | 108.2929257 | 0 | 3.602815381 |
| SERTAD1 | 108.9171763 | 0 | 0.937342138 |
| SPN | 109.3853941 | 0 | 0.723228335 |
| PLEKHA5 | 109.648229 | 0 | 1.133601228 |
| METTL7A | 109.8547037 | 0 | 1.031387618 |
| NAIP | 110.1819887 | 0 | 1.560199227 |
| SLC7A5 | 110.3705087 | 0 | 2.561352651 |
| PXDC1 | 110.3828207 | 0 | 1.487976628 |
| FKBP4 | 110.5594908 | 0 | 2.432532796 |
| KCNK6 | 110.6505599 | 0 | 0.917354335 |
| NRROS | 110.8130432 | 0 | 1.688113772 |
| ELL2 | 110.8143313 | 0 | 2.406733609 |
| HPGDS | 110.8162226 | 0 | 4.124970521 |
| CD4 | 111.1980493 | 0 | 0.803049585 |
| PLSCR1 | 111.2581253 | 0 | 1.347465832 |
| CMTM7 | 111.3874073 | 0 | 2.05902122 |
| MIDN | 111.5418411 | 0 | 1.279780138 |
| PDLIM4 | 112.2077807 | 0 | 0.795581827 |
| PER1 | 112.3402588 | 0 | 1.562011276 |
| CHD2 | 112.9254658 | 0 | 1.046393426 |
| CCL19 | 113.3471585 | 0 | 2.31866478 |
| SNHG15 | 113.3505971 | 0 | 1.290819244 |
| GAS6 | 113.5964868 | 0 | 1.534093906 |
| PTPN1 | 113.6113546 | 0 | 1.012327659 |
| GGT5 | 114.8510068 | 0 | 1.740731099 |
| SUCNR1 | 115.2628189 | 0 | 1.230896935 |
| TMEM273 | 115.3375689 | 0 | 2.728625402 |
| ATP1A1 | 115.6312226 | 0 | 1.449484472 |
| JMJD1C | 116.1264124 | 0 | 0.949306652 |
| UBL3 | 116.5885124 | 0 | 0.962808657 |
| NIBAN2 | 117.1591759 | 0 | 1.165353284 |
| MGAT1 | 117.1912691 | 0 | 1.247159946 |
| METTL21A | 117.6026174 | 0 | 2.103728863 |
| LPXN | 117.6050327 | 0 | 1.693166078 |
| MAP4K4 | 118.1969398 | 0 | 0.555847402 |
| PDGFB | 118.3676767 | 0 | 2.215093199 |
| CYTOR | 118.4067699 | 0 | 0.906748221 |
| IDO1 | 118.5444334 | 0 | 1.944957457 |
| RBBP8 | 118.9210091 | 0 | 2.065476919 |
| TSPYL2 | 119.5441069 | 0 | 2.446341267 |
| SELENOW | 119.6399362 | 0 | 0.939909033 |
| HBEGF | 120.5652371 | 0 | 2.373044038 |
| LY86 | 120.629108 | 0 | 0.798403945 |
| CPM | 121.2103497 | 0 | 0.76358777 |
| DAB2 | 121.2709612 | 0 | 1.816580235 |
| RPL13 | 122.0967402 | 0 | 0.553789726 |
| EPS8 | 122.1613131 | 0 | 1.715493822 |
| PILRA | 122.6772245 | 0 | 1.172691724 |
| MYO1F | 123.3736869 | 0 | 0.512458661 |
| PPP1R15B | 123.9720434 | 0 | 1.428735048 |
| UBXN11 | 124.0953148 | 0 | 2.117012233 |
| BLVRB | 125.0059669 | 0 | 0.68923914 |
| CST6 | 125.535165 | 0 | 1.64137237 |
| ITGAX | 125.6084462 | 0 | 0.917016146 |
| ATP2B1 | 125.7751196 | 0 | 0.647447174 |
| MCUB | 126.0472188 | 0 | 2.132740258 |
| XIST | 126.2625603 | 0 | 1.412432572 |
| FRMD4B | 126.6109506 | 0 | 1.133070918 |
| GLUD1 | 127.1792421 | 0 | 0.832313554 |
| CCND2 | 127.238808 | 0 | 1.216807191 |
| MARCKSL1 | 127.2604655 | 0 | 0.941157329 |
| NBPF26 | 127.8394335 | 0 | 1.589834337 |
| KRT1 | 127.9595738 | 0 | 1.493159967 |
| CTSD | 128.3898494 | 0 | 1.565784239 |
| CD48 | 130.4647286 | 0 | 1.133619933 |
| 1-Mar | 130.4838798 | 0 | 1.275747726 |
| ARL4A | 130.7159093 | 0 | 1.624417473 |
| RAB5B | 131.5505371 | 0 | 0.574124223 |
| SLC7A7 | 131.9451294 | 0 | 2.339084638 |
| PHLDA1 | 132.4096659 | 0 | 3.490096846 |
| DNAJB6 | 132.7044218 | 0 | 0.874849751 |
| IRF4 | 133.1147926 | 0 | 0.641803955 |
| RAB20 | 133.316737 | 0 | 5.030370567 |
| SERPINF2 | 133.4843565 | 0 | 1.74689328 |
| KIF2A | 133.5426868 | 0 | 1.016675361 |
| SLAMF8 | 134.360727 | 0 | 1.196804008 |
| S100A7 | 134.4061855 | 0 | 2.152587872 |
| COMMD2 | 134.7571345 | 0 | 0.949970636 |
| YWHAH | 135.2184032 | 0 | 0.549942134 |
| TPRA1 | 135.3615451 | 0 | 2.090438753 |
| POU2F2 | 136.0376008 | 0 | 1.838711874 |
| N4BP2L2 | 136.8178743 | 0 | 0.505799808 |
| HDAC9 | 137.8689585 | 0 | 1.900969042 |
| CSF1R | 138.588859 | 0 | 1.133128305 |
| OLR1 | 138.9450846 | 0 | 3.965111961 |
| GADD45A | 139.0105964 | 0 | 0.879132381 |
| EGR1 | 139.5211737 | 0 | 2.082477811 |
| RBM17 | 140.2721282 | 0 | 1.795618944 |
| THBD | 140.6671727 | 0 | 2.598679174 |
| SLC25A19 | 140.7551358 | 0 | 1.833016354 |
| BRD2 | 141.0521985 | 0 | 0.917640934 |
| CALHM6 | 141.2356417 | 0 | 1.862983343 |
| ADA2 | 141.708534 | 0 | 1.674171288 |
| GPAT3 | 142.0299093 | 0 | 1.452786549 |
| GLRX | 142.2984869 | 0 | 0.882666418 |
| CD9 | 142.3360387 | 0 | 0.544197165 |
| CACUL1 | 142.4301882 | 0 | 1.544342198 |
| LILRB2 | 142.7695969 | 0 | 1.853395562 |
| ZMIZ1 | 143.1585558 | 0 | 0.743471171 |
| ABI3 | 143.6347693 | 0 | 1.006707634 |
| RCSD1 | 143.6411932 | 0 | 1.454071793 |
| TOR3A | 143.8459903 | 0 | 1.592303573 |
| TMEM176B | 143.9802504 | 0 | 0.966860921 |
| IL10RA | 144.5088957 | 0 | 1.323615881 |
| PPP1R14A | 144.8819866 | 0 | 1.173763161 |
| GAS7 | 145.2718399 | 0 | 2.34861622 |
| CLECL1 | 145.3621824 | 0 | 1.076895295 |
| PDE4DIP | 145.9664885 | 0 | 1.468099963 |
| RHOF | 146.087172 | 0 | 0.676444059 |
| RNASE1 | 147.1192531 | 0 | 1.568775731 |
| FILIP1L | 147.5226004 | 0 | 0.768568259 |
| PPFIBP1 | 148.1938406 | 0 | 0.849094739 |
| ACTN1 | 149.2228969 | 0 | 0.580250692 |
| DIPK2A | 149.35576 | 0 | 1.884664508 |
| BHLHE40 | 149.4565319 | 0 | 0.512538391 |
| LACTB | 149.5571689 | 0 | 0.975717936 |
| SLC40A1 | 149.6868908 | 0 | 3.623043934 |
| CLEC5A | 150.0208609 | 0 | 1.735269559 |
| LYN | 151.6929616 | 0 | 1.300399554 |
| EHD1 | 152.0652089 | 0 | 2.392376853 |
| LCP2 | 152.8561434 | 0 | 1.091516493 |
| MAP1S | 152.9014905 | 0 | 1.543399943 |
| FOLR2 | 153.1082284 | 0 | 5.455620396 |
| MSR1 | 153.1203896 | 0 | 7.919530772 |
| GPX3 | 153.3109839 | 0 | 0.78445132 |
| ACAP2 | 154.400955 | 0 | 1.526675894 |
| NSUN6 | 154.6530036 | 0 | 2.11998202 |
| INTS3 | 155.2235448 | 0 | 0.700468134 |
| TUBA1A | 156.2310732 | 0 | 0.508658882 |
| APOC2 | 156.3067231 | 0 | 0.999315575 |
| GLUL | 157.3374102 | 0 | 1.085828741 |
| SFN | 157.6102033 | 0 | 1.729779599 |
| APLP2 | 157.623824 | 0 | 0.81335163 |
| RAB10 | 158.5569235 | 0 | 0.525866506 |
| DHRS9 | 158.9106353 | 0 | 1.216186838 |
| ATP6V1G1 | 159.2253686 | 0 | 0.716428707 |
| CCL4 | 160.0236014 | 0 | 3.966375291 |
| MPEG1 | 161.5577977 | 0 | 1.174394585 |
| HSPA1B | 162.5776265 | 0 | 0.519063963 |
| SWAP70 | 162.6801699 | 0 | 0.924401747 |
| CRIP2 | 163.070641 | 0 | 1.237863968 |
| GRASP | 163.2349856 | 0 | 2.107355403 |
| ZYX | 163.2930596 | 0 | 1.075738007 |
| MYO1G | 163.7253211 | 0 | 0.837451745 |
| EMP1 | 164.5042723 | 0 | 0.744060016 |
| JOSD1 | 164.6653203 | 0 | 2.221476653 |
| SLA | 165.1708531 | 0 | 0.970963716 |
| QPRT | 165.6710384 | 0 | 0.852417588 |
| ISG20 | 167.2364064 | 0 | 1.286634945 |
| CDH1 | 167.3863614 | 0 | 1.764537528 |
| TWISTNB | 167.584776 | 0 | 2.778525488 |
| ZFAND5 | 167.6281969 | 0 | 1.773694545 |
| SLC38A1 | 168.9011805 | 0 | 0.737644931 |
| NCF4 | 169.3605871 | 0 | 1.51306598 |
| HLA-DOB | 169.6579846 | 0 | 1.535409135 |
| PLEK2 | 169.6889133 | 0 | 1.797559396 |
| HSD11B1-AS1 | 169.7877225 | 0 | 4.300109013 |
| MAFF | 170.1905547 | 0 | 1.66574543 |
| IVNS1ABP | 171.1050994 | 0 | 1.342102059 |
| MCOLN2 | 171.184815 | 0 | 0.561865298 |
| RASSF5 | 172.0674327 | 0 | 1.875036411 |
| JAK1 | 172.2888547 | 0 | 1.181940264 |
| WSB1 | 173.3502849 | 0 | 0.990333552 |
| TMEM176A | 174.3146384 | 0 | 1.033411494 |
| SYAP1 | 175.2410237 | 0 | 1.298489935 |
| SDF2L1 | 175.6552425 | 0 | 0.894816328 |
| LY6E | 178.3736253 | 0 | 0.531030261 |
| KRT6A | 179.3218581 | 0 | 1.505457024 |
| GLA | 179.5300731 | 0 | 1.790128277 |
| CYTH1 | 181.2240465 | 0 | 0.630453275 |
| ACOT7 | 183.2090046 | 0 | 0.864818903 |
| SERPINA1 | 184.0372545 | 0 | 0.943314435 |
| B4GALT1 | 184.0376259 | 0 | 2.04268577 |
| FBP1 | 186.3915028 | 0 | 1.506564362 |
| ENTPD1 | 186.5457135 | 0 | 0.877767231 |
| ACP5 | 186.7514928 | 0 | 1.107758832 |
| FHOD1 | 188.0089217 | 0 | 1.579905896 |
| SERPINB9 | 188.2897361 | 0 | 1.308306908 |
| KLF3 | 189.2552332 | 0 | 2.690163264 |
| RGS2 | 190.4673808 | 0 | 1.018916354 |
| PHLDA2 | 190.825928 | 0 | 0.6363909 |
| PAPSS2 | 190.8734377 | 0 | 1.383548872 |
| RPS27L | 193.1796577 | 0 | 1.098480728 |
| ALOX5AP | 195.3591845 | 0 | 0.559566426 |
| IL15 | 196.2904257 | 0 | 0.762094523 |
| TGIF1 | 196.5821506 | 0 | 1.360588515 |
| PTPRE | 196.5969968 | 0 | 0.870756041 |
| JAML | 197.2753547 | 0 | 0.999098523 |
| GRAMD1A | 198.3618218 | 0 | 0.937883726 |
| LIMD2 | 199.4437334 | 0 | 0.723581576 |
| POLE4 | 199.6193435 | 0 | 0.694242208 |
| C4orf48 | 199.6277825 | 0 | 2.223876168 |
| PDLIM7 | 200.7103151 | 0 | 1.204545875 |
| CD163 | 201.0196016 | 0 | 4.998171929 |
| CAVIN3 | 201.5173015 | 0 | 0.824828103 |
| EREG | 203.245774 | 0 | 4.601912725 |
| RUFY4 | 204.7113113 | 0 | 1.937938174 |
| ITGA5 | 205.8265916 | 0 | 1.117158467 |
| P3H2 | 206.0419807 | 0 | 1.095563266 |
| RASGEF1B | 206.2108986 | 0 | 1.240548761 |
| IRF8 | 206.803649 | 0 | 2.28678475 |
| ANKRD37 | 208.5030949 | 0 | 2.076298002 |
| HCAR2 | 208.510921 | 0 | 1.234874879 |
| ANP32B | 209.5227924 | 0 | 1.537660573 |
| CHD1 | 210.5459755 | 0 | 1.437731873 |
| SIGLEC10 | 211.7345753 | 0 | 1.137631384 |
| CFD | 212.151248 | 0 | 2.874811389 |
| PID1 | 212.4455954 | 0 | 2.699921647 |
| MS4A7 | 212.6750983 | 0 | 3.01469497 |
| DUSP4 | 214.0375686 | 0 | 1.240574356 |
| LGALS2 | 214.0433435 | 0 | 0.695287655 |
| JPT1 | 216.9840848 | 0 | 0.652110264 |
| MAT2A | 217.4325337 | 0 | 1.594971095 |
| PLAAT3 | 217.4338163 | 0 | 1.119842126 |
| TMEM123 | 219.5255653 | 0 | 1.466552916 |
| TIFAB | 220.6822065 | 0 | 1.772326043 |
| ATP1B1 | 221.4177038 | 0 | 0.847472372 |
| HSPA5 | 221.431327 | 0 | 0.5659481 |
| CDC42EP2 | 221.7656365 | 0 | 3.04337555 |
| ARHGAP18 | 221.9412161 | 0 | 0.944507368 |
| GNG10 | 221.9418156 | 0 | 1.146404706 |
| RNASET2 | 222.1887529 | 0 | 1.670034731 |
| CREB5 | 223.2622655 | 0 | 1.428877959 |
| PLBD1 | 224.4643118 | 0 | 1.633030185 |
| ATP6V0C | 225.6678106 | 0 | 1.189867533 |
| TAGLN2 | 225.8683928 | 0 | 0.514363072 |
| CHMP1B | 227.7824664 | 0 | 1.323916452 |
| DDIT4 | 227.8184686 | 0 | 1.525060738 |
| AREG | 228.0870156 | 0 | 2.067404242 |
| IL1RAP | 230.3944389 | 0 | 0.971162238 |
| CYBB | 231.9889737 | 0 | 2.342037718 |
| AVPI1 | 232.9423295 | 0 | 1.714550055 |
| CISH | 233.9832749 | 0 | 1.811528321 |
| RNASE6 | 234.2255131 | 0 | 1.738321303 |
| AC007384.1 | 235.0918509 | 0 | 1.869155458 |
| NFKB1 | 235.4578313 | 0 | 1.343012773 |
| GPR132 | 236.7072211 | 0 | 1.729558976 |
| MEF2C | 237.0300338 | 0 | 1.21173985 |
| KRT5 | 237.8476044 | 0 | 1.17885101 |
| TGFBI | 238.3876615 | 0 | 0.991703096 |
| EZR | 238.8018352 | 0 | 0.808253865 |
| PRELID1 | 238.8607762 | 0 | 1.871033302 |
| CLPTM1 | 238.9661415 | 0 | 1.258258506 |
| DUSP1 | 239.3076354 | 0 | 0.67697166 |
| DDX3X | 239.6676271 | 0 | 1.089233657 |
| FCGR2A | 240.2805247 | 0 | 2.395527604 |
| HMGN3 | 240.7089927 | 0 | 0.61561909 |
| MMP19 | 243.7684307 | 0 | 3.779226718 |
| FYB1 | 246.6309583 | 0 | 1.160912297 |
| CD36 | 246.7479544 | 0 | 1.674520893 |
| IL1RN | 247.2401346 | 0 | 1.838853189 |
| PPP1R15A | 248.362994 | 0 | 1.128922209 |
| GK | 250.7185081 | 0 | 1.592526522 |
| EGLN3 | 250.9153865 | 0 | 2.031480828 |
| ATOX1 | 251.7741136 | 0 | 0.837805969 |
| SLC43A2 | 252.0729271 | 0 | 1.198108895 |
| SPAG9 | 252.2250665 | 0 | 1.858337309 |
| CCL3L1 | 254.5045479 | 0 | 4.314885614 |
| SH3BP5 | 255.3957046 | 0 | 1.843427579 |
| RIPK2 | 256.8475392 | 0 | 1.62098423 |
| CD14 | 257.2734392 | 0 | 4.121135682 |
| LGALS7B | 257.7229582 | 0 | 1.232680991 |
| SCGB2A2 | 257.7346074 | 0 | 4.552083693 |
| IL13RA1 | 258.9746551 | 0 | 1.161293093 |
| VPS37B | 259.1778745 | 0 | 2.505612974 |
| GNB2 | 260.354175 | 0 | 1.411117321 |
| DBI | 261.1782954 | 0 | 0.576319786 |
| RAMP1 | 265.1373174 | 0 | 1.32125915 |
| MAP3K8 | 266.1277068 | 0 | 1.89632299 |
| VCAN | 266.6436658 | 0 | 1.312116369 |
| DENND1B | 266.82941 | 0 | 0.523945153 |
| CORO1A | 267.8644043 | 0 | 0.685724975 |
| VSIG4 | 268.2220906 | 0 | 3.240042811 |
| KDM2B | 268.3995843 | 0 | 0.697192313 |
| VEGFA | 268.6594995 | 0 | 2.1002762 |
| TUBB | 269.1512697 | 0 | 0.761669357 |
| RILPL2 | 271.0469536 | 0 | 2.162114125 |
| ANPEP | 271.2639782 | 0 | 1.376081813 |
| C1QC | 271.2828137 | 0 | 1.985733328 |
| BTG2 | 271.6723462 | 0 | 0.907475163 |
| SELENOP | 274.0632443 | 0 | 4.298085561 |
| TIPARP | 274.5356586 | 0 | 1.967249478 |
| LST1 | 275.3694441 | 0 | 0.66157423 |
| CCDC85B | 280.0651436 | 0 | 2.520571608 |
| PFKFB3 | 280.782797 | 0 | 2.520351824 |
| PKIB | 281.17454 | 0 | 1.245171156 |
| HCST | 282.9738256 | 0 | 0.88365961 |
| RRM2 | 283.5161097 | 0 | 1.178490111 |
| TLR2 | 285.4163841 | 0 | 2.148601221 |
| CSF2RB | 286.2454744 | 0 | 1.029891643 |
| SAT1 | 292.9781911 | 0 | 1.036756411 |
| BCL3 | 298.9263145 | 0 | 2.484589804 |
| PPP1R10 | 301.0495507 | 0 | 1.813943613 |
| ETV3 | 301.3417656 | 0 | 0.902464534 |
| QPCT | 303.3608235 | 0 | 1.24296941 |
| DAPP1 | 303.6752353 | 0 | 1.006089324 |
| RAC2 | 304.0758755 | 0 | 0.569185872 |
| MMP9 | 304.9018079 | 0 | 3.121931051 |
| PIM3 | 305.7007355 | 0 | 2.084070639 |
| CD302 | 305.8831866 | 0 | 1.882539747 |
| CEBPD | 307.111161 | 0 | 2.677717367 |
| RPS26 | 307.7443876 | 0 | 0.52859805 |
| C1QB | 308.3407479 | 0 | 1.851705837 |
| PHACTR1 | 309.2550856 | 0 | 1.983549074 |
| GPR157 | 309.2879605 | 0 | 1.148204936 |
| CD52 | 310.0471816 | 0 | 0.814206266 |
| TVP23A | 310.6168225 | 0 | 0.9689899 |
| SAMSN1 | 313.7763788 | 0 | 1.099748449 |
| SQSTM1 | 316.2402619 | 0 | 1.881075811 |
| C1QA | 316.5220516 | 0 | 2.553371985 |
| DNASE1L3 | 320.3512346 | 0 | 1.183618154 |
| LSP1 | 320.7229773 | 0 | 0.974327445 |
| PFN1 | 321.9388545 | 0 | 1.204023643 |
| ZEB2 | 322.6136588 | 0 | 0.949949836 |
| ETS2 | 322.8122751 | 0 | 2.439159133 |
| FCGRT | 325.4287806 | 0 | 1.262406331 |
| CD58 | 326.9122222 | 0 | 0.659573233 |
| DOK2 | 330.6391089 | 0 | 0.99111401 |
| CD68 | 331.7356353 | 0 | 1.467572422 |
| PSME2 | 335.9354585 | 0 | 0.798854688 |
| CTSB | 336.2521738 | 0 | 0.993039274 |
| CFP | 336.4422146 | 0 | 0.95746987 |
| LIMS1 | 338.0813034 | 0 | 0.820305292 |
| FPR3 | 338.120378 | 0 | 1.170555409 |
| MRC1 | 338.8503597 | 0 | 2.829873466 |
| TOB2 | 341.9076932 | 0 | 2.138375654 |
| DNMT1 | 343.1725419 | 0 | 0.638715521 |
| SAMHD1 | 343.9717907 | 0 | 0.838639955 |
| PTGS2 | 350.5373485 | 0 | 2.640213808 |
| CTSS | 355.7919686 | 0 | 0.943417134 |
| CEBPB | 356.8314103 | 0 | 3.199918491 |
| EIF4A3 | 359.5766045 | 0 | 2.931741643 |
| EVL | 363.075673 | 0 | 1.852255404 |
| NINJ1 | 364.743136 | 0 | 1.923656123 |
| NPC2 | 367.2702588 | 0 | 0.898070546 |
| TAGAP | 367.6300513 | 0 | 2.650855422 |
| LYPD3 | 368.4728388 | 0 | 0.804090256 |
| FCN1 | 370.1414308 | 0 | 5.040847868 |
| CRTAP | 370.2653813 | 0 | 2.142274827 |
| C1orf162 | 376.6302088 | 0 | 1.169136295 |
| SOCS3 | 378.0214443 | 0 | 1.978306573 |
| HINT1 | 380.5755775 | 0 | 0.916593596 |
| EPSTI1 | 383.8536564 | 0 | 0.846236525 |
| PSAP | 387.513392 | 0 | 1.03568142 |
| GRB2 | 393.3864153 | 0 | 2.063365177 |
| IL1R2 | 395.5765746 | 0 | 1.891538904 |
| CPVL | 397.0342938 | 0 | 1.212870147 |
| HLA-DQA2 | 397.6908864 | 0 | 1.394151242 |
| IGSF6 | 397.7872538 | 0 | 1.343060337 |
| CDC42EP3 | 398.609465 | 0 | 2.349513489 |
| MTHFD2 | 401.8288968 | 0 | 0.537473746 |
| SGK1 | 402.779874 | 0 | 1.107604603 |
| CCL4L2 | 402.9109752 | 0 | 6.937508892 |
| FOSL2 | 403.1739867 | 0 | 2.34960836 |
| ZNF331 | 403.5525472 | 0 | 2.073293081 |
| CCR7 | 407.4490907 | 0 | 2.524475916 |
| AQP3 | 409.259771 | 0 | 1.536957257 |
| RGCC | 411.5671739 | 0 | 3.612578785 |
| F13A1 | 415.483998 | 0 | 1.838891083 |
| MAP1LC3B | 420.0311158 | 0 | 2.169047836 |
| PYCARD | 421.5356711 | 0 | 0.877753937 |
| XBP1 | 423.5311398 | 0 | 2.428419756 |
| RAB7B | 426.8020733 | 0 | 0.818915447 |
| FCGR2B | 428.9365554 | 0 | 1.86759504 |
| LAPTM5 | 430.6274688 | 0 | 0.821077897 |
| RNF130 | 431.1271877 | 0 | 2.323361206 |
| PDE4B | 435.0471112 | 0 | 1.809191101 |
| SERP1 | 436.1719149 | 0 | 1.872551733 |
| CHCHD10 | 437.5096587 | 0 | 3.02843783 |
| CREM | 440.342583 | 0 | 2.081745455 |
| CD1C | 442.419737 | 0 | 0.918419839 |
| MT-ND3 | 444.7077396 | 0 | 0.656215896 |
| CERS6 | 448.4890119 | 0 | 1.008692695 |
| HERPUD1 | 448.9936159 | 0 | 2.198717647 |
| REL | 455.5979914 | 0 | 1.318659393 |
| KLF4 | 458.146829 | 0 | 2.30008573 |
| AP1S2 | 459.6606407 | 0 | 1.57015171 |
| LAMP3 | 464.8632407 | 0 | 2.595034889 |
| CD40 | 470.3867361 | 0 | 1.011573346 |
| TRAF1 | 474.7404253 | 0 | 1.470960482 |
| MAP2K3 | 477.1489104 | 0 | 3.00585937 |
| FSCN1 | 478.4243725 | 0 | 1.353581053 |
| ATP5F1D | 488.7856451 | 0 | 2.380059041 |
| DCAF6 | 493.1019047 | 0 | 0.594647091 |
| EMP3 | 495.4477103 | 0 | 0.504796908 |
| CLEC4A | 497.6583939 | 0 | 1.1066985 |
| PPT1 | 498.5588067 | 0 | 0.908253833 |
| CALR | 499.424097 | 0 | 0.589126622 |
| NDRG2 | 512.1236812 | 0 | 1.482370286 |
| GAPDH | 517.951492 | 0 | 0.569104593 |
| CKLF | 522.2204862 | 0 | 1.270685885 |
| PPIF | 523.6562051 | 0 | 3.005588681 |
| ARL5B | 523.9342325 | 0 | 2.992696529 |
| DNAJA1 | 525.2088523 | 0 | 1.445420326 |
| ZFP36L2 | 528.8998616 | 0 | 2.118616774 |
| SOAT1 | 534.3979505 | 0 | 1.032334557 |
| FOS | 534.5528041 | 0 | 0.747263138 |
| SOD2 | 537.6842955 | 0 | 2.850259682 |
| FGL2 | 543.5997494 | 0 | 1.089350071 |
| LCP1 | 547.5287395 | 0 | 0.55288286 |
| NAMPT | 556.1808453 | 0 | 1.625908953 |
| KDM6B | 559.3042763 | 0 | 2.098058406 |
| TACSTD2 | 567.2982783 | 0 | 2.823250666 |
| TSPO | 569.8466487 | 0 | 0.500326289 |
| MNDA | 579.2375148 | 0 | 0.836766557 |
| IRF1 | 582.1469004 | 0 | 1.168726164 |
| HLA-DMA | 590.5782527 | 0 | 0.625850358 |
| MCL1 | 599.4200749 | 0 | 1.726969301 |
| CSRNP1 | 602.8967526 | 0 | 2.227865159 |
| NR4A3 | 604.2808239 | 0 | 1.941146743 |
| FTL | 612.3279036 | 0 | 1.177147544 |
| NUB1 | 613.3185424 | 0 | 1.494881723 |
| ZC3H12A | 626.508118 | 0 | 2.265363699 |
| CD63 | 634.8816607 | 0 | 0.568166932 |
| GNG5 | 636.1341147 | 0 | 0.744357406 |
| CD55 | 645.5491922 | 0 | 2.430620101 |
| MTSS1 | 646.7836166 | 0 | 0.812020188 |
| STX11 | 648.3129705 | 0 | 2.58485094 |
| MS4A6A | 659.396807 | 0 | 1.855387185 |
| CKS2 | 660.2669032 | 0 | 1.761990563 |
| TMSB10 | 662.274312 | 0 | 0.523311637 |
| ITM2B | 667.222956 | 0 | 2.006849466 |
| PLEK | 677.2330964 | 0 | 1.389706726 |
| NFKBID | 677.3927513 | 0 | 1.889395859 |
| TIMP1 | 685.3602956 | 0 | 1.443073019 |
| ZFP36 | 686.3501944 | 0 | 1.053376768 |
| SLC27A3 | 700.8577528 | 0 | 1.986149677 |
| CD1E | 701.3952538 | 0 | 2.435979139 |
| CD207 | 703.4236392 | 0 | 8.902123805 |
| COTL1 | 707.2196632 | 0 | 1.675664769 |
| FCGBP | 707.526259 | 0 | 2.466066327 |
| MIR155HG | 721.0334592 | 0 | 1.833136136 |
| LDHA | 732.6399721 | 0 | 0.84213952 |
| HLA-DPB1 | 735.0878665 | 0 | 0.574116828 |
| CXCL8 | 737.3119936 | 0 | 3.161693385 |
| ACTG1 | 742.9633794 | 0 | 0.938516852 |
| SLC2A3 | 750.2441479 | 0 | 1.357888981 |
| GPR183 | 763.4178042 | 0 | 1.602609348 |
| ATF3 | 763.6855576 | 0 | 2.648248522 |
| PDCD1LG2 | 771.9537412 | 0 | 2.223052913 |
| NLRP3 | 786.6108276 | 0 | 4.529251598 |
| ANXA1 | 793.4380243 | 0 | 0.804853905 |
| NR4A1 | 802.1168526 | 0 | 1.819490763 |
| IER3 | 809.018408 | 0 | 2.316049426 |
| ICAM1 | 811.1512179 | 0 | 2.052775112 |
| CXCL2 | 813.5871862 | 0 | 5.991695674 |
| HLA-DRB5 | 817.6894168 | 0 | 1.031941395 |
| ALOX15 | 821.4916426 | 0 | 1.497765331 |
| TNFAIP3 | 835.1491039 | 0 | 2.227486105 |
| NFKB2 | 839.2718957 | 0 | 0.724806105 |
| CRIP1 | 839.5350987 | 0 | 0.856622533 |
| NFE2L2 | 840.4346393 | 0 | 2.146166999 |
| ACTB | 841.398441 | 0 | 0.604263357 |
| CD1B | 842.4637437 | 0 | 3.487570736 |
| SELENOK | 846.2929156 | 0 | 1.557907053 |
| CXCL3 | 950.9957807 | 0 | 5.455266024 |
| NR4A2 | 961.8025678 | 0 | 2.313509972 |
| AIF1 | 987.367076 | 0 | 1.384345815 |
| DUSP2 | 989.5727775 | 0 | 3.119661474 |
| CALM1 | 995.455837 | 0 | 0.521810569 |
| S100B | 1029.531786 | 0 | 2.747845009 |
| BTG1 | 1034.478768 | 0 | 1.409237342 |
| SRGN | 1054.491082 | 0 | 1.07789432 |
| RPS2 | 1092.836806 | 0 | 1.572240278 |
| SEC61B | 1094.00921 | 0 | 1.343059023 |
| INSIG1 | 1111.034884 | 0 | 3.384191122 |
| FCER1A | 1115.78534 | 0 | 1.215689276 |
| CD83 | 1127.781253 | 0 | 1.015960557 |
| BCL2A1 | 1157.878692 | 0 | 2.688870171 |
| S100A11 | 1185.619051 | 0 | 0.592742171 |
| HLA-DQB2 | 1205.143284 | 0 | 1.438737724 |
| PLAUR | 1267.490152 | 0 | 2.414240102 |
| RHOB | 1269.217661 | 0 | 1.298114227 |
| FCER1G | 1291.462617 | 0 | 1.085117781 |
| LYZ | 1366.888979 | 0 | 0.877531827 |
| FOSB | 1370.710835 | 0 | 2.344227209 |
| S100A10 | 1419.102557 | 0 | 0.848500589 |
| GSN | 1439.048586 | 0 | 1.058815414 |
| CLEC10A | 1444.424056 | 0 | 1.048162937 |
| NFKBIZ | 1496.676545 | 0 | 3.322676088 |
| CD37 | 1557.544468 | 0 | 0.670532198 |
| S100A4 | 1597.868425 | 0 | 0.836191203 |
| C15orf48 | 1602.613736 | 0 | 1.321497906 |
| FTH1 | 1651.213892 | 0 | 2.452957001 |
| HLA-DRB1 | 1820.685588 | 0 | 0.591494063 |
| HLA-DQB1 | 1831.019182 | 0 | 0.956113898 |
| SH3BGRL3 | 2085.311154 | 0 | 0.75659519 |
| IL1B | 2121.540494 | 0 | 4.675529109 |
| CD1A | 2126.183806 | 0 | 2.919571312 |
| CCL22 | 2324.820074 | 0 | 2.370071563 |
| NFKBIA | 2339.432941 | 0 | 3.455217128 |
| BASP1 | 2880.168655 | 0 | 0.674856739 |
| TXN | 2883.614827 | 0 | 1.948814614 |
| HLA-DQA1 | 3539.10469 | 0 | 0.692319704 |
| RAB13 | 3663.808416 | 0 | 0.635860627 |
| HLA-DRA | 4388.597042 | 0 | 0.583880819 |
| RAB29 | 5592.225896 | 0 | 1.383641365 |
| PNRC1 | 8584.826991 | 0 | 0.981356629 |
| SLC16A3 | 12203.20245 | 0 | 0.854628143 |
| HLA-DPA1 | 21874.03367 | 0 | 0.618973676 |

| **Table S6. Highly Variable Genes Between Trajectory Start and End Points** | | | |
| --- | --- | --- | --- |
| **gene** | **waldStat** | **pvalue** | **logFC** |
| MATK | 10.02304503 | 0.001545937 | -4.935610519 |
| DNMT1 | 10.02420397 | 0.001544964 | -1.332776258 |
| ZSWIM6 | 10.02460357 | 0.001544629 | -1.36944511 |
| GPR34 | 10.0264179 | 0.001543108 | -3.967301619 |
| SPINK5 | 10.04062821 | 0.00153125 | 2.815739288 |
| CST6 | 10.05017804 | 0.001523332 | -5.94271007 |
| OPTN | 10.05402537 | 0.001520154 | 1.234437211 |
| SEL1L | 10.10141127 | 0.001481558 | -1.150079406 |
| COL6A1 | 10.12019545 | 0.001466534 | 2.272040815 |
| ANKH | 10.12339487 | 0.001463991 | -2.49952504 |
| CAV1 | 10.16048091 | 0.001434832 | 1.127383279 |
| GPR171 | 10.16986996 | 0.001427543 | 1.939468256 |
| PHF14 | 10.24018016 | 0.001374142 | -1.302295959 |
| APOE | 10.26816427 | 0.001353454 | -1.820172739 |
| WDR45B | 10.3228194 | 0.001313954 | -1.330846766 |
| GUSB | 10.35988438 | 0.001287833 | -1.241151994 |
| CCDC170 | 10.38488351 | 0.001270512 | -4.483159319 |
| QSOX1 | 10.40980685 | 0.001253479 | -1.522136605 |
| CHCHD1 | 10.49255467 | 0.001198565 | 1.172854704 |
| LBR | 10.50171553 | 0.001192638 | -1.45028999 |
| SBSN | 10.52694731 | 0.001176464 | 1.612147683 |
| DSG3 | 10.57979439 | 0.001143304 | 3.249906434 |
| KIAA0100 | 10.59300352 | 0.001135164 | -1.64391738 |
| FKBP15 | 10.61611878 | 0.001121061 | -1.841463711 |
| ENG | 10.64156291 | 0.001105742 | -1.388900262 |
| FLT3 | 10.65690715 | 0.001096606 | 1.321153548 |
| TMEM273 | 10.69460418 | 0.001074485 | -9.990321381 |
| HEXIM1 | 10.69745615 | 0.001072829 | -1.176309994 |
| PRKCB | 10.70386841 | 0.001069118 | -1.860128365 |
| TMEM60 | 10.71026387 | 0.001065428 | 1.547290621 |
| USP48 | 10.71206872 | 0.00106439 | -1.010730778 |
| WWP1 | 10.71933635 | 0.001060217 | -1.719526201 |
| SATB1 | 10.81322223 | 0.001007777 | -3.507249935 |
| SCLT1 | 10.81562795 | 0.001006469 | -1.598395541 |
| SDC2 | 10.84669974 | 0.00098972 | -2.012014384 |
| ID3 | 10.988362 | 0.000916858 | -1.000102107 |
| CD300C | 11.03640016 | 0.000893402 | -4.193960419 |
| MAPK6 | 11.05412406 | 0.000884901 | -1.475267914 |
| OPN3 | 11.07062714 | 0.00087706 | -1.764405812 |
| HTT | 11.10297713 | 0.000861892 | -1.570870288 |
| DCT | 11.1301013 | 0.00084938 | -5.300296917 |
| AGPAT2 | 11.165046 | 0.000833529 | -2.197609023 |
| MYO1F | 11.18913445 | 0.000822777 | -1.26659825 |
| HAVCR2 | 11.19586006 | 0.0008198 | -1.828542292 |
| TLR1 | 11.21896998 | 0.000809654 | -2.79556727 |
| MAN1A2 | 11.25270715 | 0.00079507 | -1.761350675 |
| IRF4 | 11.27020803 | 0.000787609 | 1.030950996 |
| DHRS9 | 11.27419953 | 0.000785917 | -3.68174247 |
| ARHGAP25 | 11.29376933 | 0.000777676 | -1.962657082 |
| ETNK1 | 11.3056291 | 0.000772725 | -1.388826025 |
| RYBP | 11.32692579 | 0.000763913 | -1.120817101 |
| CCNT2 | 11.33538105 | 0.000760442 | 1.058393752 |
| COL6A6 | 11.37500301 | 0.000744391 | 2.851032045 |
| KRT2 | 11.43933855 | 0.000719054 | 1.77314085 |
| CTSA | 11.51586967 | 0.000690045 | -1.275358906 |
| BEX3 | 11.53150657 | 0.000684265 | 1.298392708 |
| LRIF1 | 11.56645052 | 0.000671525 | -1.091768674 |
| MILR1 | 11.5709573 | 0.000669899 | -2.331509943 |
| GADD45A | 11.58581368 | 0.000664568 | 1.242054236 |
| ADAMTSL4 | 11.58979064 | 0.000663149 | 1.837800891 |
| CALCRL | 11.62552288 | 0.00065053 | 1.363185739 |
| COMP | 11.63008871 | 0.000648935 | 2.937021726 |
| IL1R1 | 11.64631163 | 0.0006433 | -1.580438994 |
| CCR1 | 11.66322629 | 0.000637477 | -6.019267887 |
| CERS2 | 11.66985764 | 0.000635209 | -1.667566248 |
| HEATR5B | 11.67867038 | 0.000632207 | -2.183411793 |
| C3AR1 | 11.73374364 | 0.00061377 | -6.955144387 |
| SCGB2A2 | 11.7378022 | 0.000612433 | -3.571478124 |
| GOLIM4 | 11.75700972 | 0.000606145 | -1.642759167 |
| LACTB | 11.85921373 | 0.000573768 | -1.180763601 |
| CD300E | 11.87131046 | 0.000570053 | -6.059455274 |
| DNM2 | 11.88229191 | 0.000566702 | -1.238835574 |
| APCDD1 | 11.88481301 | 0.000565935 | 1.671097868 |
| CNRIP1 | 11.90329729 | 0.000560347 | -2.323875968 |
| FABP4 | 11.90663229 | 0.000559344 | -3.168278138 |
| P2RY6 | 11.9101491 | 0.000558289 | -6.892190328 |
| FAM126A | 11.9158272 | 0.00055659 | -1.121221007 |
| LILRA5 | 11.92673067 | 0.000553342 | -4.083459794 |
| C12orf75 | 11.92827002 | 0.000552885 | -1.346122885 |
| EIF4EBP2 | 11.92965978 | 0.000552473 | -1.00793947 |
| ATP2A2 | 11.93563637 | 0.000550703 | -1.390851669 |
| PLBD2 | 11.93565831 | 0.000550697 | -2.061766363 |
| STAB1 | 11.97842161 | 0.000538202 | -7.585749736 |
| ARPIN | 11.97991711 | 0.00053777 | -1.375977961 |
| AC090559.1 | 12.0465968 | 0.00051887 | -1.642640321 |
| CCNB1 | 12.07286238 | 0.000511611 | 1.853241406 |
| UBE2E3 | 12.10399316 | 0.00050314 | -1.410957482 |
| LTC4S | 12.11916084 | 0.000499064 | -2.127997805 |
| SH3PXD2A | 12.12265159 | 0.00049813 | 1.943971918 |
| HRH2 | 12.17184413 | 0.000485163 | -5.526175172 |
| CAVIN2 | 12.21020806 | 0.000475287 | -4.464519923 |
| PNPLA2 | 12.22331979 | 0.000471959 | -1.471339101 |
| XAF1 | 12.25421897 | 0.000464207 | -2.067203354 |
| MDM4 | 12.2552811 | 0.000463943 | -1.307407371 |
| SFXN3 | 12.25691134 | 0.000463538 | -1.513309398 |
| TBC1D14 | 12.26123552 | 0.000462465 | -1.576616728 |
| HSPA6 | 12.35215653 | 0.000440477 | -1.847581481 |
| EIF4EBP3 | 12.49747425 | 0.000407503 | -1.261211202 |
| IDI1 | 12.51246229 | 0.000404246 | -1.210164849 |
| ACP2 | 12.51818461 | 0.00040301 | 1.334670888 |
| IL10 | 12.52209473 | 0.000402168 | -14.40261966 |
| PID1 | 12.53181928 | 0.00040008 | -5.264793282 |
| CUL4A | 12.53219216 | 0.0004 | -1.373135083 |
| SPRED1 | 12.54345676 | 0.000397596 | -2.290551944 |
| SLAMF1 | 12.61627647 | 0.000382402 | 1.90210346 |
| RB1CC1 | 12.6494674 | 0.000375673 | -1.198253585 |
| PTAFR | 12.65160888 | 0.000375243 | -2.625023904 |
| MT1M | 12.65587804 | 0.000374387 | 1.98772969 |
| DSC3 | 12.68085112 | 0.000369419 | 1.59409662 |
| ADAP2 | 12.69891039 | 0.000365869 | -2.084616145 |
| CDCP1 | 12.70959625 | 0.000363784 | -1.046957981 |
| P2RY14 | 12.751089 | 0.000355802 | -1.35129616 |
| CCL20 | 12.7678026 | 0.000352637 | -5.877057337 |
| DCAF12 | 12.7723354 | 0.000351783 | -2.048228422 |
| ZFYVE16 | 12.93560642 | 0.000322391 | -1.559929958 |
| OSGEP | 12.9569117 | 0.000318743 | -1.5095748 |
| EEA1 | 13.01056092 | 0.000309739 | -1.241778859 |
| TTYH3 | 13.0305466 | 0.000306451 | -3.036652271 |
| MAP3K20 | 13.0885455 | 0.000297107 | -1.328408936 |
| NUMB | 13.14826439 | 0.000287786 | -1.146172378 |
| MX1 | 13.1742241 | 0.000283826 | -1.807807101 |
| FAM91A1 | 13.22625918 | 0.000276054 | -1.202987221 |
| APOC1 | 13.23622814 | 0.00027459 | -1.855366394 |
| NSD3 | 13.2796404 | 0.000268304 | -1.3232492 |
| AP3D1 | 13.28880952 | 0.000266995 | -1.829285667 |
| ARIH1 | 13.29954358 | 0.000265471 | -1.460939045 |
| BATF | 13.31406606 | 0.000263423 | 1.84761004 |
| RABGAP1L | 13.37759562 | 0.000254648 | 1.085864294 |
| SLC8B1 | 13.38312035 | 0.000253899 | -1.55928221 |
| LRRK2 | 13.39768694 | 0.000251935 | -4.012213914 |
| STAT2 | 13.40238901 | 0.000251304 | -1.151855688 |
| MIR205HG | 13.43031639 | 0.00024759 | 2.107470609 |
| LRMP | 13.44769843 | 0.000245307 | -2.415165786 |
| GPR65 | 13.45570991 | 0.000244262 | -2.254165821 |
| BEST1 | 13.48377971 | 0.000240635 | -1.568075849 |
| LCE3D | 13.53778453 | 0.000233808 | 2.694659561 |
| AOC1 | 13.56187217 | 0.000230827 | 6.340986779 |
| ARHGAP22 | 13.5647196 | 0.000230477 | 1.601377105 |
| POSTN | 13.57932993 | 0.00022869 | 2.063496188 |
| DDB2 | 13.65823852 | 0.000219277 | 2.181146945 |
| GJA1 | 13.66026049 | 0.000219041 | 2.613511476 |
| VSIG4 | 13.70039931 | 0.000214409 | -4.623200875 |
| ARAP1 | 13.70574018 | 0.0002138 | -2.58719906 |
| ATP2B1-AS1 | 13.73031815 | 0.00021102 | -1.005443067 |
| XIST | 13.74294302 | 0.000209607 | -2.421097513 |
| EIF2AK2 | 13.74457127 | 0.000209425 | -1.158086105 |
| ZBTB7A | 13.7740002 | 0.00020617 | -1.695910621 |
| FTX | 13.78505344 | 0.00020496 | 1.095134019 |
| TYRP1 | 13.7877725 | 0.000204664 | -3.087407005 |
| LILRB1 | 13.80029006 | 0.000203305 | -5.107153267 |
| PAM16 | 13.90372425 | 0.000192417 | 1.426535773 |
| GPNMB | 13.92688876 | 0.00019006 | -2.203708996 |
| MITF | 13.93115651 | 0.000189629 | -3.453758436 |
| NSMCE1 | 13.95763724 | 0.000186977 | 1.235776481 |
| SLC46A3 | 13.97917264 | 0.000184847 | -3.23460017 |
| CDV3 | 13.98280147 | 0.000184491 | -1.342550118 |
| SS18 | 14.02000867 | 0.000180876 | -1.779220928 |
| RIPOR2 | 14.02029049 | 0.000180849 | -1.928487929 |
| NUP58 | 14.03648632 | 0.000179298 | -1.342405284 |
| TREM1 | 14.06261868 | 0.000176823 | -9.906377317 |
| ENC1 | 14.11770666 | 0.00017172 | -10.20639476 |
| CCL3L1 | 14.13368178 | 0.000170268 | -8.269647138 |
| CREG1 | 14.1366716 | 0.000169997 | -1.296891594 |
| CRIP2 | 14.19792304 | 0.000164552 | 1.205523535 |
| CYTOR | 14.23149268 | 0.000161643 | -2.00198442 |
| AOAH | 14.29175104 | 0.000156549 | -3.718681136 |
| PER3 | 14.31042963 | 0.000155004 | -2.120951569 |
| REV3L | 14.38176671 | 0.00014924 | -1.349639197 |
| ATP6V0A1 | 14.40517535 | 0.000147397 | -2.692714743 |
| NUDT3 | 14.49567347 | 0.000140482 | -1.637426373 |
| GRK2 | 14.49855529 | 0.000140267 | -1.401751555 |
| ENO3 | 14.51177378 | 0.000139286 | 6.244687609 |
| GNL3 | 14.51626977 | 0.000138954 | -1.259892433 |
| CRLF2 | 14.53815847 | 0.000137349 | 4.493038665 |
| GOLGA4 | 14.62650704 | 0.000131058 | -1.073004537 |
| HDAC9 | 14.64900864 | 0.000129503 | -4.478968584 |
| SNAI1 | 14.70102876 | 0.000125978 | -3.82247669 |
| IL1RAP | 14.73708184 | 0.000123591 | -3.170360731 |
| PAG1 | 14.84541338 | 0.000116691 | -2.004070354 |
| LY6E | 14.85753522 | 0.000115944 | -1.122091365 |
| PLD4 | 14.90930321 | 0.000112805 | -6.112955469 |
| CALML5 | 14.95545841 | 0.000110079 | 2.032795979 |
| OXR1 | 14.96273364 | 0.000109656 | -1.707738429 |
| IQCG | 14.99142479 | 0.000108001 | 1.934047253 |
| SPRR2E | 15.02000521 | 0.000106378 | 2.967443694 |
| ARAP2 | 15.02302773 | 0.000106207 | 1.219272285 |
| LY6D | 15.07703476 | 0.000103211 | 1.358000699 |
| YWHAH | 15.08313059 | 0.000102879 | -1.454215732 |
| WIPI2 | 15.1582298 | 9.89E-05 | -1.392069294 |
| CLN8 | 15.18999229 | 9.72E-05 | -2.061021924 |
| ADAM19 | 15.20301228 | 9.65E-05 | 3.303948425 |
| FXYD3 | 15.21559781 | 9.59E-05 | 2.121072507 |
| PRDM1 | 15.29786558 | 9.18E-05 | -1.228727489 |
| NAAA | 15.34242321 | 8.97E-05 | -1.876835097 |
| RERE | 15.34374278 | 8.96E-05 | -1.280527914 |
| STAG2 | 15.38449576 | 8.77E-05 | -1.144459642 |
| IKZF1 | 15.38853905 | 8.75E-05 | -1.057019894 |
| MPP1 | 15.41700106 | 8.62E-05 | -2.286535668 |
| WAPL | 15.4302707 | 8.56E-05 | -1.360657392 |
| SAFB | 15.50140227 | 8.24E-05 | -1.234447647 |
| FBN1 | 15.51680926 | 8.18E-05 | 2.323510253 |
| FYTTD1 | 15.54086927 | 8.07E-05 | 1.199998131 |
| PTTG1 | 15.55954845 | 7.99E-05 | -1.45683954 |
| ABHD2 | 15.58020639 | 7.91E-05 | -2.391221821 |
| CYP1B1 | 15.61389175 | 7.77E-05 | -4.523557268 |
| CYSTM1 | 15.65716448 | 7.59E-05 | 1.222016197 |
| TMEM106A | 15.68218931 | 7.49E-05 | -4.479100532 |
| RABGAP1 | 15.73322277 | 7.29E-05 | 1.246589205 |
| TSC22D2 | 15.73872665 | 7.27E-05 | -1.423348666 |
| PSD3 | 15.74855674 | 7.23E-05 | 2.188913041 |
| PPP1R21 | 15.75160198 | 7.22E-05 | 1.069336278 |
| TNC | 15.81105765 | 7.00E-05 | 2.579614295 |
| SIGLEC1 | 15.82158259 | 6.96E-05 | -3.550333201 |
| RUFY3 | 15.82665461 | 6.94E-05 | 1.501798463 |
| RNF145 | 15.83004308 | 6.93E-05 | -1.503843527 |
| TMX4 | 15.88935074 | 6.72E-05 | -1.989963155 |
| CTTNBP2 | 15.90049387 | 6.68E-05 | -3.483873745 |
| ALOX5 | 15.90779745 | 6.65E-05 | -1.785762944 |
| KRT6C | 15.91252566 | 6.63E-05 | 2.118405488 |
| MALT1 | 15.96567657 | 6.45E-05 | -1.508556141 |
| VEZT | 15.97498938 | 6.42E-05 | 1.268042503 |
| CSGALNACT2 | 16.00111712 | 6.33E-05 | -2.155664864 |
| KPNA2 | 16.0203519 | 6.27E-05 | -1.213271832 |
| DNAJA4 | 16.03557089 | 6.22E-05 | -3.092637816 |
| MYO9B | 16.03837968 | 6.21E-05 | -1.256838377 |
| RHOQ | 16.04170817 | 6.20E-05 | -1.829100222 |
| ENTPD1 | 16.05993868 | 6.14E-05 | -1.811628805 |
| LMNB1 | 16.08224746 | 6.06E-05 | 1.065102642 |
| STK38 | 16.0975832 | 6.02E-05 | -1.384605732 |
| DSG1 | 16.11335008 | 5.97E-05 | 1.646258227 |
| CD40 | 16.15319376 | 5.84E-05 | 1.178109005 |
| HCAR3 | 16.15324093 | 5.84E-05 | -4.342835628 |
| IQSEC1 | 16.15536357 | 5.84E-05 | -2.27755296 |
| FPR1 | 16.22291253 | 5.63E-05 | -3.691792004 |
| TNFRSF11B | 16.24001682 | 5.58E-05 | 8.349973161 |
| FGD2 | 16.27363577 | 5.48E-05 | 1.461420951 |
| TENT5A | 16.27521138 | 5.48E-05 | -1.508968795 |
| FES | 16.29583412 | 5.42E-05 | -2.431091725 |
| NLRP3 | 16.3981993 | 5.13E-05 | -9.531084327 |
| CCDC6 | 16.40441405 | 5.12E-05 | -1.871576654 |
| MAF | 16.42271851 | 5.07E-05 | -2.472641259 |
| PRMT9 | 16.45375043 | 4.99E-05 | -2.174563691 |
| TLR4 | 16.52105215 | 4.81E-05 | -5.085751012 |
| SERPINB5 | 16.52515427 | 4.80E-05 | 2.490335534 |
| DGKZ | 16.53206464 | 4.78E-05 | -1.527757542 |
| SH3TC1 | 16.53296888 | 4.78E-05 | -2.694643167 |
| SLC4A7 | 16.57263324 | 4.68E-05 | -1.533684088 |
| ARFGAP3 | 16.59909587 | 4.62E-05 | -1.473367115 |
| MAGOHB | 16.62357684 | 4.56E-05 | 1.634572014 |
| ZNF22 | 16.63524055 | 4.53E-05 | 1.142629999 |
| SIRPA | 16.69727544 | 4.38E-05 | -1.232179249 |
| FILIP1L | 16.71059335 | 4.35E-05 | -1.630522339 |
| VMO1 | 16.71403054 | 4.35E-05 | -2.250450961 |
| IL23A | 16.72044489 | 4.33E-05 | -5.296228789 |
| AAK1 | 16.75148749 | 4.26E-05 | -1.874108187 |
| NAPRT | 16.96573825 | 3.81E-05 | -1.746880402 |
| POLE4 | 16.98686575 | 3.76E-05 | -1.910894692 |
| SUSD6 | 16.99053945 | 3.76E-05 | -2.148070467 |
| ASPH | 17.07302828 | 3.60E-05 | -1.913007239 |
| KCNQ1OT1 | 17.09528605 | 3.56E-05 | -2.271171362 |
| SERPINB3 | 17.1428465 | 3.47E-05 | 3.345779849 |
| PIP4P2 | 17.14927061 | 3.46E-05 | -3.618840707 |
| PERP | 17.15647154 | 3.44E-05 | 1.405670808 |
| MARCO | 17.20711505 | 3.35E-05 | -3.583008152 |
| ZEB1 | 17.36764281 | 3.08E-05 | 1.947920817 |
| JMY | 17.37478641 | 3.07E-05 | -2.639483499 |
| COLGALT1 | 17.42191637 | 2.99E-05 | -1.366469068 |
| MAP7D1 | 17.54106527 | 2.81E-05 | -1.45193115 |
| DNAJB4 | 17.56517652 | 2.78E-05 | -1.49123899 |
| PSTPIP1 | 17.57138575 | 2.77E-05 | -2.385542187 |
| DOCK2 | 17.62929064 | 2.68E-05 | -1.573308575 |
| S100A14 | 17.65637267 | 2.65E-05 | 1.92879499 |
| CX3CR1 | 17.70919033 | 2.57E-05 | -3.250755999 |
| KRT10 | 17.72475491 | 2.55E-05 | 1.161719117 |
| S100A16 | 17.73430375 | 2.54E-05 | 2.195034024 |
| SKI | 17.73910304 | 2.53E-05 | -1.537223301 |
| PLBD1 | 17.79574205 | 2.46E-05 | -2.724904625 |
| IFNGR2 | 17.89984035 | 2.33E-05 | -1.39671945 |
| STK17B | 17.9106606 | 2.32E-05 | -1.027532209 |
| CPM | 17.92109666 | 2.30E-05 | -2.343957966 |
| AQP3 | 18.02170898 | 2.18E-05 | 1.489657019 |
| DENND1B | 18.0778812 | 2.12E-05 | -2.0050887 |
| CD300A | 18.08280996 | 2.12E-05 | -2.842226295 |
| OAS1 | 18.18022119 | 2.01E-05 | -2.17301414 |
| BCAS2 | 18.21147697 | 1.98E-05 | -1.326320214 |
| NPTN | 18.25139446 | 1.94E-05 | -1.805838251 |
| MCUR1 | 18.25622291 | 1.93E-05 | -2.637535647 |
| OSBPL11 | 18.28210145 | 1.90E-05 | -1.639034466 |
| ST3GAL6 | 18.2959369 | 1.89E-05 | 1.427414483 |
| NDE1 | 18.31535543 | 1.87E-05 | 1.287658932 |
| RREB1 | 18.34716964 | 1.84E-05 | -2.508182115 |
| AQP9 | 18.3764632 | 1.81E-05 | -7.373047496 |
| STX12 | 18.37684059 | 1.81E-05 | -1.726642368 |
| ITPR2 | 18.40028071 | 1.79E-05 | -1.901206299 |
| NUDT4 | 18.45816721 | 1.74E-05 | -2.472805659 |
| EDEM1 | 18.4684046 | 1.73E-05 | -1.29383741 |
| ACP5 | 18.49735771 | 1.70E-05 | -2.30895402 |
| WDFY2 | 18.51222477 | 1.69E-05 | -1.757618143 |
| LINC00963 | 18.5227635 | 1.68E-05 | -3.166200455 |
| SERPINB4 | 18.5974668 | 1.61E-05 | 2.392249349 |
| C6orf62 | 18.60723348 | 1.61E-05 | 1.302593622 |
| MCOLN1 | 18.64876111 | 1.57E-05 | -3.140360671 |
| EVL | 18.67606967 | 1.55E-05 | -3.867541112 |
| AC020916.1 | 18.69399534 | 1.53E-05 | -1.37277975 |
| TLE4 | 18.71515087 | 1.52E-05 | -1.329285268 |
| ARHGEF1 | 18.73495197 | 1.50E-05 | -1.007348434 |
| TRMT1 | 18.76424244 | 1.48E-05 | -1.877553991 |
| KCNAB2 | 18.77096053 | 1.47E-05 | -2.419282227 |
| ARRDC3 | 18.78192187 | 1.47E-05 | -1.662991117 |
| CRYL1 | 18.79458522 | 1.46E-05 | -3.435210942 |
| LGALS8 | 18.82682372 | 1.43E-05 | -2.209330613 |
| ATM | 18.84839342 | 1.42E-05 | -1.668279121 |
| CCL27 | 18.90001383 | 1.38E-05 | 3.899034432 |
| TMEM176B | 18.90318825 | 1.38E-05 | 1.213976729 |
| UAP1 | 19.02384104 | 1.29E-05 | -2.206953273 |
| BID | 19.03347529 | 1.28E-05 | -1.252095204 |
| RHOF | 19.04666672 | 1.28E-05 | 1.330143869 |
| FOXN2 | 19.04893567 | 1.27E-05 | 1.172731359 |
| WARS | 19.05549023 | 1.27E-05 | -1.311464441 |
| CCDC85B | 19.07096467 | 1.26E-05 | -4.121774701 |
| ACOT13 | 19.11901309 | 1.23E-05 | 1.919282481 |
| SNAPC1 | 19.15237652 | 1.21E-05 | -2.163860138 |
| ERGIC1 | 19.18827851 | 1.18E-05 | 1.594506251 |
| MAPRE2 | 19.19415895 | 1.18E-05 | -2.312816364 |
| FNIP2 | 19.21671536 | 1.17E-05 | -2.14365858 |
| C5AR2 | 19.21928753 | 1.17E-05 | -4.315575837 |
| KRT6B | 19.23523619 | 1.16E-05 | 2.181727328 |
| DUSP10 | 19.23564528 | 1.16E-05 | -1.391597422 |
| GRK3 | 19.37351776 | 1.07E-05 | 1.116863371 |
| CD200R1 | 19.39980909 | 1.06E-05 | -7.816353661 |
| TBC1D4 | 19.45279575 | 1.03E-05 | 3.099066771 |
| SMCHD1 | 19.58306321 | 9.63E-06 | -1.363205261 |
| NRP1 | 19.60758937 | 9.51E-06 | -3.894933086 |
| CXCL1 | 19.65280938 | 9.29E-06 | -6.312396223 |
| FDX1 | 19.69441813 | 9.09E-06 | -3.263122197 |
| ARMCX3 | 19.71169309 | 9.00E-06 | -1.664282844 |
| HVCN1 | 19.71232674 | 9.00E-06 | -3.479250655 |
| RAC2 | 19.74310583 | 8.86E-06 | -1.395785689 |
| LGALS7 | 19.76350832 | 8.76E-06 | 1.795645352 |
| SERPINF1 | 19.76435308 | 8.76E-06 | 1.132149598 |
| LILRB3 | 19.81368695 | 8.54E-06 | -4.631779914 |
| LAP3 | 19.90879765 | 8.12E-06 | 1.038221854 |
| C4orf48 | 19.93889381 | 8.00E-06 | -2.683381571 |
| LAMP1 | 19.97351531 | 7.85E-06 | -1.851103772 |
| PTGS1 | 20.00579199 | 7.72E-06 | -2.582791952 |
| LPAR6 | 20.09890824 | 7.35E-06 | -1.544760967 |
| LEPROT | 20.14463614 | 7.18E-06 | -1.273092467 |
| PURB | 20.17817243 | 7.06E-06 | -1.488385093 |
| BLNK | 20.21898288 | 6.91E-06 | -10.34917347 |
| TDP2 | 20.24184501 | 6.82E-06 | 1.266026141 |
| OAZ2 | 20.30148125 | 6.61E-06 | -1.510240626 |
| ME1 | 20.32442101 | 6.54E-06 | -3.39466887 |
| SERPINF2 | 20.34124998 | 6.48E-06 | 2.428510758 |
| PDXK | 20.36044951 | 6.41E-06 | -2.88239837 |
| PIK3R1 | 20.36099574 | 6.41E-06 | -1.794274928 |
| ATP1B1 | 20.39759085 | 6.29E-06 | -1.36753342 |
| ISG20L2 | 20.40299661 | 6.27E-06 | -2.052909855 |
| KIAA0930 | 20.40403166 | 6.27E-06 | -2.225188889 |
| ARID1B | 20.42502065 | 6.20E-06 | 1.029229892 |
| IQGAP2 | 20.44273383 | 6.14E-06 | -2.113479655 |
| IFRD1 | 20.44506481 | 6.14E-06 | -1.398235405 |
| NUMA1 | 20.46561728 | 6.07E-06 | -1.227761269 |
| BCL6 | 20.50395908 | 5.95E-06 | -1.858034813 |
| SUZ12 | 20.5723445 | 5.74E-06 | 1.11839716 |
| ARHGAP5 | 20.70865629 | 5.35E-06 | -2.231658056 |
| DSC2 | 20.73925885 | 5.26E-06 | -1.062824807 |
| EED | 20.74885733 | 5.24E-06 | 2.455141506 |
| PLEKHO2 | 20.75330223 | 5.22E-06 | -3.298955933 |
| ITGA5 | 20.78368463 | 5.14E-06 | -2.331972163 |
| EVI5 | 20.78728676 | 5.13E-06 | -2.071329017 |
| NCOR1 | 20.83917101 | 5.00E-06 | -1.293105344 |
| ABCA6 | 20.86774533 | 4.92E-06 | -3.781216775 |
| LAD1 | 20.9190632 | 4.79E-06 | 5.542735315 |
| TIFAB | 20.99148629 | 4.61E-06 | 2.220693992 |
| PPP1R15B | 21.00793772 | 4.57E-06 | -1.215060267 |
| FAM111A | 21.04752689 | 4.48E-06 | -2.079873484 |
| WAC-AS1 | 21.14779505 | 4.25E-06 | -1.928825933 |
| TFEC | 21.17603129 | 4.19E-06 | -2.816513501 |
| NME4 | 21.1760668 | 4.19E-06 | -3.279236449 |
| DNAJC21 | 21.17877437 | 4.18E-06 | 1.122784504 |
| GZF1 | 21.19263618 | 4.15E-06 | -3.479199833 |
| RPS6KA3 | 21.19327274 | 4.15E-06 | -1.389072856 |
| HES1 | 21.19627201 | 4.15E-06 | -2.19087397 |
| HPGDS | 21.21656309 | 4.10E-06 | -10.45485668 |
| NCK1 | 21.30621784 | 3.91E-06 | 1.103335307 |
| TRIP10 | 21.32077068 | 3.88E-06 | 1.321687078 |
| SH3BP2 | 21.34926803 | 3.83E-06 | -2.045089047 |
| CD80 | 21.37357131 | 3.78E-06 | 2.824267453 |
| KCNN4 | 21.38792778 | 3.75E-06 | -4.308001671 |
| SLC7A7 | 21.49517316 | 3.55E-06 | -2.101307403 |
| CTSL | 21.56547485 | 3.42E-06 | -2.923493485 |
| TRADD | 21.57211384 | 3.41E-06 | 1.796653018 |
| CD14 | 21.58252974 | 3.39E-06 | -6.317572656 |
| SPEN | 21.58509418 | 3.38E-06 | -1.407566158 |
| CALML3 | 21.58520289 | 3.38E-06 | 3.105332899 |
| NAV1 | 21.66455174 | 3.25E-06 | 1.44027073 |
| NAIP | 21.68522176 | 3.21E-06 | -4.797711637 |
| DIAPH2 | 21.75942869 | 3.09E-06 | -1.494553048 |
| TBXAS1 | 21.76391784 | 3.08E-06 | -5.426236308 |
| IGFBP7 | 21.78404836 | 3.05E-06 | -2.065971198 |
| TRAF3IP3 | 21.81529591 | 3.00E-06 | -1.570247744 |
| MANBA | 21.83115638 | 2.98E-06 | -1.435342733 |
| EIF4E | 21.86480628 | 2.93E-06 | -1.368933086 |
| HECA | 21.87651866 | 2.91E-06 | -1.425965249 |
| ADGRE5 | 21.87661943 | 2.91E-06 | -1.217220619 |
| KRTDAP | 21.91882222 | 2.84E-06 | 1.939448661 |
| DDX3Y | 21.98172378 | 2.75E-06 | -1.781775178 |
| RAB9A | 21.98251858 | 2.75E-06 | 1.624765297 |
| ARL14EP | 22.28991788 | 2.34E-06 | 1.972946228 |
| FAM53C | 22.35620366 | 2.26E-06 | -1.961861505 |
| DENND4A | 22.37908177 | 2.24E-06 | -1.102578336 |
| CCDC167 | 22.42596255 | 2.18E-06 | 2.047095016 |
| ARHGAP4 | 22.42657466 | 2.18E-06 | -2.989461023 |
| ELP2 | 22.43567523 | 2.17E-06 | 1.260878564 |
| BLVRB | 22.4594189 | 2.15E-06 | -1.133165375 |
| NCOA1 | 22.5030563 | 2.10E-06 | -1.529181174 |
| RAB7B | 22.60821382 | 1.99E-06 | -2.078092377 |
| CEP170 | 22.67980821 | 1.91E-06 | -1.36860671 |
| EHBP1L1 | 22.68940282 | 1.90E-06 | 1.214782857 |
| TLR2 | 22.69523455 | 1.90E-06 | -2.733888384 |
| ERGIC2 | 22.78592322 | 1.81E-06 | 1.06342453 |
| MX2 | 22.81079082 | 1.79E-06 | -2.834722712 |
| CXCL16 | 22.9149461 | 1.69E-06 | -1.170814197 |
| FCGBP | 22.9525139 | 1.66E-06 | -3.225813047 |
| NDEL1 | 22.95545194 | 1.66E-06 | -1.730330094 |
| AZIN1 | 23.03794939 | 1.59E-06 | -1.013691154 |
| NIPSNAP2 | 23.05824912 | 1.57E-06 | -2.275489555 |
| AC020656.1 | 23.09626255 | 1.54E-06 | -5.445958406 |
| ADGRE2 | 23.11970506 | 1.52E-06 | -5.90002322 |
| FAM50A | 23.14618036 | 1.50E-06 | -1.742752379 |
| SLC43A2 | 23.15451957 | 1.49E-06 | -2.158585352 |
| IL7R | 23.2002939 | 1.46E-06 | 1.414706966 |
| LDLRAD4 | 23.28039793 | 1.40E-06 | -3.14579059 |
| RCC2 | 23.31350429 | 1.38E-06 | -2.221410995 |
| TNFSF13B | 23.36005195 | 1.34E-06 | 1.031012215 |
| CHD2 | 23.36602431 | 1.34E-06 | -1.277809255 |
| SLC8A1 | 23.40226444 | 1.31E-06 | -2.084518618 |
| MKNK1 | 23.53238967 | 1.23E-06 | -3.000198177 |
| ARL4D | 23.61757008 | 1.18E-06 | -2.223032831 |
| SNCA | 23.6748605 | 1.14E-06 | -3.032332659 |
| DNPH1 | 23.77483662 | 1.08E-06 | -2.065767392 |
| ITGAM | 23.77840035 | 1.08E-06 | -10.90329286 |
| PDGFB | 23.90948393 | 1.01E-06 | -5.275629549 |
| FBP1 | 23.9151915 | 1.01E-06 | -6.290376751 |
| ARHGEF6 | 24.04029901 | 9.43E-07 | -2.72875061 |
| USP36 | 24.15027646 | 8.91E-07 | -1.845718263 |
| EPB41L3 | 24.16462168 | 8.84E-07 | -2.468440102 |
| SWAP70 | 24.18317743 | 8.76E-07 | 1.444583031 |
| MIR22HG | 24.22761753 | 8.56E-07 | -2.061386516 |
| MIR181A1HG | 24.24678891 | 8.47E-07 | -2.294157821 |
| ARHGAP6 | 24.25362626 | 8.44E-07 | -4.298021249 |
| STAT1 | 24.25459169 | 8.44E-07 | -1.803769502 |
| ABHD12 | 24.25873714 | 8.42E-07 | -2.722538447 |
| CD93 | 24.28479727 | 8.31E-07 | -2.963523816 |
| PRKCD | 24.33165161 | 8.11E-07 | -1.546867265 |
| ADAM15 | 24.34050804 | 8.07E-07 | -2.883187866 |
| PARVB | 24.38068784 | 7.91E-07 | -1.905555114 |
| DMKN | 24.45349382 | 7.61E-07 | 1.885514175 |
| RALGAPA1 | 24.4914888 | 7.46E-07 | -1.943671325 |
| VCAN | 24.52931691 | 7.32E-07 | -2.204671715 |
| ABHD5 | 24.57785308 | 7.14E-07 | -1.429675879 |
| NUFIP2 | 24.58207036 | 7.12E-07 | -1.232462982 |
| ATP13A3 | 24.67917547 | 6.77E-07 | -2.000727227 |
| OLR1 | 24.69142893 | 6.73E-07 | -7.227410102 |
| STK10 | 24.70614669 | 6.68E-07 | -2.022452923 |
| KIF1B | 24.80214585 | 6.35E-07 | -2.864908215 |
| UBL3 | 24.80249467 | 6.35E-07 | -2.115012861 |
| EPSTI1 | 24.81642237 | 6.31E-07 | 1.460583662 |
| TIMP2 | 24.84454738 | 6.21E-07 | -1.34905682 |
| SPG11 | 24.87734862 | 6.11E-07 | 1.420093386 |
| LYPD3 | 24.88657305 | 6.08E-07 | 1.412219699 |
| RASSF2 | 24.94907086 | 5.89E-07 | 1.188189552 |
| VDR | 24.96701212 | 5.83E-07 | -2.927881454 |
| RAB20 | 24.99833936 | 5.74E-07 | -8.763835526 |
| RTN1 | 25.03420931 | 5.63E-07 | -3.079491217 |
| EMB | 25.0574133 | 5.56E-07 | -1.937626685 |
| NAA25 | 25.07825297 | 5.51E-07 | 1.781238505 |
| BAG1 | 25.14635892 | 5.31E-07 | -2.060837128 |
| RIN3 | 25.21241256 | 5.14E-07 | -3.329139676 |
| TOR3A | 25.23882888 | 5.07E-07 | -2.181858014 |
| DDIT3 | 25.2458063 | 5.05E-07 | -2.165894432 |
| UBAC2 | 25.27741642 | 4.96E-07 | -1.749848492 |
| LIMD2 | 25.27882718 | 4.96E-07 | -1.804820885 |
| AP1B1 | 25.34348732 | 4.80E-07 | -2.414842775 |
| RAB32 | 25.35731357 | 4.76E-07 | -2.767989577 |
| RALGDS | 25.54234052 | 4.33E-07 | -2.362824218 |
| NCF4 | 25.54571718 | 4.32E-07 | -1.465994835 |
| GNA13 | 25.61459491 | 4.17E-07 | -1.551040634 |
| ITSN2 | 25.67862522 | 4.03E-07 | -1.307761927 |
| ATOX1 | 25.68471521 | 4.02E-07 | 1.217125419 |
| CCNH | 25.70961557 | 3.97E-07 | -2.241186451 |
| PRKACB | 25.75468501 | 3.88E-07 | -2.257869686 |
| NAGK | 25.75921418 | 3.87E-07 | -1.760593984 |
| NUP214 | 25.78777907 | 3.81E-07 | -1.443742299 |
| NBPF14 | 25.9073329 | 3.58E-07 | -2.256477209 |
| HACD4 | 25.98530684 | 3.44E-07 | -3.390726063 |
| THBS1 | 25.99385725 | 3.43E-07 | -3.708887803 |
| KCTD20 | 26.03670347 | 3.35E-07 | -1.469019272 |
| RXRA | 26.09758304 | 3.25E-07 | -2.592881 |
| TMEM176A | 26.12429524 | 3.20E-07 | 1.705018775 |
| ANO6 | 26.2015366 | 3.08E-07 | -2.216888201 |
| SLC9A3R1 | 26.22487694 | 3.04E-07 | -2.525561841 |
| OSM | 26.30916924 | 2.91E-07 | -4.328769783 |
| PNPLA8 | 26.32109731 | 2.89E-07 | -1.510580217 |
| LY86 | 26.38994131 | 2.79E-07 | -2.61092882 |
| PLXDC2 | 26.45948157 | 2.69E-07 | -3.188864494 |
| LRP1 | 26.64837032 | 2.44E-07 | -3.422076378 |
| CYTH4 | 26.67700542 | 2.40E-07 | -4.881739886 |
| SKAP2 | 26.68188167 | 2.40E-07 | -2.588281829 |
| LYST | 26.8908015 | 2.15E-07 | 1.25294947 |
| EVI2A | 26.96938095 | 2.07E-07 | -3.122626015 |
| SLC40A1 | 27.04588405 | 1.99E-07 | -4.536002674 |
| APAF1 | 27.09695472 | 1.94E-07 | -1.715392941 |
| HEXA | 27.12062088 | 1.91E-07 | -2.131883964 |
| OGA | 27.13679513 | 1.90E-07 | 1.467678773 |
| DSP | 27.16209245 | 1.87E-07 | 1.655560671 |
| SELPLG | 27.21057405 | 1.82E-07 | 1.275594494 |
| RBPJ | 27.22674623 | 1.81E-07 | -1.143961482 |
| CMTM3 | 27.26551649 | 1.77E-07 | -2.739746859 |
| IFI6 | 27.26655233 | 1.77E-07 | -2.198556688 |
| SNX9 | 27.3757869 | 1.68E-07 | -1.893641467 |
| GPRIN3 | 27.5155583 | 1.56E-07 | -4.569747532 |
| BRI3 | 27.61617298 | 1.48E-07 | -2.11993428 |
| ANPEP | 27.67122849 | 1.44E-07 | -3.239771353 |
| SLC7A5 | 27.68022522 | 1.43E-07 | -3.696952768 |
| ADIPOR1 | 27.83538261 | 1.32E-07 | -2.059186336 |
| FGD4 | 28.04144051 | 1.19E-07 | -3.920764369 |
| ABCA1 | 28.05776503 | 1.18E-07 | -1.83963125 |
| SLC39A8 | 28.05993792 | 1.18E-07 | -4.718409984 |
| SEMA4A | 28.06829544 | 1.17E-07 | -3.511242815 |
| BATF3 | 28.07918869 | 1.16E-07 | 2.370271354 |
| S100A7 | 28.14173273 | 1.13E-07 | 2.201023926 |
| PER1 | 28.20565129 | 1.09E-07 | -2.969530202 |
| SDF2L1 | 28.24487026 | 1.07E-07 | -3.001248369 |
| SDS | 28.32687626 | 1.02E-07 | -5.759650177 |
| DDX27 | 28.32776974 | 1.02E-07 | -1.742578001 |
| YPEL5 | 28.3385765 | 1.02E-07 | 1.046320688 |
| TOB1 | 28.40831988 | 9.82E-08 | -2.062990953 |
| H2AFV | 28.42353988 | 9.75E-08 | -1.591738769 |
| STARD8 | 28.45728483 | 9.58E-08 | -3.29500893 |
| TSC22D3 | 28.60484032 | 8.88E-08 | -1.755489551 |
| CMTM7 | 28.68480659 | 8.52E-08 | -4.137064811 |
| FOXO3 | 28.69228579 | 8.48E-08 | -2.079097195 |
| HIST1H2BK | 28.73371895 | 8.30E-08 | 2.294736761 |
| CCNL1 | 28.73744718 | 8.29E-08 | -1.034502977 |
| ATP8B4 | 28.75377364 | 8.22E-08 | -2.254865589 |
| RAB13 | 28.78635604 | 8.08E-08 | 1.505874261 |
| SEPTIN9 | 28.83617 | 7.88E-08 | -1.537168437 |
| GABARAPL1 | 28.8972434 | 7.63E-08 | -3.421086498 |
| ATP6V1B2 | 28.95768668 | 7.40E-08 | -1.617830095 |
| TUBB2B | 28.96000506 | 7.39E-08 | 4.711047326 |
| METTL9 | 28.99713739 | 7.25E-08 | -2.247247779 |
| TET2 | 29.04822706 | 7.06E-08 | -2.416799565 |
| RBM47 | 29.10875915 | 6.84E-08 | -1.770738152 |
| 1-Mar | 29.14212289 | 6.73E-08 | -3.480514147 |
| ARL5A | 29.15226301 | 6.69E-08 | -2.29060931 |
| LIPA | 29.1750864 | 6.61E-08 | -2.353778673 |
| ATP6V1C1 | 29.35769521 | 6.02E-08 | -2.11428792 |
| PPP1CB | 29.39700469 | 5.90E-08 | -1.071946091 |
| PXDC1 | 29.40289253 | 5.88E-08 | -2.833124321 |
| AMD1 | 29.45020981 | 5.74E-08 | -1.028759176 |
| MAP3K14 | 29.4560963 | 5.72E-08 | 2.268115279 |
| THEMIS2 | 29.49234899 | 5.61E-08 | -1.749388079 |
| CERS6 | 29.4989415 | 5.59E-08 | 2.278320474 |
| ELL2 | 29.61681129 | 5.26E-08 | -2.284697754 |
| CALHM6 | 29.81593668 | 4.75E-08 | -2.064744432 |
| CD47 | 29.88107257 | 4.59E-08 | -1.673648771 |
| RGL1 | 29.92955387 | 4.48E-08 | -2.818892042 |
| TNF | 30.30635556 | 3.69E-08 | -2.743102733 |
| SLAMF8 | 30.31485113 | 3.67E-08 | -4.340507109 |
| KDELR2 | 30.43693447 | 3.45E-08 | -2.59751095 |
| IL18BP | 30.63988857 | 3.11E-08 | -3.325676132 |
| ZNF106 | 30.64419977 | 3.10E-08 | -1.924353643 |
| CHRAC1 | 30.74539745 | 2.94E-08 | -1.46669566 |
| IFITM2 | 30.75655445 | 2.93E-08 | -1.02698381 |
| PKIB | 30.98425686 | 2.60E-08 | -2.598310763 |
| KRT6A | 31.1853956 | 2.35E-08 | 1.81696704 |
| OXSR1 | 31.20305375 | 2.32E-08 | -2.1927862 |
| PDE4B | 31.29073809 | 2.22E-08 | -1.357822931 |
| LRRK1 | 31.34664641 | 2.16E-08 | 2.63551631 |
| UPP1 | 31.43438618 | 2.06E-08 | -1.919795221 |
| PPA1 | 31.43605293 | 2.06E-08 | 1.700351466 |
| ANAPC16 | 31.54755921 | 1.95E-08 | 1.047217547 |
| KRT1 | 31.58452566 | 1.91E-08 | 2.002850215 |
| RAB5B | 31.72914054 | 1.77E-08 | 1.609759096 |
| BBX | 31.73274697 | 1.77E-08 | -1.275418396 |
| MPEG1 | 31.81029113 | 1.70E-08 | -3.665802985 |
| METTL21A | 31.91153623 | 1.61E-08 | -2.62866406 |
| CXorf21 | 31.94272894 | 1.59E-08 | -3.796649202 |
| GPR155 | 31.94702885 | 1.58E-08 | -2.23201841 |
| MEF2A | 32.06633318 | 1.49E-08 | -2.269967856 |
| NKRF | 32.10544309 | 1.46E-08 | -2.631985105 |
| SLC43A3 | 32.12061495 | 1.45E-08 | -2.665310625 |
| ADAM10 | 32.17129745 | 1.41E-08 | -1.591582493 |
| SCARB2 | 32.27686894 | 1.34E-08 | -2.291769609 |
| MAFF | 32.41166176 | 1.25E-08 | -1.561099413 |
| AGTRAP | 32.46808773 | 1.21E-08 | -2.004889475 |
| RB1 | 32.50569078 | 1.19E-08 | -1.761789974 |
| EP300 | 32.76570353 | 1.04E-08 | -2.300491173 |
| DAPP1 | 32.76969894 | 1.04E-08 | 1.575186339 |
| YTHDF3 | 32.86081003 | 9.90E-09 | -2.480876944 |
| GATM | 32.90451167 | 9.68E-09 | -2.528715477 |
| EPN1 | 32.94079156 | 9.50E-09 | -2.927695289 |
| RBM17 | 32.96841552 | 9.37E-09 | -1.932518178 |
| HOPX | 32.9987316 | 9.22E-09 | 2.636924333 |
| MARCKS | 33.15246938 | 8.52E-09 | 1.042213574 |
| RAB8B | 33.1846664 | 8.38E-09 | 1.58923052 |
| KCTD12 | 33.36755206 | 7.63E-09 | -2.547675078 |
| MCUB | 33.49770296 | 7.13E-09 | -3.91101761 |
| SKIL | 33.49989551 | 7.13E-09 | -1.989049269 |
| CHCHD10 | 33.53096454 | 7.01E-09 | -6.399055304 |
| PTX3 | 33.54624389 | 6.96E-09 | -8.354074929 |
| DCAF6 | 33.59552935 | 6.78E-09 | 1.919242283 |
| EHD1 | 33.61894757 | 6.70E-09 | -2.230557922 |
| LRRC58 | 33.84354454 | 5.97E-09 | -2.001899281 |
| METTL7A | 34.07197549 | 5.31E-09 | -1.892045801 |
| GIMAP4 | 34.21197959 | 4.94E-09 | -4.211576825 |
| AKR1B1 | 34.26766389 | 4.80E-09 | -2.684137924 |
| DUSP3 | 34.37745723 | 4.54E-09 | -2.324805984 |
| NFKBIE | 34.39675625 | 4.49E-09 | -2.875325933 |
| JMJD6 | 34.59260328 | 4.06E-09 | -1.879010357 |
| GPCPD1 | 34.67400032 | 3.90E-09 | -2.328815614 |
| B3GNT5 | 34.6939723 | 3.86E-09 | -3.30994304 |
| FLI1 | 34.77852873 | 3.69E-09 | -2.659932517 |
| GRASP | 34.78668086 | 3.68E-09 | -2.841092877 |
| EFHD2 | 34.79867043 | 3.66E-09 | -1.943021789 |
| MTCH1 | 35.19567553 | 2.98E-09 | -2.242111192 |
| PTPN18 | 35.23391897 | 2.92E-09 | -2.674513607 |
| KRT16 | 35.2410643 | 2.91E-09 | 1.892544291 |
| H2AFX | 35.46319252 | 2.60E-09 | -2.354922944 |
| BAG3 | 35.49257404 | 2.56E-09 | -2.336092088 |
| PLAU | 35.55796128 | 2.48E-09 | -3.050965235 |
| KRT5 | 35.73437649 | 2.26E-09 | 1.672219829 |
| SNHG15 | 35.81517753 | 2.17E-09 | -1.550298522 |
| SRSF9 | 35.88039108 | 2.10E-09 | -2.185265058 |
| KMO | 36.02745294 | 1.95E-09 | -5.170006261 |
| OSBPL1A | 36.06820127 | 1.91E-09 | -2.236120008 |
| CLEC4A | 36.2143275 | 1.77E-09 | -2.507517114 |
| TGIF1 | 36.32124379 | 1.67E-09 | -1.440896736 |
| KDM7A | 36.33695271 | 1.66E-09 | -4.328552824 |
| COCH | 36.3567083 | 1.64E-09 | 6.064621921 |
| PHLDA1 | 36.47229853 | 1.55E-09 | -4.568044736 |
| CST7 | 36.50294131 | 1.52E-09 | 1.730441926 |
| QKI | 36.5339548 | 1.50E-09 | -1.208933627 |
| METRNL | 36.6273334 | 1.43E-09 | -4.41341235 |
| MXD1 | 36.71055257 | 1.37E-09 | -1.926390156 |
| ZNF385A | 36.97642456 | 1.20E-09 | -2.226554027 |
| NBPF26 | 37.33119021 | 9.97E-10 | -1.862529983 |
| KRT14 | 37.36567121 | 9.79E-10 | 1.394019361 |
| SAV1 | 37.40480849 | 9.60E-10 | -2.785563723 |
| ANTXR2 | 37.45560208 | 9.35E-10 | 1.614116076 |
| PLEKHO1 | 37.47447492 | 9.26E-10 | -2.214782002 |
| NDRG2 | 37.55132771 | 8.90E-10 | -3.28631427 |
| CYB5A | 37.57857252 | 8.78E-10 | 1.985895758 |
| PPM1K | 37.61706958 | 8.61E-10 | 2.495162587 |
| LUCAT1 | 37.77959905 | 7.92E-10 | -5.798102399 |
| TPRA1 | 37.81430821 | 7.78E-10 | -2.559508168 |
| SSH2 | 37.88876985 | 7.49E-10 | -1.511492821 |
| HCAR2 | 37.95327042 | 7.25E-10 | -5.11280548 |
| AC009630.4 | 37.95991762 | 7.22E-10 | -13.89342233 |
| SINHCAF | 37.97744485 | 7.16E-10 | 1.750411532 |
| SERTAD1 | 37.99914286 | 7.08E-10 | -1.825462861 |
| WTAP | 38.11552266 | 6.67E-10 | -1.780266456 |
| ASAH1 | 38.12127572 | 6.65E-10 | -2.19194213 |
| KLF3 | 38.17613088 | 6.46E-10 | -3.222105641 |
| IRF2 | 38.22243629 | 6.31E-10 | 1.041677591 |
| UTRN | 38.38757026 | 5.80E-10 | -2.505036712 |
| CTSD | 38.56387133 | 5.30E-10 | -2.07496491 |
| MBP | 38.60929812 | 5.18E-10 | -3.30385348 |
| CXCL2 | 38.71561237 | 4.90E-10 | -13.87874455 |
| HSPH1 | 38.80233478 | 4.69E-10 | -1.244139571 |
| IL15 | 38.81603098 | 4.66E-10 | 2.31171633 |
| JUND | 38.90377232 | 4.45E-10 | -1.834510792 |
| TMEM131 | 38.99359384 | 4.25E-10 | 1.779601208 |
| NRROS | 39.37697267 | 3.49E-10 | -4.45701108 |
| SLC25A19 | 39.38888803 | 3.47E-10 | -2.458345035 |
| C9orf72 | 39.39096803 | 3.47E-10 | -1.489956818 |
| NIBAN1 | 39.59683804 | 3.12E-10 | 1.719266016 |
| GASK1B | 39.73524234 | 2.91E-10 | -2.669175278 |
| ITGAX | 39.90850585 | 2.66E-10 | -3.677039894 |
| SMIM25 | 40.01909677 | 2.51E-10 | -5.900739207 |
| PAPSS2 | 40.02382518 | 2.51E-10 | -2.653958471 |
| TTF2 | 40.11893569 | 2.39E-10 | 1.95329501 |
| CDC42EP2 | 40.20045199 | 2.29E-10 | -4.807507053 |
| CSF3R | 40.39976566 | 2.07E-10 | -4.00599617 |
| TSPYL2 | 40.42204574 | 2.05E-10 | -3.690542324 |
| PLD3 | 40.53026275 | 1.94E-10 | -2.77795226 |
| ATP1B3 | 40.55261878 | 1.91E-10 | -1.481609813 |
| LYN | 40.57492499 | 1.89E-10 | -1.695359463 |
| MAFB | 40.66976507 | 1.80E-10 | -3.133071599 |
| CD99 | 40.71447435 | 1.76E-10 | -1.025595148 |
| CCL4L2 | 40.76857698 | 1.71E-10 | -25.66075979 |
| SH3BP5 | 40.79904512 | 1.69E-10 | -2.140810522 |
| JMJD1C | 41.05664957 | 1.48E-10 | -1.835177022 |
| TXNIP | 41.21566831 | 1.36E-10 | 1.322697573 |
| EGR1 | 41.23101504 | 1.35E-10 | -2.578504452 |
| RBBP8 | 41.27883022 | 1.32E-10 | -2.977162155 |
| MID1IP1 | 41.30779879 | 1.30E-10 | -3.288771773 |
| PPT1 | 41.42593355 | 1.22E-10 | -1.929787847 |
| LILRB2 | 41.46091568 | 1.20E-10 | -3.944893322 |
| JOSD1 | 41.55306314 | 1.15E-10 | -2.649813195 |
| DPYSL2 | 41.61274507 | 1.11E-10 | 1.137969618 |
| TRIB1 | 41.91645341 | 9.53E-11 | 1.31329196 |
| MIIP | 41.95376584 | 9.35E-11 | 1.340685103 |
| SELENOW | 42.00423397 | 9.11E-11 | 1.288526589 |
| IRF8 | 42.06721196 | 8.82E-11 | -2.33385795 |
| SMIM4 | 42.06800371 | 8.82E-11 | -2.023556556 |
| EREG | 42.30462061 | 7.81E-11 | -7.231920795 |
| CD36 | 42.34734696 | 7.64E-11 | -6.771148487 |
| NCOA4 | 42.45058609 | 7.25E-11 | -1.783010397 |
| RASGEF1B | 42.52524173 | 6.98E-11 | -2.037848594 |
| MS4A7 | 42.59313712 | 6.74E-11 | -8.625458256 |
| PALM2-AKAP2 | 42.62654233 | 6.63E-11 | 1.435793337 |
| EPS8 | 42.65756145 | 6.52E-11 | -2.672408421 |
| CYBB | 42.71172091 | 6.34E-11 | -5.463699035 |
| FKBP4 | 42.72895957 | 6.29E-11 | -3.189877237 |
| PTPRC | 42.76069958 | 6.19E-11 | -1.086965441 |
| PHLDA2 | 42.7860413 | 6.11E-11 | -2.313390617 |
| AP1S2 | 42.90439781 | 5.75E-11 | -2.025347589 |
| C12orf57 | 43.01393319 | 5.44E-11 | 1.266731518 |
| CD52 | 43.0708894 | 5.28E-11 | -1.573098692 |
| SAMHD1 | 43.13384993 | 5.11E-11 | -2.170472704 |
| KLF10 | 43.15893911 | 5.05E-11 | -1.518489328 |
| IRF2BP2 | 43.21094224 | 4.91E-11 | -1.640862801 |
| RNASE1 | 43.21222114 | 4.91E-11 | -3.098712534 |
| NOTCH2 | 43.30358669 | 4.69E-11 | -2.000173133 |
| CCND2 | 43.33682659 | 4.61E-11 | -2.206265737 |
| COA1 | 43.39797815 | 4.47E-11 | 1.980053673 |
| MOSPD2 | 43.40712601 | 4.45E-11 | 1.852615349 |
| OTUD1 | 43.6417892 | 3.94E-11 | -2.812388306 |
| POU2F2 | 43.85855716 | 3.53E-11 | -4.286530869 |
| YME1L1 | 44.15918989 | 3.03E-11 | -1.283286053 |
| GEM | 44.3648716 | 2.73E-11 | -3.942728943 |
| NSUN6 | 44.46296148 | 2.59E-11 | -3.194161827 |
| CITED2 | 44.76239424 | 2.22E-11 | -2.585089453 |
| BNIP3L | 44.81517658 | 2.17E-11 | -1.497628884 |
| CCL3 | 45.57444883 | 1.47E-11 | -7.819944454 |
| DAB2 | 45.61973808 | 1.44E-11 | -4.82862458 |
| FCGR2B | 45.62776455 | 1.43E-11 | -4.918520796 |
| DDX3X | 46.14424481 | 1.10E-11 | -1.482386128 |
| ARL4A | 46.46364934 | 9.33E-12 | -2.629518307 |
| SFN | 46.46887846 | 9.31E-12 | 2.298742397 |
| CASP1 | 46.48917875 | 9.21E-12 | -3.163989165 |
| F11R | 46.59095119 | 8.75E-12 | 1.589992604 |
| KRT17 | 47.13392995 | 6.63E-12 | 4.134021739 |
| CEP350 | 47.2501264 | 6.25E-12 | 1.535459644 |
| TVP23A | 47.47976754 | 5.56E-12 | 2.082190615 |
| HSPD1 | 47.70208738 | 4.96E-12 | -1.287978953 |
| VPS37B | 48.46968704 | 3.35E-12 | -4.320145148 |
| PDE4A | 49.02436836 | 2.53E-12 | -3.418457838 |
| HEXB | 49.18820939 | 2.33E-12 | -3.432108972 |
| CACUL1 | 49.38541375 | 2.10E-12 | -2.105456044 |
| MCM5 | 49.42404739 | 2.06E-12 | 1.48498879 |
| ANP32B | 49.43079409 | 2.05E-12 | -1.907471416 |
| THBD | 49.62703151 | 1.86E-12 | -4.628278098 |
| PNISR | 49.71876113 | 1.77E-12 | 1.050234162 |
| MIDN | 50.02853656 | 1.52E-12 | -1.954925024 |
| MEF2C | 50.06370058 | 1.49E-12 | -3.114232399 |
| PLTP | 50.11474056 | 1.45E-12 | -3.091430853 |
| AC007384.1 | 50.13974463 | 1.43E-12 | -3.783926331 |
| PDE4DIP | 50.46096474 | 1.22E-12 | -2.472407434 |
| HSP90B1 | 50.64092724 | 1.11E-12 | -1.195889879 |
| ID2 | 50.66806292 | 1.09E-12 | 1.484227941 |
| IL4I1 | 50.81686074 | 1.01E-12 | 2.323072843 |
| MFSD1 | 51.05109259 | 9.00E-13 | -2.782711754 |
| PILRA | 51.30211477 | 7.92E-13 | -3.246426457 |
| C1orf162 | 51.58432263 | 6.86E-13 | -3.040166337 |
| SERPINB9 | 51.63607938 | 6.68E-13 | -2.15379906 |
| TMEM123 | 51.67294227 | 6.56E-13 | -1.388142791 |
| GRSF1 | 51.75215416 | 6.30E-13 | 1.979562931 |
| HBEGF | 52.06591918 | 5.37E-13 | -4.500949196 |
| CYCS | 52.1014127 | 5.27E-13 | -1.553869736 |
| TUBA1C | 52.2589807 | 4.86E-13 | 1.248346109 |
| ABL2 | 52.28448293 | 4.80E-13 | -4.665601671 |
| PTGS2 | 52.333437 | 4.68E-13 | -6.503026312 |
| SUB1 | 52.57864049 | 4.13E-13 | 1.036915728 |
| LPXN | 52.65167286 | 3.98E-13 | -3.342288056 |
| RIPK2 | 52.65965394 | 3.97E-13 | -2.5511336 |
| ZEB2 | 52.74686176 | 3.79E-13 | -1.867887325 |
| MRC1 | 52.79991995 | 3.69E-13 | -9.578065208 |
| SNX10 | 52.81149659 | 3.67E-13 | -2.130069516 |
| ERICH1 | 52.91187993 | 3.49E-13 | 1.795542261 |
| ANKRD37 | 53.13086399 | 3.12E-13 | -4.075041308 |
| IGSF6 | 53.22512457 | 2.97E-13 | -2.719533292 |
| EBI3 | 53.23543693 | 2.96E-13 | 3.693369521 |
| CD274 | 53.27281691 | 2.90E-13 | 4.117486544 |
| CELF2 | 53.34445126 | 2.80E-13 | 1.133008541 |
| ATP5F1D | 53.52637896 | 2.55E-13 | -3.128226027 |
| LCP2 | 53.53732265 | 2.54E-13 | -4.052246347 |
| BLVRA | 53.72274493 | 2.31E-13 | 2.146035271 |
| GNB2 | 53.7620028 | 2.26E-13 | -1.987631047 |
| GADD45B | 53.81953306 | 2.20E-13 | -1.299029432 |
| SYAP1 | 53.95380007 | 2.05E-13 | -2.603414848 |
| CCR7 | 53.98333366 | 2.02E-13 | 2.075570087 |
| TAP2 | 53.98634772 | 2.02E-13 | 1.513554593 |
| CSF2RB | 54.04470645 | 1.96E-13 | 2.086960094 |
| FYB1 | 54.34609538 | 1.68E-13 | -2.592717733 |
| RNF130 | 54.83022046 | 1.31E-13 | -4.498359351 |
| F13A1 | 55.14060865 | 1.12E-13 | -4.638547703 |
| ADA2 | 55.50816709 | 9.30E-14 | -6.093208308 |
| HLX | 55.54219097 | 9.15E-14 | 1.979380319 |
| AREG | 55.59727092 | 8.89E-14 | -7.556357669 |
| CFD | 55.81531832 | 7.96E-14 | -4.952104361 |
| CD37 | 55.86661433 | 7.76E-14 | -1.285996871 |
| BCL3 | 56.04808115 | 7.07E-14 | -2.161331245 |
| DOK2 | 56.22578925 | 6.46E-14 | -2.795206777 |
| AVPI1 | 56.25147593 | 6.37E-14 | -3.745868576 |
| SELENOP | 56.45817933 | 5.74E-14 | -5.144634277 |
| CEBPD | 56.6482508 | 5.21E-14 | -4.67599071 |
| CEBPB | 56.65270142 | 5.20E-14 | -3.869114986 |
| VEGFA | 56.98955214 | 4.39E-14 | -3.865639684 |
| PRNP | 57.03046204 | 4.30E-14 | -2.500732566 |
| RNASET2 | 57.15737081 | 4.02E-14 | -3.336352169 |
| CLEC2B | 57.18719922 | 3.96E-14 | -2.096196119 |
| BRD2 | 57.30361123 | 3.73E-14 | -1.356234709 |
| RNASE6 | 57.75622734 | 2.96E-14 | -7.256504813 |
| FCGR2A | 57.83873244 | 2.84E-14 | -6.152945114 |
| GPAT3 | 57.83899082 | 2.84E-14 | -7.051429352 |
| LGALS7B | 58.04996444 | 2.55E-14 | 1.840556709 |
| ATP1A1 | 58.06866769 | 2.53E-14 | -2.494132858 |
| PLEK | 58.27751327 | 2.28E-14 | -1.230442089 |
| EMILIN2 | 58.72591436 | 1.81E-14 | -1.65141313 |
| LAMP3 | 58.76468423 | 1.78E-14 | 5.242410075 |
| MAP4 | 58.82377945 | 1.72E-14 | 1.245852001 |
| CRTAP | 58.91305028 | 1.64E-14 | -2.466707629 |
| PRELID1 | 59.1759584 | 1.44E-14 | -3.373314122 |
| B4GALT1 | 59.27585406 | 1.37E-14 | -3.325543048 |
| LIMCH1 | 59.30852846 | 1.34E-14 | 3.374014874 |
| ACAP2 | 59.37780059 | 1.30E-14 | -2.143077933 |
| IL17RA | 59.48856785 | 1.23E-14 | -2.271781441 |
| NFKBID | 60.00057542 | 9.44E-15 | -1.722423392 |
| NMRK1 | 60.06319146 | 9.21E-15 | 3.004832795 |
| N4BP2L2 | 60.44074238 | 7.55E-15 | 1.574707239 |
| C1QB | 60.50302887 | 7.33E-15 | -5.452369515 |
| KDM2B | 60.64793794 | 6.77E-15 | 2.378265387 |
| TUBB | 60.68302536 | 6.66E-15 | 1.129054407 |
| SERPINA1 | 60.94002616 | 5.88E-15 | -3.145271697 |
| CCL4 | 61.19692793 | 5.22E-15 | -9.231318036 |
| GAS6 | 61.27615455 | 5.00E-15 | -2.516228317 |
| S100A2 | 61.41453613 | 4.66E-15 | 2.592387174 |
| ARL4C | 61.45676231 | 4.55E-15 | -2.75022194 |
| CKLF | 61.98941654 | 3.44E-15 | -2.253862701 |
| RASSF5 | 62.24974332 | 3.00E-15 | -3.958251206 |
| FPR3 | 62.52522689 | 2.66E-15 | -4.805549675 |
| TOB2 | 62.66678856 | 2.44E-15 | -3.122651549 |
| POGLUT1 | 63.37867181 | 1.67E-15 | 2.970747275 |
| GLUD1 | 63.8992135 | 1.33E-15 | -1.525860153 |
| CD48 | 64.07258927 | 1.22E-15 | -2.72205763 |
| ARHGAP18 | 64.35908347 | 9.99E-16 | -3.575773874 |
| GAS7 | 64.71910325 | 8.88E-16 | -4.00366978 |
| SIK1B | 65.44261778 | 5.55E-16 | -3.558878025 |
| CDC42EP3 | 65.4895453 | 5.55E-16 | -3.524697281 |
| G0S2 | 65.73117193 | 5.55E-16 | -3.32203673 |
| CXCL3 | 66.3465764 | 3.33E-16 | -9.499992684 |
| APLP2 | 66.77670701 | 3.33E-16 | -1.601358154 |
| PRDX1 | 66.92110765 | 3.33E-16 | -1.116314074 |
| PIM3 | 67.57458586 | 2.22E-16 | -2.834797546 |
| MYADM | 68.5591435 | 1.11E-16 | -1.86695394 |
| CYBA | 68.81488763 | 1.11E-16 | -1.016104863 |
| RAB10 | 69.01037245 | 1.11E-16 | 1.334099558 |
| CDKN1B | 69.29014285 | 1.11E-16 | -1.621213949 |
| EIF4A3 | 69.59930862 | 1.11E-16 | -5.340500729 |
| ICAM1 | 69.60150138 | 1.11E-16 | -1.702341917 |
| MAP1LC3B | 70.04679071 | 1.11E-16 | -2.401420405 |
| WSB1 | 70.13309716 | 0 | -1.421026633 |
| NPC2 | 70.49876424 | 0 | -1.207015872 |
| TIPARP | 70.75469888 | 0 | -3.153741937 |
| STK4 | 71.14796034 | 0 | 1.450463534 |
| REL | 71.15880137 | 0 | -1.45063153 |
| MRPS33 | 71.86851074 | 0 | 1.678742442 |
| MAP3K8 | 71.91852159 | 0 | -2.533674532 |
| DIPK2A | 72.09899748 | 0 | -3.094631984 |
| TWISTNB | 72.14172782 | 0 | -3.499004412 |
| GK | 72.41904992 | 0 | -3.894548595 |
| RGCC | 72.42868718 | 0 | -8.408239888 |
| ZYX | 72.68963254 | 0 | -1.556131789 |
| PIK3AP1 | 73.05175433 | 0 | -4.722638572 |
| IL10RA | 73.29368466 | 0 | -1.937143806 |
| C1QA | 73.39432079 | 0 | -4.943533497 |
| MGAT1 | 73.42039209 | 0 | -2.202587726 |
| RAC1 | 73.45721014 | 0 | -1.386008605 |
| MAP2K3 | 73.92682739 | 0 | -4.783978156 |
| IL32 | 74.03955024 | 0 | 3.221086011 |
| SPAG9 | 74.40712524 | 0 | -2.944811532 |
| HNRNPU | 74.72810963 | 0 | -1.003350616 |
| ZFAND5 | 74.89442863 | 0 | -1.98938831 |
| CHD1 | 75.29422934 | 0 | -2.028148363 |
| MYO1G | 75.84537445 | 0 | 2.129066955 |
| JAML | 76.70061636 | 0 | -3.935595586 |
| PFKFB3 | 77.23521164 | 0 | -2.949838393 |
| LIMS1 | 77.4765596 | 0 | -3.249781521 |
| TRAFD1 | 77.49681137 | 0 | 3.180871711 |
| TGFBR2 | 77.63701323 | 0 | -2.061343967 |
| PLSCR1 | 78.68643383 | 0 | -2.939178565 |
| BTG2 | 78.78686625 | 0 | -2.383548242 |
| XBP1 | 79.16587866 | 0 | -4.080102647 |
| PPP1R10 | 79.68140932 | 0 | -2.341255604 |
| TGFBI | 81.76715542 | 0 | -3.881442592 |
| HSD11B1-AS1 | 82.25054988 | 0 | 7.883581725 |
| IVNS1ABP | 82.43503225 | 0 | -2.499246634 |
| CORO1A | 83.18426626 | 0 | -2.527282505 |
| ZNF331 | 83.25567848 | 0 | -4.807254146 |
| ARL5B | 83.58289654 | 0 | -4.074581123 |
| FOSL2 | 83.64743256 | 0 | -3.443830777 |
| CLEC10A | 84.00548324 | 0 | -2.088505813 |
| MMP19 | 84.08551086 | 0 | -6.327685278 |
| ETS2 | 84.11206801 | 0 | -3.400684143 |
| PPIF | 84.44778276 | 0 | -4.905209674 |
| PLAAT3 | 85.34106413 | 0 | 2.973576958 |
| KLF2 | 87.66263758 | 0 | -4.54459032 |
| GNS | 88.90432362 | 0 | -2.252687971 |
| FRMD4B | 89.08112509 | 0 | -2.233825709 |
| TSPO | 89.48595027 | 0 | -1.659882545 |
| PYCARD | 89.62268226 | 0 | -3.260829914 |
| KIF2A | 90.27273461 | 0 | 3.438654069 |
| MAP3K13 | 90.47854596 | 0 | 4.092685545 |
| CCL19 | 91.02228819 | 0 | 4.251257429 |
| ZFAS1 | 91.67699159 | 0 | 1.30615836 |
| CTSS | 92.4287859 | 0 | -1.672820933 |
| RASSF4 | 92.66155483 | 0 | 1.817327616 |
| CSRNP1 | 93.17571869 | 0 | -3.742822374 |
| BASP1 | 93.4565205 | 0 | 2.085368956 |
| PFN1 | 93.70545664 | 0 | -1.137450236 |
| CKS2 | 94.05594038 | 0 | -3.102006843 |
| RGS2 | 94.18524308 | 0 | -2.693565813 |
| CD4 | 94.70157003 | 0 | -1.554583678 |
| PTPRE | 95.1854379 | 0 | -3.722874311 |
| SOCS3 | 95.37963909 | 0 | -2.43477888 |
| HSPA5 | 95.94798711 | 0 | -2.465716411 |
| MAT2A | 96.02665925 | 0 | -2.841351684 |
| IL1B | 96.14893255 | 0 | -12.29510693 |
| MS4A6A | 96.56432421 | 0 | -6.147956979 |
| CLPTM1 | 96.94503 | 0 | 2.561393363 |
| MCTP1 | 97.26258044 | 0 | -2.242292504 |
| TUBA1B | 97.28641616 | 0 | -1.226610881 |
| ARHGAP24 | 97.45166792 | 0 | -4.435564273 |
| CARD16 | 98.00099379 | 0 | -1.433132086 |
| TAGAP | 98.23111169 | 0 | -4.738029809 |
| GNG5 | 98.89134753 | 0 | 1.100104516 |
| LY75 | 99.36604269 | 0 | 3.995074318 |
| UBXN11 | 100.2375547 | 0 | -3.881465228 |
| CSF2RA | 100.2494395 | 0 | 2.290918724 |
| COMMD2 | 102.8614244 | 0 | 1.940238975 |
| NR4A3 | 102.9903075 | 0 | -2.482275355 |
| GLRX | 103.1421307 | 0 | -3.905471077 |
| HLA-DQA1 | 103.5931538 | 0 | -1.077142057 |
| RAB29 | 103.9063918 | 0 | 2.93433793 |
| DUSP1 | 104.6824687 | 0 | -1.183774918 |
| RHOB | 104.6839122 | 0 | -2.379490071 |
| RILPL2 | 105.1927406 | 0 | -3.550923197 |
| STX11 | 105.9547208 | 0 | -4.15049239 |
| GLA | 105.9987338 | 0 | -4.185619192 |
| MTHFD2 | 107.7249667 | 0 | 1.851740053 |
| KLF4 | 108.807327 | 0 | -2.981674665 |
| PSME2 | 109.6693237 | 0 | 1.690744403 |
| GLUL | 109.8958332 | 0 | -3.496340703 |
| CSF1R | 110.5495705 | 0 | -4.740759557 |
| MMP9 | 110.5828216 | 0 | -6.448809705 |
| SQSTM1 | 112.5719361 | 0 | -2.090238853 |
| CALR | 113.0586487 | 0 | -2.155068229 |
| CAPG | 113.3257199 | 0 | 1.61400109 |
| PLIN2 | 114.0648127 | 0 | -3.039766315 |
| BCL2A1 | 115.2383138 | 0 | -2.384632697 |
| CHMP1B | 118.1378795 | 0 | -3.225541465 |
| CREM | 118.2630751 | 0 | -4.215952656 |
| ANXA1 | 118.3969267 | 0 | -1.631928279 |
| SERP1 | 119.3821033 | 0 | -2.381472531 |
| CD55 | 120.3034319 | 0 | -2.915786878 |
| CD302 | 121.8301243 | 0 | -3.865972845 |
| GRB2 | 123.4424514 | 0 | -2.954840078 |
| ZC3H12A | 123.828458 | 0 | -3.798853803 |
| KDM6B | 124.2582815 | 0 | -3.314210009 |
| PPP1R15A | 124.9539875 | 0 | -2.102693298 |
| C1QC | 125.3219083 | 0 | -5.557961512 |
| FOS | 126.6639036 | 0 | -1.967379222 |
| APOBEC3A | 126.7694711 | 0 | -6.45020024 |
| EZR | 128.1386663 | 0 | -2.101936481 |
| UCP2 | 129.5669512 | 0 | 2.365764862 |
| NET1 | 129.7877322 | 0 | 3.308109929 |
| HCST | 130.2297392 | 0 | -2.679293817 |
| TNFAIP3 | 131.2942226 | 0 | -2.445104818 |
| DNAJA1 | 131.6626136 | 0 | -2.116088959 |
| HMGN3 | 131.8268149 | 0 | 1.942179853 |
| FCGRT | 131.9804743 | 0 | -3.235220973 |
| NINJ1 | 132.9557978 | 0 | -3.382533261 |
| IL1RN | 133.1254184 | 0 | -5.55681758 |
| CD68 | 135.0798939 | 0 | -4.148034224 |
| CD1B | 135.4307263 | 0 | 4.776590813 |
| IL1R2 | 138.4651099 | 0 | -6.914547435 |
| SLFN11 | 140.1146839 | 0 | -4.620959042 |
| CCL22 | 141.0112389 | 0 | 4.783752168 |
| SELENOK | 142.607687 | 0 | -3.127107843 |
| ATP6V1G1 | 143.5187447 | 0 | 1.757153425 |
| FGL2 | 147.9508176 | 0 | -4.048443869 |
| ATF3 | 150.1296177 | 0 | -3.678037745 |
| ITM2B | 153.0563359 | 0 | -2.315581208 |
| SGK1 | 154.2574242 | 0 | -4.55880621 |
| EMP3 | 154.9562462 | 0 | -1.596141287 |
| PDCD1LG2 | 156.7230291 | 0 | 4.104609517 |
| NUB1 | 157.3363949 | 0 | 3.555477559 |
| ATP6V0C | 159.3896538 | 0 | -2.729249094 |
| CPVL | 160.7232071 | 0 | -4.062866426 |
| MALAT1 | 160.7476868 | 0 | 1.352779883 |
| CTSB | 161.3150058 | 0 | -3.061231759 |
| ANXA6 | 161.4799013 | 0 | 3.119882547 |
| CXCL8 | 165.8023739 | 0 | -7.410911295 |
| PSAP | 167.9923496 | 0 | -1.579163598 |
| FCER1A | 168.5702689 | 0 | -5.07293173 |
| COTL1 | 169.4204714 | 0 | -2.444437978 |
| IER3 | 170.2805874 | 0 | -2.746951289 |
| CRIP1 | 170.2951355 | 0 | 1.47658525 |
| PHACTR1 | 170.8029365 | 0 | -3.425617043 |
| LST1 | 170.8679083 | 0 | -3.388836065 |
| HLA-DQB1 | 174.9460399 | 0 | -1.312803197 |
| LAPTM5 | 177.13848 | 0 | -3.57894493 |
| RPS2 | 180.3927687 | 0 | -1.756529613 |
| SH3BGRL3 | 182.9147205 | 0 | -1.31207592 |
| RPS27L | 183.0354102 | 0 | 2.85981688 |
| DDIT4 | 183.5768269 | 0 | -2.714369392 |
| NFE2L2 | 186.0694008 | 0 | -2.953024331 |
| ZFP36L2 | 190.5646969 | 0 | -3.60709915 |
| LMNA | 196.7504252 | 0 | -2.130513317 |
| HLA-DPA1 | 197.5397754 | 0 | -1.05237961 |
| MCL1 | 198.9639132 | 0 | -2.292744909 |
| FSCN1 | 199.4768549 | 0 | 3.695136134 |
| CD1E | 199.6159297 | 0 | 3.255245074 |
| ACTG1 | 201.4360517 | 0 | 1.125467359 |
| NR4A1 | 202.0224841 | 0 | -3.165992478 |
| NAMPT | 202.066571 | 0 | -3.780681494 |
| C15orf48 | 203.5104665 | 0 | -4.32011746 |
| HERPUD1 | 203.8307233 | 0 | -3.685453728 |
| SOD2 | 204.5403994 | 0 | -3.926774371 |
| SLC2A3 | 208.1971552 | 0 | -4.178821365 |
| NFKBIZ | 224.4032809 | 0 | -3.96241634 |
| NR4A2 | 227.0376514 | 0 | -5.08721977 |
| INSIG1 | 228.4095589 | 0 | -6.365644258 |
| HINT1 | 235.6419608 | 0 | 1.594884855 |
| HLA-DMA | 238.0321627 | 0 | -2.207676547 |
| CCL17 | 244.3610458 | 0 | 6.510499228 |
| GPR183 | 245.4565682 | 0 | -3.21411082 |
| LSP1 | 247.8496981 | 0 | 1.874529826 |
| SEC61B | 248.944535 | 0 | 2.241448536 |
| DUSP2 | 249.1874445 | 0 | -5.420716938 |
| FOSB | 249.445196 | 0 | -4.35048109 |
| ZFP36 | 253.6880357 | 0 | -2.348877937 |
| PLAUR | 264.1863727 | 0 | -7.138800886 |
| AIF1 | 287.5911495 | 0 | -4.011530948 |
| HLA-DRB5 | 290.7193113 | 0 | -3.464066074 |
| GSTP1 | 305.3102356 | 0 | 1.710888026 |
| FCER1G | 312.2430627 | 0 | -3.476998153 |
| JUNB | 316.3148505 | 0 | -3.068254259 |
| FTH1 | 357.6246959 | 0 | -2.1605632 |
| TIMP1 | 361.6395842 | 0 | -3.954875028 |
| LYZ | 377.6313062 | 0 | -2.908292078 |
| TXN | 415.1995561 | 0 | 3.563212427 |
| HLA-DRA | 418.5884853 | 0 | -1.433659259 |
| B2M | 452.9908118 | 0 | 1.160963365 |
| S100A4 | 481.7203447 | 0 | -2.518404479 |
| C5AR1 | 524.5067208 | 0 | -7.447454985 |
| NFKBIA | 561.8608256 | 0 | -4.10082338 |
| FTL | 626.6035916 | 0 | -2.625751757 |
| HLA-DRB1 | 647.6746507 | 0 | -1.914967047 |
| CST3 | 835.6309456 | 0 | -4.664231663 |

| **Table S7. Differentially Expressed Genes Between NS and KD Groups in DC Subsets** | | | | | |
| --- | --- | --- | --- | --- | --- |
| **p_val** | **log_2_FC** | **pct.1** | **pct.2** | **FDR** | **gene** |
| 6.84696739224691e-125 | -4.722955231 | 0.01 | 0.162 | 1.69003696142831e-120 | CCL3L1 |
| 1.42617899035721e-198 | -4.590671377 | 0.033 | 0.313 | 3.52023760189869e-194 | CCL4L2 |
| 6.93693628843103e-101 | -4.247595091 | 0.006 | 0.125 | 1.71224398407343e-96 | LRMDA |
| 4.78951493444557e-224 | -4.130733804 | 0.018 | 0.289 | 1.1821959712692e-219 | CCDC85B |
| 1.77329053138792e-270 | -3.84389162 | 0.036 | 0.392 | 4.37701301862481e-266 | CHCHD10 |
| 1.45972613770311e-189 | -3.817063213 | 0.02 | 0.264 | 3.60304202569258e-185 | C4orf48 |
| 9.05809762118724e-77 | -3.760569523 | 0.009 | 0.111 | 2.23581023583765e-72 | NDUFAF8 |
| 6.485499061728e-306 | -3.66962826 | 0.045 | 0.454 | 1.60081573340632e-301 | ATP5F1D |
| 8.54055483920868e-93 | -3.646008165 | 0.01 | 0.131 | 2.10806515096188e-88 | AL135905.2 |
| 1.45527942087327e-130 | -3.618485487 | 0.016 | 0.192 | 3.59206619454149e-126 | NENF |
| 6.73308885973236e-206 | -3.609184857 | 0.144 | 0.534 | 1.66192832324774e-201 | IL1B |
| 1.34603448696718e-200 | -3.554892149 | 0.053 | 0.371 | 3.3224169241811e-196 | NLRP3 |
| 1.73158504526018e-181 | -3.513689343 | 0.101 | 0.446 | 4.27407136721571e-177 | CXCL3 |
| 1.70673752684653e-118 | -3.507803453 | 0.017 | 0.183 | 4.21274023751529e-114 | TMEM160 |
| 3.99740262289204e-158 | -3.283991032 | 0.072 | 0.368 | 9.86678889408441e-154 | CFD |
| 4.18704530709393e-156 | -3.178339693 | 0.04 | 0.292 | 1.03348839315e-151 | PID1 |
| 1.09458332910668e-187 | -3.175951958 | 0.186 | 0.572 | 2.70176003123402e-183 | DUSP2 |
| 9.39533776907556e-122 | -3.131863829 | 0.117 | 0.396 | 2.31905122154092e-117 | RGCC |
| 1.46285578927465e-129 | -3.091717864 | 0.024 | 0.219 | 3.61076694466661e-125 | XIST |
| 5.22702224466058e-178 | -3.073852588 | 0.13 | 0.484 | 1.29018590064957e-173 | NFKBIZ |
| 1.91518568488343e-34 | -3.039780608 | 0.026 | 0.108 | 4.72725282599777e-30 | CXCL1 |
| 5.85338789951727e-71 | -3.032679551 | 0.016 | 0.131 | 1.44479173523785e-66 | ZNF503 |
| 1.92044170761692e-149 | -3.025811569 | 0.139 | 0.47 | 4.74022626691085e-145 | CXCL2 |
| 1.28153676613295e-197 | -3.017824041 | 0.159 | 0.554 | 3.16321719984596e-193 | ATF3 |
| 1.8171550969862e-210 | -2.984588561 | 0.445 | 0.814 | 4.48528392589103e-206 | NFKBIA |
| 3.19245456010454e-255 | -2.963265471 | 0.863 | 0.977 | 7.87993559070603e-251 | CST3 |
| 3.35879816182427e-177 | -2.957172238 | 0.223 | 0.608 | 8.29052150283084e-173 | INSIG1 |
| 1.6717030358532e-116 | -2.89734486 | 0.063 | 0.3 | 4.12626460339646e-112 | PPIF |
| 9.11000950113289e-113 | -2.881442016 | 0.029 | 0.215 | 2.24862364516463e-108 | FAAP20 |
| 2.36750560760578e-134 | -2.864381954 | 0.138 | 0.447 | 5.84371409125334e-130 | BCL2A1 |
| 1.78195681744723e-50 | -2.861156552 | 0.014 | 0.1 | 4.398404012505e-46 | FOSL1 |
| 8.90910826849486e-69 | -2.802810248 | 0.017 | 0.132 | 2.19903519391259e-64 | MSRA |
| 2.3287351147305e-199 | -2.75984856 | 0.109 | 0.486 | 5.74801688368929e-195 | NFKBID |
| 2.29805572392047e-58 | -2.75125749 | 0.027 | 0.145 | 5.67229094335291e-54 | KLF2 |
| 1.71955971371766e-146 | -2.750638244 | 0.065 | 0.343 | 4.2443892413693e-142 | CCL4 |
| 3.13400276030263e-48 | -2.673951763 | 0.015 | 0.101 | 7.73565901325499e-44 | ZNF511 |
| 1.83591012703913e-131 | -2.662172914 | 0.067 | 0.328 | 4.53157696657069e-127 | CDC42EP3 |
| 1.26647783142156e-88 | -2.636391506 | 0.03 | 0.192 | 3.12604723129785e-84 | MZT2B |
| 1.40730388679424e-45 | -2.609258328 | 0.018 | 0.105 | 3.47364818377423e-41 | RAB20 |
| 1.07143929465154e-82 | -2.584057125 | 0.037 | 0.199 | 2.6446336109884e-78 | KLF3 |
| 2.94872810964331e-98 | -2.568377588 | 0.065 | 0.281 | 7.27834559303258e-94 | CEBPD |
| 1.71178452910088e-68 | -2.559104681 | 0.032 | 0.171 | 4.2251977531797e-64 | ARHGAP6 |
| 1.15768261298528e-171 | -2.537448914 | 0.123 | 0.48 | 2.85750799363157e-167 | AP1S2 |
| 3.81472754175903e-92 | -2.52923184 | 0.038 | 0.217 | 9.41589199132381e-88 | TLR2 |
| 3.70219730780931e-191 | -2.465365142 | 0.13 | 0.517 | 9.13813361486571e-187 | RNF130 |
| 1.00534650702406e-104 | -2.460672706 | 0.055 | 0.27 | 2.4814967832875e-100 | PLBD1 |
| 6.08139144775028e-149 | -2.456806319 | 0.223 | 0.575 | 1.5010698510482e-144 | TNFAIP3 |
| 4.03660167106466e-151 | -2.450821218 | 0.092 | 0.405 | 9.96354390468891e-147 | MAP1LC3B |
| 5.60594249971828e-63 | -2.446194039 | 0.029 | 0.156 | 1.38371478720546e-58 | ARHGAP24 |
| 4.96398389801157e-121 | -2.423102564 | 0.132 | 0.426 | 1.2252601455462e-116 | CD55 |
| 2.56859011264095e-84 | -2.404573296 | 0.065 | 0.262 | 6.34005097503166e-80 | ARL5B |
| 6.18449712605761e-65 | -2.404244925 | 0.03 | 0.161 | 1.5265194256248e-60 | HEBP1 |
| 2.1678589388048e-213 | -2.403589493 | 0.378 | 0.763 | 5.3509262186519e-209 | ITM2B |
| 6.8855096928593e-139 | -2.402912061 | 0.104 | 0.407 | 1.69955035748846e-134 | CSRNP1 |
| 3.69711679536288e-40 | -2.393972758 | 0.031 | 0.128 | 9.12559338599419e-36 | LYVE1 |
| 2.38699649702463e-90 | -2.380217489 | 0.051 | 0.242 | 5.89182345360588e-86 | HDAC9 |
| 1.62610871590328e-129 | -2.361823084 | 0.102 | 0.398 | 4.01372414346407e-125 | PAPSS2 |
| 1.6615490178707e-39 | -2.354387538 | 0.035 | 0.135 | 4.10120144081025e-35 | TSPYL2 |
| 1.28822057515888e-136 | -2.344263017 | 0.204 | 0.537 | 3.17971484566465e-132 | ICAM1 |
| 9.34288233567856e-56 | -2.344209542 | 0.037 | 0.164 | 2.30610364691554e-51 | PDGFB |
| 3.11337325318102e-47 | -2.335409351 | 0.025 | 0.125 | 7.6847392008267e-43 | AKR7A2 |
| 4.28647184554468e-141 | -2.328723588 | 0.169 | 0.514 | 1.05802984563579e-136 | CXCL8 |
| 1.70327865559714e-87 | -2.32133838 | 0.08 | 0.296 | 4.20420270561041e-83 | EIF4A3 |
| 1.16640557287909e-151 | -2.318400664 | 0.288 | 0.643 | 2.87903887553745e-147 | IER3 |
| 1.36739445551726e-126 | -2.318298082 | 0.075 | 0.343 | 3.37513973455325e-122 | PRELID1 |
| 3.78226285316836e-108 | -2.294147183 | 0.095 | 0.351 | 9.33575940047546e-104 | STX11 |
| 6.00730974471122e-140 | -2.292455692 | 0.093 | 0.395 | 1.48278426428707e-135 | VSIG4 |
| 3.78075793727675e-54 | -2.290431289 | 0.034 | 0.155 | 9.33204481658019e-50 | NME4 |
| 8.88132622890594e-79 | -2.281300277 | 0.052 | 0.23 | 2.19217775308085e-74 | VPS37B |
| 4.32320707996863e-76 | -2.27403049 | 0.062 | 0.246 | 1.06709720354866e-71 | CCL3 |
| 2.88157886908346e-33 | -2.244973525 | 0.028 | 0.111 | 7.11260112255871e-29 | CDC42EP2 |
| 8.77716458531214e-94 | -2.244297501 | 0.072 | 0.291 | 2.16646753459259e-89 | MAP2K3 |
| 3.33436800582643e-46 | -2.236973347 | 0.028 | 0.13 | 8.23022054878138e-42 | CPED1 |
| 4.56950256328476e-71 | -2.223330341 | 0.053 | 0.22 | 1.12789031769558e-66 | CEBPB |
| 7.66825312180399e-38 | -2.218357669 | 0.026 | 0.115 | 1.89275491805488e-33 | PER3 |
| 6.25930892691656e-34 | -2.217499766 | 0.027 | 0.11 | 1.54498522243081e-29 | CCRL2 |
| 1.74261348506012e-43 | -2.208199358 | 0.028 | 0.126 | 4.30129286517389e-39 | MZT2A |
| 2.43014482230924e-106 | -2.207289323 | 0.126 | 0.398 | 5.99832646490591e-102 | MMP9 |
| 2.80258134098162e-56 | -2.206108671 | 0.034 | 0.159 | 6.91761152394494e-52 | GRASP |
| 1.14187218496249e-71 | -2.200728486 | 0.049 | 0.214 | 2.81848311414291e-67 | MCUR1 |
| 1.46122999140691e-47 | -2.195274056 | 0.033 | 0.145 | 3.60675398778968e-43 | ADAM28 |
| 1.06695370100642e-113 | -2.191202656 | 0.158 | 0.455 | 2.63356182019414e-109 | KLF4 |
| 1.25354009949861e-45 | -2.189893501 | 0.11 | 0.268 | 3.09411302759243e-41 | G0S2 |
| 7.20687501997917e-107 | -2.185146483 | 0.115 | 0.381 | 1.77887296118146e-102 | ZC3H12A |
| 8.06243392170988e-55 | -2.179332512 | 0.033 | 0.156 | 1.99005056489565e-50 | SURF1 |
| 6.20506281802967e-142 | -2.176522882 | 0.106 | 0.421 | 1.53159565537426e-137 | CRTAP |
| 8.53907986576162e-49 | -2.176165233 | 0.03 | 0.14 | 2.10770108326594e-44 | SNHG7 |
| 4.6984231697856e-101 | -2.170584897 | 0.072 | 0.304 | 1.15971179099818e-96 | TOB2 |
| 5.62788105293607e-69 | -2.163042575 | 0.044 | 0.2 | 1.38912988029621e-64 | CMTM7 |
| 3.38658677768843e-51 | -2.155344689 | 0.034 | 0.152 | 8.35911214336835e-47 | DEPTOR |
| 1.27064108914745e-115 | -2.152927765 | 0.1 | 0.375 | 3.13632340034264e-111 | IL13RA1 |
| 5.07877682689749e-53 | -2.150452535 | 0.039 | 0.165 | 1.25359448418311e-48 | PTGER3 |
| 1.11989843976994e-53 | -2.14852532 | 0.038 | 0.163 | 2.76424531888414e-49 | UBE2D1 |
| 7.71428936356062e-66 | -2.14767345 | 0.088 | 0.274 | 1.90411804360767e-61 | FILIP1L |
| 9.23885412725715e-64 | -2.143162023 | 0.044 | 0.193 | 2.28042636423088e-59 | CYC1 |
| 7.81261188437783e-78 | -2.131369416 | 0.077 | 0.276 | 1.92838699142098e-73 | PIM3 |
| 4.50306773397187e-51 | -2.125212576 | 0.047 | 0.178 | 1.11149220877628e-46 | IGFBP7 |
| 5.3344193550957e-109 | -2.120902143 | 0.143 | 0.427 | 1.31669472941827e-104 | KDM6B |
| 5.66291717142113e-73 | -2.114282553 | 0.045 | 0.208 | 1.39777784542188e-68 | CALHM6 |
| 3.46218703115545e-64 | -2.105152041 | 0.054 | 0.213 | 8.545716249001e-60 | BCL3 |
| 4.75368483441469e-31 | -2.099180793 | 0.032 | 0.116 | 1.17335202767858e-26 | EHD1 |
| 1.76524262191638e-227 | -2.097235753 | 0.943 | 0.994 | 4.3571483636762e-223 | FTH1 |
| 3.08374648588288e-34 | -2.091060842 | 0.03 | 0.117 | 7.61161145110471e-30 | STARD8 |
| 5.0354147265644e-168 | -2.090292055 | 0.343 | 0.715 | 1.24289141695789e-163 | FOSB |
| 1.707618269219e-85 | -2.07583543 | 0.419 | 0.619 | 4.21491417391326e-81 | COTL1 |
| 2.0832550672573e-107 | -2.072525161 | 0.152 | 0.439 | 5.14209848251119e-103 | NR4A3 |
| 1.0617768388969e-87 | -2.069648003 | 0.123 | 0.369 | 2.62078377144921e-83 | TAGAP |
| 3.69885967201301e-85 | -2.065856502 | 0.09 | 0.31 | 9.12989532842972e-81 | TIPARP |
| 3.28920434340865e-34 | -2.065424105 | 0.045 | 0.145 | 8.11874308083558e-30 | METTL21A |
| 1.0170203594077e-76 | -2.064347466 | 0.07 | 0.264 | 2.51031135312603e-72 | RCSD1 |
| 2.01752475740332e-34 | -2.062636287 | 0.028 | 0.113 | 4.97985635869861e-30 | BCL7B |
| 6.47070034670051e-77 | -2.057694455 | 0.217 | 0.459 | 1.59716296657609e-72 | SELENOP |
| 5.00852616833294e-61 | -2.055576319 | 0.058 | 0.215 | 1.23625451412962e-56 | GAS7 |
| 1.87409683810593e-68 | -2.053997369 | 0.08 | 0.268 | 4.62583322549686e-64 | PTGS2 |
| 1.00504138166469e-55 | -2.051968246 | 0.074 | 0.234 | 2.48074364236296e-51 | PFKFB3 |
| 1.18151206827293e-140 | -2.048912006 | 0.158 | 0.503 | 2.91632623811807e-136 | CD302 |
| 1.78196230641044e-56 | -2.047989511 | 0.067 | 0.225 | 4.39841756091289e-52 | ANKRD37 |
| 5.33871328749153e-137 | -2.026103296 | 0.226 | 0.571 | 1.31775460075153e-132 | FCGR2B |
| 4.88089075861236e-90 | -2.019060409 | 0.103 | 0.341 | 1.20475026594829e-85 | GAS6 |
| 1.99683447889532e-126 | -2.015726088 | 0.209 | 0.544 | 4.92878654425731e-122 | ZFP36L2 |
| 9.68328898736643e-45 | -2.014390985 | 0.023 | 0.117 | 2.39012622075165e-40 | CXCL12 |
| 5.85616343283777e-38 | -2.014291895 | 0.03 | 0.123 | 1.44547682012735e-33 | MPST |
| 9.43856376006884e-301 | -2.001458194 | 0.597 | 0.963 | 2.32972069289779e-296 | HLA-DRB5 |
| 4.47016926324775e-88 | -1.996675935 | 0.107 | 0.345 | 1.10337187924744e-83 | MAP3K8 |
| 1.25295893281273e-59 | -1.992650224 | 0.069 | 0.233 | 3.09267853386166e-55 | VEGFA |
| 6.1493789758824e-74 | -1.988172834 | 0.062 | 0.244 | 1.51785121261705e-69 | RBM17 |
| 1.50152331457104e-80 | -1.981894891 | 0.078 | 0.284 | 3.7062099973557e-76 | SH3BP5 |
| 2.26067208081923e-33 | -1.973594252 | 0.029 | 0.115 | 5.58001689708611e-29 | TBC1D7 |
| 9.42655882739116e-35 | -1.972136248 | 0.027 | 0.112 | 2.32675751536496e-30 | VPS4A |
| 1.9220695720901e-98 | -1.966167073 | 0.116 | 0.376 | 4.74424432478999e-94 | RASGEF1B |
| 4.34111566842568e-88 | -1.963695791 | 0.104 | 0.341 | 1.07151758043751e-83 | XBP1 |
| 1.57056152353731e-36 | -1.955890918 | 0.038 | 0.136 | 3.87661700854714e-32 | NLRP1 |
| 5.50973736160774e-53 | -1.954013884 | 0.048 | 0.185 | 1.35996847296564e-48 | BAG1 |
| 2.16726703010819e-103 | -1.950039093 | 0.125 | 0.398 | 5.34946521041606e-99 | COX5A |
| 4.6601980653954e-61 | -1.93560945 | 0.055 | 0.21 | 1.15027668848155e-56 | PARVB |
| 9.6133894834184e-48 | -1.933153326 | 0.053 | 0.184 | 2.37287292619216e-43 | TOR3A |
| 2.03046227537296e-56 | -1.932952268 | 0.048 | 0.191 | 5.01179003430307e-52 | MCUB |
| 1.50045771106892e-42 | -1.9328611 | 0.036 | 0.143 | 3.70357976823142e-38 | GPR132 |
| 5.77261692799375e-84 | -1.923671902 | 0.158 | 0.407 | 1.4248550363367e-79 | PPP1R10 |
| 4.59123840613666e-48 | -1.922282525 | 0.052 | 0.184 | 1.13325537578671e-43 | PER1 |
| 2.73003530065176e-52 | -1.919064261 | 0.049 | 0.186 | 6.73854613259874e-48 | GNPTG |
| 2.05060244298102e-154 | -1.918716742 | 0.334 | 0.698 | 5.06150201001005e-150 | NR4A1 |
| 3.85985923479717e-52 | -1.916628431 | 0.062 | 0.209 | 9.52729054924986e-48 | NCF1 |
| 1.27096608878365e-35 | -1.911549791 | 0.045 | 0.148 | 3.13712559694468e-31 | NSUN6 |
| 2.27419988107818e-48 | -1.908154031 | 0.047 | 0.174 | 5.61340756646526e-44 | ME1 |
| 5.02181136308457e-34 | -1.905767439 | 0.041 | 0.138 | 1.23953369875016e-29 | RALGAPA1 |
| 1.32059794256942e-59 | -1.898715816 | 0.125 | 0.32 | 3.25963190164409e-55 | C5AR1 |
| 2.83983803012014e-42 | -1.8942718 | 0.045 | 0.161 | 7.00957220974554e-38 | AFF3 |
| 9.94401915954232e-25 | -1.891904785 | 0.032 | 0.104 | 2.45448224914983e-20 | CCDC170 |
| 1.90968997079384e-60 | -1.884319019 | 0.082 | 0.258 | 4.71368775491043e-56 | GYPC |
| 7.82021744480069e-148 | -1.877851904 | 0.346 | 0.7 | 1.93026427190015e-143 | NR4A2 |
| 3.05471355762441e-49 | -1.871333942 | 0.047 | 0.177 | 7.53994947428433e-45 | EPN1 |
| 3.23347013680158e-144 | -1.870685947 | 0.4 | 0.729 | 7.98117433866734e-140 | CD83 |
| 1.64906720526537e-62 | -1.868486423 | 0.083 | 0.264 | 4.07039258275651e-58 | RILPL2 |
| 4.43558505988729e-32 | -1.868445812 | 0.032 | 0.119 | 1.09483546033198e-27 | YPEL2 |
| 2.31407643453149e-39 | -1.859963749 | 0.048 | 0.161 | 5.71183486335409e-35 | PLD4 |
| 4.6978605333239e-93 | -1.852847124 | 0.121 | 0.376 | 1.15957291544034e-88 | PGLS |
| 6.82801705676908e-94 | -1.844930958 | 0.127 | 0.393 | 1.68535945012231e-89 | CD163 |
| 1.36535581848802e-119 | -1.842081247 | 0.197 | 0.518 | 3.37010776677397e-115 | GRB2 |
| 3.65949180417112e-98 | -1.841958808 | 0.173 | 0.455 | 9.03272362023559e-94 | RNASET2 |
| 1.2285019371026e-80 | -1.840049687 | 0.191 | 0.445 | 3.03231133135034e-76 | CKS2 |
| 1.17477524120675e-31 | -1.826816097 | 0.031 | 0.115 | 2.89969772787062e-27 | RELB |
| 1.11664198194847e-59 | -1.825684715 | 0.094 | 0.275 | 2.7562074040434e-55 | ETS2 |
| 7.63890560309997e-31 | -1.82307465 | 0.033 | 0.119 | 1.88551107001317e-26 | PSIP1 |
| 2.39093307133742e-32 | -1.822941008 | 0.043 | 0.139 | 5.90154009998216e-28 | PRMT9 |
| 2.70157674903495e-92 | -1.821053955 | 0.182 | 0.464 | 6.66830188964296e-88 | CD14 |
| 6.72946478014297e-27 | -1.8147565 | 0.043 | 0.128 | 1.66103379168269e-22 | PXDC1 |
| 4.91262031292804e-46 | -1.814471605 | 0.048 | 0.173 | 1.21258207184003e-41 | NT5C |
| 1.37605597082205e-48 | -1.811130565 | 0.054 | 0.19 | 3.39651895278007e-44 | ABHD12 |
| 2.86678244894158e-79 | -1.80871085 | 0.067 | 0.265 | 7.07607911872251e-75 | MSR1 |
| 6.56947683513491e-65 | -1.805182914 | 0.086 | 0.274 | 1.62154396721635e-60 | B4GALT1 |
| 3.35535441433798e-60 | -1.800567347 | 0.101 | 0.288 | 8.28202130091043e-56 | AVPI1 |
| 3.86570693244907e-31 | -1.788072396 | 0.039 | 0.129 | 9.54172442136404e-27 | TSPAN3 |
| 4.78655619385307e-85 | -1.78781718 | 0.131 | 0.379 | 1.18146566532875e-80 | LYN |
| 2.23085840956604e-78 | -1.784578966 | 0.489 | 0.676 | 5.50642781233187e-74 | PLAUR |
| 4.54284603541028e-115 | -1.784036562 | 0.267 | 0.594 | 1.12131068692032e-110 | SQSTM1 |
| 1.86724947123958e-44 | -1.774090378 | 0.049 | 0.173 | 4.60893186986065e-40 | METTL9 |
| 2.44203420728998e-62 | -1.769547794 | 0.113 | 0.309 | 6.02767303385387e-58 | PDE4B |
| 2.92563991300242e-30 | -1.767624281 | 0.049 | 0.146 | 7.22135699726389e-26 | FOXO3 |
| 8.29038645436363e-88 | -1.766388526 | 0.137 | 0.393 | 2.04631608853058e-83 | ATP1B1 |
| 5.24542397143291e-48 | -1.763306696 | 0.114 | 0.283 | 1.29472799886878e-43 | SLC40A1 |
| 4.49636129103393e-37 | -1.757375599 | 0.048 | 0.157 | 1.1098368574659e-32 | JOSD1 |
| 9.65534369365962e-62 | -1.755997867 | 0.087 | 0.269 | 2.383228483906e-57 | AC007384.1 |
| 1.77669124026704e-77 | -1.754026677 | 0.102 | 0.323 | 4.38540698835112e-73 | TRAPPC5 |
| 2.03457269974598e-28 | -1.746855976 | 0.055 | 0.153 | 5.02193579478301e-24 | RBBP8 |
| 1.75777158333612e-69 | -1.746831921 | 0.283 | 0.521 | 4.33870759914854e-65 | HERPUD1 |
| 4.92833462213648e-50 | -1.746020841 | 0.067 | 0.216 | 1.21646083478195e-45 | EPS8 |
| 4.31752071906172e-42 | -1.743193215 | 0.058 | 0.184 | 1.065693639086e-37 | RTN1 |
| 1.1589169138723e-133 | -1.736092776 | 0.287 | 0.641 | 2.860554618511e-129 | REL |
| 1.66333097986784e-31 | -1.734156061 | 0.039 | 0.13 | 4.10559985760778e-27 | NBPF14 |
| 1.14618847597177e-19 | -1.728706256 | 0.092 | 0.183 | 2.82913701524113e-15 | MMP19 |
| 8.38881697724034e-48 | -1.726443739 | 0.067 | 0.21 | 2.07061169449223e-43 | POU2F2 |
| 4.3125226364395e-113 | -1.717211249 | 0.168 | 0.482 | 1.06445996235236e-108 | GNB2 |
| 1.98889801767534e-81 | -1.716398732 | 0.13 | 0.374 | 4.90919697702805e-77 | ACAP2 |
| 2.57226320276407e-28 | -1.714117666 | 0.037 | 0.121 | 6.34911726338256e-24 | HPCAL1 |
| 4.69735114736058e-77 | -1.708065282 | 0.155 | 0.399 | 1.15944718370301e-72 | PTMS |
| 2.25666451581979e-75 | -1.706490398 | 0.134 | 0.367 | 5.57012502439799e-71 | NCF4 |
| 1.3763685061166e-64 | -1.706381709 | 0.088 | 0.278 | 3.39729038364762e-60 | CMTM3 |
| 2.18172164860357e-36 | -1.703428965 | 0.047 | 0.154 | 5.38514354524819e-32 | AGPAT2 |
| 5.75048071436553e-20 | -1.698671204 | 0.036 | 0.103 | 1.41939115472684e-15 | TNS1 |
| 1.48131590242803e-22 | -1.697180817 | 0.032 | 0.101 | 3.65633204196312e-18 | ZFAND3 |
| 2.1525674158346e-70 | -1.696041877 | 0.123 | 0.343 | 5.31318215250454e-66 | ANP32B |
| 1.80474332037418e-94 | -1.694194966 | 0.243 | 0.531 | 4.45464793767959e-90 | NFE2L2 |
| 1.36051937028142e-41 | -1.693633931 | 0.067 | 0.198 | 3.35816996166564e-37 | JMJD6 |
| 1.50029488478277e-42 | -1.68415296 | 0.055 | 0.18 | 3.70317786410931e-38 | GADD45GIP1 |
| 9.1972516415487e-23 | -1.68415296 | 0.045 | 0.125 | 2.27015762268347e-18 | GPR34 |
| 3.01813908105997e-30 | -1.683034398 | 0.059 | 0.163 | 7.44967269378033e-26 | WWP1 |
| 5.40850361432752e-85 | -1.681118161 | 0.159 | 0.423 | 1.33498094712446e-80 | ZNF331 |
| 9.214531746807e-44 | -1.677631241 | 0.069 | 0.206 | 2.27442287106437e-39 | RAB32 |
| 5.82009788478515e-27 | -1.673593997 | 0.034 | 0.113 | 1.43657476090152e-22 | PITHD1 |
| 2.9020473059395e-59 | -1.668995718 | 0.109 | 0.301 | 7.16312336525047e-55 | SPAG9 |
| 1.29912595534699e-36 | -1.66812619 | 0.067 | 0.189 | 3.20663259558297e-32 | A2M |
| 1.87142381225135e-167 | -1.664665691 | 0.688 | 0.875 | 4.61923539578e-163 | RPS2 |
| 1.27567562904413e-64 | -1.657968895 | 0.4 | 0.597 | 3.14875015516963e-60 | FCER1A |
| 1.143886071373e-21 | -1.653621349 | 0.036 | 0.107 | 2.82345398996998e-17 | BTG3 |
| 3.1328409322703e-10 | -1.651707837 | 0.095 | 0.158 | 7.73279127312278e-06 | TWISTNB |
| 7.34997425068306e-18 | -1.650906964 | 0.044 | 0.111 | 1.8141941442961e-13 | ITSN1 |
| 5.41263177770531e-26 | -1.649822458 | 0.033 | 0.11 | 1.335999901691e-21 | CNRIP1 |
| 3.66429049987604e-39 | -1.648271216 | 0.086 | 0.224 | 9.04456824084404e-35 | MAFF |
| 1.00730111238061e-39 | -1.647364482 | 0.056 | 0.178 | 2.48632133568906e-35 | RAB4A |
| 3.55995713039204e-81 | -1.643568651 | 0.425 | 0.65 | 8.78704218494668e-77 | GPR183 |
| 3.31890120430145e-39 | -1.63758973 | 0.111 | 0.261 | 8.19204384257728e-35 | TRAF1 |
| 1.32578407300859e-29 | -1.636658518 | 0.043 | 0.134 | 3.2724328274071e-25 | FAM174C |
| 2.79443980539484e-101 | -1.634709468 | 0.176 | 0.478 | 6.8975157716561e-97 | MEF2C |
| 1.00448760791566e-31 | -1.633755437 | 0.095 | 0.222 | 2.47937676261823e-27 | TNF |
| 7.86702835622342e-25 | -1.630454703 | 0.032 | 0.105 | 1.94181860916663e-20 | CAMK1 |
| 1.1374022245608e-22 | -1.629168305 | 0.058 | 0.145 | 2.80744991088343e-18 | SLFN11 |
| 2.0527455563877e-21 | -1.626522806 | 0.033 | 0.1 | 5.06679185683175e-17 | MEPCE |
| 1.69494546498011e-23 | -1.61605099 | 0.033 | 0.104 | 4.1836338912104e-19 | OAS1 |
| 1.59860884665929e-89 | -1.615807072 | 0.206 | 0.488 | 3.94584621620914e-85 | SERP1 |
| 4.49535846051179e-35 | -1.615511564 | 0.055 | 0.166 | 1.10958932880812e-30 | PIK3R1 |
| 1.01010778493838e-40 | -1.608357453 | 0.063 | 0.192 | 2.4932490455634e-36 | FAM50A |
| 3.26503310345076e-23 | -1.607284197 | 0.038 | 0.113 | 8.0590812092475e-19 | MMP24OS |
| 1.02633322616735e-54 | -1.605643714 | 0.097 | 0.273 | 2.53329830214887e-50 | GFRA2 |
| 1.84566605671346e-47 | -1.604722308 | 0.113 | 0.283 | 4.55565752778583e-43 | TNFRSF1B |
| 5.6933034001733e-24 | -1.601860751 | 0.032 | 0.103 | 1.40527807826477e-19 | PDCD2 |
| 3.56707194196202e-37 | -1.600028582 | 0.064 | 0.187 | 8.80460367434485e-33 | IFNGR2 |
| 3.73727959677721e-43 | -1.599664311 | 0.071 | 0.209 | 9.22472722872519e-39 | SMCHD1 |
| 1.17181177466162e-32 | -1.574253295 | 0.05 | 0.154 | 2.89238300339729e-28 | AOAH |
| 3.1022598118319e-30 | -1.574201651 | 0.053 | 0.154 | 7.65730789354468e-26 | NFKBIE |
| 8.06690592941117e-24 | -1.566346986 | 0.042 | 0.122 | 1.99115439055656e-19 | PIM2 |
| 7.10131815331146e-29 | -1.565788448 | 0.057 | 0.158 | 1.75281835978187e-24 | GEM |
| 1.55735764465247e-52 | -1.562689166 | 0.301 | 0.501 | 3.84402587429569e-48 | IL1R2 |
| 1.54820063172196e-162 | -1.561205247 | 0.662 | 0.858 | 3.82142361927932e-158 | PFN1 |
| 4.16775986383692e-29 | -1.549623628 | 0.051 | 0.148 | 1.02872816719087e-24 | PNPLA2 |
| 8.9445009988854e-21 | -1.549623628 | 0.037 | 0.106 | 2.20777118155488e-16 | SNX18 |
| 3.70595199182228e-24 | -1.546114843 | 0.045 | 0.127 | 9.14740130141494e-20 | TPRA1 |
| 1.00769055389996e-22 | -1.545736706 | 0.037 | 0.111 | 2.48728259419128e-18 | PIK3AP1 |
| 4.88079438381246e-38 | -1.543954878 | 0.071 | 0.2 | 1.20472647775643e-33 | RIPK2 |
| 6.49240593932158e-32 | -1.542568871 | 0.059 | 0.167 | 1.60252055800275e-27 | CRYL1 |
| 3.90009526228143e-29 | -1.535817829 | 0.044 | 0.135 | 9.62660513588925e-25 | UBXN11 |
| 6.65534908807576e-34 | -1.535603158 | 0.088 | 0.218 | 1.64273981540974e-29 | FCN1 |
| 7.60742068199894e-22 | -1.535204569 | 0.033 | 0.102 | 1.8777396469378e-17 | HSF1 |
| 2.49620756666459e-19 | -1.534785119 | 0.039 | 0.107 | 6.1613891367982e-15 | FLT3 |
| 4.89047296027688e-28 | -1.534673287 | 0.053 | 0.149 | 1.20711544078514e-23 | IL17RA |
| 1.10791813554892e-33 | -1.533682085 | 0.066 | 0.183 | 2.73467433397539e-29 | CCDC50 |
| 5.08762828413748e-122 | -1.530893749 | 0.373 | 0.679 | 1.25577928937365e-117 | RAC1 |
| 6.36790510486737e-24 | -1.526635968 | 0.049 | 0.133 | 1.57179001703441e-19 | AC004687.1 |
| 1.25821794372633e-23 | -1.523896803 | 0.041 | 0.12 | 3.1056593504997e-19 | UROS |
| 2.28916185612686e-25 | -1.521258737 | 0.074 | 0.175 | 5.65033820947792e-21 | H1F0 |
| 1.93485821313126e-22 | -1.521103588 | 0.042 | 0.119 | 4.77581052747188e-18 | SLBP |
| 3.66988112257593e-25 | -1.520477283 | 0.043 | 0.126 | 9.05836757485417e-21 | ARRDC2 |
| 1.04078941533421e-22 | -1.517733041 | 0.047 | 0.127 | 2.56898051386943e-18 | ZBTB1 |
| 1.091887419427e-19 | -1.508059818 | 0.09 | 0.183 | 2.69510571737168e-15 | EGR1 |
| 5.73009929783949e-38 | -1.503668995 | 0.064 | 0.189 | 1.41436040968572e-33 | ACAA1 |
| 2.63111383678181e-32 | -1.502317914 | 0.07 | 0.186 | 6.49437828332854e-28 | MTMR14 |
| 2.23533299699754e-22 | -1.498823621 | 0.044 | 0.122 | 5.51747243648902e-18 | RC3H1 |
| 1.15660002035382e-38 | -1.498656855 | 0.098 | 0.243 | 2.85483583023933e-34 | CCND2 |
| 9.05944119207215e-50 | -1.497302593 | 0.138 | 0.323 | 2.23614186943917e-45 | PHACTR1 |
| 8.87707307056944e-67 | -1.497095449 | 0.206 | 0.446 | 2.19112794600866e-62 | NINJ1 |
| 2.2916809083687e-109 | -1.496979671 | 0.594 | 0.815 | 5.65655598612647e-105 | JUNB |
| 4.09933697143706e-47 | -1.496398689 | 0.087 | 0.245 | 1.01183934465981e-42 | MTCH1 |
| 1.74631472321527e-71 | -1.49605399 | 0.361 | 0.599 | 4.31042863131225e-67 | SELENOK |
| 1.08770057826577e-91 | -1.492227954 | 0.463 | 0.718 | 2.68477133733341e-87 | PLEK |
| 5.28858394521453e-22 | -1.488432654 | 0.038 | 0.111 | 1.3053811751973e-17 | IGFBP5 |
| 5.5607322825884e-52 | -1.485000764 | 0.2 | 0.403 | 1.37255554931129e-47 | CREM |
| 6.68354218204409e-25 | -1.483106698 | 0.045 | 0.129 | 1.64969871679394e-20 | NAPRT |
| 5.71115550925161e-45 | -1.479589382 | 0.109 | 0.274 | 1.40968451434858e-40 | H2AFV |
| 2.05614926388086e-22 | -1.475005587 | 0.045 | 0.124 | 5.07519322803712e-18 | RAB5IF |
| 9.38887212876883e-32 | -1.474707922 | 0.061 | 0.17 | 2.31745530754401e-27 | PRKCD |
| 7.77828727037969e-23 | -1.472467733 | 0.046 | 0.126 | 1.91991464694782e-18 | RNF24 |
| 4.96594069356067e-31 | -1.471687491 | 0.081 | 0.199 | 1.22574314139158e-26 | DIPK2A |
| 2.1317562615568e-29 | -1.468703633 | 0.075 | 0.188 | 5.26181398040065e-25 | SNX9 |
| 4.14361981737865e-22 | -1.468235302 | 0.04 | 0.115 | 1.02276967952357e-17 | AAK1 |
| 2.72362653269609e-65 | -1.46811737 | 0.173 | 0.405 | 6.72272737065376e-61 | FRMD4B |
| 3.06658726418615e-40 | -1.462160787 | 0.096 | 0.245 | 7.56925734419067e-36 | CREG1 |
| 4.34977907017475e-34 | -1.462160787 | 0.063 | 0.179 | 1.07365596789123e-29 | ARL5A |
| 6.39738348753764e-22 | -1.462160787 | 0.038 | 0.111 | 1.57906616622892e-17 | RNF166 |
| 4.23333736826555e-49 | -1.455854898 | 0.247 | 0.449 | 1.04491466260898e-44 | SOD2 |
| 7.17932587119137e-20 | -1.4521271 | 0.037 | 0.105 | 1.77207300478617e-15 | REEP3 |
| 4.33681638025968e-115 | -1.449469067 | 0.501 | 0.774 | 1.0704563871395e-110 | GADD45B |
| 3.57114998407067e-22 | -1.445286969 | 0.054 | 0.138 | 8.81466950568163e-18 | GPCPD1 |
| 8.06945578677814e-22 | -1.444187353 | 0.055 | 0.138 | 1.99178377185045e-17 | DDIT3 |
| 1.68170462817121e-24 | -1.44401344 | 0.05 | 0.136 | 4.150951533715e-20 | AKIRIN1 |
| 2.27726069056231e-90 | -1.43967404 | 0.371 | 0.644 | 5.62096256251494e-86 | DNAJA1 |
| 3.69477914001382e-23 | -1.43729068 | 0.051 | 0.135 | 9.11982335129611e-19 | TNFRSF10B |
| 1.32905036819185e-36 | -1.437148899 | 0.078 | 0.209 | 3.28049502380794e-32 | RASSF5 |
| 6.43518276000816e-42 | -1.432953608 | 0.081 | 0.226 | 1.58839616065281e-37 | ARHGAP4 |
| 1.86118210960618e-26 | -1.425774498 | 0.048 | 0.137 | 4.59395580114092e-22 | STUB1 |
| 9.58034027304082e-25 | -1.425376201 | 0.05 | 0.138 | 2.36471538959467e-20 | HMG20B |
| 5.17358017232602e-26 | -1.424264179 | 0.073 | 0.177 | 1.27699479393523e-21 | ARL4A |
| 4.03573014451856e-63 | -1.424243746 | 0.178 | 0.415 | 9.96139271571517e-59 | FOLR2 |
| 5.31344659363474e-46 | -1.419308764 | 0.163 | 0.348 | 1.31151802270686e-41 | SERTAD1 |
| 2.43636992690752e-45 | -1.41561046 | 0.107 | 0.272 | 6.01369189058582e-41 | NRROS |
| 4.04548197856183e-19 | -1.414291567 | 0.043 | 0.114 | 9.98546316768417e-15 | ST6GAL1 |
| 7.11348938081429e-40 | -1.413320184 | 0.16 | 0.329 | 1.75582258386639e-35 | STAB1 |
| 3.95564091180304e-24 | -1.41283828 | 0.073 | 0.172 | 9.76370846260344e-20 | LRP1 |
| 2.98823105469763e-49 | -1.412263293 | 0.141 | 0.329 | 7.37585071231015e-45 | ITSN2 |
| 4.15272974331982e-39 | -1.412120105 | 0.118 | 0.273 | 1.02501828254363e-34 | IRF1 |
| 3.05558556131555e-29 | -1.408935848 | 0.07 | 0.179 | 7.54210184099518e-25 | RANBP1 |
| 6.48780053704536e-73 | -1.40777715 | 0.301 | 0.568 | 1.60138380655891e-68 | BTG2 |
| 1.00813312432538e-75 | -1.40322135 | 0.203 | 0.468 | 2.48837499077233e-71 | IL10RA |
| 1.25942604987118e-46 | -1.401906576 | 0.149 | 0.334 | 3.10864131889703e-42 | AC020916.1 |
| 3.19804288530167e-64 | -1.400274988 | 0.178 | 0.41 | 7.89372925379012e-60 | JAK1 |
| 3.27957461921937e-23 | -1.399982898 | 0.061 | 0.152 | 8.09497403261917e-19 | FAM53C |
| 4.38797304229874e-19 | -1.399063952 | 0.04 | 0.108 | 1.0830833860306e-14 | GPR155 |
| 8.0109201876307e-36 | -1.389966962 | 0.088 | 0.223 | 1.97733542991289e-31 | NBPF26 |
| 2.15680733608429e-18 | -1.387803677 | 0.038 | 0.104 | 5.32364754765684e-14 | BIN1 |
| 5.70106994093632e-44 | -1.385236811 | 0.151 | 0.33 | 1.40719509352131e-39 | CYBB |
| 2.19604005794888e-38 | -1.380239367 | 0.143 | 0.304 | 5.42048567503523e-34 | GK |
| 2.36754893868972e-17 | -1.379698627 | 0.039 | 0.102 | 5.84382104536783e-13 | DPEP2 |
| 1.80330897456546e-30 | -1.376705483 | 0.063 | 0.172 | 4.45110754191993e-26 | GIMAP4 |
| 1.66204849499979e-41 | -1.373466673 | 0.106 | 0.263 | 4.10243430020798e-37 | FOSL2 |
| 1.49555385403501e-30 | -1.369198215 | 0.09 | 0.213 | 3.69147557791462e-26 | PPP1R15B |
| 5.01721967571479e-22 | -1.366970322 | 0.054 | 0.138 | 1.23840033255668e-17 | WAC-AS1 |
| 4.23207321357363e-24 | -1.360450742 | 0.056 | 0.146 | 1.04460263130638e-19 | SLC7A7 |
| 3.27007309340569e-48 | -1.358279643 | 0.153 | 0.342 | 8.07152141645326e-44 | ETV3 |
| 5.84863492378251e-18 | -1.357756074 | 0.048 | 0.118 | 1.44361855823724e-13 | KIAA1109 |
| 6.150810742525e-27 | -1.357664115 | 0.086 | 0.199 | 1.51820461557745e-22 | PTGER4 |
| 1.58160479629453e-24 | -1.352708113 | 0.075 | 0.176 | 3.90387511869379e-20 | GATM |
| 1.41071157811288e-25 | -1.352622145 | 0.064 | 0.161 | 3.48205938825603e-21 | LAIR1 |
| 5.95066205169502e-53 | -1.349299949 | 0.243 | 0.459 | 1.46880191421988e-48 | DDX3X |
| 2.68475808926553e-17 | -1.34878735 | 0.04 | 0.105 | 6.62678839173412e-13 | NEK7 |
| 6.88153528953291e-17 | -1.345658871 | 0.04 | 0.103 | 1.69856935551541e-12 | UBN1 |
| 2.00563367761412e-35 | -1.344583324 | 0.143 | 0.299 | 4.95050560645492e-31 | SYAP1 |
| 5.71559759852927e-28 | -1.338726846 | 0.07 | 0.177 | 1.41078095524498e-23 | SLC43A2 |
| 4.02489627431213e-24 | -1.33710675 | 0.083 | 0.186 | 9.93465147388463e-20 | ADAP2 |
| 7.0870132587279e-21 | -1.335894274 | 0.06 | 0.144 | 1.74928748265181e-16 | RNF145 |
| 2.00824938636709e-19 | -1.331271937 | 0.058 | 0.138 | 4.95696196036988e-15 | LAGE3 |
| 2.91750144969151e-19 | -1.329575148 | 0.053 | 0.129 | 7.20126882827356e-15 | IGF1 |
| 2.0442448645268e-41 | -1.326671263 | 0.146 | 0.317 | 5.04580959911151e-37 | CHD1 |
| 1.78196833869767e-23 | -1.324992735 | 0.055 | 0.143 | 4.39843245040746e-19 | RCOR1 |
| 1.90343780575399e-20 | -1.323619954 | 0.055 | 0.135 | 4.69825553594257e-16 | TBC1D14 |
| 8.49376073567344e-35 | -1.319641005 | 0.131 | 0.283 | 2.09651496238627e-30 | NAAA |
| 8.16983782417817e-22 | -1.318150484 | 0.056 | 0.14 | 2.0165610701419e-17 | ATP2B1-AS1 |
| 2.67021651595074e-19 | -1.316962872 | 0.051 | 0.126 | 6.59089542632121e-15 | NIPSNAP2 |
| 3.99845692283925e-17 | -1.316962872 | 0.059 | 0.133 | 9.86939122264412e-13 | TSC22D2 |
| 2.81473347820974e-58 | -1.314706096 | 0.136 | 0.345 | 6.94760664426509e-54 | TNFAIP2 |
| 4.63250239481156e-23 | -1.314110285 | 0.074 | 0.171 | 1.14344056611134e-18 | SLC25A19 |
| 8.01138270439859e-17 | -1.314110285 | 0.045 | 0.112 | 1.9774495929267e-12 | AC100810.1 |
| 2.06656767533478e-23 | -1.302463302 | 0.065 | 0.159 | 5.10090899302884e-19 | CX3CR1 |
| 1.16296912227054e-42 | -1.299528278 | 0.113 | 0.277 | 2.87055668450037e-38 | DPP7 |
| 3.28596380857516e-15 | -1.299396999 | 0.045 | 0.108 | 8.11074446870606e-11 | IGFBP4 |
| 3.64208224320178e-23 | -1.292790562 | 0.059 | 0.149 | 8.98975160089495e-19 | APAF1 |
| 4.99296316778287e-25 | -1.291636354 | 0.059 | 0.153 | 1.23241309870385e-20 | SMIM4 |
| 1.05626985140013e-16 | -1.290705331 | 0.039 | 0.102 | 2.60719087421095e-12 | NOTCH2NLA |
| 2.4554531493023e-34 | -1.28991093 | 0.103 | 0.242 | 6.06079500842286e-30 | NCOR1 |
| 4.72527798965677e-40 | -1.28954419 | 0.154 | 0.326 | 1.16634036618698e-35 | SOCS3 |
| 1.48865076551581e-16 | -1.282301864 | 0.057 | 0.129 | 3.67443668452268e-12 | DNAJB4 |
| 6.89804315231071e-16 | -1.281333497 | 0.048 | 0.114 | 1.70264399128485e-11 | C5orf24 |
| 3.97351250671242e-22 | -1.28099376 | 0.067 | 0.159 | 9.80782092031827e-18 | FCHSD2 |
| 4.28946442251715e-72 | -1.276976436 | 0.388 | 0.633 | 1.05876850340991e-67 | MCL1 |
| 5.7124804214604e-16 | -1.273087436 | 0.04 | 0.101 | 1.41001154242907e-11 | DNAJC1 |
| 5.99232155035974e-19 | -1.267925378 | 0.066 | 0.149 | 1.47908472827529e-14 | USP36 |
| 6.79196226994537e-27 | -1.265800139 | 0.074 | 0.182 | 1.67646004709062e-22 | UQCRFS1 |
| 2.10517904646835e-24 | -1.265592141 | 0.069 | 0.166 | 5.19621344039783e-20 | SERPING1 |
| 6.39128734781729e-25 | -1.264054034 | 0.079 | 0.184 | 1.57756145606174e-20 | C1QBP |
| 5.22505004773126e-39 | -1.261303926 | 0.102 | 0.253 | 1.28969910328151e-34 | CACUL1 |
| 4.65720969052699e-27 | -1.260560167 | 0.077 | 0.186 | 1.14953906791278e-22 | EHD4 |
| 6.85071545291983e-43 | -1.258946468 | 0.16 | 0.34 | 1.6909620952442e-38 | UVRAG |
| 3.51038086044037e-15 | -1.258589541 | 0.043 | 0.104 | 8.66467307782496e-11 | ZRSR2 |
| 1.43455384445922e-16 | -1.256264686 | 0.053 | 0.124 | 3.54090925427869e-12 | MCOLN1 |
| 4.13602892369955e-66 | -1.255014767 | 0.219 | 0.475 | 1.02089601923676e-61 | WASF2 |
| 1.29372543213631e-32 | -1.254662581 | 0.095 | 0.227 | 3.19330248414206e-28 | CCND3 |
| 1.31971698799929e-63 | -1.254448261 | 0.205 | 0.448 | 3.25745744147866e-59 | FCGR2A |
| 3.85515222897466e-26 | -1.253969228 | 0.155 | 0.289 | 9.51567224677816e-22 | GLUD1 |
| 1.58848729352635e-16 | -1.251486097 | 0.073 | 0.152 | 3.92086318661109e-12 | ARHGAP5 |
| 2.7512376719766e-19 | -1.250779147 | 0.067 | 0.152 | 6.79087994573984e-15 | OXSR1 |
| 1.47138839598939e-19 | -1.250595936 | 0.133 | 0.244 | 3.63182797782061e-15 | HIST1H4C |
| 6.63440746796985e-21 | -1.242432077 | 0.088 | 0.185 | 1.637570795319e-16 | LRIF1 |
| 4.66953220991055e-11 | -1.239701387 | 0.057 | 0.113 | 1.15258063537222e-06 | COLEC12 |
| 4.89394583848203e-25 | -1.236601087 | 0.117 | 0.235 | 1.20797265131252e-20 | HMGA1 |
| 1.45819506083646e-14 | -1.233431903 | 0.045 | 0.106 | 3.59926286866263e-10 | EPB41L4A-AS1 |
| 6.73196390105271e-20 | -1.232932182 | 0.085 | 0.178 | 1.66165064969684e-15 | PMAIP1 |
| 4.70895790548343e-22 | -1.230948511 | 0.077 | 0.173 | 1.16231207981048e-17 | SLC4A7 |
| 8.85110061954604e-25 | -1.227695534 | 0.075 | 0.177 | 2.18471716592255e-20 | FLI1 |
| 4.52101850123017e-14 | -1.225252358 | 0.088 | 0.164 | 1.11592299665864e-09 | PLAU |
| 2.5532146347977e-25 | -1.224934394 | 0.094 | 0.207 | 6.30209968307117e-21 | PPM1G |
| 1.67576504928357e-36 | -1.224181049 | 0.135 | 0.293 | 4.13629087114663e-32 | CHD2 |
| 1.97644601229856e-14 | -1.222659944 | 0.051 | 0.115 | 4.87846169215654e-10 | KIF1B |
| 8.83198713510749e-14 | -1.222515294 | 0.05 | 0.111 | 2.17999938455858e-09 | HERC2 |
| 1.2119786197142e-27 | -1.222070984 | 0.088 | 0.204 | 2.99152682704056e-23 | TGFBR2 |
| 2.23886336515453e-13 | -1.218477004 | 0.057 | 0.121 | 5.52618644421093e-09 | RELL1 |
| 2.69987760018069e-15 | -1.21635056 | 0.065 | 0.136 | 6.664107880526e-11 | SCARB2 |
| 9.4059165386214e-16 | -1.215417204 | 0.042 | 0.104 | 2.32166237922792e-11 | MEF2D |
| 2.65262823627662e-19 | -1.213048758 | 0.061 | 0.143 | 6.54748227560158e-15 | TLR4 |
| 3.18598631183634e-40 | -1.204425754 | 0.169 | 0.346 | 7.86397001350565e-36 | PNRC2 |
| 2.81745326549781e-35 | -1.200214797 | 0.202 | 0.374 | 6.95431989522823e-31 | GLA |
| 1.44722535437565e-26 | -1.2000038 | 0.094 | 0.21 | 3.57218634220543e-22 | PSTPIP1 |
| 2.00096793206878e-11 | -1.199350908 | 0.047 | 0.1 | 4.93898914672537e-07 | CCDC93 |
| 7.97343992221505e-18 | -1.199126381 | 0.057 | 0.132 | 1.96808417600034e-13 | RIN3 |
| 7.26231152106328e-69 | -1.198290895 | 0.377 | 0.633 | 1.79255635274405e-64 | MRC1 |
| 4.72315440258436e-60 | -1.195199152 | 0.263 | 0.505 | 1.1658162011899e-55 | ZYX |
| 2.61630743404675e-38 | -1.19452867 | 0.135 | 0.299 | 6.45783163945759e-34 | PABPC4 |
| 8.79755740319872e-16 | -1.193344029 | 0.064 | 0.137 | 2.17150109383154e-11 | TMX4 |
| 2.61151585109565e-39 | -1.193128454 | 0.15 | 0.32 | 6.44600457525939e-35 | HMGN1 |
| 5.40855814957799e-17 | -1.192506105 | 0.059 | 0.133 | 1.33499440806034e-12 | WIPI2 |
| 5.70148288193339e-31 | -1.192438209 | 0.138 | 0.281 | 1.40729701974762e-26 | MBP |
| 1.32820235024277e-12 | -1.191475346 | 0.045 | 0.1 | 3.27840186110423e-08 | TP53BP2 |
| 1.24568424833806e-33 | -1.191284893 | 0.201 | 0.366 | 3.07472243017284e-29 | ZFAND5 |
| 8.3829411653417e-50 | -1.190220828 | 0.159 | 0.366 | 2.06916136784129e-45 | IRF8 |
| 5.65081630193429e-17 | -1.189940612 | 0.049 | 0.119 | 1.39479098780644e-12 | SMARCB1 |
| 1.37092439355845e-11 | -1.187894525 | 0.052 | 0.107 | 3.38385268062033e-07 | EIF4EBP3 |
| 7.47862515834191e-16 | -1.18624509 | 0.077 | 0.156 | 1.84594904783353e-11 | FUCA1 |
| 8.18436786688585e-14 | -1.183026484 | 0.058 | 0.124 | 2.02014752058344e-09 | COLGALT1 |
| 2.87658514969015e-39 | -1.182517128 | 0.204 | 0.385 | 7.10027512498021e-35 | TMEM123 |
| 4.844852814889e-33 | -1.182005626 | 0.283 | 0.453 | 1.19585502029905e-28 | DAB2 |
| 8.02777471374245e-87 | -1.181210631 | 0.412 | 0.718 | 1.98149563259305e-82 | FCGRT |
| 6.86322075331956e-07 | -1.180389819 | 0.068 | 0.113 | 0.0169404877854187 | IL10 |
| 7.63002520479422e-86 | -1.174760532 | 0.518 | 0.764 | 1.88331912129936e-81 | PPP1R15A |
| 1.16360516729478e-18 | -1.171388093 | 0.092 | 0.186 | 2.87212663443371e-14 | MARCO |
| 1.55274445556122e-31 | -1.170844402 | 0.112 | 0.25 | 3.83263913966176e-27 | LPCAT2 |
| 7.65437138343626e-40 | -1.170183075 | 0.118 | 0.279 | 1.88932848857357e-35 | MCTP1 |
| 5.10306768591765e-51 | -1.169561358 | 0.188 | 0.403 | 1.25959019691505e-46 | GUK1 |
| 1.98242162920055e-13 | -1.169199884 | 0.052 | 0.113 | 4.89321130735573e-09 | FKBP4 |
| 1.95475787932685e-24 | -1.168533461 | 0.078 | 0.182 | 4.82492887354246e-20 | KDELR2 |
| 4.80369632260848e-34 | -1.168277323 | 0.156 | 0.314 | 1.18569636330945e-29 | OTULINL |
| 1.89864478944483e-27 | -1.167221675 | 0.104 | 0.227 | 4.68642493378668e-23 | AFF4 |
| 5.11894356503487e-35 | -1.160642512 | 0.167 | 0.331 | 1.26350884015756e-30 | KLF10 |
| 1.02771852702502e-20 | -1.158868409 | 0.105 | 0.208 | 2.53671764025586e-16 | LILRB5 |
| 9.08589194474916e-18 | -1.156562357 | 0.053 | 0.126 | 2.24267070872244e-13 | MIR155HG |
| 6.17052425292752e-29 | -1.155813505 | 0.159 | 0.303 | 1.5230705013501e-24 | AKAP9 |
| 1.06598535403073e-13 | -1.153056733 | 0.043 | 0.1 | 2.63117164935406e-09 | PANK2 |
| 1.52565604449298e-15 | -1.148406624 | 0.067 | 0.141 | 3.76577681462202e-11 | TCEA3 |
| 5.97631748152101e-13 | -1.147771989 | 0.06 | 0.123 | 1.47513444396383e-08 | SGMS1 |
| 5.07706221337793e-19 | -1.145233373 | 0.064 | 0.147 | 1.25317126612807e-14 | PLCB2 |
| 3.60250376807142e-37 | -1.14377305 | 0.194 | 0.369 | 8.8920600507307e-33 | VSIR |
| 2.93870602903534e-17 | -1.143162023 | 0.066 | 0.145 | 7.25360809146794e-13 | CASC3 |
| 3.07083259971312e-13 | -1.142972855 | 0.048 | 0.107 | 7.5797361058719e-09 | RNF115 |
| 8.87369338655186e-23 | -1.14172231 | 0.086 | 0.189 | 2.19029373860259e-18 | GTF3A |
| 1.59942489300665e-22 | -1.13912136 | 0.083 | 0.185 | 3.94786046340831e-18 | CLTB |
| 1.76233299133077e-49 | -1.138971632 | 0.29 | 0.505 | 4.34996652250174e-45 | BRD2 |
| 5.90247993257287e-22 | -1.13693007 | 0.085 | 0.186 | 1.45690912175696e-17 | NUMA1 |
| 2.25983952703846e-19 | -1.134825959 | 0.068 | 0.154 | 5.57796190458903e-15 | MKNK2 |
| 3.0759606080629e-12 | -1.132275968 | 0.046 | 0.101 | 7.59239356888165e-08 | ANKLE2 |
| 1.70643485364975e-14 | -1.12945144 | 0.058 | 0.125 | 4.21199314926368e-10 | TBC1D9 |
| 8.45029317479924e-22 | -1.129131699 | 0.11 | 0.22 | 2.0857858643357e-17 | RBM47 |
| 1.00403393898418e-44 | -1.127733972 | 0.264 | 0.478 | 2.47825697159465e-40 | MS4A7 |
| 6.2459314812008e-23 | -1.127368475 | 0.088 | 0.193 | 1.54168326750479e-18 | SLC8A1 |
| 2.41183696139423e-29 | -1.127048385 | 0.129 | 0.268 | 5.95313717180939e-25 | SLC66A3 |
| 9.28136999931448e-21 | -1.125333816 | 0.091 | 0.191 | 2.29092055693079e-16 | JAK2 |
| 7.73068891298142e-14 | -1.123106632 | 0.049 | 0.11 | 1.9081659443912e-09 | OSBPL1A |
| 1.73518740352264e-13 | -1.12078033 | 0.047 | 0.106 | 4.28296306811494e-09 | PTGES2 |
| 6.73052479611867e-13 | -1.119635788 | 0.049 | 0.108 | 1.66129543542597e-08 | SLC9A3R1 |
| 6.94135715111303e-19 | -1.119362912 | 0.089 | 0.182 | 1.71333518560923e-14 | C7orf50 |
| 2.49054022863253e-14 | -1.117271544 | 0.05 | 0.113 | 6.14740044633366e-10 | FAM111A |
| 5.63152542664812e-11 | -1.117271544 | 0.065 | 0.124 | 1.39002942105956e-06 | RALGDS |
| 2.61508715432724e-26 | -1.11486878 | 0.168 | 0.304 | 6.45481962302593e-22 | GTF2B |
| 2.77318474605589e-09 | -1.114686572 | 0.052 | 0.1 | 6.84505190868975e-05 | ATP6V0A1 |
| 3.21894952100617e-26 | -1.112218316 | 0.098 | 0.217 | 7.94533310269953e-22 | BRI3 |
| 1.31178178127841e-14 | -1.111553939 | 0.065 | 0.135 | 3.23787097072949e-10 | CHRAC1 |
| 1.95650547806554e-15 | -1.109698324 | 0.043 | 0.104 | 4.82924247150918e-11 | CCDC80 |
| 3.43951771049585e-15 | -1.108909381 | 0.075 | 0.151 | 8.4897615648169e-11 | HBEGF |
| 4.01259869107787e-12 | -1.108909381 | 0.054 | 0.113 | 9.9042973491875e-08 | DAP |
| 1.9232250038255e-15 | -1.1074013 | 0.062 | 0.134 | 4.74709627694249e-11 | STK38 |
| 4.71459136250381e-18 | -1.104584991 | 0.067 | 0.149 | 1.16370258600682e-13 | RERE |
| 6.40580351085049e-22 | -1.103637541 | 0.092 | 0.196 | 1.58114448058323e-17 | ADIPOR1 |
| 2.5436647737071e-10 | -1.103574222 | 0.052 | 0.104 | 6.27852776094123e-06 | NRIP1 |
| 3.72223242901966e-23 | -1.103021613 | 0.134 | 0.255 | 9.18758630454924e-19 | RGL1 |
| 1.94136913785215e-24 | -1.102682954 | 0.09 | 0.2 | 4.79188144296047e-20 | SRSF9 |
| 3.31807479046595e-11 | -1.101943464 | 0.108 | 0.178 | 8.19000400530709e-07 | AREG |
| 5.53391999077847e-12 | -1.101786636 | 0.063 | 0.125 | 1.36593747132385e-07 | HIVEP1 |
| 4.67134629798972e-33 | -1.101554503 | 0.142 | 0.297 | 1.1530284067328e-28 | GRINA |
| 2.58851237743733e-24 | -1.100656345 | 0.091 | 0.201 | 6.38922510122856e-20 | GAA |
| 1.36939863883255e-21 | -1.100460345 | 0.174 | 0.299 | 3.38008666023038e-17 | MAFB |
| 2.65632973785418e-17 | -1.097136692 | 0.152 | 0.257 | 6.55661869194547e-13 | RHOB |
| 1.01070404458346e-17 | -1.096991842 | 0.088 | 0.177 | 2.49472079324536e-13 | SKIL |
| 5.60280681187305e-38 | -1.095171448 | 0.219 | 0.404 | 1.38294080537463e-33 | IVNS1ABP |
| 1.23553682306359e-47 | -1.09178747 | 0.383 | 0.592 | 3.04967554036786e-43 | C1QA |
| 3.3890655478963e-37 | -1.089600013 | 0.164 | 0.336 | 8.36523049187245e-33 | DOCK8 |
| 3.39103112900429e-18 | -1.085091138 | 0.083 | 0.171 | 8.3700821357213e-14 | IFRD1 |
| 3.81057429118865e-26 | -1.085070389 | 0.145 | 0.277 | 9.40564052294094e-22 | TGIF1 |
| 7.11024388568659e-20 | -1.084168222 | 0.081 | 0.174 | 1.75502149830402e-15 | BAG3 |
| 1.03435998940342e-18 | -1.083305624 | 0.129 | 0.234 | 2.55311076184445e-14 | POLR2A |
| 1.93149278434086e-24 | -1.081675276 | 0.233 | 0.373 | 4.76750363958855e-20 | MAT2A |
| 1.66139392936311e-28 | -1.081617274 | 0.161 | 0.304 | 4.10081863584697e-24 | HLA-DOA |
| 4.27367613302999e-42 | -1.080784374 | 0.193 | 0.388 | 1.05487147991579e-37 | CFP |
| 2.353061136305e-21 | -1.079525085 | 0.096 | 0.199 | 5.80806080274163e-17 | LRRFIP2 |
| 1.10943102758481e-31 | -1.078832148 | 0.15 | 0.301 | 2.73840860538758e-27 | SNX10 |
| 1.9053396974215e-16 | -1.076149061 | 0.061 | 0.135 | 4.7029499751455e-12 | HINT2 |
| 6.25886315127321e-11 | -1.075173578 | 0.057 | 0.113 | 1.54487519162877e-06 | UBAP1 |
| 1.00770991755534e-15 | -1.073624483 | 0.119 | 0.21 | 2.48733038950185e-11 | PMP22 |
| 5.721642097401e-18 | -1.072006537 | 0.083 | 0.172 | 1.41227291890149e-13 | PYURF |
| 4.978212125227e-10 | -1.070512201 | 0.056 | 0.109 | 1.22877209886978e-05 | STAT2 |
| 3.38282449788431e-26 | -1.070154257 | 0.109 | 0.232 | 8.34982570812784e-22 | LILRB2 |
| 2.1039721527443e-12 | -1.070154257 | 0.048 | 0.105 | 5.19323446461875e-08 | RAPH1 |
| 2.25204116952579e-17 | -1.068387639 | 0.08 | 0.165 | 5.55871321874052e-13 | PTPN18 |
| 4.62781137221056e-17 | -1.067984345 | 0.075 | 0.158 | 1.14228268100273e-12 | MGLL |
| 8.52969383249307e-20 | -1.064637252 | 0.096 | 0.195 | 2.10538432867427e-15 | ARHGEF2 |
| 4.4728794803335e-14 | -1.059495213 | 0.07 | 0.141 | 1.10404084213072e-09 | SLC15A3 |
| 4.17945174171915e-22 | -1.057142044 | 0.1 | 0.208 | 1.03161407340854e-17 | FNBP4 |
| 4.85268453968364e-10 | -1.056854826 | 0.064 | 0.12 | 1.19778812493011e-05 | VAT1 |
| 2.20461860664928e-55 | -1.056077029 | 0.283 | 0.521 | 5.44166010679242e-51 | EIF5 |
| 1.49942489659714e-13 | -1.051953205 | 0.081 | 0.154 | 3.70103047227072e-09 | MID1IP1 |
| 9.1082373412389e-18 | -1.049358292 | 0.083 | 0.171 | 2.248186222938e-13 | EPSTI1 |
| 1.98712544557302e-20 | -1.04902449 | 0.04 | 0.112 | 4.9048217373079e-16 | MGP |
| 5.2422122314462e-09 | -1.047874496 | 0.073 | 0.127 | 0.000129393524508787 | TFRC |
| 6.37614036832661e-18 | -1.047487559 | 0.091 | 0.183 | 1.57382272711406e-13 | IDH2 |
| 8.35756737629617e-23 | -1.045133706 | 0.132 | 0.252 | 2.06289835549118e-18 | PLEKHO1 |
| 1.8711390064139e-10 | -1.044690705 | 0.05 | 0.101 | 4.61853240953142e-06 | USP48 |
| 4.86256670079526e-52 | -1.043319827 | 0.346 | 0.574 | 1.2002273387573e-47 | WSB1 |
| 1.84719330572538e-11 | -1.042820191 | 0.048 | 0.102 | 4.55942723652195e-07 | NTAN1 |
| 1.18405386823602e-31 | -1.042777818 | 0.168 | 0.325 | 2.92260016296698e-27 | JMJD1C |
| 4.63958165990181e-13 | -1.042416285 | 0.054 | 0.115 | 1.14518794111356e-08 | C20orf194 |
| 1.99431719381999e-16 | -1.04213988 | 0.054 | 0.125 | 4.92257312950588e-12 | C3AR1 |
| 6.02699432896057e-20 | -1.040364255 | 0.131 | 0.241 | 1.48764301021734e-15 | ATP1A1 |
| 4.03426484897199e-19 | -1.038217735 | 0.089 | 0.184 | 9.95777592671756e-15 | ADAM15 |
| 2.94338447880216e-11 | -1.037011972 | 0.058 | 0.115 | 7.26515590902737e-07 | LRRC58 |
| 3.42965457449448e-17 | -1.035050456 | 0.082 | 0.167 | 8.46541638622473e-13 | SLC25A24 |
| 6.74558335634969e-17 | -1.034627192 | 0.086 | 0.172 | 1.66501233984779e-12 | PAXX |
| 1.88574555541234e-13 | -1.033522986 | 0.074 | 0.144 | 4.65458575442429e-09 | FGD5-AS1 |
| 8.00164730237514e-15 | -1.033391868 | 0.064 | 0.135 | 1.97504660364526e-10 | ST3GAL1 |
| 1.19907804445145e-12 | -1.032967141 | 0.072 | 0.139 | 2.95968433711952e-08 | TRIM8 |
| 9.91382845986711e-15 | -1.032144724 | 0.075 | 0.151 | 2.447030278749e-10 | RBM33 |
| 1.3132323861821e-24 | -1.030803484 | 0.124 | 0.248 | 3.24145149881327e-20 | CASP1 |
| 5.44049397118839e-32 | -1.030639405 | 0.187 | 0.35 | 1.34287712690843e-27 | PLD3 |
| 1.39046268001811e-24 | -1.026826825 | 0.127 | 0.252 | 3.43207903308871e-20 | MEF2A |
| 2.43838926661328e-20 | -1.024355855 | 0.12 | 0.229 | 6.01867622678157e-16 | SH3KBP1 |
| 3.24319513860089e-51 | -1.023718684 | 0.306 | 0.541 | 8.00517856060858e-47 | CSF1R |
| 3.99107760691751e-75 | -1.019355097 | 0.646 | 0.778 | 9.85117685715449e-71 | YBX1 |
| 1.25154819847737e-31 | -1.01470181 | 0.146 | 0.301 | 3.0891964183017e-27 | EMILIN2 |
| 1.16782393997098e-10 | -1.01398884 | 0.049 | 0.101 | 2.88253983103038e-06 | CTTNBP2 |
| 2.04066987210657e-12 | -1.012378629 | 0.063 | 0.126 | 5.03698544532065e-08 | SLC25A39 |
| 3.52251633415737e-27 | -1.010967815 | 0.149 | 0.288 | 8.69462706760064e-23 | PTPN1 |
| 3.30919890307931e-12 | -1.009349945 | 0.064 | 0.127 | 8.16809565247065e-08 | RASSF1 |
| 6.32650864618594e-40 | -1.009209329 | 0.309 | 0.507 | 1.56157212913807e-35 | PDIA3 |
| 2.16915759005945e-14 | -1.008588686 | 0.115 | 0.2 | 5.35413167954373e-10 | SCN9A |
| 1.33851486143191e-20 | -1.006836263 | 0.108 | 0.214 | 3.30385623247237e-16 | ZFHX3 |
| 5.20553691751981e-28 | -1.005893775 | 0.134 | 0.272 | 1.28488267735142e-23 | EIF2AK4 |
| 2.64128414050833e-13 | -1.002207199 | 0.053 | 0.115 | 6.51948164401672e-09 | DIAPH2 |
| 1.82538819746345e-09 | -1.001033517 | 0.089 | 0.149 | 4.50560568779904e-05 | SOX4 |
| 9.64271771630633e-40 | 1.091460768 | 0.946 | 0.919 | 2.38011201391589e-35 | CRIP1 |
| 6.40069639567369e-09 | 1.0003656378862 | 0.175 | 0.104 | 0.000157988389134414 | POLE4 |
| 2.99221139300452e-17 | 1.0030993287952 | 0.274 | 0.15 | 7.38567538135305e-13 | ANXA4 |
| 7.68592436940331e-33 | 1.0041474321181 | 0.481 | 0.294 | 1.89711671209982e-28 | TMEM50A |
| 2.28727351834919e-08 | 1.00447790859209 | 0.174 | 0.107 | 0.000564567722534131 | SLC16A3 |
| 8.65974045306639e-07 | 1.00752092815709 | 0.101 | 0.053 | 0.0213748373603038 | SMIM30 |
| 1.95951323371507e-78 | 1.00849161668506 | 0.964 | 0.971 | 4.83666651477892e-74 | SH3BGRL3 |
| 1.31119651315347e-16 | 1.01195487722044 | 0.275 | 0.156 | 3.23642635341672e-12 | NDUFB3 |
| 9.41782271159979e-08 | 1.0143792536612 | 0.109 | 0.054 | 0.00232460117990418 | ARL6IP6 |
| 7.90771725485411e-29 | 1.01476801546385 | 0.434 | 0.257 | 1.95186185001564e-24 | NOP10 |
| 1.52963474385165e-22 | 1.01993417929853 | 0.344 | 0.193 | 3.77559743824903e-18 | FAM162A |
| 2.07253345300867e-19 | 1.02098357931978 | 0.29 | 0.155 | 5.11563432206131e-15 | DYNLRB1 |
| 2.48684346827564e-11 | 1.02156349365219 | 0.173 | 0.09 | 6.13827573274477e-07 | SYPL1 |
| 1.2485638893933e-54 | 1.02176292301797 | 0.661 | 0.445 | 3.08183024818949e-50 | RAN |
| 9.92823817986639e-08 | 1.02239981658935 | 0.114 | 0.058 | 0.00245058702993642 | CD300C |
| 1.14330929638514e-10 | 1.02304151349347 | 0.176 | 0.096 | 2.82203033626743e-06 | DCTPP1 |
| 3.54595033958249e-25 | 1.02677906750328 | 0.404 | 0.245 | 8.75246922319147e-21 | OSTC |
| 2.55222851276961e-13 | 1.0305739241281 | 0.205 | 0.108 | 6.29966563806924e-09 | VMP1 |
| 2.41532243768009e-08 | 1.03157695350065 | 0.132 | 0.07 | 0.000596174037292576 | MRPL15 |
| 2.03033445533184e-09 | 1.03254023894418 | 0.143 | 0.074 | 5.01147453609559e-05 | PSMD14 |
| 4.31051472913116e-44 | 1.03381963433968 | 0.609 | 0.426 | 1.06396435059144e-39 | ACTR3 |
| 1.07605788906934e-12 | 1.03485401427149 | 0.191 | 0.099 | 2.65603368758986e-08 | POLR2I |
| 1.22275056492769e-07 | 1.0393706263159 | 0.117 | 0.061 | 0.00301811521941101 | GOLT1B |
| 1.26883052572488e-09 | 1.04267435531394 | 0.153 | 0.082 | 3.13185438664671e-05 | COPS2 |
| 1.52081065795563e-14 | 1.04419187885082 | 0.204 | 0.101 | 3.75381694703189e-10 | PSMB5 |
| 4.7132075205286e-09 | 1.0464404628935 | 0.142 | 0.075 | 0.000116336101229208 | RAB29 |
| 9.12844050668957e-12 | 1.04670419126783 | 0.172 | 0.088 | 2.25317297026619e-07 | MRPL27 |
| 1.60239896234748e-07 | 1.04932933678737 | 0.106 | 0.053 | 0.00395520135876229 | SERPINF2 |
| 9.10931093971158e-07 | 1.05005311278187 | 0.107 | 0.057 | 0.0224845121924901 | TRADD |
| 6.06753625946315e-21 | 1.05282629902569 | 0.298 | 0.158 | 1.49764997492329e-16 | PSMA3 |
| 1.66788947951587e-39 | 1.05428318130271 | 0.532 | 0.331 | 4.11685160228903e-35 | MICOS10 |
| 3.69990704496154e-83 | 1.05921811739858 | 0.991 | 0.979 | 9.13248055907856e-79 | S100A10 |
| 9.67256030629661e-08 | 1.06407059084017 | 0.102 | 0.049 | 0.00238747806040319 | ZMPSTE24 |
| 8.6801871028087e-18 | 1.06440133789931 | 0.298 | 0.175 | 2.14253058258627e-13 | TUBB6 |
| 4.91808687119253e-65 | 1.06697997677298 | 0.716 | 0.507 | 1.21393138241645e-60 | ATP5MC3 |
| 4.13336220534259e-08 | 1.06776034998923 | 0.114 | 0.056 | 0.00102023779314471 | NMI |
| 2.25306764502361e-12 | 1.07205577050574 | 0.186 | 0.096 | 5.56124686821179e-08 | CNIH1 |
| 4.4033561583725e-13 | 1.0733155938224 | 0.203 | 0.108 | 1.08688040057108e-08 | TAX1BP3 |
| 6.94679605878631e-27 | 1.07464275988821 | 0.392 | 0.227 | 1.71467767119022e-22 | HSBP1 |
| 5.03776196704608e-16 | 1.07844367554629 | 0.24 | 0.127 | 1.24347078632598e-11 | TIMM17A |
| 4.46309078532995e-10 | 1.08299558946772 | 0.171 | 0.095 | 1.10162469854299e-05 | ACTN1 |
| 1.12736724986346e-27 | 1.08423025681632 | 0.382 | 0.211 | 2.78268058283797e-23 | UBE2L3 |
| 1.65225561453195e-29 | 1.08984301930014 | 0.464 | 0.299 | 4.07826253334921e-25 | CIB1 |
| 2.08567967439266e-18 | 1.09070949478672 | 0.296 | 0.17 | 5.14808314030341e-14 | PSMD7 |
| 5.73219519434725e-08 | 1.10031561340226 | 0.119 | 0.061 | 0.00141487773982073 | FHOD1 |
| 3.43502071498424e-07 | 1.10108623239148 | 0.104 | 0.054 | 0.0084786616307956 | NDRG1 |
| 1.83454908736375e-09 | 1.11099744382982 | 0.135 | 0.068 | 4.52821751233994e-05 | ZNRD2 |
| 3.53849070738961e-08 | 1.11243715672801 | 0.114 | 0.056 | 0.000873405661304977 | POLR2J3 |
| 7.55626070427234e-11 | 1.11369287535624 | 0.145 | 0.07 | 1.86511182963554e-06 | MED4 |
| 4.53568703704789e-11 | 1.11926135584194 | 0.144 | 0.068 | 1.11954363135453e-06 | TMEM126A |
| 3.64830903550233e-16 | 1.11953399971328 | 0.217 | 0.106 | 9.00512119233039e-12 | POLR2K |
| 1.61714785020827e-09 | 1.12036627082341 | 0.147 | 0.077 | 3.99160603866907e-05 | TMEM14A |
| 1.20598212559825e-09 | 1.12985647108114 | 0.124 | 0.058 | 2.97672568061417e-05 | MRPS33 |
| 3.91948770972771e-11 | 1.13440299982595 | 0.153 | 0.075 | 9.67447151392092e-07 | CBWD1 |
| 1.66701263314556e-22 | 1.13824298750163 | 0.656 | 0.567 | 4.11468728239318e-18 | HSPB1 |
| 3.99668891268124e-32 | 1.1422607434887 | 0.421 | 0.236 | 9.8650272431711e-28 | ATOX1 |
| 2.44083110007385e-167 | 1.1580935675649 | 0.993 | 0.959 | 6.02470340431228e-163 | S100A11 |
| 2.72188308134122e-54 | 1.15901324268341 | 0.597 | 0.365 | 6.71842400967454e-50 | POMP |
| 2.15577802119766e-17 | 1.15910066152765 | 0.264 | 0.144 | 5.32110688972218e-13 | QPCT |
| 4.38626550726475e-18 | 1.16194278904594 | 0.248 | 0.126 | 1.08266191515816e-13 | TIMM8B |
| 2.93691834941927e-29 | 1.17127144383646 | 0.384 | 0.212 | 7.24919556187159e-25 | PSMA1 |
| 2.11943648905975e-09 | 1.1808869436046 | 0.108 | 0.047 | 5.23140508594617e-05 | REXO2 |
| 2.34514211766696e-08 | 1.18904225473174 | 0.102 | 0.047 | 0.000578851428903736 | MRPL50 |
| 2.72966200615905e-22 | 1.19644417284915 | 0.268 | 0.127 | 6.73762472980239e-18 | LSM5 |
| 4.36069064786512e-14 | 1.2043423182681 | 0.163 | 0.071 | 1.07634927261255e-09 | CHCHD1 |
| 3.51703719780433e-25 | 1.2134042623281 | 0.338 | 0.183 | 8.68110291534043e-21 | GNG10 |
| 1.19491743140774e-13 | 1.21406299711918 | 0.188 | 0.093 | 2.94941469594373e-09 | MYO1G |
| 2.56378100820741e-35 | 1.21562115812644 | 0.423 | 0.229 | 6.32818066255836e-31 | PHPT1 |
| 6.23302814885168e-13 | 1.21723651540524 | 0.163 | 0.076 | 1.53849833798106e-08 | CYB5A |
| 3.22814367444196e-26 | 1.21815128565861 | 0.317 | 0.157 | 7.96802703162508e-22 | SELENOT |
| 5.22647705966694e-20 | 1.22503754704949 | 0.259 | 0.129 | 1.29005133263759e-15 | CYSTM1 |
| 2.97798912278945e-20 | 1.22543413106337 | 0.252 | 0.122 | 7.3505705517812e-16 | TMEM167A |
| 6.30691202189757e-18 | 1.23383302593593 | 0.227 | 0.11 | 1.55673509436498e-13 | MTCH2 |
| 1.23162315096196e-15 | 1.23610127766176 | 0.172 | 0.073 | 3.04001542351941e-11 | BLOC1S2 |
| 1.52430535778895e-18 | 1.23665828380508 | 0.233 | 0.112 | 3.76244291463047e-14 | GTF3C6 |
| 1.35744084460512e-30 | 1.2385924704768 | 0.351 | 0.171 | 3.35057123673882e-26 | EIF6 |
| 6.09810504126434e-17 | 1.23972334448254 | 0.221 | 0.109 | 1.50519526733528e-12 | NME1 |
| 3.52377137680529e-11 | 1.24081537901122 | 0.138 | 0.063 | 8.6977248893685e-07 | GLRX3 |
| 1.2171869469717e-08 | 1.24141863849769 | 0.103 | 0.047 | 0.000300438254121024 | CLECL1 |
| 8.095873629997e-19 | 1.24361018538865 | 0.221 | 0.1 | 1.99830448809216e-14 | SRP19 |
| 2.20777798112432e-24 | 1.24409991045148 | 0.29 | 0.14 | 5.44945839080917e-20 | NUTF2 |
| 1.32919746012482e-15 | 1.24664779450428 | 0.193 | 0.09 | 3.28085809082609e-11 | GTF2H5 |
| 7.88320912667422e-76 | 1.24846441481346 | 0.735 | 0.524 | 1.945812508737e-71 | SEC61B |
| 5.22717842645459e-33 | 1.25343115619967 | 0.387 | 0.202 | 1.29022445100179e-28 | STMP1 |
| 1.30596725083981e-28 | 1.25564093580675 | 0.309 | 0.139 | 3.22351896524791e-24 | MRPL51 |
| 1.54123469788734e-164 | 1.26181485373892 | 0.979 | 0.913 | 3.80422960479533e-160 | LGALS1 |
| 1.33621605664311e-08 | 1.26240778084277 | 0.11 | 0.053 | 0.000329818209261218 | EEF1E1 |
| 1.28702081469502e-15 | 1.26584393942062 | 0.183 | 0.082 | 3.17675347691172e-11 | MRPL13 |
| 7.15976771379391e-12 | 1.2736090381373 | 0.141 | 0.063 | 1.76724546479575e-07 | PLGRKT |
| 2.51561204766269e-48 | 1.27405643798911 | 0.51 | 0.283 | 6.20928521724581e-44 | SEC61G |
| 6.64907841360702e-10 | 1.27926445518294 | 0.116 | 0.052 | 1.64119202483062e-05 | TMEM60 |
| 5.91784333217923e-16 | 1.2931366297645 | 0.178 | 0.077 | 1.4607012696818e-11 | C11orf98 |
| 2.66401923698075e-11 | 1.30260575094887 | 0.132 | 0.058 | 6.57559868263958e-07 | CHCHD7 |
| 3.1301718846187e-46 | 1.31085017756246 | 0.471 | 0.247 | 7.72620326280434e-42 | PSMA2 |
| 6.75795673339009e-10 | 1.31190643378434 | 0.119 | 0.054 | 1.66806646050268e-05 | SUCNR1 |
| 1.20568773226184e-17 | 1.31268287371717 | 0.261 | 0.143 | 2.9759990295419e-13 | ANPEP |
| 1.05164670544351e-30 | 1.31791252854733 | 0.322 | 0.146 | 2.59577956304622e-26 | PSMA4 |
| 4.41697553826883e-66 | 1.32369432900204 | 0.614 | 0.354 | 1.09024207211089e-61 | CAPG |
| 1.19778396852709e-18 | 1.32430912890004 | 0.21 | 0.093 | 2.95649016951542e-14 | PSMD11 |
| 1.21208828147251e-36 | 1.32642100023145 | 0.399 | 0.201 | 2.9917975051586e-32 | TXNDC17 |
| 2.27480186528405e-09 | 1.3281206215247 | 0.101 | 0.042 | 5.61489344408063e-05 | MAP1S |
| 1.52995966735701e-90 | 1.32876245041412 | 0.784 | 0.587 | 3.77639944693732e-86 | LDHA |
| 3.19105282854417e-08 | 1.33885493007033 | 0.125 | 0.065 | 0.000787647569669558 | CAV1 |
| 4.35682509586553e-11 | 1.35132260343496 | 0.214 | 0.13 | 1.07539513841249e-06 | CD1B |
| 1.02640625245712e-20 | 1.3814627553003 | 0.271 | 0.135 | 2.53347855293991e-16 | IFI27 |
| 5.25343743351343e-26 | 1.38986079957973 | 0.312 | 0.157 | 1.29670596171412e-21 | ELOVL1 |
| 4.26907255552983e-21 | 1.40457268196257 | 0.19 | 0.068 | 1.05373517888143e-16 | RAB18 |
| 3.05890755582285e-103 | 1.42733820554927 | 0.753 | 0.481 | 7.55030152003754e-99 | SUB1 |
| 3.97871083294437e-14 | 1.43707623589112 | 0.142 | 0.056 | 9.82065194895658e-10 | MRPL47 |
| 3.02142603418922e-13 | 1.4385959621923 | 0.218 | 0.124 | 7.45778588018926e-09 | NUB1 |
| 4.2459693600913e-15 | 1.44392134943839 | 0.15 | 0.058 | 1.04803261715134e-10 | CNIH4 |
| 1.64611874079524e-35 | 1.44484816398988 | 0.487 | 0.307 | 4.0631148879049e-31 | KRT10 |
| 8.83408682387034e-26 | 1.44713137573879 | 0.276 | 0.125 | 2.18051765073592e-21 | MAP3K13 |
| 1.47601826760593e-18 | 1.4537298066855 | 0.193 | 0.08 | 3.64325588993171e-14 | SPTSSA |
| 2.12511342740946e-55 | 1.4683235791379 | 0.475 | 0.214 | 5.24541747287477e-51 | CD59 |
| 6.6716888681829e-15 | 1.47742573021861 | 0.147 | 0.057 | 1.64677296333359e-10 | PFKP |
| 5.33013849681189e-24 | 1.48971726320786 | 0.225 | 0.088 | 1.31563808516808e-19 | ORMDL2 |
| 2.533573142839e-17 | 1.50434337954934 | 0.225 | 0.114 | 6.2536185884695e-13 | SLC27A3 |
| 1.97738844275193e-18 | 1.51471182989319 | 0.24 | 0.12 | 4.8807878932446e-14 | IL7R |
| 1.16284437606121e-12 | 1.51719530157401 | 0.128 | 0.051 | 2.87024877343189e-08 | NIBAN2 |
| 1.72843799365729e-23 | 1.5277315964768 | 0.213 | 0.079 | 4.26630349974429e-19 | DYNLT1 |
| 1.42847189309266e-12 | 1.53263405369477 | 0.12 | 0.046 | 3.52589717372062e-08 | TXNDC9 |
| 6.35453398215261e-101 | 1.5510084276275 | 0.652 | 0.317 | 1.56848962281473e-96 | FABP5 |
| 1.57307490076626e-13 | 1.55314975850218 | 0.117 | 0.04 | 3.88282077756135e-09 | CISD1 |
| 1.11433946023671e-47 | 1.56510129283049 | 0.417 | 0.189 | 2.75052408970227e-43 | JPT1 |
| 1.27016051116503e-15 | 1.56566611644116 | 0.154 | 0.06 | 3.13513718970865e-11 | ACOT7 |
| 3.21766587566805e-30 | 1.60268405597319 | 0.254 | 0.091 | 7.94216468091144e-26 | HIGD1A |
| 7.40053928519508e-16 | 1.60324620883828 | 0.133 | 0.044 | 1.8266751117647e-11 | CCDC167 |
| 3.15166551063827e-16 | 1.63862603123109 | 0.178 | 0.078 | 7.77925597990844e-12 | IL4I1 |
| 4.45673372665219e-12 | 1.6467735843792 | 0.115 | 0.044 | 1.10005558574956e-07 | LYPD3 |
| 4.32607230273742e-14 | 1.64962194668823 | 0.118 | 0.039 | 1.06780442648468e-09 | DDB2 |
| 2.1962567881611e-18 | 1.65598034849582 | 0.154 | 0.052 | 5.42102063021804e-14 | CALCRL |
| 2.20514882985283e-26 | 1.66335485337569 | 0.341 | 0.19 | 5.44296885672575e-22 | PERP |
| 1.76491885617619e-54 | 1.67003324495303 | 0.482 | 0.237 | 4.35634921269969e-50 | RPS27L |
| 3.81837370780978e-91 | 1.68798698440736 | 0.545 | 0.182 | 9.42489182298688e-87 | RPS4Y1 |
| 2.44006868734e-13 | 1.70359171637904 | 0.109 | 0.035 | 6.02282154096133e-09 | PDLIM7 |
| 1.45180664676494e-18 | 1.74688776293321 | 0.161 | 0.056 | 3.58349434620991e-14 | CYTOR |
| 4.863913038714e-13 | 1.75643806100403 | 0.104 | 0.033 | 1.20055965534578e-08 | ABI3 |
| 2.67589353442788e-33 | 1.80287268899214 | 0.356 | 0.179 | 6.60490801102834e-29 | DMKN |
| 1.09761678592139e-12 | 1.89629318373851 | 0.104 | 0.034 | 2.70924751268976e-08 | LAMP3 |
| 2.63115090757587e-14 | 1.8994163843664 | 0.108 | 0.032 | 6.49446978516953e-10 | ADAM19 |
| 5.28621107750597e-24 | 1.95898728509951 | 0.259 | 0.119 | 1.3047954802608e-19 | LIPA |
| 2.51989292959088e-43 | 1.98370610388153 | 0.465 | 0.268 | 6.21985171810916e-39 | CST7 |
| 1.58091687564418e-14 | 1.99684215963106 | 0.107 | 0.03 | 3.90217712415252e-10 | MGST1 |
| 9.19620696221056e-49 | 2.03130130562396 | 0.418 | 0.193 | 2.26989976448243e-44 | KRT1 |
| 1.06204165143817e-35 | 2.03285725826479 | 0.377 | 0.197 | 2.62143740824483e-31 | CD1A |
| 2.20521975674821e-46 | 2.04726493860366 | 0.299 | 0.084 | 5.4431439255816e-42 | CRIP2 |
| 1.22149207055234e-17 | 2.08206718037349 | 0.23 | 0.12 | 3.01500887774433e-13 | CCL13 |
| 1.90356662431583e-24 | 2.0967762500039 | 0.199 | 0.068 | 4.69857349879876e-20 | S100A14 |
| 1.54009183892459e-111 | 2.17819628464429 | 0.746 | 0.412 | 3.80140868601758e-107 | KRT14 |
| 2.23260108248619e-44 | 2.21685184801437 | 0.339 | 0.13 | 5.51072925190067e-40 | DSP |
| 1.14602022438715e-15 | 2.25576977730001 | 0.123 | 0.039 | 2.82872171985481e-11 | RAMP1 |
| 1.33700516759294e-120 | 2.25606615043918 | 0.692 | 0.345 | 3.30012985516967e-116 | LGALS7B |
| 7.10579325062747e-20 | 2.2600414543205 | 0.13 | 0.031 | 1.75392294805238e-15 | CAVIN3 |
| 8.15350597143315e-61 | 2.26134254718568 | 0.474 | 0.225 | 2.01252987892885e-56 | SFN |
| 3.73022732288631e-48 | 2.26906843744309 | 0.388 | 0.172 | 9.20732010108028e-44 | AQP3 |
| 2.22968485676036e-143 | 2.28276283524589 | 0.642 | 0.238 | 5.5035311319416e-139 | S100A8 |
| 2.25066060623439e-94 | 2.29742506325363 | 0.504 | 0.161 | 5.55530557436835e-90 | S100A9 |
| 1.99212704210831e-37 | 2.30033989909938 | 0.248 | 0.069 | 4.91716717803595e-33 | KRTDAP |
| 4.55805091128104e-23 | 2.30673423316522 | 0.173 | 0.052 | 1.1250637064315e-18 | IL32 |
| 1.03689076489775e-44 | 2.34980310221078 | 0.357 | 0.145 | 2.5593574749971e-40 | S100B |
| 5.20584221867488e-24 | 2.42501157382005 | 0.23 | 0.095 | 1.28495803483552e-19 | LTB |
| 1.35839464271017e-24 | 2.43526947918549 | 0.147 | 0.03 | 3.35292549660151e-20 | MIR4435-2HG |
| 2.08728943568108e-85 | 2.44034600643843 | 0.577 | 0.281 | 5.1520565140916e-81 | KRT5 |
| 7.88770071320151e-27 | 2.44812039763533 | 0.174 | 0.044 | 1.94692116703953e-22 | S100A16 |
| 8.11585255545902e-60 | 2.57055380206775 | 0.421 | 0.172 | 2.00323588626395e-55 | LY6D |
| 3.41199553098687e-59 | 2.63816382468798 | 0.425 | 0.18 | 8.42182856913489e-55 | S100A2 |
| 8.89001652601185e-47 | 2.64838283724085 | 0.284 | 0.074 | 2.1943227791155e-42 | LGALS7 |
| 4.10236024861668e-178 | 2.80153790400972 | 0.867 | 0.555 | 1.01258558016605e-173 | TXN |
| 1.08045282812182e-18 | 2.86618214727682 | 0.113 | 0.023 | 2.66688171565309e-14 | MIR205HG |
| 9.90988909801428e-28 | 3.10960778723733 | 0.145 | 0.022 | 2.44605792606286e-23 | DSC3 |
| 5.48230712382568e-87 | 3.25994355383535 | 0.463 | 0.153 | 1.35319786737389e-82 | KRT6A |
| 5.65754753416552e-95 | 3.26088243075987 | 0.469 | 0.142 | 1.39645245785808e-90 | KRT16 |
| 2.1191471050157e-41 | 3.66368696731554 | 0.204 | 0.029 | 5.23069079931025e-37 | CLDN1 |
| 1.30712861440243e-78 | 3.70983433442194 | 0.354 | 0.063 | 3.22638555892952e-74 | S100A7 |
| 9.29980000623737e-38 | 3.7620214932012 | 0.171 | 0.017 | 2.29546963553957e-33 | DSG1 |
| 3.21612490304816e-17 | 4.01225525918381 | 0.118 | 0.031 | 7.93836109819377e-13 | CCL22 |
| 2.03009674501831e-34 | 4.28958015967264 | 0.142 | 0.006 | 5.01088779572869e-30 | KRT17 |
| 7.48596571975335e-28 | 4.68519380269302 | 0.116 | 0.005 | 1.84776091860672e-23 | CALML5 |
| 2.47869511191335e-57 | 5.04636817285688 | 0.255 | 0.031 | 6.11816314473572e-53 | CCL17 |
| 4.57889688502709e-42 | 5.26326673532331 | 0.171 | 0.008 | 1.13020911813124e-37 | SERPINB4 |
| 4.87110813146849e-37 | 5.28603206241549 | 0.15 | 0.006 | 1.20233562009037e-32 | KRT6B |
| 2.73950579689939e-28 | 5.57158608798498 | 0.112 | 0.002 | 6.76192215848677e-24 | KRT2 |
| 6.07671112921694e-62 | 6.87758082796853 | 0.243 | 0.014 | 1.49991460802462e-57 | MMP12 |
| 5.76605599816274e-57 | 7.14578858375238 | 0.261 | 0.038 | 1.42323560202651e-52 | CCL18 |
| 5.89908440460645e-34 | 8.07950527560387 | 0.129 | 0 | 1.45607100358901e-29 | KRT6C |

| **Table S8: Mendelian randomization analysis results** | | | | | | | | | | | | |
| --- | --- | --- | --- | --- | --- | --- | --- | --- | --- | --- | --- | --- |
| **id** | **nsnp** | **beta** | **se** | **or** | **or_lci95** | **or_uci95** | **FDR** | **Heter.Stat** | **Heter.p** | **egger_intercept** | **egger_intercept_se** | **egger_intercept_pval** |
| FOSL2 | 92 | 0.3753 | 0.058 | 1.45543 | 1.29903 | 1.63065 | ＜0.00001 | 497.76109 | ＜0.00001 | -0.03918 | 0.00834 | 0.00001 |
| ZC3H12A | 222 | 0.22049 | 0.01194 | 1.24669 | 1.21786 | 1.27621 | ＜0.00001 | 188.25923 | 0.94625 | -0.00677 | 0.00166 | 0.00007 |
| INSIG1 | 1068 | 0.00213 | 0.0017 | 1.00213 | 0.99879 | 1.00549 | 1 | 767.40179 | 1 | 0.00203 | 0.00055 | 0.00022 |
| DUSP2 | 42 | 0.10651 | 0.03684 | 1.11239 | 1.03491 | 1.19567 | 1 | 79.37174 | ＜0.00001 | 0.01485 | 0.004 | 0.00063 |
| RPS27L | 352 | -0.00326 | 0.00854 | 0.99674 | 0.98019 | 1.01357 | 1 | 234.29962 | 1 | -0.00367 | 0.00108 | 0.00078 |
| BCL2A1 | 561 | -0.02858 | 0.00469 | 0.97183 | 0.96293 | 0.9808 | 0.00045 | 555.89616 | 0.54102 | 0.00201 | 0.00096 | 0.03578 |
| MMP9 | 780 | 0.0088 | 0.00555 | 1.00883 | 0.99792 | 1.01987 | 1 | 439.09676 | 1 | -0.00135 | 0.00066 | 0.03984 |
| KDM6B | 318 | 0.0051 | 0.0102 | 1.00511 | 0.98521 | 1.02541 | 1 | 608.27242 | ＜0.00001 | -0.0031 | 0.00151 | 0.04125 |
| FOSB | 134 | -0.31423 | 0.01786 | 0.73035 | 0.70523 | 0.75637 | 0.00213 | 130.57646 | ＜0.00001 | 0.00493 | 0.00305 | 0.10831 |
| CD1B | 1801 | 0.36623 | 0.07306 | 1.44245 | 1.28412 | 1.70933 | 0.01814 | 2328.61143 | 0.31483 | -0.00187 | 0.00059 | 0.15149 |
| ARL5B | 317 | -0.0616 | 0.01599 | 0.94026 | 0.91124 | 0.9702 | 0.1691 | 127.15811 | 1 | -0.00332 | 0.00234 | 0.15835 |
| PDE4B | 1033 | -0.11718 | 0.04293 | 0.88943 | 0.81729 | 0.96707 | 0.00632 | 1096.5167 | 0.07984 | 0.0042 | 0.0007 | 0.23947 |
| IER3 | 1644 | 0.00401 | 0.00551 | 1.00402 | 0.99324 | 1.01492 | 1 | 10523.64095 | ＜0.00001 | -0.00208 | 0.00186 | 0.26399 |
| NFKBIA | 1989 | -0.0741 | 0.03335 | 0.92863 | 0.86989 | 0.99142 | 0.02613 | 10346.3435 | 0.54322 | -0.0059 | 0.00102 | 0.5272 |
| NR4A3 | 46 | -0.02411 | 0.0455 | 0.97618 | 0.89289 | 1.06724 | 1 | 43.58558 | 0.53194 | 0.00416 | 0.0072 | 0.56676 |
| NR4A2 | 499 | -0.06564 | 0.00737 | 0.93647 | 0.92303 | 0.95009 | 0.32125 | 350.88894 | 1 | -0.00059 | 0.00128 | 0.06473 |
| NR4A1 | 73 | -0.11501 | 0.03067 | 0.89135 | 0.83936 | 0.94657 | 0.50955 | 55.11005 | 0.93041 | -0.00176 | 0.00477 | 0.71422 |
| STX11 | 358 | -0.04786 | 0.00722 | 0.95326 | 0.93987 | 0.96685 | ＜0.00001 | 263.26051 | 0.02 | -0.00026 | 0.00078 | 0.00742 |
| NLRP3 | 1810 | -0.06944 | 0.03311 | 0.93293 | 0.90625 | 1.03185 | 0.01822 | 2026.54252 | 0.08396 | -0.00244 | 0.00051 | 0.77734 |
| NUB1 | 269 | 0.00707 | 0.00772 | 1.0071 | 0.99197 | 1.02245 | 1 | 205.97638 | 0.99807 | -0.00029 | 0.0014 | 0.83713 |
| DNAJA1 | 95 | -0.05519 | 0.01992 | 0.94631 | 0.91008 | 0.98398 | 1 | 37.71171 | 1 | 0.00054 | 0.0031 | 0.86242 |
| TXN | 969 | -0.00478 | 0.00404 | 0.99523 | 0.98739 | 1.00314 | 1 | 601.62086 | 1 | -0.00003 | 0.0005 | 0.95628 |
| NFKBID | 83 | -0.22778 | 0.02585 | 0.7963 | 0.75696 | 0.83768 | 0.25571 | 20.71417 | 1 | 0.00028 | 0.00709 | 0.09686 |
| HERPUD1 | 900 | 0.00427 | 0.00358 | 1.00428 | 0.99726 | 1.01135 | 1 | 969.75394 | 0.05026 | -0.00531 | 0.00089 | ＜0.00001 |
| CKS2 | 1162 | 0.03921 | 0.00384 | 1.03999 | 1.03218 | 1.04785 | ＜0.00001 | 1196.69764 | 0.22749 | 0.00617 | 0.00052 | ＜0.00001 |
| CREM | 769 | -0.14745 | 0.00446 | 0.8629 | 0.85539 | 0.87049 | ＜0.00001 | 740.03117 | 0.75973 | 0.0057 | 0.00087 | ＜0.00001 |
| NFE2L2 | 434 | -0.18414 | 0.01184 | 0.83182 | 0.81273 | 0.85135 | ＜0.00001 | 321.36045 | 0.99998 | 0.01378 | 0.00129 | ＜0.00001 |
| LAMP3 | 684 | -0.00824 | 0.00616 | 0.99179 | 0.9799 | 1.00384 | 1 | 539.57768 | 0.99998 | -0.01365 | 0.00101 | ＜0.00001 |
| ICAM1 | 771 | 0.06727 | 0.00848 | 1.06958 | 1.05195 | 1.08751 | 0.00323 | 1210.15381 | ＜0.00001 | 0.00527 | 0.00112 | ＜0.00001 |
| PLAUR | 701 | 0.04704 | 0.0106 | 1.04816 | 1.02662 | 1.07016 | 0.01303 | 1503.95627 | ＜0.00001 | 0.0147 | 0.00144 | ＜0.00001 |
| MCL1 | 1300 | -0.22361 | 0.00776 | 0.79963 | 0.78756 | 0.81189 | 0.02162 | 5696.16056 | ＜0.00001 | -0.01464 | 0.00176 | ＜0.00001 |
| NFKBIZ | 1634 | -0.02096 | 0.00516 | 0.97926 | 0.96941 | 0.98921 | 0.06999 | 4765.32181 | ＜0.00001 | -0.01032 | 0.00079 | ＜0.00001 |
| FTH1 | 845 | -0.00117 | 0.00545 | 0.99884 | 0.98823 | 1.00956 | 1 | 2353.33872 | ＜0.00001 | 0.00396 | 0.00076 | ＜0.00001 |
| CD55 | 2819 | 0.00296 | 0.00132 | 1.00297 | 1.00038 | 1.00556 | 1 | 6093.83365 | ＜0.00001 | -0.00415 | 0.00041 | ＜0.00001 |
| IL1B | 319 | 0.13724 | 0.01374 | 1.1471 | 1.11662 | 1.17841 | ＜0.00001 | 616.94738 | ＜0.00001 | 0.01496 | 0.00089 | ＜0.00001 |
| GPR183 | 254 | -0.1364 | 0.01884 | 0.87249 | 0.84086 | 0.90531 | ＜0.00001 | 548.48634 | ＜0.00001 | -0.01018 | 0.00223 | ＜0.00001 |
| PLEK | 1779 | -0.01107 | 0.00188 | 0.98899 | 0.98534 | 0.99265 | ＜0.00001 | 2812.2297 | ＜0.00001 | 0.0097 | 0.00043 | ＜0.00001 |
| TNFAIP3 | 248 | -0.19205 | 0.02493 | 0.82527 | 0.78591 | 0.86659 | ＜0.00001 | 677.50372 | ＜0.00001 | -0.02511 | 0.00348 | ＜0.00001 |
| REL | 713 | -0.22743 | 0.01183 | 0.79658 | 0.77833 | 0.81526 | ＜0.00001 | 2992.63465 | ＜0.00001 | -0.01039 | 0.00134 | ＜0.00001 |
| CSRNP1 | 599 | -0.01252 | 0.00788 | 0.98756 | 0.97241 | 1.00293 | 1 | 439.29262 | 0.999999782 | 0.00633 | 0.0013 | ＜0.00001 |
| PPIF | 445 | -0.18137 | 0.04012 | 0.83412 | 0.77092 | 0.90222 | 0.00612 | 508.40626 | 0.45844 | -0.00755 | 0.00149 | 0.70326 |

| **Table S9: Results of Bayesian Colocalization Analysis** | | | | | |
| --- | --- | --- | --- | --- | --- |
| **id** | **PP.H0** | **PP.H1** | **PP.H2** | **PP.H3** | **PP.H4** |
| PDE4B | 4.12E-34 | 0.588018042 | 2.86E-34 | 0.40730484 | 0.004677117 |
| NLRP3 | 6.86E-244 | 0.756062685 | 2.07E-244 | 0.228485247 | 0.015452068 |
| PPIF | 9.72E-61 | 0.673917208 | 3.50E-61 | 0.242748523 | 0.08333427 |
| NFKBIA | 8.31E-32 | 0.605341181 | 2.38E-32 | 0.173196662 | 0.221462157 |
| CD1B | 1.18E-11 | 0.002171442 | 1.71E-10 | 0.030451025 | 0.967377532 |
